# Selective Activation of Dynamics in Kinetically Frozen Supramolecular Polymer Bottlebrush Assemblies

**DOI:** 10.1002/smll.202505481

**Published:** 2025-09-15

**Authors:** Hans F. Ulrich, Tobias Klein, Ziliang Zhao, Zoltán Cseresnyés, Pablo Carravilla, Ruman Gerst, Alina Kasberg, Frederic P. Scharfenberg, Marc Thilo Figge, Christian Eggeling, Johannes C. Brendel

**Affiliations:** ^1^ Laboratory of Organic and Macromolecular Chemistry (IOMC) Friedrich‐Schiller‐University Jena Humboldtstraße 10 07743 Jena Germany; ^2^ Jena Center for Soft Matter (JCSM) Friedrich‐Schiller‐University Jena Philosophenweg 7 07743 Jena Germany; ^3^ Institute for Applied Optics and Biophysics Friedrich Schiller University Jena 07743 Jena Germany; ^4^ Leibniz Institute of Photonic Technology e.V. member of the Leibniz Centre for Photonics in Infection Research (LPI) 07745 Jena Germany; ^5^ Applied Systems Biology Leibniz Institute for Natural Product Research and Infection Biology Hans Knöll Institute (HKI) 07743 Jena Germany; ^6^ Institute of Microbiology Faculty of Biological Sciences Friedrich‐Schiller‐University Jena 07743 Jena Germany; ^7^ Macromolecular Chemistry I University of Bayreuth Universitätsstr. 30 Bayreuth 95447

**Keywords:** automated image analysis, dynamic exchange, nanofibers, self‐assembly, STED‐microscopy, supramolecular assembly

## Abstract

Supramolecular assemblies are typically characterized by their dynamic nature due to the comparable weak non‐covalent interactions. While these properties confer adaptability, stability issues may limit application in areas such as drug delivery or tissue engineering. Here, supramolecular assemblies of amphiphilic polymers containing benzenetrispeptide and benzenetrisureas motifs are inherently stable and non‐dynamic at ambient conditions is shown, but dynamic exchange can be selectively activated. Stimulated emission depletion microscopy combined with automated image analysis revealed no dynamic exchange between complementary labeled fibers independent of the length of the hydrophobic domains at ambient conditions in pure water for several days. Competitive solvent addition facilitates dynamic exchange but compromises stability. Raising the temperature of the samples in pure water to 60 °C, however, induces similar dynamics while fiber stability is maintained. The amphiphilic character, in combination with the strong hydrogen bonds, seems to endow these supramolecular polymer brushes with unique switchable dynamics.

## Graphical abstract

Static at rest—dynamic on demand: Although supramolecular polymers are known for their dynamic nature, this study investigates the dynamics of amphiphilic assemblies based on polymer‐modified benzenetrispeptide and benzenetrisurea motifs. These structures remain frozen under ambient conditions but can be selectively triggered by changes in temperature or solvent. This unique “static at rest — dynamic on demand” behavior has promising implications for biomedical applications.

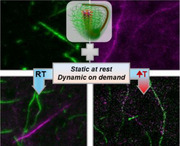

## 1 Introduction

Supramolecular chemistry was defined by J. M. Lehn as “chemistry beyond the molecule” and relies on non‐covalent interactions such as hydrogen bonds, van‐der‐Waals forces, electrostatic, or π‐π interactions, which are considered inherently dynamic.^[^ ^1^, ^2^, ^3^, ^4^, ^5^, ^6^ ^]^ Their dynamic behavior stems from the weaker nature of intermolecular bonds and is also the reason why the common supramolecular motifs are normally able to form multiple intermolecular bonds.^[^ ^7^, ^8^, ^9^ ^]^ The motifs that form these bonds are not only useful for recognition, but also for the organisation into more complex structures.^[^ ^10^, ^11^, ^12^, ^13^ ^]^

Through advances in recent decades, supramolecular concepts have been integrated into polymer chemistry, giving rise to supramolecular polymers.^[^ ^14^ ^]^ These typically rely on either π–π interactions or hydrogen bonding.  Common systems based on π−π interactions include perylenes, cyclophanes and linear rigid aromatic units.^[^ ^5^, ^15^, ^16^, ^17^, ^18^ ^]^ Hydrogen‐bond based systems often contain peptide motifs, which are also utilised by nature to form one dimensional (1D) fibres. These systems have the ability to form hydrogen bonds, in particular β‐sheets which have been the focus of a plethora of investigations over the last decades.^[^ ^19^, ^20^ ^]^ An interesting example is cyclic peptides, which consist of alternating l and d amino acids and aggregating into large nanotubes.^[^ ^21^, ^22^ ^]^ Another hydrogen bond‐based motif is benzenetrisamides (BTA), which form fibrous aggregates through directional amid‐amid hydrogen bonding and have been thoroughly studied by the groups of Meijer or Schmidt.^[^ ^23^, ^24^, ^25^, ^26^, ^27^ ^]^

In recent years, supramolecular polymerization has advanced to the assembly of macromolecular building blocks into fibrous structures. Amphiphilic systems, which combine hydrophilic polymers with hydrophobic supramolecular motifs, have been shown to form fibrillar aggregates in water, resembling supramolecular polymer bottlebrushes. The aforementioned linear and cyclic peptide motifs proved to be good exemplary systems for the formation of such structures.^[^ ^28^, ^29^, ^30^ ^]^ For these systems, the attachment of hydrophilic polymers did not impede the aggregation, but lead to the formation of a comb or bottlebrush‐like structure. Similar behavior has been observed for π–π‐stacking systems based on rigid aromatics and perylenes.^[^ ^31^, ^32^, ^33^, ^34^ ^]^ Recently, we reported benzenetrisurea (BTU) and benzenetrispeptide (BTP) building blocks which can form large fibres in water driven by hydrophobic interactions and directing hydrogen bonds.^[^ ^35^, ^36^ ^]^ Our investigations showed that the assembly behavior for the BTP systems is pathway dependent and that a solvent switch is needed to obtain extended fibers.

The dynamic nature of supramolecular interactions generally extends to supramolecular polymers. However, in aqueous environments, hydrophobic effects can significantly reduce exchange dynamics.^[^ ^37^, ^38^, ^39^ ^]^ This is particularly relevant for amphiphilic assemblies such as block copolymer micelles, where parameters like core hydrophobicity, composition, and temperature influence unimer exchange.^[^ ^40^, ^41^, ^42^, ^43^, ^44^, ^45^, ^46^ ^]^ Certain material characteristics may ultimately slow dynamics to such an extent that they cannot be observed in realistic time scales, resulting in a frozen or kinetically trapped state.^[^ ^47^ ^]^ While only a small number of supramolecular systems have been studied explicitly in water, many exhibit pathway‐dependent behaviour, which is a strong indication of kinetic trapping.^[^ ^37^, ^39^, ^48^, ^49^, ^50^ ^]^ The BTA motif represents an exception in this regard, given the thorough investigations by Meijer et al. who visualized the exchange between fibers by fluorescence resonance energy transfer (FRET) and super‐resolution microscopy.^[^ ^51^, ^52^ ^]^ Another interesting example for a macromolecular building block comprising cyclic peptides is given by Rho et al., where it is demonstrated that an additional hydrophobic block impedes dynamic exchange.^[^ ^53^ ^]^

In this work, we studied the intrinsic dynamics of our supramolecular polymer bottlebrushes based on BTU or BTP building blocks. We investigated if either a slow dynamic behavior or a kinetically trapped state is present in our systems. Therefore, different building blocks were analysed, which comprise either urea (BTU) or diamide units (BTP) as hydrogen bonding sites, and we varied the hydrophobic spacer length (C6‐C12) to elucidate any impact of the hydrophobic moieties on the dynamics. We implemented two complementary fluorescent dyes in our system and used stimulated emission depletion (STED) microscopy to monitor the dynamic exchange. An automated image analysis pipeline was further developed to determine the component distribution within and along the fibers based on color intensity In addition, we conducted NMR studies to analyze the aggregation state of selected building blocks.

## 2 Results and Discussion

### 2.1 Integrating Dye Labels into the Building Block Motifs

**Figure 1:**
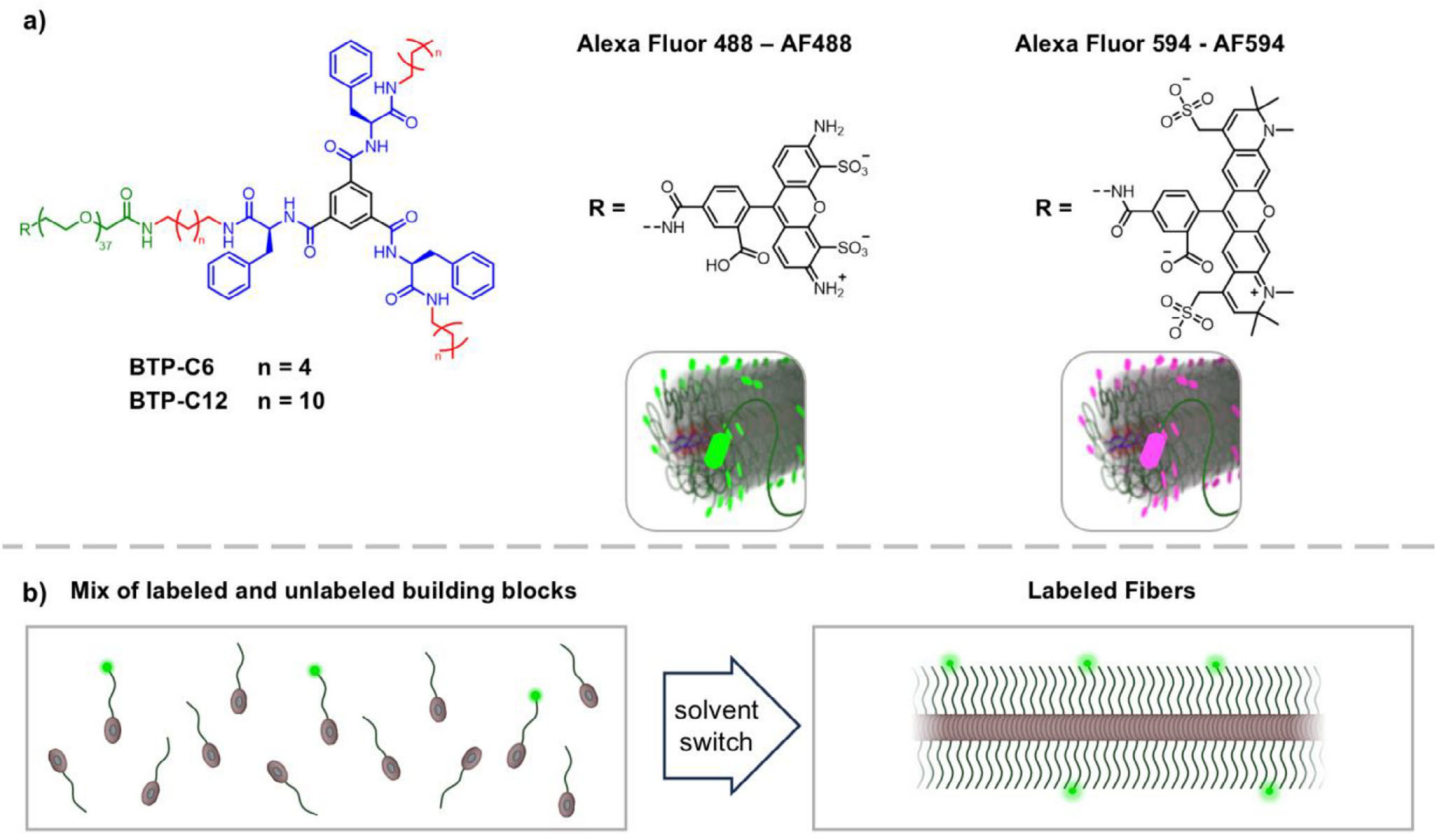
a) Overview of the applied benzenetrispeptide (BTP) and benzenetrisurea (BTU) building blocks with poly(ethylene oxide) (PEO) of ≈2 kg mol^−1^. For confocal microscopy and FRET measurements, the polymer end groups were modified with Alexa Fluor 488 and Alexa Fluor 594. b) Schematic representation illustrates the process of obtaining the labeled fibers using unmodified (grey) and labeled building blocks (red).

Based on our previous investigations, we have selected building blocks for this study which all form large fibers or supramolecular polymer bottlebrushes, respectively.^[^ ^36^, ^54^ ^]^ These are based on either a BTU unit or on a more variable BTP unit (coined core), which are surrounded by three alkyl chains (coined spacer). To obtain the amphiphilic character, one of these spacers is conjugated with a hydrophilic polymer, in this case poly(ethylene oxide) (PEO) of ≈2 kg mol^−1^. It is necessary to note that the BTU motif requires dodecyl chains (C12) as hydrophobic spacers to enable fiber formation. The BTP motif features a more robust aggregation into fibers even if only a hexyl spacer (C6) is applied.^[^ ^35^, ^54^ ^]^ An overview of all prepared systems and their different functionalization is given in **Figure** **1**a.

### 2.2 Screening Visualization Techniques and Sample Preparation

We started by screening methods applied for investigating the dynamics of the comparable BTA system by Meijer et al. They were able to monitor and confirm the dynamic behavior of their fibers via a hydrogen/deuterium exchange in mass spectrometry (MS) or stochastic optical reconstruction microscopy (STORM) imaging.^[^ ^37^, ^51^ ^]^ Another method is based on FRET, which is frequently applied for studying the dynamics of supramolecular systems.^[^ ^37^, ^39^, ^55^, ^56^, ^57^ ^]^ We first intended to use matrix‐assisted laser desorption/ionization time of flight mass spectrometry (MALDI‐ToF‐MS) and tried to evaluate the dynamics by monitoring of the hydrogen/deuterium‐exchange in deuterated water (D_2_O) over time. However, the initiated exchange appeared to be disturbed by a rapid proton exchange with the applied matrix in MALDI, which was reported by others previously.^[^ ^58^ ^]^ As a consequence, we turned our attention to FRET and, therefore, modified the polymer chain ends not connected to supramolecular building blocks with complementary dyes known to form strong FRET pairs (Cy3 and Cy5). Unfortunately, no sufficient signal could be observed when mixing both building blocks as positive control experiment, even though a large majority of chains were modified (see Figure S1a, Supporting Information). We assume that the extended distance between the dye placed at the outer corona and the fiber core by the PEO chain causes a too large separation of the dye molecules for a sufficient FRET signal. An attachment closer to the core unit was abandoned due to interference with the aggregation.

**Figure 2:**
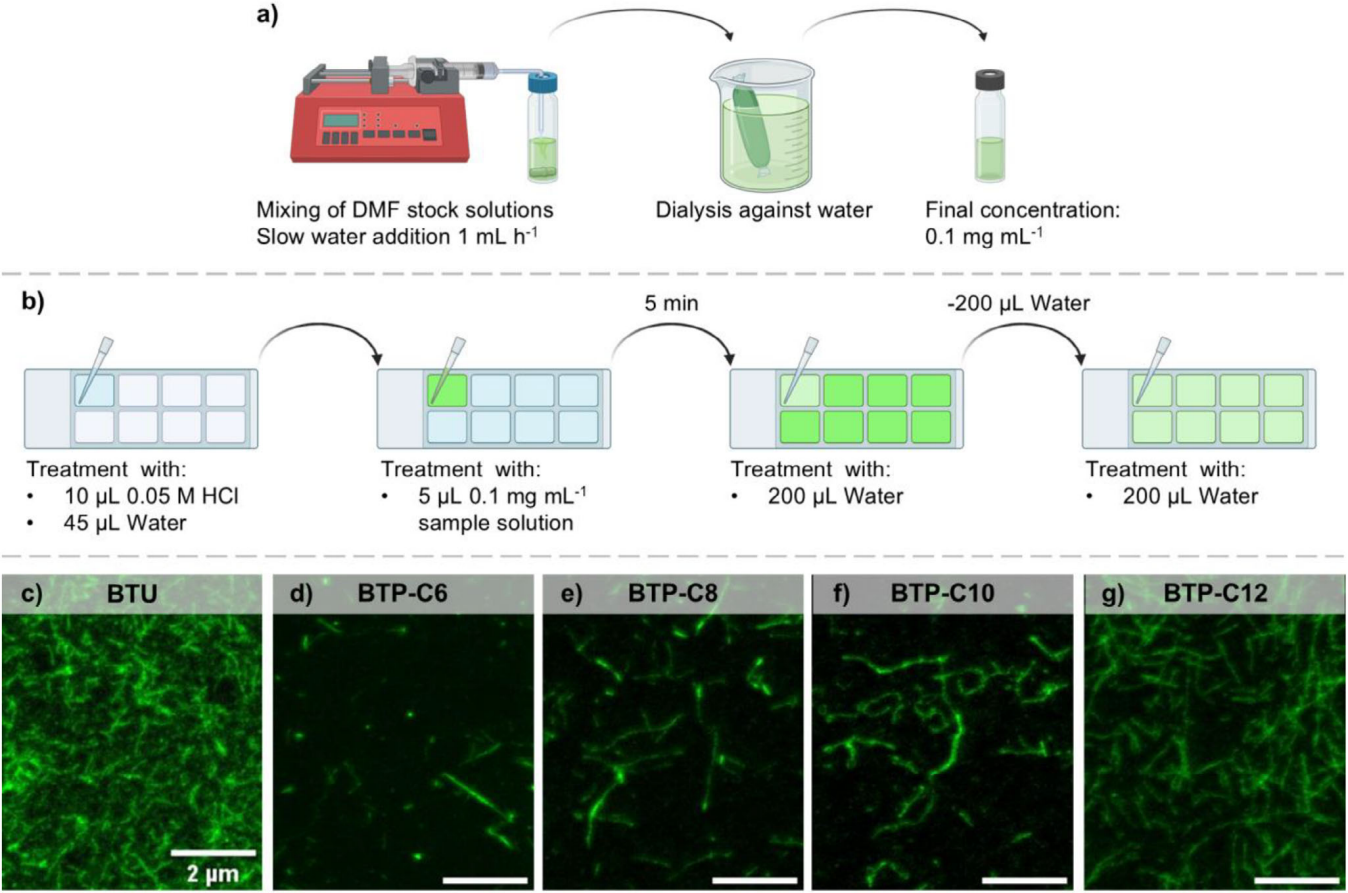
Schematic representation of the solvent switch a) and sample preparation for STED‐measurements b). STED‐microscopy images of BTP and BTU samples containing only AF488 (green) dye in the solution c–g).

As an alternative, we further focused on microscopy methods, where fibers were again modified with two complementary dye labels to visualize a potential dynamic exchange of units between the fibers. Therefore, we evaluated STED‐microscopy, which proved to be a suitable analysis method when compared to confocal microscopy (see Figure S2, Supporting Information).^[^ ^59^, ^60^ ^]^ Similar to the initial tested FRET study, we prepared building blocks labeled with either Alexa Fluor 488 (AF488) or Alexa Fluor 594 (AF594) starting from a bifunctional PEO and prepared labeled fibers using a mix of labeled and unlabeled building blocks (Figure 1b). An initial obstacle for our investigation was the limited adhesion of our fibers to the glass substrate, as approaches comprising drying of the samples caused severe artifacts due to aggregation and crystallization of the PEO chains. A viable approach proved to be a treatment of the surface with hydrochloric acid to induce partial protonation and a positive surface charge. The fibers are slightly negatively charged due to the anionic groups on the dyes, inducing sufficient but weak interaction with cationic surfaces to make the fibers adhere to the surface. After the surface treatment, a neutral solution of the samples was added, and any unbound materials were washed off, while all bound fibers could be maintained in a fully hydrated state by maintaining a water film on top. An overview of the preparation procedure is given in **Figure** **2**a. Based on the initial microscopy tests, we adjusted the dye content in the fibers by mixing 1 mol% of AF488 or 2 mol% of AF594 labeled building block, respectively, into the unlabeled building blocks. However, despite these efforts, which should lead to a similar density of labels on the fibers, we observed in fluorescence spectroscopy that the fluorescence intensity is significantly lower for the AF594 labeled materials, which might cause some deviations in the brightness of the two different fibers (see Figure S1b,c, Supporting Information). The results of an initial screening of the nanostructures formed from the different building blocks (BTU and BTP) are given in Figure 2b for the AF488 labeled materials (for AF594 labeled molecules see Figure S3, Supporting Information).

In all cases, fibers with lengths of up to several micrometers can be observed which is in accordance with our previous observations in cryo‐TEM measurements.^[^ ^36^, ^54^ ^]^ The presented sample preparation and STED‐microscopy therefore, appears suitable to image these fibers and analyze an exchange between them. In this regard, we could also verify that the previously not reported BTP‐C8 (octyl spacer) and BTP‐C10 (decyl spacer) also form similar fibers as the BTP‐C6 and BTP‐C12.

### 2.3 Investigating Dynamic Exchange Over Time

**Figure 3:**
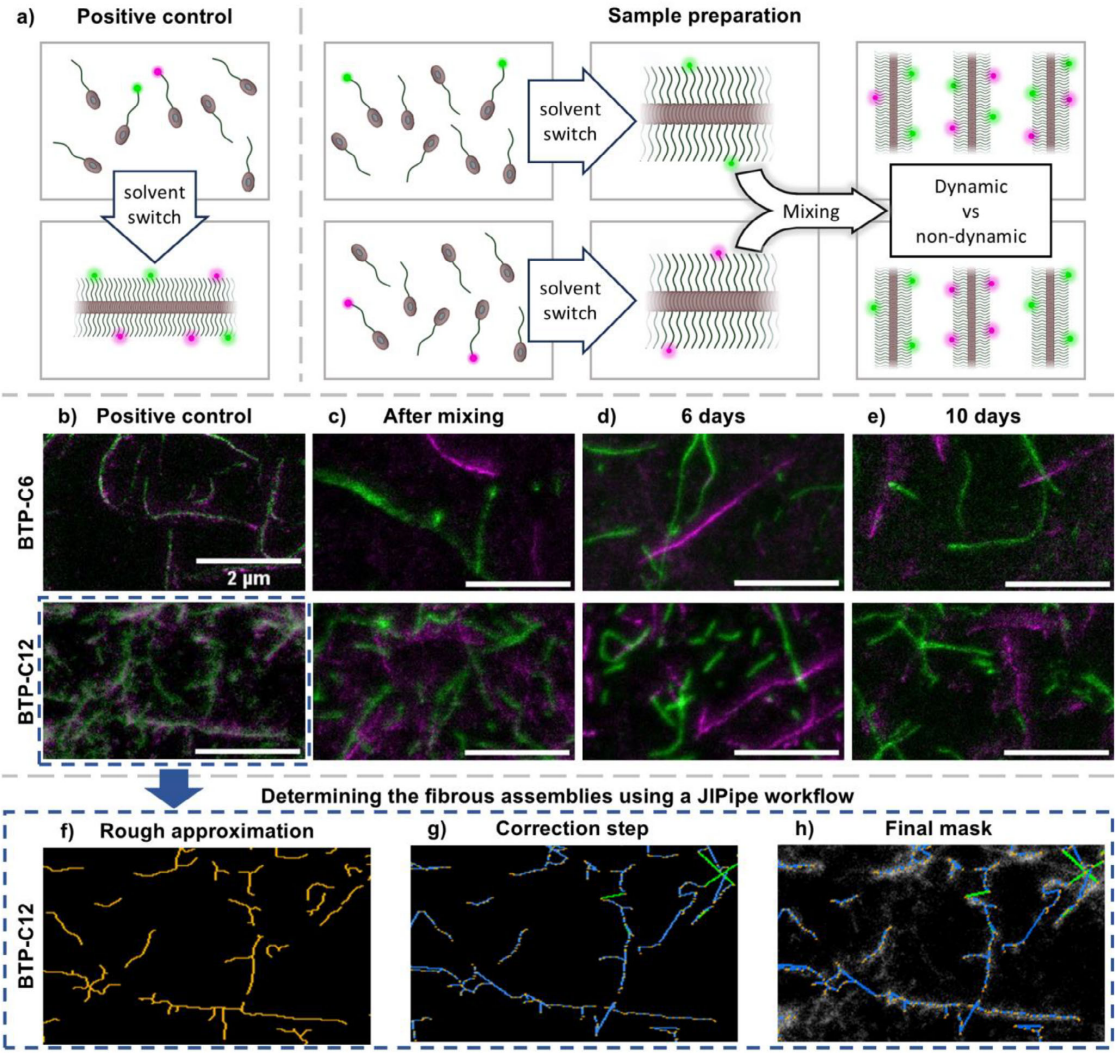
Schematic representation of the sample preparation for the positive control and samples a) and STED‐microscopy images of BTP‐C6 and BTP‐C12 fibers containing AF488 (green) and AF594 (magenta) dyes. For the positive control, the labeled building blocks were mixed before assembly b). Otherwise, the individually labeled fibers were assembled separately, mixed post‐assembly, and measured after being kept mixed for 6 and 10 days c–e). The final concentration of each mixed solution was 0.1 mg mL^−1^. See Figures S5 and S8 (Supporting Information) for individual channels. For the mask generation process a JIPipe workflow was applied to create the first rough approximation of the filaments f, orange) to be fitted on the individual molecules, using the merged images. After the correction steps applied to these filaments g, correction elements in green, first approximation segments in blue), the final mask followed the original images well h). See Figures S13–S18 (Supporting Information) for the full process.

The imaging of the fibers in STED‐microscopy set the basis for our further investigations of the fiber dynamics, in which case we started with the more hydrophobic motifs BTU and BTP‐C12. To monitor the exchange between the fibers, individual solutions of fibers labeled with either AF488 or AF594 were prepared separately and subsequently combined (**Figure** **3**a). The combined solutions were then analyzed by STED‐microscopy at different time points to visualize any exchange of units between the differently labeled fibers of the same system over time. For comparison, we also prepared mixed fibers as a positive control where both AF488 and AF594 labeled building blocks were combined in dimethylformamide (DMF) before they were assembled into fibers by a solvent switch (Figure 3b; Figures S4–S8, Supporting Information). The resulting images revealed a clear overlap of the two signals (AF488 and AF594) and represent a positive control.

Analyzing the combined solutions of individually prepared assemblies, clearly separated fibers can be identified in the images, which were recorded immediately after combining the solutions and sample preparation (Figure 3c; Figures S5 and S8 for individual channels, Supporting Information). Based on previous reports on exchange of supramolecular systems, we first looked at the systems with the highest hydrophobic shielding (BTP‐C12 and BTU (Figure S4, Supporting Information)) and left the samples to mix for up to 1 h at room temperature.^[^ ^37^, ^53^ ^]^ Both samples showed no sign of mixing, nor could the formation of block‐like structures be observed in the recorded images. Instead, the differently labeled fibers remain separated throughout the observed areas, which contrasts with the above‐mentioned previously reported systems.^[^ ^37^, ^53^ ^]^ We continued the experiment with both systems over several days, as we considered that the dynamics might be very slow or early traces of exchange might be overlooked in the experiments. However, for all tested building blocks we were not able to identify any mixing or exchange between the differently labeled fibers after up to 10 days (Figure 3d,e, see Figures S4 and S5 for individual channels, Supporting Information).

In consequence, we investigated the impact of shortening the hydrophobic spacers, and hence a reduction of the hydrophobic shielding of the hydrogen bonds in the system, next, and anticipated an increased dynamic behavior of the fibers. While in the case of the BTU a C12 spacer is required for fiber assembly, the BTP motif has been demonstrated to form fibers even with a shorter C6 spacer.^[^ ^36^, ^54^ ^]^ We therefore focused on the dynamics of the BTP systems with gradually shorter alkyl spacers (BTP‐C10, BTP‐C8 and BTP‐C6). The STED images of similarly prepared BTP‐C6 samples is given in Figure 3 for different time points (see Figures S6 and S7 for BTP‐C8, BTP C10 and individual channels, Supporting Information). The results show that despite the shortened spacer length and thus decreased hydrophobic shielding, still no indication of any dynamic exchange of building blocks can be observed in all these samples, even after 10 days (Figure 3b; Figures S4–S8, Supporting Information). Although the dyes are considered very stable, we also observed during our long‐term experiments that the fluorescence signal for the AF594 decreases over time. The origin of this decay remains unclear, but we assume that long term storage in solution despite exclusion of light caused some bleaching of the samples.

In addition to the visual control of the samples, we also established an automated image analysis procedure providing a data‐based characterization of the images. Based on the positive control measurements, we therefore established an automated workflow in JIPipe (see Figures S13–S18, Supporting Information) to create a mask of the fibrous assemblies. This workflow consists of two steps. During the first step, a rough approximation of the fibrous assemblies is made using normalized merged images from the two colors (AF488 and AF594) (Figure 3f). This crude mask is then refined by a subsequent correction step (Figure 3g), resulting in the final mask that aligns well with the determined structures in the original image (Figure 3h). However, the results show limits when it comes to areas with overlapping fibers, where the automated tool has difficulties determining all fibers, and which has to be kept in mind.

**Figure 4:**
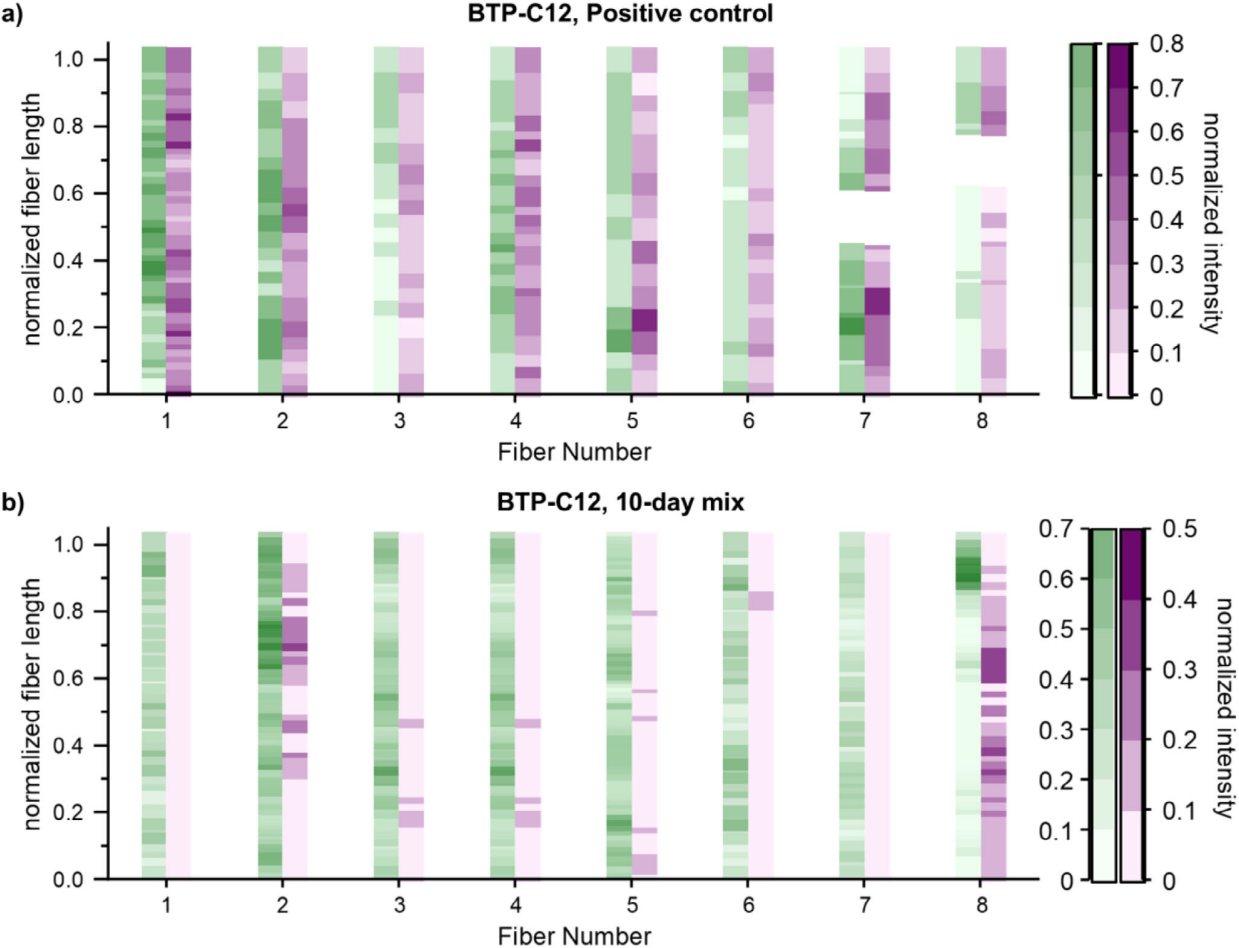
Overview of the relation between the normalized intensities for the AF488 (green) and AF594 (magenta) channels, for multiple points alongside a representative fiber from one image of the BTP‐C12 positive control a) and 10‐day mixed sample b).

The generated mask enables the evaluation of the dye distribution throughout the fibers. In case of the positive control, a homogenous distribution of both dyes throughout the fibers was observable (see Figure S20, Supporting Information). A comparison between the BTP‐C12 positive control (Figure 3b) and the 10‐day mixed sample (Figure 3e) further showed that no exchange between the fibers occurred (**Figure** **4**; Figure S23, Supporting Information). Here, the normalized color intensity is plotted for each automatically determined fiber along the normalized length. The plot reveals that the positive control displays a similar color intensity for both dyes within representative fibers (Figure 4a), which confirms that both labels are present in a similar ratio (see Figure S20 for color intensity plot, Supporting Information). In contrast, the 10‐day mixed sample (Figure 4b) displays higher intensities for one dye per fiber, while the other intensity remains mostly at the lower end of the scale, which equals the background intensity in the images. These results verify that the labels remain separated in individual fibers and support our previous observation that dynamic exchange is absent (Figure S23 for color intensity plot, Supporting Information). It should be noted that in some cases the 10‐day sample displays segments that occur to be oppositely labeled at the fiber termini. Such outcomes are related to smaller fibers being falsely connected by the software. Likewise, potential holes can appear as seen in the positive control. Furthermore, an inspection of the generated mask for a low signal‐to‐noise ratio sample, such as the BTP‐C6 10 day sample, shows the robustness of the fiber identification algorithm of the JIPipe workflow (see Figure S19, Supporting Information).

Applying the automated workflow to the 10‐day mix samples of samples with shorter hydrophobic spacers confirmed that no dynamic exchange occurs for any of these samples (see Figures S23–S25, Supporting Information). We assume that the phenylalanine units play a crucial role in stabilizing the assembly in combination with the six hydrogen bonding sites. Based on these interactions and considering the amphiphilic character of the building block, a very robust assembly is created, which prevents the exchange of unimers between different fibers. Further attempts to shorten the hydrophobic spacers further and introduce an ethyl spacer render the building blocks too hydrophilic and impedes the assembly into fibers.

Overall, these results were surprising when compared to literature‐reported systems, as the majority of supramolecular systems feature a dynamic nature within the given time scales.^[^ ^39^, ^56^, ^58^ ^]^ Although not considered at first, the absence of significant dynamics is further corroborated by previously reported asymmetrical flow field‐flow fractionation (AF4) measurements.^[^ ^35^, ^61^ ^]^ Given a rather large membrane cutoff (10 kg mol^−1^), any unimers should be flushed out by the focus flow and crossflow, which would cause a significant dilution and continuous leaching from the assembled systems, if the system is in a dynamic equilibrium. Given these results, we assumed that the strong hydrophobic interactions and the shielding of the hydrogen bonds in our motifs might cause the absent dynamics of the assemblies.

### 2.4 Dynamic Exchange Induced by a Cosolvent

**Figure 5:**
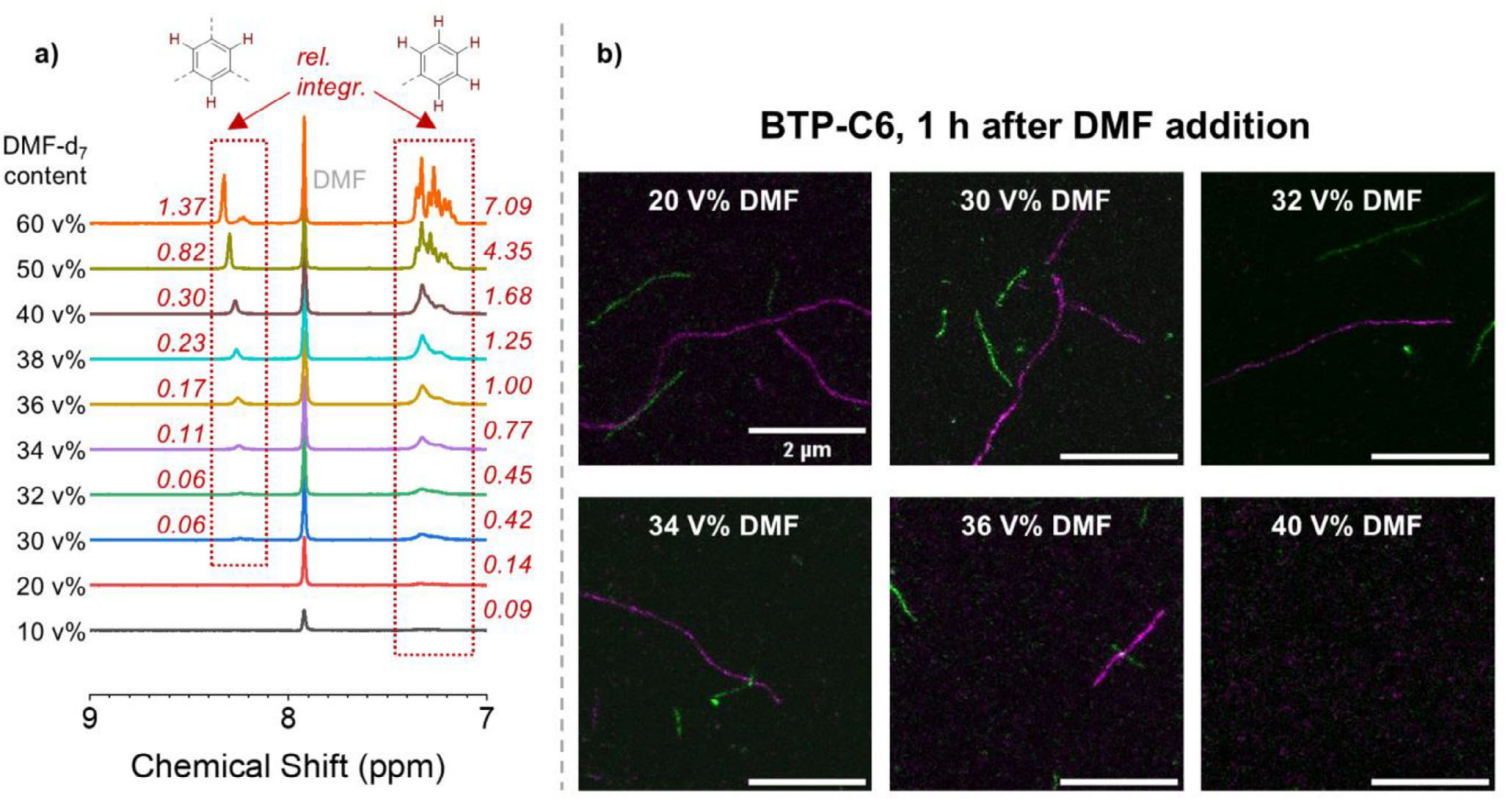
a) ^1^H‐NMR‐Measurments of BTP‐C6 building block, in a solvent mixture of D2O:DMF‐d_7_ with an increasing DMF content, showing the dissolving of the fibrous assemblies through the gradual increase of the aromatic signals for the benzene core and aromatic hydrogen atoms. The relative integrals (rel. integr.) are given and calibrated to an internal trioxane standard. b) STED‐microscopy images of BTP‐C6 fibers containing AF488 (green) and AF594 (magenta) dyes at various solvent mixtures of H_2_O:DMF. The process was stopped through the fiber attachment to the glass surface. The labeled fibers were assembled separately before mixing, and DMF was added 1 h before measurement.

Considering this all‐or‐nothing case (non‐dynamic fibers or no fibers at all) for structural variations, the question remained, if dynamics can be induced by changes of the environment. Therefore, we focused on the systems with the lowest (BTP‐C6) and highest hydrophobic shielding (BTP‐C12). In a first set of experiments, we investigated the impact of an organic co‐solvent on these systems, which are initially used for a controlled assembly via a solvent switch, but can also break the structures once a critical threshold is reached.^[^ ^35^ ^]^ We focused on DMF here, since previous experiments revealed that the assemblies of the motifs with C12 spacer are present over a rather wide range of solvent mixtures with water.^[^ ^35^, ^54^ ^]^ Following the hypothesis that an increased DMF content can induce dynamic exchange of unimers, we first had a closer look at the BTP‐C6 building block, which should be affected most. In this case, we initially tried to evaluate the presence of free unimers of unlabeled systems in solution by ^1^H‐NMR measurements varying the ratio of DMF‐d_7_:D_2_O. While in pure D_2_O, the aromatic signals of the core‐forming units should be suppressed due to aggregation, a stepwise increase of the DMF content should reveal an increasing content of dissolved building blocks once a critical solvent content is reached. Indeed, a significant increase of the signals of the benzene core unit can be observed between 30–40 v% of DMF‐d_7_ (**Figure** **5**), which indicates an increasing dissolution of the fibers.

In consequence, we focused on this DMF concentration range for subsequent exchange experiments in STED‐microscopy. The aim was to reveal whether dynamics are enhanced under these conditions before disassembly occurs. We therefore prepared solutions of separately assembled fibers again, but gradually increased the DMF content up to 40 v%. STED images were recorded after 1 h of mixing focusing on fast exchange processes. Interestingly, again no sign of dynamic exchange can be observed in the images of the BTP‐C6 system (Figure 5b) even if >30 v% of DMF is present. Instead, we observed that the C6 fibers started dismantling at 30 v% DMF, and no further fibers were adsorbed on the glass at ≈40 v% DMF. While in the NMR measurements the samples appear not yet fully dissolved, an increasing content of unimers might impede the fiber adsorption required for STED, or the fibers start to break down into smaller fragments at this DMF content. Nevertheless, the results reveal slow dynamics as long as stable, large fibers are formed. We assume that the rather large number of hydrogen bonds still provide sufficient stability to suppress dynamic exchange on this time scale.

**Figure 6:**
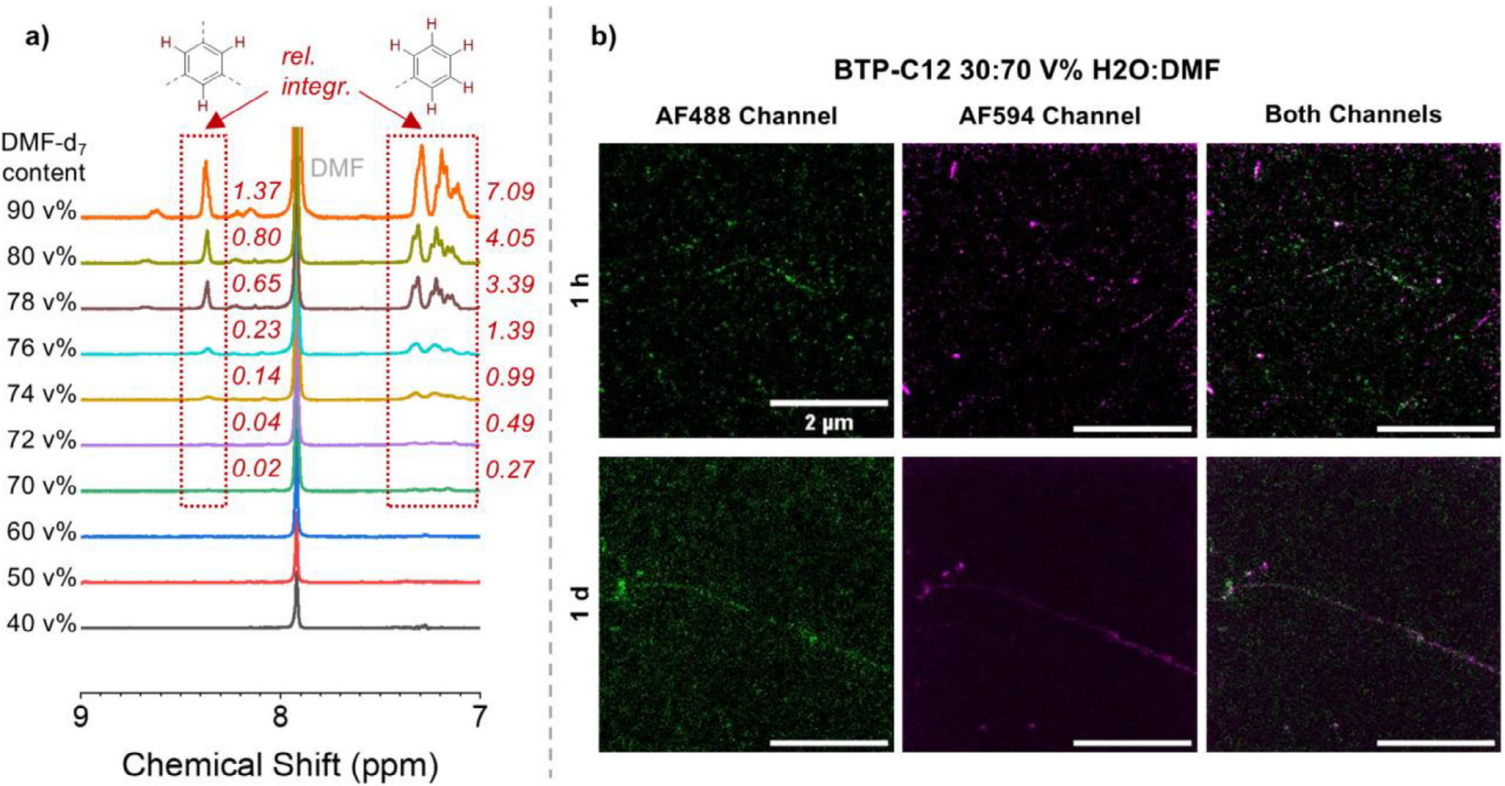
^1^H‐NMR‐measurments of BTP‐C12 building block, in a solvent mixture of D_2_O:DMF‐d_7_ with an increasing DMF content, showing the dissolving of the fibre assemblies a). STED‐microscopy images of BTP fibers containing AF488 (green) and AF594 (magenta) dyes in a solvent mixture of H_2_O:DMF 30:70 after different time periods b). The relative integrals (rel. integr.) are given and calibrated to an internal trioxane standard.

We subsequently analyzed the BTP‐C12 building block, which has previously been proven to form fibers over a wider range of H_2_O:DMF ratios. In accordance, the ^1^H‐NMR measurements revealed a tolerance for DMF of up to ≈70 v% before significant signals for the core can be observed (**Figure** **6**a). We therefore focused on this solvent composition range to first match the close to critical conditions tested for BTP‐C6. Interestingly, an increase to 74 v% of DMF, which is only slightly above the onset of disintegration determined in NMR (70 v% DMF) resulted in a complete absence of fibers (Figure S9, Supporting Information), which contrasts with the BTP‐C6 samples. In consequence, we refrained from a full solvent composition screening but focused on the most relevant composition with 70 v% DMF. In contrast to all previous experiments, the STED images already revealed the first signs of exchange after 1 h (Figure 6b). We then decided to extend the mixing time to 1 d and 7 d, as the overall assembly of fibers remain stable. At these extended time‐points, the fibers become exchanged over their entire length, but their appearance remains more fragmented than comparable positive controls (Figure 3b, i.e., when mixing labeled building blocks before the assembly). This fragmented appearance can be attributed to a number of factors. However, we hypothesize that the fibers become frequently fragmented and reassembled, rather than undergoing the expected exchange of unimers under the given conditions. Nevertheless, in order to corroborate this theory, a more extensive investigation is required.

**Figure 7:**
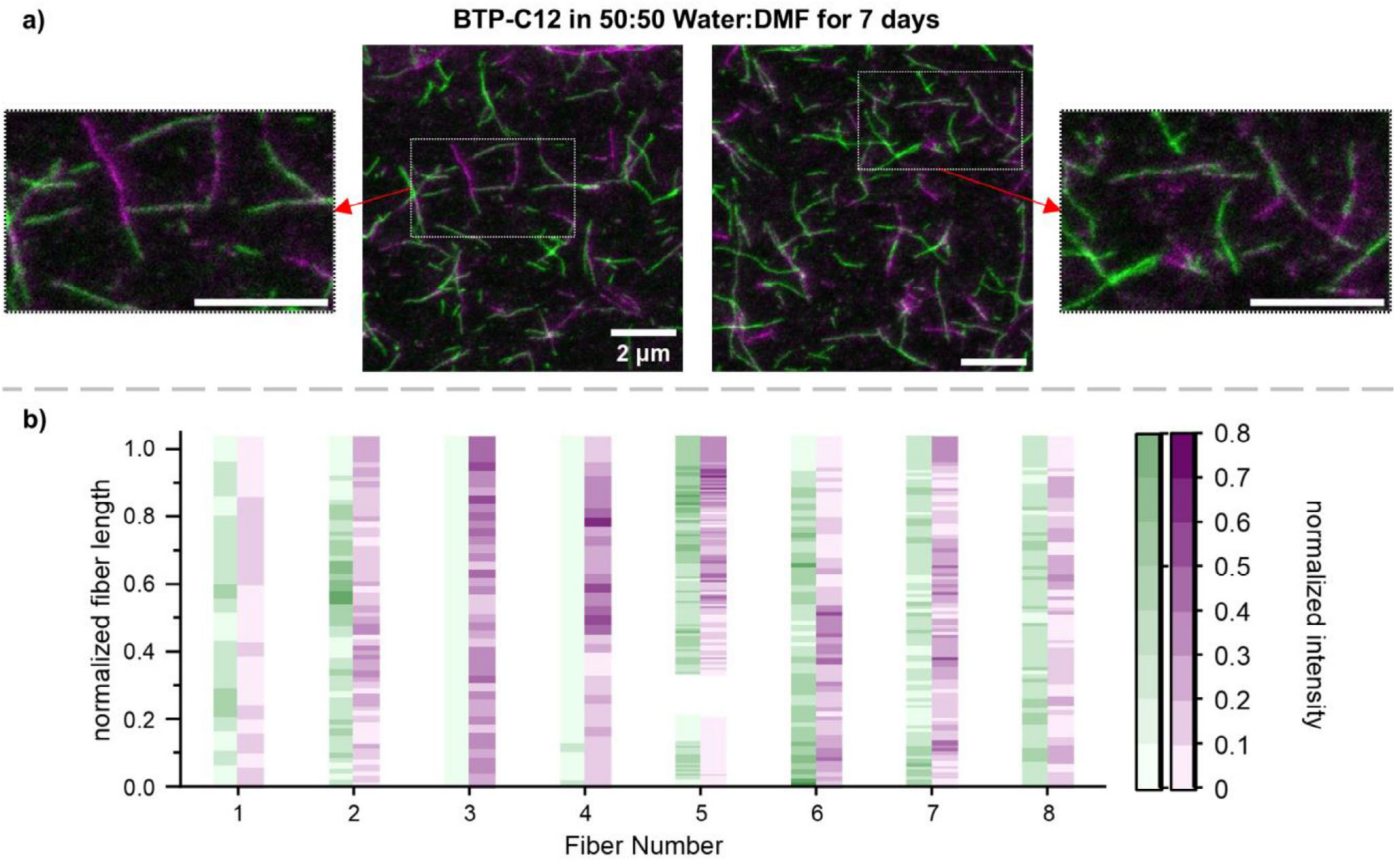
STED‐microscopy images of BTP fibers containing AF488 (green) and AF594 (magenta) dyes stored in a solvent mixture of 50:50 H_2_O:DMF for 7 d (a, see Figure S10 for individual channels, Supporting Information). Overview of the relation between the normalized intensities for the AF488 (green) and AF594 (magenta) channels, for multiple points alongside representative fiber from one image of the BTP‐C12 50:50 Water:DMF sample b).

Intrigued by these results, we additionally analyzed a lower content of DMF of 50 v%, where no traces of unimers can be observed in NMR, but an extended mixing time might still enable a partial fragmentation and reassembly. Indeed, the STED measurements after 7 d displayed several fibers, which featured a block‐like structure (**Figure** **7**a). Utilizing the automated image analysis, we visualize the color distribution per fiber (Figure 7b), which further confirmed the exchange when compared to the 10‐day sample (Figure 4b). Furthermore, the component analysis allowed the visualization of the dyes along the fibers (see Figure S26, Supporting Information). The data revealed that the intensity distribution is not as homogenous along all fibers when compared to the positive sample, which is also confirmed by the corresponding Pearson's R coefficient (see Figure S26, Supporting Information). This result confirms the possibility of fibers connecting at the chain ends and, considering the similar overall size, substantiates the assumption that fragmentation and reassembly occur at increased DMF content and extended time.

### 2.5 Temperature‐Dependent Dynamic Behavior

**Figure 8:**
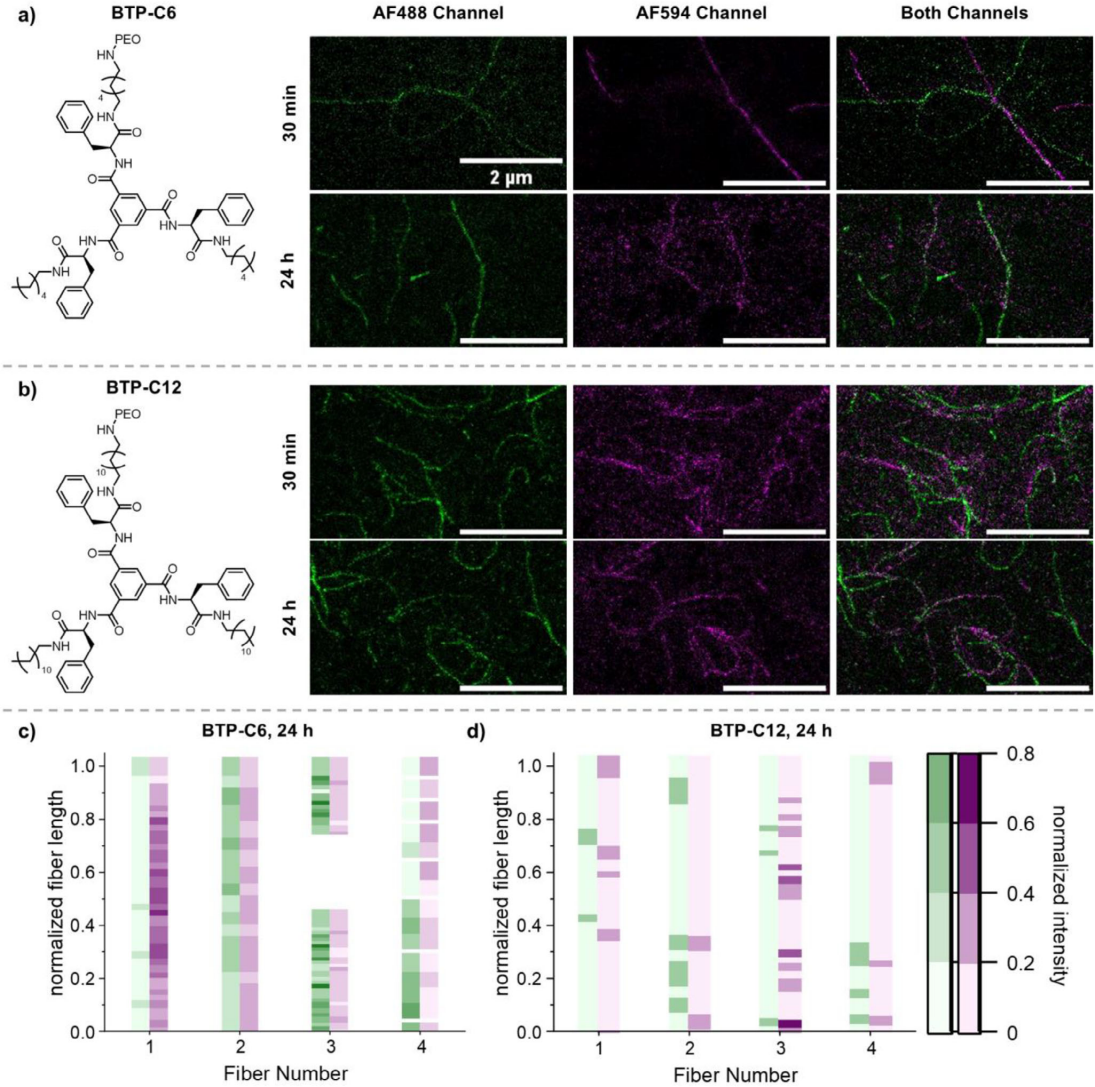
STED‐microscopy images of BTP‐C6 a) and BTP‐C12 b) fibers containing AF488 (green) and AF594 (magenta) dyes that were incubated as 60 °C for 30 min and 24 h. For full‐size images, see Figures S10 and S11 (Supporting Information). Overview of the relation between the normalized intensities for the AF488 (green) and AF594 (magenta) channels, for multiple points alongside a representative fiber from one image of the 24 h tempered BTP‐C6 c) and BTP‐C12 sample d).

In a second set of experiments, we investigated the effect of elevated temperature on the exchange between the fibers, which has previously been shown to induce enhanced dynamics in supramolecular systems as well as amphiphilic block copolymers.^[^ ^50^, ^62^ ^]^ In this case, we focused on the two interesting building blocks BTP‐C6 and BTP‐C12. We combined solutions of differently labeled fibers incubated for different time periods up to 24 h at 60 °C. The temperature was chosen due to literature reports on amphiphilic block copolymers, where an increase in exchange rates could be observed at 60 °C.^[^ ^46^ ^]^ The corresponding STED images for 30 min and 24 h are displayed in **Figure** **8** while additional images for 10 and 60 min are given in the SI (Figures S11 and S12, Supporting Information).

Despite the absence of any solvent, the elevated temperatures triggered an exchange between the differently labeled fibers, which is in stark contrast to the same samples at room temperature, where no signs of dynamics, even at much longer time scales can be observed. First fibers with both dyes present become already apparent at short incubation times of 10 and 30 min, but the degree of mixing becomes significantly enhanced for the samples that were incubated for 24 h. Again, small, fragmented structures can be observed, but also sections in fibers, which appear more uniform in their mixing. This becomes particularly apparent when the samples are incubated for 24 h, where some fibers with a predominant label become infiltrated by the other building block. The observed mixing of the fibers could also be confirmed through our automated image analysis (Figure 8c,d) which confirmed an increase of complementary color intensities in fibers with a predominating label.

## 3 Conclusion

In this work, we investigated the dynamic behavior of our chosen benzene‐1,3,5‐trisurea (BTU) and benzene‐1,3,5‐trispeptid (BTP) motifs in water. After failed attempts to analyse the dynamics of these systems via hydrogen/deuterium exchange using MALDI‐MS and spectroscopic methods based on FRET, we found STED‐microscopy to be a suitable method for our investigations. Therefore, building blocks labeled with AF488 and AF594 were synthesized for each investigated system, and a sample preparation procedure for STED‐microscopy was established preventing artifacts from drying the samples. For the investigation of their dynamic behavior, we mixed preassembled fibers solutions containing different dyes, monitored if an exchange occurred between them, and compared the results to a positive control with premixed fibers. To our surprise, we were not able to observe a dynamic exchange between the fibers under ambient conditions monitoring the samples over several days. This led us to the conclusion that the lateral aggregation induced by a combination of the amphiphilic nature of the building blocks and the presence of strong hydrogen bonds results in a trapped or frozen supramolecular assembly. An automated image analysis based on JIPipe confirmed the results observed in the microscopy images. Unexpectedly, reducing the hydrophobic shielding did not alter these results, which underlines the importance of the strong hydrogen bonding sites on the assembly, while the hydrophobic shielding is more crucial to maintain fiber stability. In a further attempt to induce dynamic behavior, we added an organic solvent to the aqueous solutions. First, we screened for the solubility threshold of chosen systems in a H_2_O:DMF mixture using ^1^H‐NMR in order to find the onset of fiber degradation. From there we analyzed the chosen systems just below this threshold to see if a dynamic exchange can occur under the most extreme conditions. For the BTP‐C6 system, we were not able observe any dynamic mixing of the fibers within 1 h but only effects of fiber destabilization. The BTP‐C12 system on the other hand, showed a higher tolerance against organic solvents and we were able to observe an exchange/rearrangement between the labeled fibers. It is noteworthy that in this case, the observed dynamic exchange is rather slow compared to the aggregation process happening in seconds, which creates interesting opportunities for a living growth of defined fibers, provided that the nucleation and growth process can be sufficiently controlled.^[^ ^12^, ^63^ ^]^ While the dynamic exchange in the presence of good solvents could be expected, we observed a more unexpected behavior when heating the sample to still rather moderate temperatures of 60 °C. This temperature increase induced a dynamic exchange similar to the previous case, although here no further competitive solvent was present. Considering the absence of any dynamics at ambient conditions, the observed exchange within minutes at this temperature renders heating a very specific trigger for inducing dynamics to the presented systems. We assume that the enhanced temperature simultaneously weakens both driving forces, the hydrophobic effect as well as the hydrogen bonds between the units, which is in accordance with results obtained for amphiphilic block copolymers and related studies on hydrogen bonds.^[^ ^46^, ^64^, ^65^ ^]^ The supramolecular fibers presented here therefore represent a unique system, which allows selective switching between a non‐dynamic or kinetically trapped state and dynamic exchange by either adding a competitive solvent or, more importantly, by a rather slight increase of the solution temperature. Such a selective triggering of dynamics might, for example, facilitate the preparation of locked‐in patterns or, in combination with spatial controlled heating, local reorganizations and disintegrations of related hydrogels and therefore intrigue unprecedented creations in supramolecular materials.^[^ ^66^ ^]^

## 4 Experimental Section

### Materials

All reagents and solvents were commercial products purchased from Sigma‐Aldrich, abcr, Iris BioTech, Rapp Polymere, TCI, Lumiprobe or Fluoroprobes and were used without further purification.

### Synthesis

The synthesis of the core BTU and BTP building block have been previously reported by our group.^[^ ^36^, ^54^ ^]^ The synthesis protocols for Alexa Flour dye attachment can be found in the Chapter S1. (Supporting Information)

### Size‐Exclusion Chromatography (SEC)

Size‐exclusion chromatography (SEC) of polymers was performed on an Agilent system (series 1200) equipped with a PSS degasser, a G1310A pump, a G1362A refractive index detector, and a PSS GRAM 30 and 1000 column with DMAc (+ 0.21 wt.% LiCl) as eluent at a flow rate of 1 mL min^−1^. The column oven was set to 40 °C, and poly(ethylene glycol) (PEO) standards were used for calibration.

### Assembly via Solvent Switch

The dye conjugates were assembled from DMF (stock solution: *c* = 5 mg mL^−1^) to water according to a previously reported procedure^[^ ^35^ ^]^ to obtain a stock solution with a total concentration of 1 mg mL^−1^ and a ratio of 99:1 pure building block: dye‐containing building block. These solutions were diluted to 0.1 mg mL^−1^ for STED imaging.

### STED Imaging

Two‐color STED images were acquired with Abberior Expert Line microscope (Abberior Instruments) using an Olympus UPlanSApo 100x/1.4 oil immersion objective. AF488 was excited by a 488 nm pulsed diode laser, and the fluorescence signal was inhibited by a pulsed 595 nm depletion laser, and AF594 was excited by a 561 nm pulsed diode laser and signal inhibited by a pulsed 775 nm depletion laser. In order to have STED perform at full potential, alignment of the excitation and depletion beams was needed as described elsewhere.^[^ ^67^ ^]^ Briefly, the center of the excitation and depletion beams were overlapped first by scanning gold beads of 150 nm (BBI Solutions) in a reflection mode. Afterward, TetraSpeck beads of four colors (TetraSpeck Microspheres, 100 nm, fluorescent blue/green/orange/dark red) were used to correct mismatches between the scattering mode and the fluorescence mode. Then the individual confocal and STED channels were compared, respectively, to ensure the correct positioning of the beads imaged by different laser conditions. During fiber imaging, sequential scanning was applied to keep photobleaching of the samples to a minimum state, with first the confocal and STED channels for AF594 labeled fibers, then AF488 labeled fibers. Both confocal and STED images were compared for the same imaging area, with STED images yielding more precise geometric information of the polymer fibers thanks to the improved lateral resolution.

### Sample Preparation for STED Imaging

The stock solutions of differently labeled fibers were mixed previously for different time periods in either water or H_2_O/DMF, and afterward investigated via STED. To guarantee immobilized fibers on the glass surface of an 8‐well glass slide (Ibidi µ‐Slide 8 Well high Glass Bottom), 10 µL of a 0.05 m HCl solution were first added to the glass grid. Then 45 µL of water were added, followed by 5 µL of the 0.1 mg mL^−1^ dye‐conjugate stock solution. The solution was then untouched for 5 min. Afterward, a washing procedure was performed, to reduce the fiber density on the glass substrate. To this end, 200 µL of water were added, and again 200 µL of the solution was removed. Then, 200 µL of water were added to guarantee a wet glass surface and to avoid a PEO crystallization upon drying of the solution. As the polymer fibers adhere onto the coverslip surface, 2D STED was employed for a higher lateral resolution. Original image size was 10 ×10 µm2 with pixel size of 20 nm, and pixel dwell time of 10 µs, each line was scanned two times. The images were later cropped for the best visual presentation, signifying the location of the polymer fibers.

### Sample Preparation for Temperature‐Treated Samples

50 µL the AF488 and AF594 containing fiber stock solutions were combined and diluted with 900 µL milliQ Water. The samples were stirred at 60 °C using a thermoshaker. After the heat treatment, the samples were cooled down to RT and measured using the standard STED procedure.

### Automated Image Analysis

Image quantification was carried out using the ImageJ‐based graphical image analysis language JIPipe.^[^ ^68^, ^69^ ^]^ The microscopy images were provided in their native “.msr” format, which contained six series: series 1–4 contained the full‐resolution confocal and STED images, recorded at 594 nm and 488 nm laser excitation wavelengths. The assignment of modalities (confocal vs STED) and wavelengths (594 nm vs 488 nm) was accomplished by JIPipe annotation tools (see the symbolic pipeline in Figure S13, Supporting Information) based on either the series number or the ImageJ window name of the various image series.

The location of the molecules was first approximated by creating a mask based on the sum of the two labels (594 and 488 nm channels) for both the confocal and the STED modalities. The confocal channel‐based mask was more continuous and thus served as the primary tool in estimating the location of the molecules and molecular segments of both colors (see Figure S13 for details; the entire JIPipe code and a detailed description of all nodes and settings are provided in the Supporting Information). The analysis tool was divided into five compartments in order to increase code readability, where the current study relies on compartments C1 to C3 (Figure S13, Supporting Information). The pipelines depicting the inner structure of these three compartments are demonstrated in Figure S14 (Supporting Information). Here C1 was responsible for reading in the native image files and identifying which series corresponded to which modality and labelling color. In C2, the segmentation of the merged images allowed the measurement of the fluorescence intensities for each individual molecule, as well as for the entire image. By identifying the molecules and molecular segments in C2, it was also possible to calculate the colocalization measures of the two labels, as performed in compartment C3 (Figure S14, Supporting Information).

The concrete steps of identifying the molecular masks are detailed in Figure S15 (Supporting Information). The processing relied heavily on the Morphological Feature Contrast (MFC) tool, which elevated the contrast for the filamentous structures in the merged images.^[^ ^70^ ^]^ The actual parameters that were used for optimal segmentation using the MFC tool can be found in the detailed JIPipe summary file, as well as in the provided JIPipe code (see Supporting Information). The optimal parameters were arrived at by testing a range of values and choosing the best combination based on visual comparisons between the masks and the raw images. The successfully identified molecules were skeletonized and converted into filaments, to be able to easily calculate geometrical characteristics (full and segmental lengths, orientation angles, branching degrees, etc.), as well as colocalization measures (Figure S15, Supporting Information).

The identified filaments were analyzed further as shown in Figure S16 (Supporting Information). The individual molecules were created by splitting the per‐image filament network into connected components, followed by a filtering node to eliminate potential artefacts, assigning a coordinate system to each filament, and normalizing the distance scale to the total length of the filament. The lattermost step allowed the per‐length measures, e.g. the fluorescence intensities of the two colors, to be plotted comparably on a zero‐to‐one scale along the length of the fibers. The main extracted measures included the intensity of the green (488 nm excitation) and magenta (594 nm excitation) fluorescence labels along the length of the molecules, the plotting of the intensity distributions for the two colors at each filament, as well as the colocalization measures interpreted on a per‐pixel basis using the Pearson's R coefficient (Figure S16, Supporting Information).

The main steps of the masking and filament‐generation process can be viewed via an example from the positive control group, as shown in Figure S18 (Supporting Information). The addition of the 488 and 594 nm images resulted in a continuous intensity pattern for both the confocal and the STED images (Figure S18a,b, Supporting Information resp.). The higher intensity levels made the confocal merged image a better choice for creating a continuous mask. When the mask was applied to the individual 488 and 594 nm images (Figure S18c,d, Supporting Information resp.) after merging (Figure S18e, Supporting Information), a set of first‐approximation filaments were created (Figure S18f, Supporting Information) according to the process detailed above. These filaments were corrected in order to reconnect those that were artificially disconnected, based on the conditions that i) the to‐be‐merged fibers were not longer than 40 pixels, ii) the vertices of the fiber‐ends were of 1^st^ or 2^nd^ degree (i.e., one or two edges were meeting at these vertices), and iii) they were pointing opposite at each other at near‐parallel directions (the dot product of the two vectors describing the two segments was set to be limited by −0.8, specifying an approximately 50 degree tolerance around 180 degrees, i.e. 130 to 230 degrees, in the direction of the facing filament segments). The green lines in Figure S18g (Supporting Information) show these corrective filament connections, whereas Figure S18h (Supporting Information) indicates the overlap between the abstract filaments and the measured image.

The correlation between the green (488 nm) and magenta (594 nm) fluorescence intensities was measured alongside the corrected filaments in each condition (positive control, 10‐day mix, 50% DMF, 24 h tempered; example results of the four conditions for BTP‐C12 are shown in Figure S20a; Figures S23d and S26d, and S27d, Supporting Information resp.). The observed positive versus negative correlation between the two labels corresponds to the segment‐mixing conditions of the positive control versus the other three conditions.

The Pearson's R coefficient was calculated for the pixels inside the corrected filaments mask, where the radii of the filaments were adjusted according to those of the original molecules, as calculated from the Euclidean distance transform of the masked raw images (see Figure S16 for the corresponding JIPipe nodes, Supporting Information). The comparison of the R‐value distributions was then utilized as another tool to characterize the positive control condition (high R‐value, Figure S20b, Supporting Information) compared to those of the other three mixing conditions (R values near zero, or slightly negative for the 10‐day mix condition, Figures S23e and S26e, and S27e, Supporting Information resp.).

The intensity distributions were calculated for the two labels alongside the fiber length, as well as per individual fiber identification number. The former curve indicates the relative occurrence of the two molecular components alongside a molecule, calculated from the raw images masked with the radii‐corrected filaments. The comparison of the example data for BTP‐C12 in positive control (Figure S20c, Supporting Information) versus the three mixing variants (Figures S23f and S26f, and S27f, Supporting Information) indicates the applicability of this tool as well.

The mixing of the two components, regardless of the position alongside of the fiber, calculated for each fiber in an image, allows the direct comparison of the labelling intensities under various conditions (Figure S20d, compared with Figures S23g and S26g, and S27g, Supporting Information).

An example of the fiber distance‐ and fiber number‐dependence for an individual molecule is also shown for the positive control in Figure S20e,f (Supporting Information).

### Nuclear Magnetic Resonance (NMR) Spectroscopy

^1^H‐NMR spectra were measured with a Bruker spectrometer (300 MHz) equipped with an Avance I console, a dual ^1^H and ^13^C sample head, and a 120x BACS automatic sample changer. The chemical shifts of the peaks were determined by using the residual solvent signal as reference and are given in ppm in comparison to TMS.

### ^1^H‐NMR Investigation of the Assembly's Solvent Tolerance

For the BTP‐C6 system a stock solution containing the unfunctionalized building block with a concentration 20 mg mL^−1^ was prepared via direct dispersion of the sample in D_2_O. For the BTP‐C12 system, a solvent switch from THF to D_2_O was performed, and the THF was removed via evaporation, yielding in a concentration of 20 mg mL^−1^. To each stock solution, 3 mg of Trioxane were added as an internal standard. For the sample preparation 300 µL of stock solution was used, and D_2_O and d_7_‐DMF were added successively to obtain the targeted volume mixture.

## Conflict of Interest

The authors declare no conflict of interest.

## Supporting information



Supporting Information

## Data Availability

The data that support the findings of this study are available from the corresponding author upon reasonable request.
