# Supplementary material for: Selective Activation of Dynamics in Kinetically Frozen Supramolecular Polymer Bottlebrush Assemblies
Source: Small. 2025 Sep 15;21(44):e05481. doi: 10.1002/smll.202505481 (PMC12590525; doi:10.1002/smll.202505481)
Supplement: Supplementary file 1 — Supporting Information [file SMLL-21-e05481-s001.docx]

**Selective activation of dynamics in kinetically frozen supramolecular polymer bottlebrush assemblies**

Hans F. Ulrich^a, b^, Tobias Klein^a,b^, Ziliang Zhao^c, d^, Zoltán Cseresnyés^e^, Pablo Carravilla^c, d^, Ruman Gerst^e^, Alina Kasberg^g^, Frederic P. Scharfenberg^a^, Marc Thilo Figge^e f^, Christian Eggeling^b, c, d^, Johannes C. Brendel^a, b, g, *^

a Laboratory of Organic and Macromolecular Chemistry (IOMC), Friedrich-Schiller-University Jena, Humboldtstraße 10, 07743 Jena, Germany

b Jena Center for Soft Matter (JCSM), Friedrich-Schiller-University Jena, Philosophenweg 7, 07743 Jena, Germany

c Institute for Applied Optics and Biophysics, Friedrich Schiller University Jena, 07743 Jena, Germany.

d Leibniz Institute of Photonic Technology e.V., member of the Leibniz Centre for Photonics in Infection Research (LPI), 07743 Jena, Germany.

e Applied Systems Biology, Leibniz Institute for Natural Product Research and Infection Biology, Hans Knöll Institute (HKI), 07743 Jena, Germany

f Institute of Microbiology, Faculty of Biological Sciences, Friedrich-Schiller-University Jena, 07743 Jena, Germany.

g Macromolecular Chemistry I, University of Bayreuth, Universitätsstr. 30, 95447 Bayreuth

*Corresponding author: johannes.brendel@uni-bayreuth.de

Table of Contents

[1. Methods and Materials 3](#_Toc204802361)

[1.1. Materials 3](#_Toc204802362)

[1.2. Synthesis 3](#_Toc204802363)

[1.3. Size-exclusion chromatography (SEC) 3](#_Toc204802364)

[1.4. Assembly via solvent switch 3](#_Toc204802365)

[1.5. STED imaging 3](#_Toc204802366)

[1.6. Sample preparation for STED imaging. 4](#_Toc204802367)

[1.7. Sample preparation for temperature treated samples. 4](#_Toc204802368)

[1.8. Automated image analysis 5](#_Toc204802369)

[1.9. Nuclear magnetic resonance (NMR) spectroscopy 7](#_Toc204802370)

[1.10. ^1^H-NMR investigation of the assembly’s solvent tolerance 7](#_Toc204802371)

[2. Synthesis 8](#_Toc204802372)

[2.1 Synthetic routes 8](#_Toc204802373)

[2.2 Synthetic protocols 11](#_Toc204802374)

[3. Characterization 18](#_Toc204802375)

[3.1 FRET 18](#_Toc204802376)

[3.2 STED 18](#_Toc204802377)

[3.3 Automated image analysis using a JIPipe workflow 27](#_Toc204802378)

[4. References 42](#_Toc204802379)

[5. Utilized nodes for the automated image analysis 43](#_Toc204802380)

[6. Dependencies 52](#_Toc204802381)

[6.1 JIPipe extensions 52](#_Toc204802382)

[6.2 ImageJ update sites 53](#_Toc204802383)

[6.3 External environments 53](#_Toc204802384)

[6.4 Citations (dependencies) 53](#_Toc204802385)

[7. Pipeline text description 55](#_Toc204802386)

[7.1 Compartment C1 "Preprocessing" 55](#_Toc204802387)

[7.2 Compartment C2 "Segmentation and colocalization" 60](#_Toc204802388)

[7.3 Compartment C3 "Mask" 77](#_Toc204802389)

[7.4 Compartment C4 "Filaments and colocalization in STED" 103](#_Toc204802390)

[7.5 Compartment C5 "Colocalization" 117](#_Toc204802391)

[7.6 Compartment C6 "Saving" 131](#_Toc204802392)

# Methods and Materials

## Materials

All reagents and solvents were commercial products purchased from Sigma-Aldrich, abcr, Iris BioTech, Rapp Polymere, TCI, Lumiprobe or Fluoroprobes and were used without further purification.

## Synthesis

The synthesis of the core BTU and BTP building block have been previously reported by our group.^[1-2]^ The synthesis protocols for Alexa Flour dye attachment can be found in the Supplementary Information Chapter 1.

## Size-exclusion chromatography (SEC)

Size-exclusion chromatography (SEC) of polymers was performed on an Agilent system (series 1200) equipped with a PSS degasser, a G1310A pump, a G1362A refractive index detector and a PSS GRAM 30 and 1000 column with DMAc (+ 0.21 wt.% LiCl) as eluent at a flow rate of 1 mL min^-1^. The column oven was set to 40 °C and poly(ethylene glycol) (PEO) standards were used for calibration.

## Assembly via solvent switch

The dye conjugates were assembled from DMF (stock solution: *c* = 5 mg mL^-1^) to water according to a previously reported procedure^[3]^ to obtain stock solution with a total concentration of 1 mg mL^-1^ and a ratio of 99:1 pure building block:dye containing building block. These solutions were diluted to 0.1 mg mL^-1^ for STED imaging.

## STED imaging

Two-color STED images were acquired with Abberior Expert Line using an Olympus UPlanSApo 100x/1.4 oil immersion objective. AF488 was excited by a 488 nm pulsed diode laser and the fluorescence signal was inhibited by a pulsed 595 nm depletion laser, and AF594 was excited by a 561 nm pulsed diode laser and signal inhibited by a pulsed 775 nm depletion laser. In order to have STED perform at full potential, alignment of the excitation and depletion beams was needed as described elsewhere.^[4]^ Briefly, the center of the excitation and depletion beams were overlapped first by scanning gold beads of 150 nm (BBI Solutions) in a reflection mode. Afterwards, TetraSpeck beads of four colors (TetraSpeck^TM^ Microspheres, 100 nm, fluorescent blue/green/orange/dark red) were used to correct mismatches between the scattering mode and the fluorescence mode. Then the individual confocal and STED channels were compared respectively, to ensure correct positioning of the beads imaged by different laser conditions. During fiber imaging, sequential scanning was applied to keep photobleaching of the samples to a minimum state, with first the confocal and STED channels for AF594 labeled fibers, then AF488 labeled fibers. Both confocal and STED images were compared for the same imaging area, with STED images yielding more precise geometric information of the polymer fibers thanks to the improved lateral resolution.

## Sample preparation for STED imaging.

The stock solutions of differently labeled fibers were mixed previously for different time periods in either water or H_2_O/DMF and afterwards investigated via STED. To guarantee immobilized fibers on the glass surface of an 8-well glass slide (Ibidi µ-Slide 8 Well high Glass Bottom), 10 µL of a 0.05 M HCl solution were first added to the glass grid. Then 45 µL of water were added followed by 5 µL of the 0.1 mg mL^-1^ dye-conjugate stock solution. The solution was then untouched for 5 min. Afterwards, a washing procedure was performed, to reduce the fiber density on the glass substrate. To this end, 200 µL of water were added and again 200 µL of the solution removed. Then, 200 µL of water were added to guarantee a wet glass surface and to avoid a PEO crystallization upon drying of the solution. As the polymer fibers adhere onto the coverslip surface, 2D STED was employed for a higher lateral resolution. Original image size was 10 ×10 µm^2^ with pixel size of 20 nm, and pixel dwell time of 10 µs, each line was scanned two times. The images were later cropped for best visual presentation signifying the location of the polymer fibers.

## Sample preparation for temperature treated samples.

50 µL the AF488 and AF594 containing fiber stock solutions were combined and diluted with 900 µL milliQ Water. The samples were stirred at 60 °C using a thermoshaker. After the heat treatment the samples were cooled down to RT and measured using the standard STED procedure.

## Automated image analysis

Image quantification was carried out using the ImageJ-based graphical image analysis language JIPipe.^[5-6]^ The microscopy images were provided in their native “.msr” format, which contained six series: series 1-4 contained the full-resolution confocal and STED images, recorded at 594 nm and 488 nm laser excitation wavelengths. The assignment of modalities (confocal vs. STED) and wavelengths (594 nm vs. 488 nm) was accomplished by JIPipe annotation tools (see the symbolic pipeline in Supplementary Figure 12) based on either the series number or the ImageJ window name of the various image series.

The location of the molecules was first approximated by creating a mask based on the sum of the two labels (594 nm and 488 nm channels) for both the confocal and the STED modalities. The confocal channel-based mask was more continuous and thus served as the primary tool in estimating the location of the molecules and molecular segments of both colors (see Supplementary Figure 12 for details; the entire JIPipe code and a detailed description of all nodes and settings are provided in the Supplementary Materials). The analysis tool was divided into five compartments in order to increase code readability, where the current study relies on compartments C1 to C3 (Supplementary Figure 13). The pipelines depicting the inner structure of these three compartments are demonstrated in Supplementary Figure 14. Here C1 was responsible for reading in the native image files and identifying which series corresponded to which modality and labelling color. In C2, the segmentation of the merged images allowed the measurement of the fluorescence intensities for each individual molecule, as well as for the entire image. By identifying the molecules and molecular segments in C2, it was also possible to calculate the colocalization measures of the two labels, as performed in compartment C3 (Supplementary Figure 14).

The concrete steps of identifying the molecular masks are detailed in Supplementary Figure 15. The processing relied heavily on the Morphological Feature Contrast (MFC) tool, which elevated the contrast for the filamentous structures in the merged images.^[7]^ The actual parameters that were used for optimal segmentation using the MFC tool can be found in the detailed JIPipe summary file, as well as in the provided JIPipe code (see Supplementary Materials). The optimal parameters were arrived at by testing a range of values and choosing the best combination chosen based on visual comparisons between the masks and the raw images. The successfully identified molecules were skeletonized and converted into filaments, to be able to easily calculate geometrical characteristics (full and segmental lengths, orientation angles, etc.), as well as colocalization measures (Supplementary Figure 15).

The identified filaments were analyzed further as shown in Supplementary Figure 16. The individual molecules were created by splitting the per-image filament network into connected components, followed by a filtering node to eliminate potential artefacts, assigning a coordinate system to each filament, and normalizing the distance scale to the total length of the filament. The lattermost step allowed the per-length measures, e.g. the fluorescence intensities of the two colors, to be plotted comparably on a zero to one scale along the length of the fibers. The main extracted measures included the intensity of the green (488 nm excitation) and magenta (594 nm excitation) fluorescence labels along the length of the molecules, the plotting of the intensity distributions for the two colors at each filament, as well as the colocalization measures interpreted on a per-pixel basis using the Pearson’s R coefficient (Supplementary Figure 16).

The main steps of the masking and filament-generation process can be viewed via an example from the positive control group, as shown in Supplementary Figure 17. The addition of the 488 nm and 594 nm images resulted in a continuous intensity pattern for both the confocal and the STED images (Supplementary Figure 17a and b, resp.). The higher intensity levels made the confocal merged image a better choice for creating a continuous mask. When the mask was applied to the individual 488 nm and 594 nm images (Supplementary Figure 17c and d, resp.) after merging (Supplementary Figure 17e), a set of first-approximation filaments were created (Supplementary Figure 17f) according to the process detailed above. These filaments were corrected in order to reconnect those that were artificially disconnected, based on the conditions that i) the to-be-merged fibers were not longer than 40 pixels, ii) the vertices of the fiber-ends were of 1^st^ or 2^nd^ order (i.e., maximum two edges were meeting at these vertices), and iii) they were pointing opposite at each other at near-parallel directions (the dot product of the two vectors describing the two segments was set to be limited by ‑0.8, specifying an approximately 50 degree tolerance around 180 degrees in the directions of the facing filament segments). The green lines in Supplementary Figure 17g show these corrective filament connections, whereas Supplementary Figure 17h indicates the overlap between the abstract filaments and the measured image.

The correlation between the green (488 nm) and magenta (594 nm) fluorescence intensities was measured alongside the corrected filaments in each condition (positive control, 10-day mix, 50% DMF, 24 hours tempered; example results for BTP-C12 are shown in Supplementary Figure 18a, and Supplementary Figure 21d, 24d & 25d, resp.). The observed positive vs. negative correlation between the two labels corresponds to the segment-mixing conditions of the positive control vs. the other three conditions.

The Pearson’s R coefficient was calculated for the pixels inside the corrected filaments mask, where the radii of the filaments were adjusted according to the radii of the original molecules, as calculated from the Euclidean distance transform of the masked raw images (see Supplementary Figure 15 for the corresponding JIPipe nodes). The comparison of the R-value distributions was then utilized as another tool to characterize the positive control condition (high R-value, Supplementary Figure 18b) compared to those of the other three mixing conditions (R values near zero, or slightly negative for the 10-day mix condition, Supplementary Figure 21e, 24e & 25e, resp.).

The intensity distributions were calculated for the two labels alongside the fiber length, as well as per individual fiber number. The former curve indicates the relative occurrence of the two molecular components alongside a molecule, calculated from the raw images masked with the radii-corrected filaments. The comparison of the example data for BTP-C12 in positive control (Supplementary Figure 18c) versus the three mixing variants (Supplementary Figure 21f, 24f & 25f) indicates the applicability of this tool as well.

The mixing of the two components regardless of the position alongside of the fiber, calculated for each fiber in an image, allows the direct comparison of the labelling intensities under various conditions (Supplementary Figure 18d, compared with Supplementary Figure 21g, 24g & 25g).

An example of the fiber distance- and fiber number dependence for an individual molecule is also shown for the positive control in Supplementary Figure 18e-f.

## Nuclear magnetic resonance (NMR) spectroscopy

^1^H-NMR spectra were measured with a Bruker spectrometer (300 MHz) equipped with an Avance I console, a dual ^1^H and ^13^C sample head and a 120x BACS automatic sample changer. The chemical shifts of the peaks were determined by using the residual solvent signal as reference and are given in ppm in comparison to TMS.

## ^1^H-NMR investigation of the assembly’s solvent tolerance

For the BTP-C6 system a stock solution containing the unfunctionalized building block with a concentration 20 mg mL^-1^ was prepared *via* direct dispersion of the sample in D_2_O. For the BTP‑C12 system a solvent switch from THF to D_2_O was performed and the THF was removed via evaporation yielding in a concentration of 20 mg mL^-1^. To each stock solution 3 mg of Trioxane were added as an internal standard. For the sample preparation 300 μL of stock solution were used and D_2_O and d_7_-DMF were added successively to obtain the targeted volume mixture.

# Synthesis

## Synthetic routes

The synthetic routes to the core moieties were performed according to previously published procedures without any changes.^[1]^


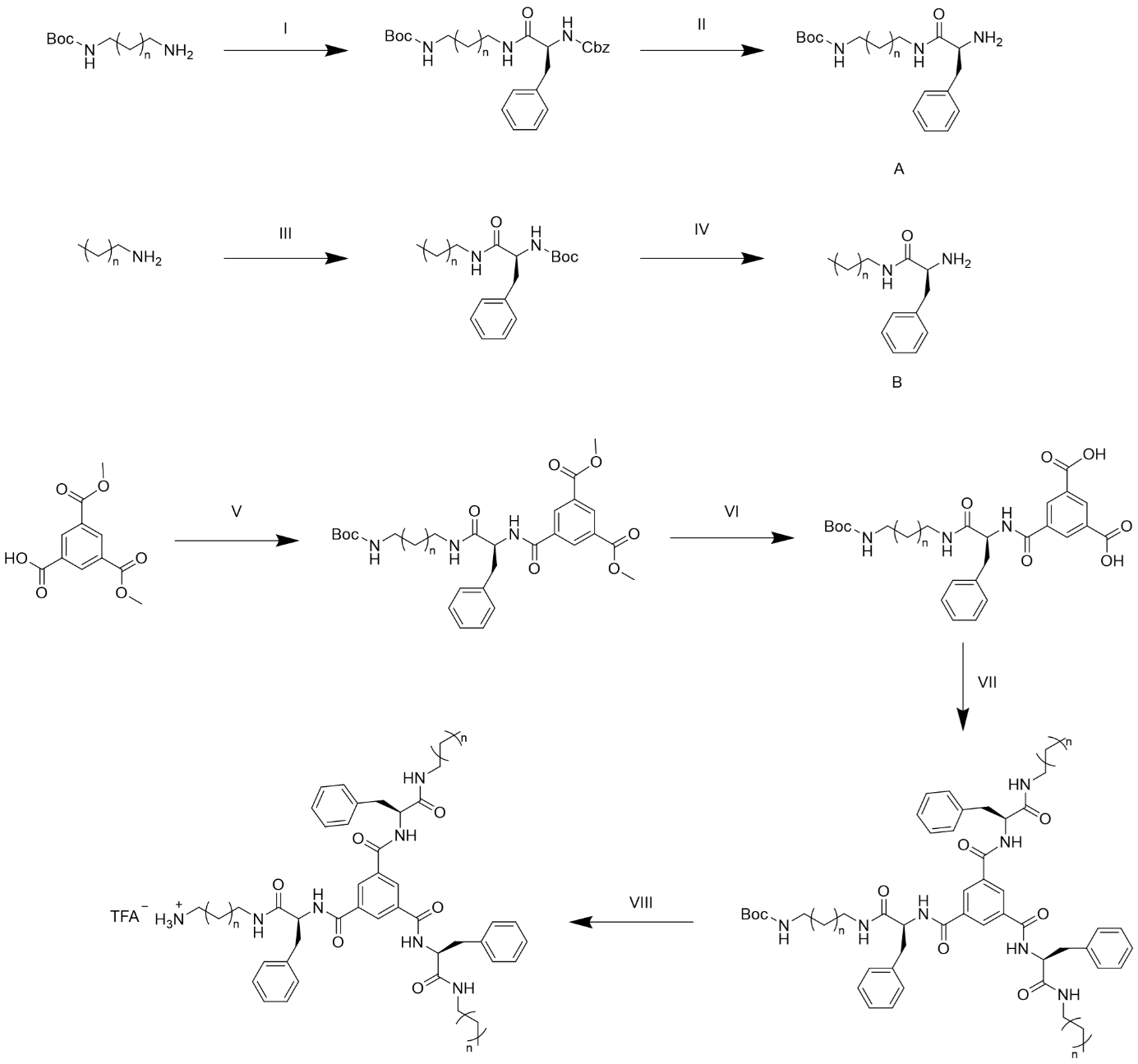


Scheme S1: Synthesis route of the core BTP moiety.^[1]^ (I, III, V and VII) using respectively Phe-Boc/Phe-Cbz/A/B, EDC·HCl, DMAP, CHCl_3_ or DMF, rt, over night; (II) 10% Pd on C, H_2_ , dioxane, rt, over night; (IV andVIII) TFA/TIPS/H_2_O, DCM, rt, 2h; (VI) KOH in EtOH:H_2_O 9:1, reflux, 30 min.^[1]^


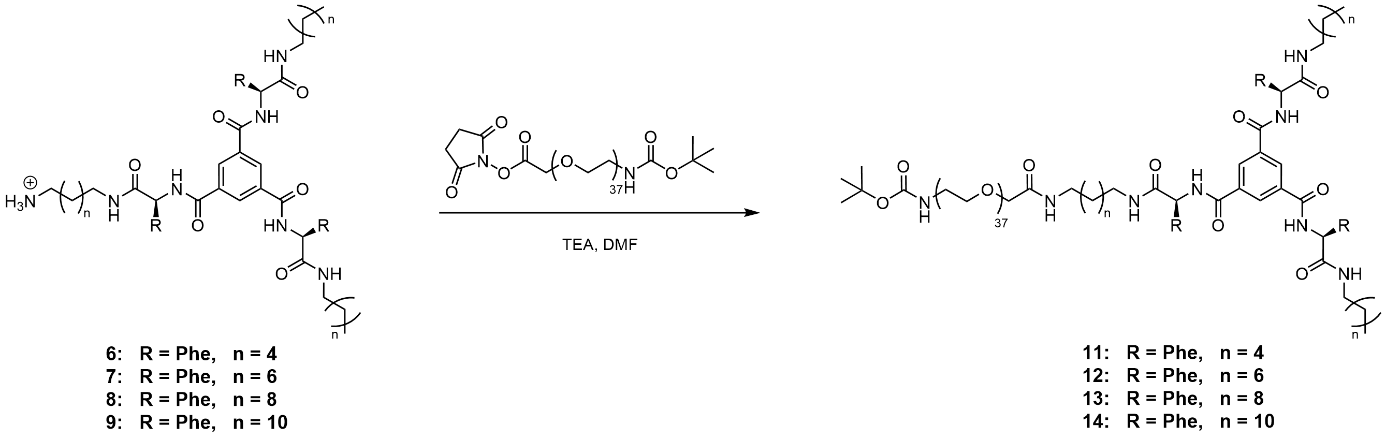


Scheme S2: Synthetic route to the benzenetrispeptide conjugates 11-14 bearing a Boc-protected PEO chain.


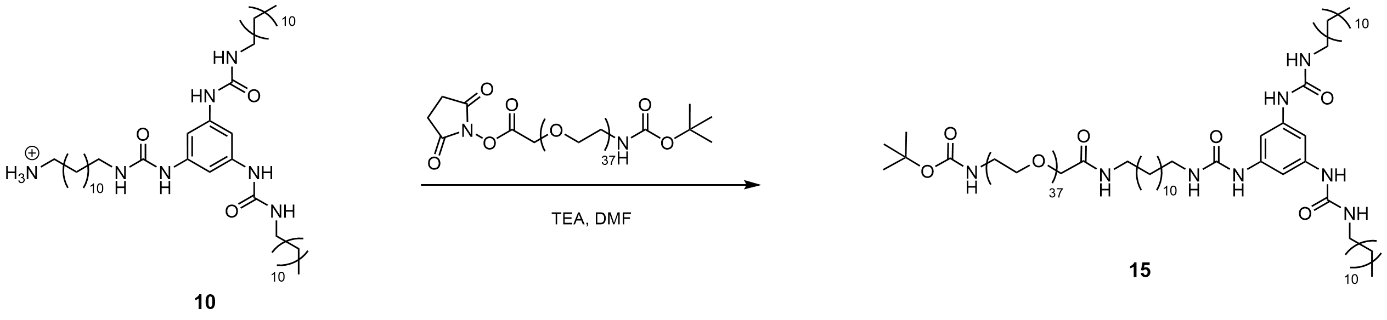


Scheme S3: Synthetic route to the benzenetrisurea conjugate 15 bearing a Boc-protected PEO chain.


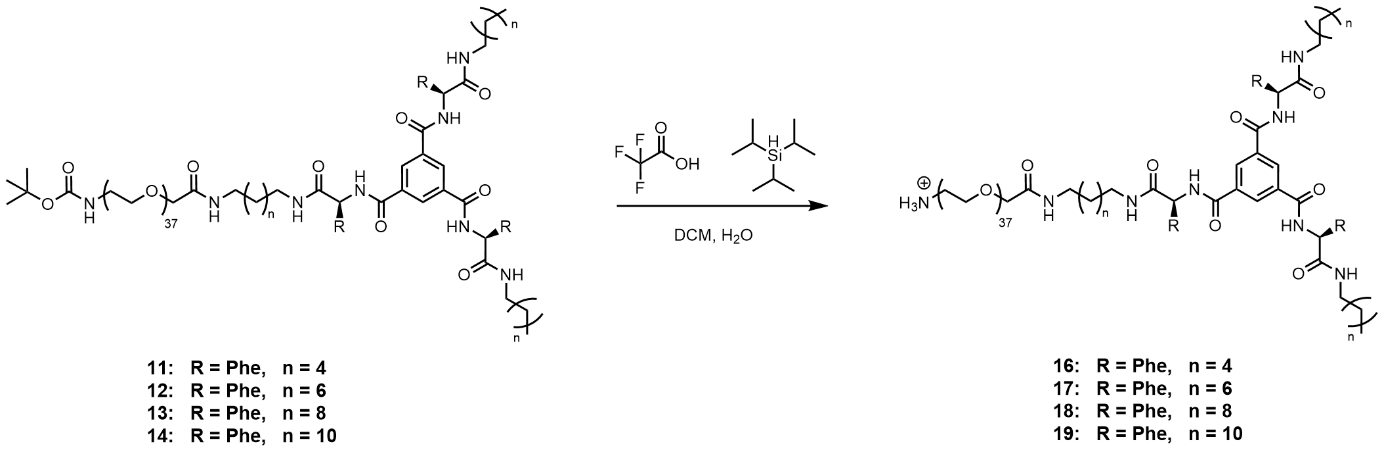


Scheme S4: Boc-deprotection of 11-14 resulting in 16-19.


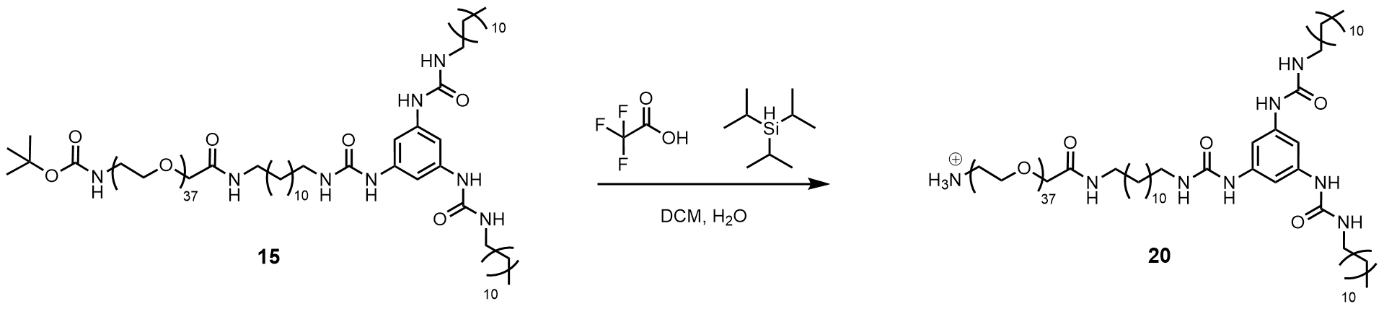


Scheme S5: Boc-deprotection of 17 resulting in 23.


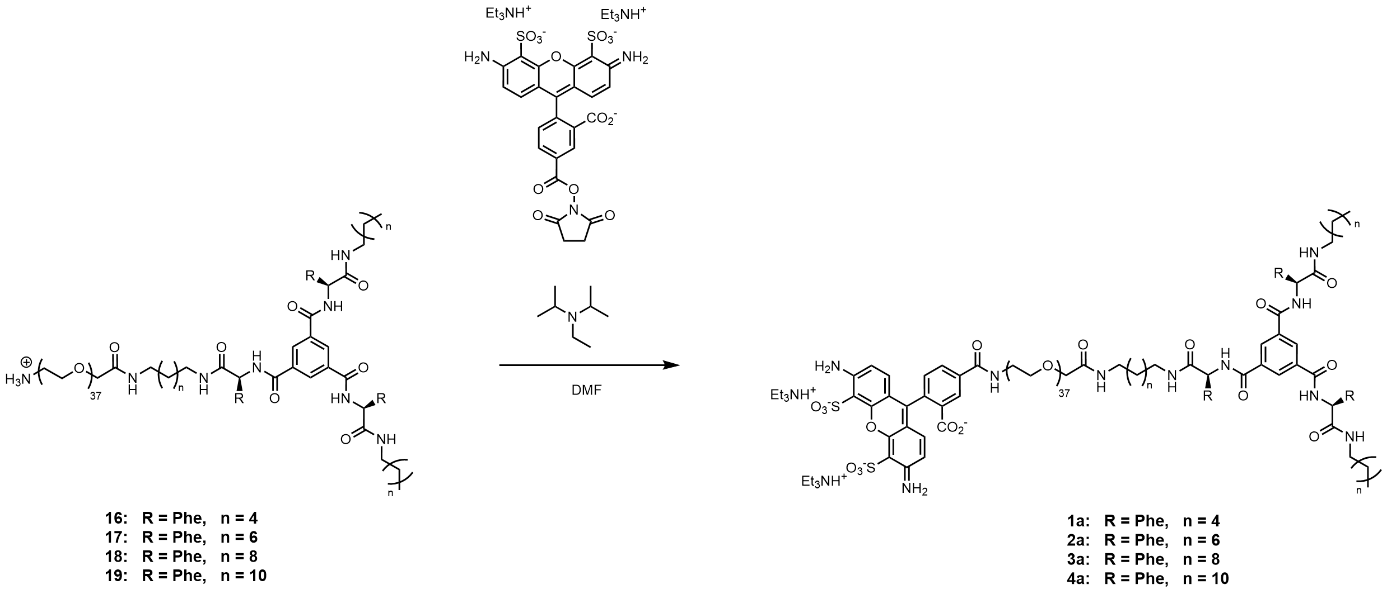


Scheme S6: Attachment of NHS-AF488 to 16-19 resulting in 1-4a.


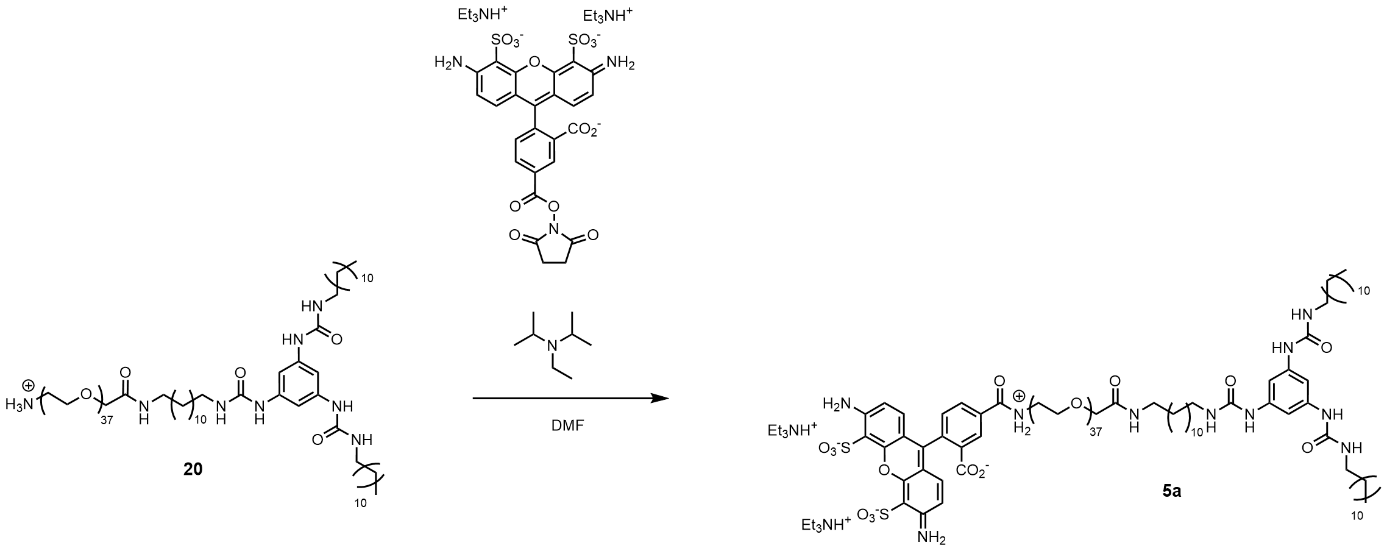


Scheme S7: Attachment of NHS-AF488 to 20 resulting in 5a.


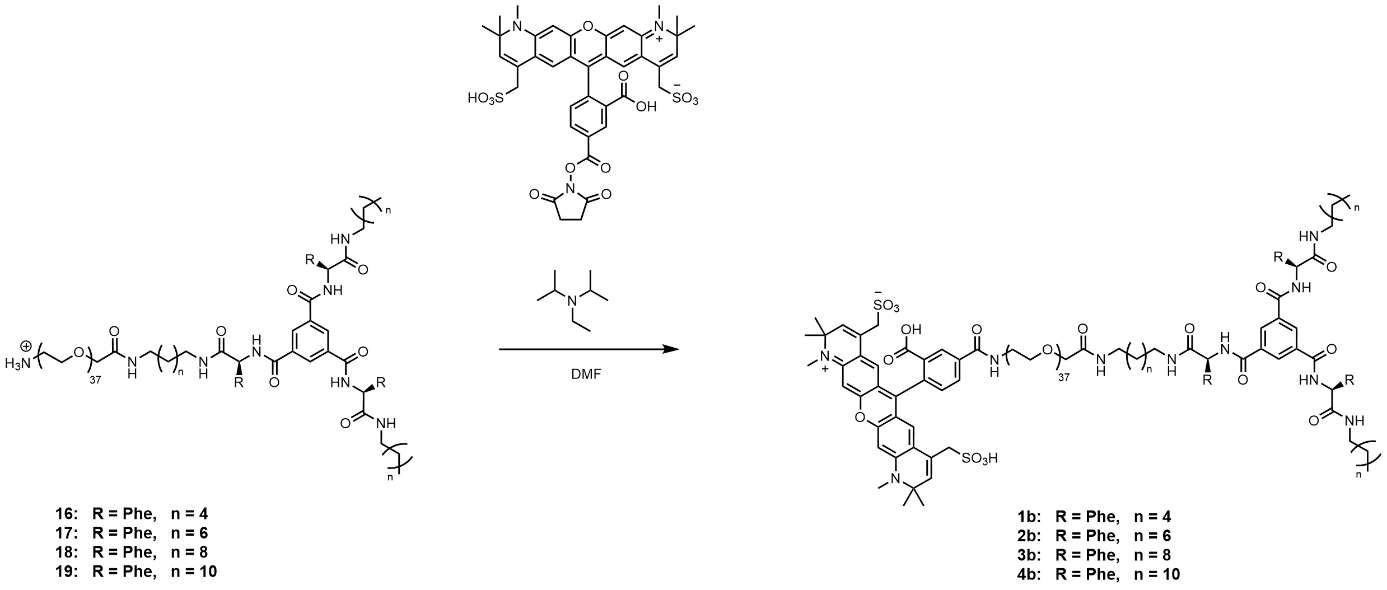


Scheme S8: Attachment of NHS-AF594 to 16-19 resulting in 1-4b.


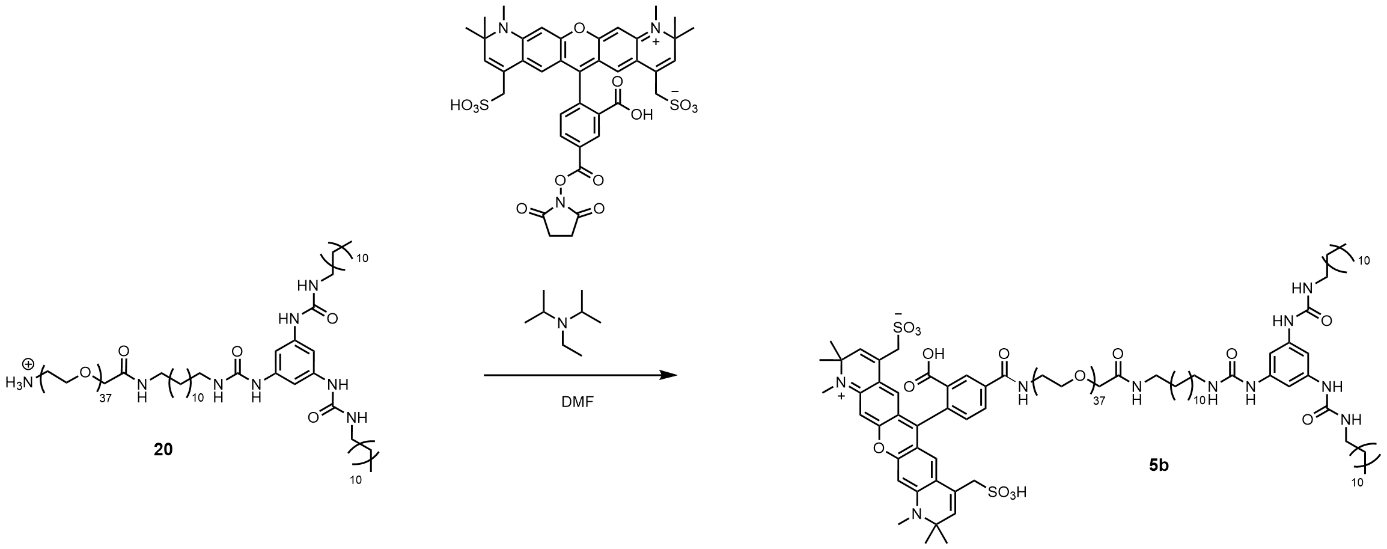


Scheme S9: Attachment of NHS-AF594 to 20 resulting in 5b.

## Synthetic protocols

**Conjugation of NHS-PEO_2k_-NHBoc**

[B][Phe]_3_[C_6_]_3_[PEO_2k_-NHBoc] **11**

The starting material **7** (0.1 g, 0.097 mmol, 1.0 eq) was dissolved in DMF. To this solution, 135 µL of triethylamine (0.97 mmol, 10 eq) were added and the solution stirred for 15 min at rt. Subsequently, NHS-PEO_2k_-NHBoc (0.25 g, 1.35 eq) was added, and the mixture stirred for 24 h at rt. After checking the conversion via SEC, the reaction mixture was precipitated into cold diethyl ether and subsequently centrifuged. The supernatant was discarded and an Et2O:acetone (9:1 v:v) solution added to the precipitate to remove residual PEO traces. After another centrifugation, the supernatant was decanted, and the precipitate dried *in vacuo* (40 °C) overnight. The obtained white solid was dissolved in MilliQ water (3 mg/mL) by stirring vigorously overnight. Residual PEO traces could be removed via several centrifugal washing steps using Amicon centrifugal filter units (10 kDa MWCO). The retained solution was then freeze dried overnight to yield a white powder.

**Yield:** 262 mg, 0.08 mmol (82%), white powder.

**^1^H-NMR (300 MHz, d_6_-DMSO, 298 K):** δ [ppm] = 8.73 (t, J = 7.2 Hz, 3H, NH), 8.3 (m, 3H, CH_aromat_), 8.10 (s, 3H, NH), 7.38 – 7.10 (m, 15H, CH_aromat_), 6.81 – 6.72 (m, 1H, NH), 4.78 – 4.66 (m, 3H, CH), 4.07 – 4.00 (m, 2H, CH_2_), 3.77 – 3.71 (m, 2H, CH_2_), 3.50 (m, 148H, PEO), 3.15 – 2.88 (m, 14H, CH_2_), 1.37 (m, 14H, CH_2_), 1.22 (m, 16H, CH_2_), 0.84 (t, J = 6.4 Hz, 6H, CH_3_).

**SEC (DMAc + 0.21 wt.% LiCl):** M_n_ = 4,200 g mol^-1^_;_ M_w_ = 4,600 g mol^-1^_;_ Đ = 1.09.

[B][Phe]_3_[C_8_]_3_[PEO_2k_-NHBoc] **12**

**12** was synthesised according to the procedure of compound **11**.

**Yield:** 126 mg, 0.045 mmol (84%), white powder.

**^1^H-NMR (300 MHz, d_6_-DMSO, 298 K):** δ [ppm] = 8.71 (t, J = 7.2 Hz, 3H, NH), 8.29 (m, 3H, CH_aromat_), 8.09 (s, 3H, NH), 7.38 – 7.10 (m, 15H, CH_aromat_), 6.75 (s, 1H, NH), 4.79 – 4.64 (m, 3H, CH), 4.07 – 3.98 (m, 2H, CH_2_), 3.73 (m, 148H, PEO), 3.14 – 2.86 (m, 14H, CH_2_), 1.37 (m, 14H, CH_2_), 1.22 (m, 28H), 0.83 (t, J = 6.4 Hz, 6H, CH_3_).

**SEC (DMAc + 0.21 wt.% LiCl):** M_n_ = 3,600 g mol^-1^_;_ M_w_ = 3,900 g mol^-1^_;_ Đ = 1.07.

[B][Phe]_3_[C_10_]_3_[PEO_2k_-NHBoc] **13**

**13** was synthesised according to the procedure of compound **11**.

**Yield:** 118 mg, 0.041 mmol (76%), white powder.

**^1^H-NMR (300 MHz, d_6_-DMSO, 298 K):** δ [ppm] = 8.71 (t, J = 6.6 Hz, 3H, NH), 8.29 (m, 3H, CH_aromat_), 8.09 (s, 3H, NH), 7.37 – 7.08 (m, 15H, CH_aromat_), 6.75 (s, 1H, NH), 4.77 – 4.66 (m, 3H, CH), 4.02 (s, 2H, CH_2_), 3.73 (m, 2H, CH_2_), 3.50 (m, 148H, PEO), 3.14 – 2.87 (m, 14H, CH_2_), 1.37 (m, 14H, CH_2_), 1.21 (m, 40H), 0.83 (t, J = 6.4 Hz, 6H, CH_3_).

**SEC (DMAc + 0.21 wt.% LiCl):** M_n_ = 3,900 g mol^-1^_;_ M_w_ = 4,100 g mol^-1^_;_ Đ = 1.06.

[B][Phe]_3_[C_12_]_3_[PEO_2k_-NHBoc] **14**

**14** was synthesised according to the procedure of compound **11**.

**Yield:** 118 mg, 0.040 mmol (79%), white powder.

**^1^H-NMR (300 MHz, d_6_-DMSO, 298 K):** δ [ppm] = 8.71 (t, J = 7.3 Hz, 3H, NH), 8.35 – 8.23 (m, 3H, CH_aromat_), 8.09 (s, 3H, NH), 7.35 – 7.10 (m, 15H, CH_aromat_), 6.75 (t, J = 5.4 Hz, 1H, NH), 4.76 – 4.65 (m, 3H, CH), 4.06 – 3.99 (m, 2H, CH_2_), 3.77 – 3.69 (m, 2H, CH_2_), 3.50 (m, 148H, PEO), 3.13 – 2.87 (m, 14H, CH_2_), 1.37 (m, 14H, CH_2_), 1.21 (m, 52H), 0.88 – 0.78 (m, 6H, CH_3_).

**SEC (DMAc + 0.21 wt.% LiCl):** M_n_ = 4,000 g mol^-1^_;_ M_w_ = 4,200 g mol^-1^_;_ Đ = 1.05.

[B][U]_3_[C_12_]_3_[PEO_2k_-NHBoc] **15**

**15** was synthesised according to the procedure of compound **11**.

**Yield:** 197 mg, 0.072 mmol (76%), white powder.

**^1^H-NMR (300 MHz, d_6_-DMSO, 298 K):** δ [ppm] = 8.66 (d, J = 7.3 Hz, 3H, NH), 8.47 (s, 3H, CH_aromat_), 7.95 (t, J = 5.2 Hz, 3H, NH), 7.17 (t, J = 5.6 Hz, 1H, NH), 6.83 – 6.70 (m, 1H, NH), 4.56 – 4.41 (m, 3H, CH_2_), 4.09 – 3.97 (m, 2H, CH_2_), 3.80 – 3.70 (m, 1H), 3.50 (m, 148H, PEO), 3.05 (q, J = 5.5 Hz, 8H, CH_2_), 2.98 – 2.87 (m, 2H, CH_2_), 1.36 (m, 14H, CH_2_), 1.22 (m, 52H), 0.84 (t, J = 6.6 Hz, 6H, CH_3_).

**SEC (DMAc + 0.21 wt.% LiCl):** M_n_ = 3,000 g mol^-1^_;_ M_w_ = 3,400 g mol^-1^_;_ Đ = 1.13.

[B][Phe]_3_[C_6_]_3_[PEO_2k_-NH_3_] **16**

Compound **11** (0.22 g, 0.081 mmol, 1.0 eq) was dissolved in 1.628 mL DCM. To this, 262 µL of an 18:1:1 solution of TFA/TIPS/H_2_O (TFA: 226 µL, 2.93 mmol, 36 eq; TIPS: 33 µL, 0.163 mmol, 2 eq; H_2_O: 2.9 µL, 0.163 mmol, 2 eq) was added dropwise and the solution stirred for 2 h at rt. Afterwards, half of the DCM volume was removed and the concentrated reaction mixture precipitated in cold diethyl ether. The suspension was centrifuged (3 min, 8,000 rpm) and the supernatant decanted. The obtained product was dried in the vacuum oven (40 °C overnight).

**Yield:** 221 mg, 0.077 mmol (95%), white solid.

**^1^H-NMR (300 MHz, d_6_-DMSO, 298 K):** δ [ppm] = 8.72 (t, J = 7.0 Hz, 3H, NH), 8.37 – 8.24 (m, 3H, CH_aromat_), 8.18 – 8.04 (m, 3H, NH), 7.71 (s, 2H, NH_2_), 7.37 – 7.09 (m, 15H, CH_aromat_), 4.79 – 4.64 (m, 3H, CH), 4.08 – 3.97 (m, 2H, CH_2_), 3.78 – 3.70 (m, 2H, CH_2_), 3.50 (m, 148H, PEO), 3.17 – 2.86 (m, 14H, CH_2_), 1.44 – 1.30 (m, 8H, CH_2_), 1.30 – 1.13 (m, 16H, CH_2_), 0.84 (t, J = 6.5 Hz, 6H, CH_3_).

**SEC (DMAc + 0.21 wt.% LiCl):** M_n_ = 3,400 g mol^-1^_;_ M_w_ = 4,100 g mol^-1^_;_ Đ = 1.19.

[B][Phe]_3_[C_8_]_3_[PEO_2k_-NH_3_] **17**

**17** was synthesised according to the procedure of compound **16**.

**Yield:** 179 mg, 0.064 mmol (91%), white solid.

**^1^H-NMR (300 MHz, d_6_-DMSO, 298 K):** δ [ppm] = 8.79 – 8.68 (m, 3H, NH), 8.37 – 8.24 (m, 3H, CH_aromat_), 8.20 – 8.04 (m, 3H, NH), 7.70 (s, 2H, NH_2_), 7.39 – 7.07 (m, 15H, CH_aromat_), 4.83 – 4.62 (m, 3H, CH), 4.08 – 3.96 (m, 2H, CH_2_), 3.80 – 3.70 (m, 2H, CH_2_), 3.50 (m, 148H, PEO), 3.18 – 2.86 (m, 14H, CH_2_), 1.43 – 1.29 (m, 8H, CH_2_), 1.29 – 1.13 (m, 28H, CH_2_), 0.91 – 0.76 (m, 3H, CH_3_).

**SEC (DMAc + 0.21 wt.% LiCl):** M_n_ = 3,700 g mol^-1^_;_ M_w_ = 4,100 g mol^-1^_;_ Đ = 1.11.

[B][Phe]_3_[C_10_]_3_[PEO_2k_-NH_3_] **18**

**18** was synthesised according to the procedure of compound **16**.

**Yield:** 204 mg, 0.071 mmol (93%), white solid.

**^1^H-NMR (300 MHz, d_6_-DMSO, 298 K):** δ [ppm] = 8.78 – 8.66 (m, 3H, NH), 8.37 – 8.22 (m, 3H, CH_aromat_), 8.09 (t, J = 6.6 Hz, 3H, NH), 7.69 (s, 2H, NH_2_), 7.38 – 7.05 (m, 15H, CH_aromat_), 4.70 (dt, J = 8.9, 5.3 Hz, 3H, CH), 4.07 – 3.96 (m, 2H, CH_2_), 3.78 – 3.68 (m, 2H, CH_2_), 3.61 – 3.45 (m, 148H. PEO), 3.12 – 2.86 (m, 14H, CH_2_), 1.45 – 1.29 (m, 8H, CH_2_), 1.29 – 1.10 (m, 40H, CH_2_), 0.83 (t, J = 6.2 Hz, 6H, CH_3_).

**SEC (DMAc + 0.21 wt.% LiCl):** M_n_ = 3,900 g mol^-1^_;_ M_w_ = 4,300 g mol^-1^_;_ Đ = 1.11.

[B][Phe]_3_[C_12_]_3_[PEO_2k_-NH_3_] **19**

**19** was synthesised according to the procedure of compound **16**.

**Yield:** 181 mg, 0.061 mmol (86%), white solid.

**^1^H-NMR (300 MHz, d_6_-DMSO, 298 K):** δ [ppm] = 8.71 (t, J = 6.7 Hz, 3H, NH), 8.36 – 8.22 (m, 3H, CH_aromat_), 8.17 – 8.03 (m, 3H, NH), 7.70 (s, 2H, NH_2_), 7.36 – 7.06 (m, 15H, CH_aromat_), 4.80 – 4.60 (m, 3H, CH), 4.02 (t, J = 4.8 Hz, 2H, CH_2_), 3.72 (d, J = 5.2 Hz, 2H, CH_2_), 3.63 – 3.45 (m, 148H, PEO), 3.13 – 2.85 (m, 14H), 1.43 – 1.29 (m, 8H, CH_2_), 1.29 – 1.09 (m, 52H, CH_2_), 0.83 (t, J = 6.2 Hz, 6H, CH_3_).

**SEC (DMAc + 0.21 wt.% LiCl):** M_n_ = 3,900 g mol^-1^_;_ M_w_ = 4,600 g mol^-1^_;_ Đ = 1.17.

[B][U]_3_[C_12_]_3_[PEO_2k_-NH_3_] **20**

**20** was synthesised according to the procedure of compound **16**.

**Yield:** 76 mg, 0.003 mmol (99%), white solid.

**^1^H-NMR (300 MHz, d_6_-DMSO, 298 K):** δ [ppm] = 8.26 (s, 3H, CH_aromat_), 7.69 (s, 2H, NH_2_), 7.17 (t, J = 5.2 Hz, 1H, NH), 7.08 (s, 3H, NH), 5.94 (t, J = 4.9 Hz, 3H, NH), 4.07 – 3.96 (m, 2H, CH_2_), 3.73 (d, J = 4.9 Hz, 2H, CH_2_), 3.65 – 3.43 (m, 148H, PEO), 3.10 – 2.86 (m, 8H, CH_2_), 1.50 – 1.32 (m, 8H, CH_2_), 1.32 – 1.13 (m, 52H, CH_2_), 0.85 (t, J = 6.6 Hz, 6H, CH_3_).

**SEC (DMAc + 0.21 wt.% LiCl):** M_n_ = 3,800 g mol^-1^_;_ M_w_ = 4,500 g mol^-1^_;_ Đ = 1.18.

[B][Phe]_3_[C_6_]_3_[PEO_2k_-AF488] **1a**

Compound **19** (9.5 mg, 3.5 µmol, 1.0 eq) was dissolved in 27 µL DMF. 162 µL of a 42 mg/mL DIPEA stock solution in DMF (DIPEA: 9.16 µL, 52 µmol, 15 eq) were added and the solution stirred for 15 min at rt. To this, 280 µL of a 12.5 mg/mL NHS-AF488 stock solution in DMF (NHS-AF488: 3.5 mg, 4.2 µmol, 1.2 eq) was added and the reaction mixture stirred for 3 d at rt. To remove excess and unconjugated dye, the reaction mixture was diluted with water and dialysed against a 0.015 M NaCl solution for 9 days, followed by dialysis against water for 2 d. The desired dye-conjugate could then be obtained via lyophilisation.

The dye-conjugation efficiency was determined via the SEC-UV detector set to the emission maximum of AF488. With the knowledge of the initially used eq of NHS-AF488 and the different elution times of NHS-AF488 and the dye-conjugates, a calculation of the coupling efficiency was possible by comparing the area of the elution peaks.

**Yield:** 9.03 mg, 2.72 µmol (78%), orange powder.

**Dye content:** 14%

**SEC (DMAc + 0.21 wt.% LiCl):** M_n_ = 3,500 g mol^-1^_;_ M_w_ = 3,900 g mol^-1^_;_ Đ = 1.13.

[B][Phe]_3_[C_8_]_3_[PEO_2k_-AF488] **2a**

**2a** was synthesised according to the procedure of compound **1a**.

**Yield:** 11.63 mg, 3.41 µmol (98%), orange powder.

**Dye content:** 15%

**SEC (DMAc + 0.21 wt.% LiCl):** M_n_ = 2,100 g mol^-1^_;_ M_w_ = 2,800 g mol^-1^_;_ Đ = 1.34.

[B][Phe]_3_[C_10_]_3_[PEO_2k_-AF488] **3a**

**3a** was synthesised according to the procedure of compound **1a**.

**Yield:** 11.82 mg, 3.40 µmol (97%), orange powder.

**Dye content:** 14%

**SEC (DMAc + 0.21 wt.% LiCl):** M_n_ = 3,300 g mol^-1^_;_ M_w_ = 4,400 g mol^-1^_;_ Đ = 1.33.

[B][Phe]_3_[C_12_]_3_[PEO_2k_-AF488] **4a**

**4a** was synthesised according to the procedure of compound **1a**.

**Yield:** 11.06 mg, 3.09 µmol (88%), orange powder.

**Dye content:** 13%

**SEC (DMAc + 0.21 wt.% LiCl):** M_n_ = 3,200 g mol^-1^_;_ M_w_ = 5,200 g mol^-1^_;_ Đ = 1.62.

[B][U]_3_[C_12_]_3_[PEO_2k_-AF488] **5a**

**5a** was synthesised according to the procedure of compound **1a**.

**Yield:** 10.73 mg, 3.38 µmol (97%), orange powder.

**Dye content:** 11%

**SEC (DMAc + 0.21 wt.% LiCl):** M_n_ = 2,800 g mol^-1^_;_ M_w_ = 4,300 g mol^-1^_;_ Đ = 1.53.

[B][Phe]_3_[C_6_]_3_[PEO_2k_-AF594] **1b**

Compound **19** (4.14 mg, 1.5 µmol, 1.0 eq) was dissolved in 13 µL DMF. 70 µL of a 42 mg/mL DIPEA stock solution in DMF (DIPEA: 3.99 µL, 23 µmol, 15 eq) were added and the solution stirred for 15 min at rt. To this, 120 µL of a 12.5 mg/mL NHS-AF594 stock solution in DMF (NHS-AF594: 1.5 mg, 1.83 µmol, 1.2 eq) was added and the reaction mixture stirred for 3 d at rt. To remove excess and unconjugated dye, the reaction mixture was diluted with water and dialysed against a 0.015 M NaCl solution for 9 days, followed by dialysis against water for 2 d. The desired dye-conjugate could then be obtained via lyophilisation.

The dye-conjugation efficiency was determined via the SEC-UV detector set to the emission maximum of AF594. With the knowledge of the initially used eq of NHS-AF488 and the different elution times of NHS-AF594 and the dye-conjugates, a calculation of the coupling efficiency was possible by comparing the area of the elution peaks.

**Yield:** 3.62 mg, 1.09 µmol (72%), purple powder.

**Dye content:** 50%

**SEC (DMAc + 0.21 wt.% LiCl):** M_n_ = 2,700 g mol^-1^_;_ M_w_ = 3,200 g mol^-1^_;_ Đ = 1.17.

[B][Phe]_3_[C_8_]_3_[PEO_2k_-AF594] **2b**

**2b** was synthesised according to the procedure of compound **1b**.

**Yield:** 4.55 mg, 1.34 µmol (88%), purple powder.

**Dye content:** 100%

**SEC (DMAc + 0.21 wt.% LiCl):** M_n_ = 3,100 g mol^-1^_;_ M_w_ = 3,600 g mol^-1^_;_ Đ = 1.15.

[B][Phe]_3_[C_10_]_3_[PEO_2k_-AF594] **3b**

**3b** was synthesised according to the procedure of compound **1b**.

**Yield:** 4.99 mg, 1.44 µmol (94%), purple powder.

**Dye content:** 90%

**SEC (DMAc + 0.21 wt.% LiCl):** M_n_ = 2,700 g mol^-1^_;_ M_w_ = 3,100 g mol^-1^_;_ Đ = 1.17.

[B][Phe]_3_[C_12_]_3_[PEO_2k_-AF594] **4b**

**4b** was synthesised according to the procedure of compound **1b**.

**Yield:** 4.53 mg, 1.27 µmol (83%), purple powder.

**Dye content:** 90%

**SEC (DMAc + 0.21 wt.% LiCl):** M_n_ = 3,100 g mol^-1^_;_ M_w_ = 3,400 g mol^-1^_;_ Đ = 1.10.

[B][U]_3_[C_12_]_3_[PEO_2k_-AF594] **5b**

**5b** was synthesised according to the procedure of compound **1b**.

**Yield:** 4.8 mg, 1.51 µmol (99%), purple powder.

**Dye content:** 100%

**SEC (DMAc + 0.21 wt.% LiCl):** M_n_ = 2,600 g mol^-1^_;_ M_w_ = 2,900 g mol^-1^_;_ Đ = 1.09.

# Characterization

## FRET


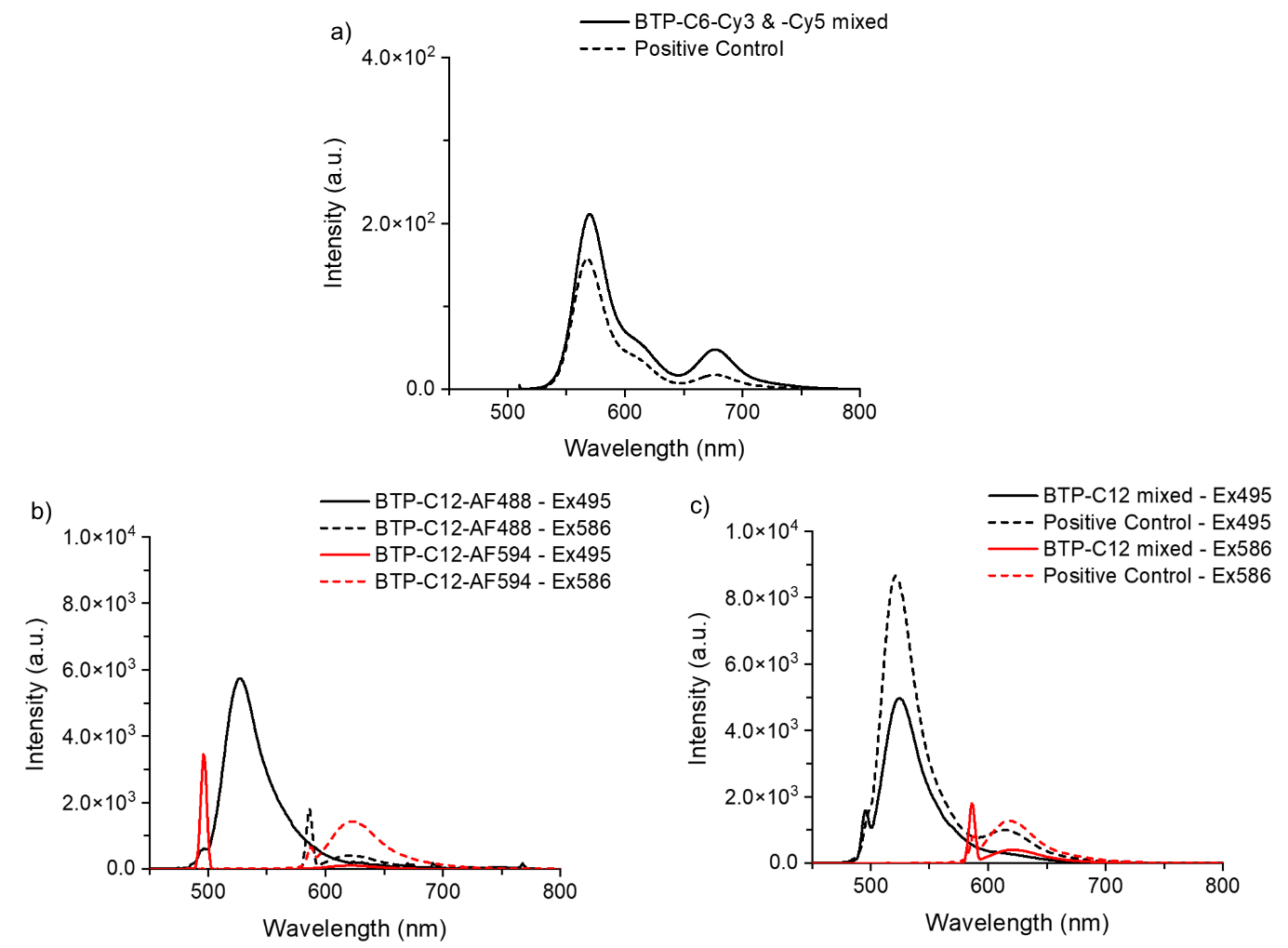


Supplementary Fig. 1. FRET measurements show the mixture of BTP-C6 fibers containing Cy3 and Cy5 immediately after mixing (a, c = 0.1 mg/mL) and BTP-C12 fibers containing either AF488 or AF594 (b), as well as a mixture of both (c, c = 1 mg/mL). An excitation wavelength of 550 nm was used for the Cy3 and Cy5 containing samples, and 495 nm and 586 nm for the AF488 and AF594 containing samples, respectively. For comparison, a preassembled positive control containing both dyes in one fiber is shown for both BTP systems.

## STED


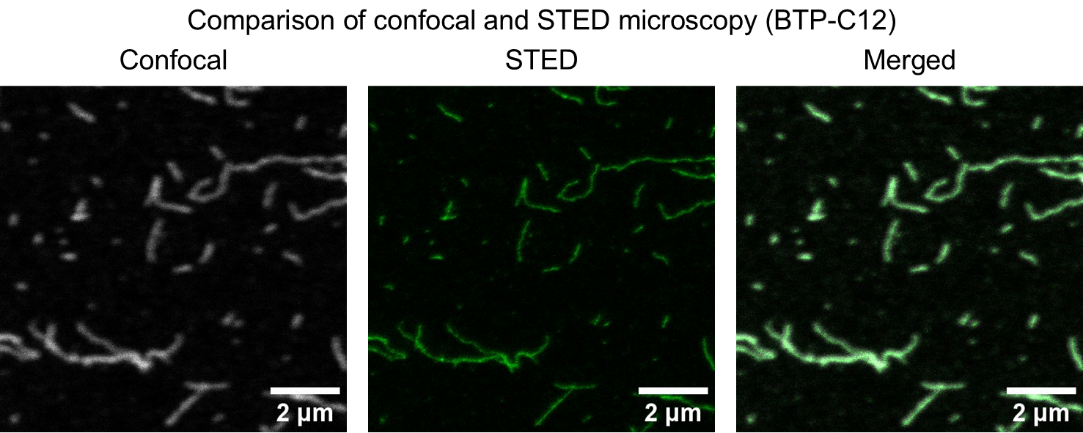


Supplementary Fig. 2. Confocal and STED images of a BTP-C12- AF488 sample. The final concentration of each solution was 0.2 mg mL^-1^ before the measurement.


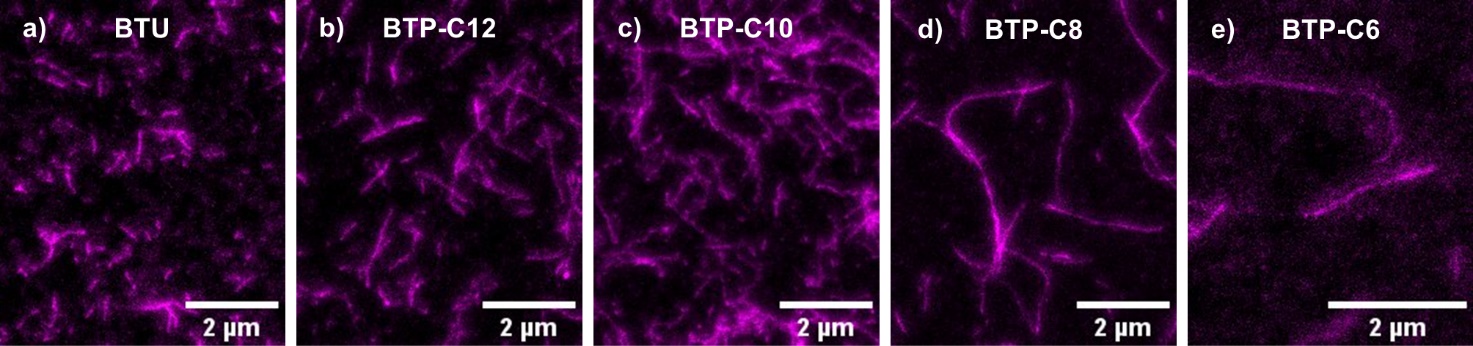


Supplementary Fig. 3. Additional STED images of the solutions containing AF594 of all BTP and BTU samples (a-e). The final concentration of each solution was 0.1 mg mL^-1^ before the measurement.


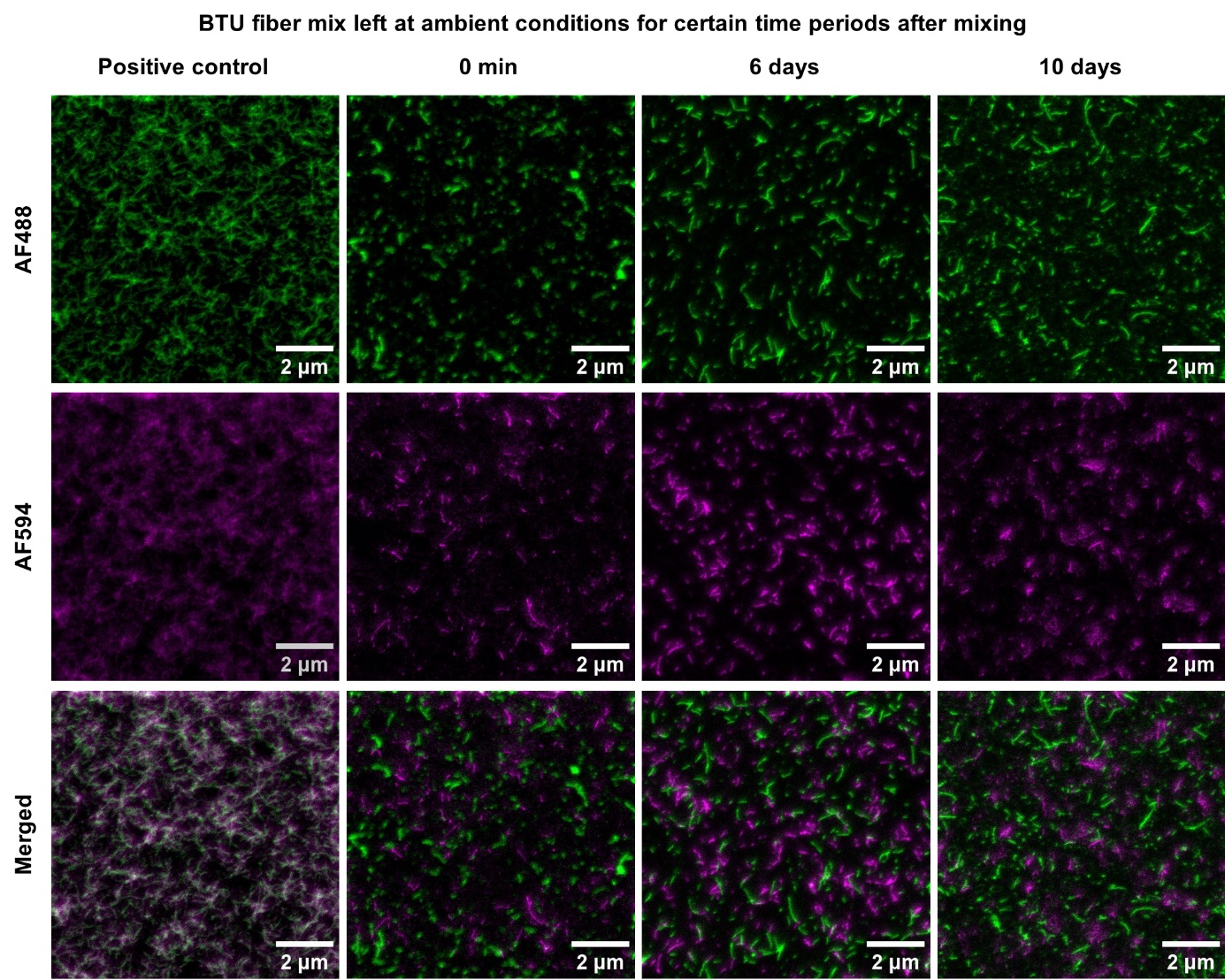


Supplementary Fig. 4. STED-microscopy images of BTU fibers containing AF488 (green) and AF594 (magenta) dyes. For the positive control the solutions were mixed before preassembly. Otherwise, the fibers were assembled separately, mixed post-assembly and measured after 0 min, 6 and 10 days. The final concentration of each solution was 0.1 mg mL-1 before the measurement.


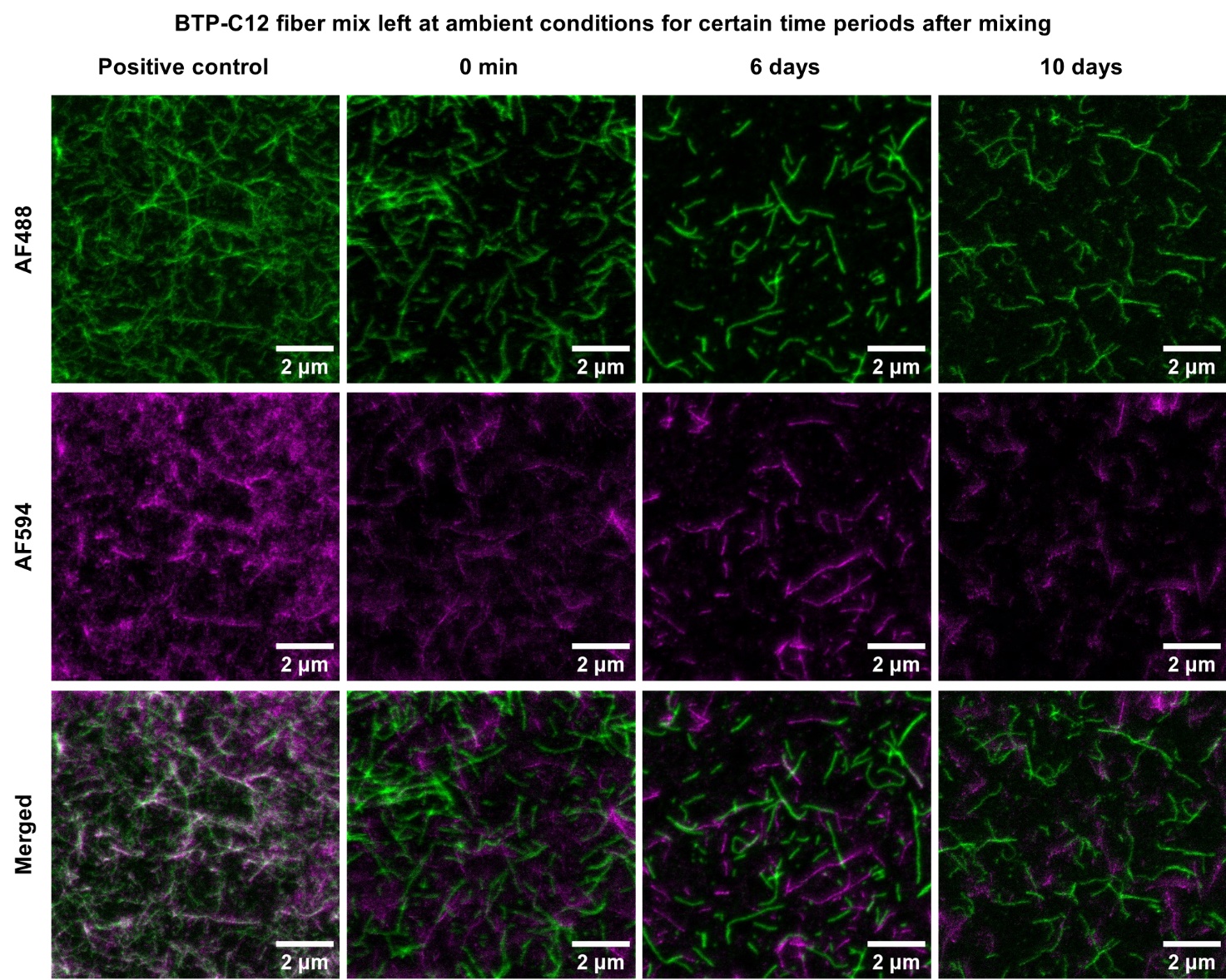


Supplementary Fig. 5. STED-microscopy images of BTP-C12 fibers containing AF488 (green) and AF594 (magenta) dyes. For the positive control the solutions were mixed before preassembly. Otherwise, the fibers were assembled separately, mixed post-assembly and measured after 0 min, 6 and 10 days. The final concentration of each solution was 0.1 mg mL-1 before the measurement.


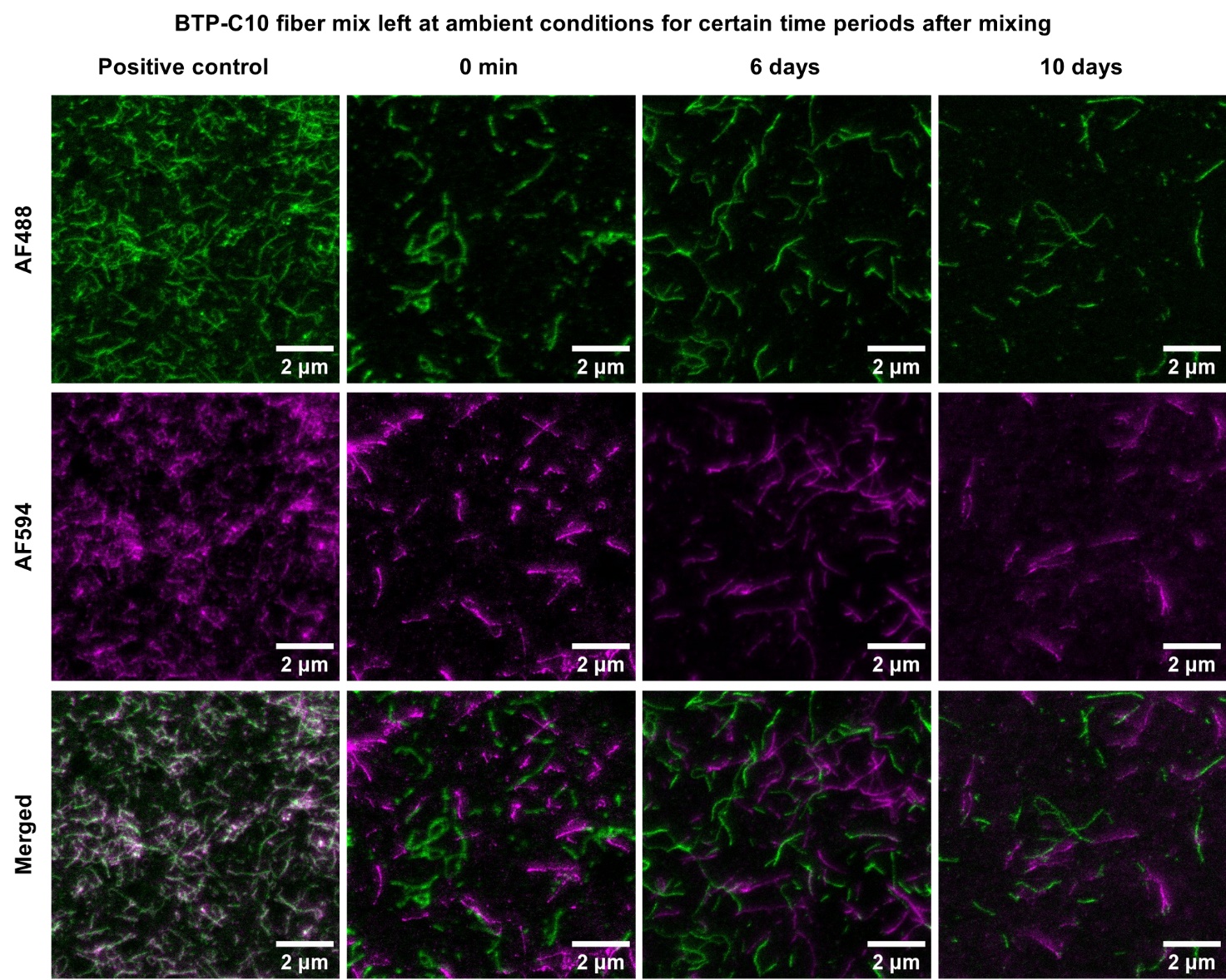


Supplementary Fig. 6. STED-microscopy images of BTP-C10 fibers containing AF488 (green) and AF594 (magenta) dyes. For the positive control the solutions were mixed before preassembly. Otherwise, the fibers were assembled separately, mixed post-assembly and measured after 0 min, 6 and 10 days. The final concentration of each solution was 0.1 mg mL-1 before the measurement.


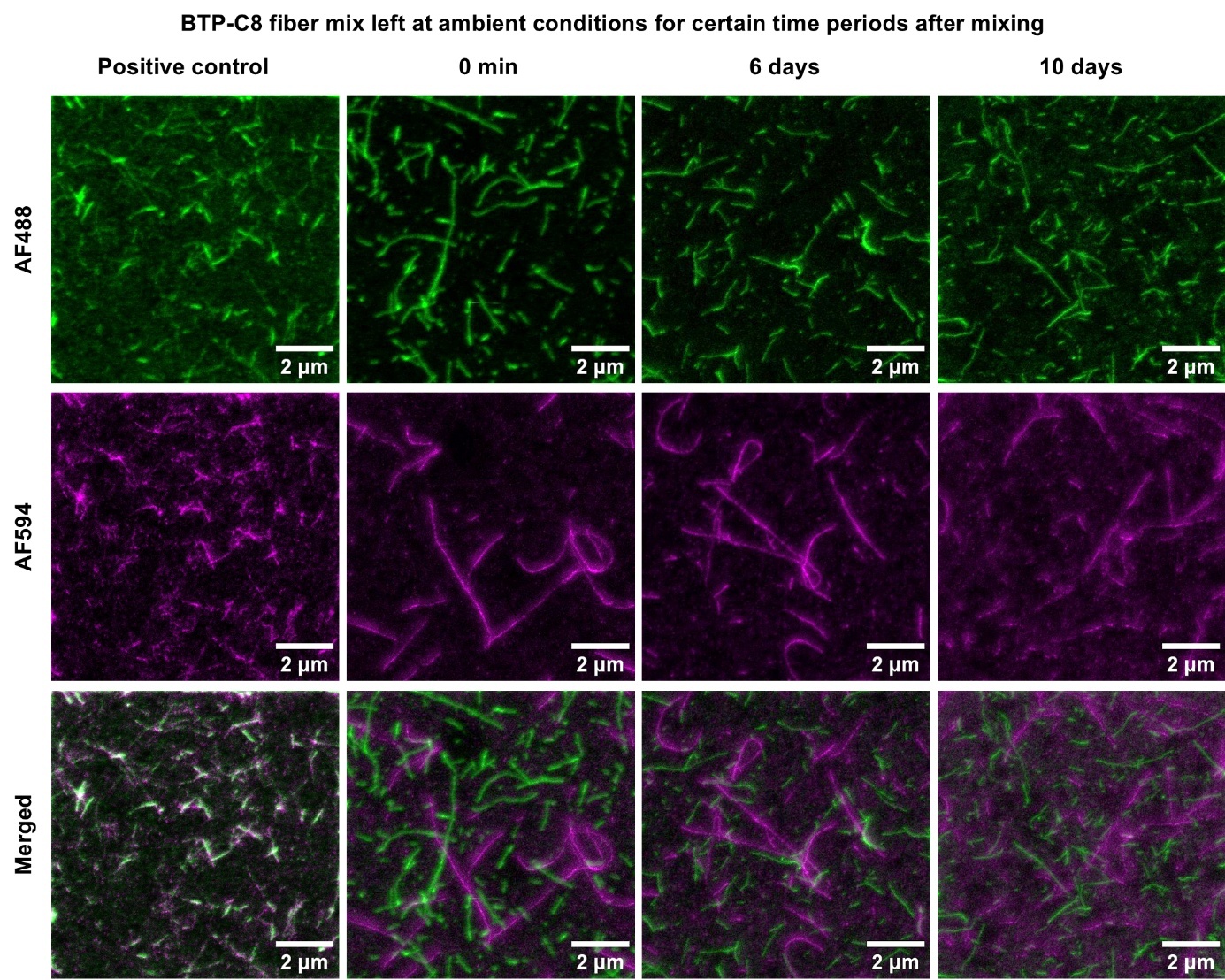


Supplementary Fig. 7. STED-microscopy images of BTP-C8 fibers containing AF488 (green) and AF594 (magenta) dyes. For the positive control the solutions were mixed before preassembly. Otherwise, the fibers were assembled separately, mixed post-assembly and measured after 0 min, 6 and 10 days. The final concentration of each solution was 0.1 mg mL-1 before the measurement.


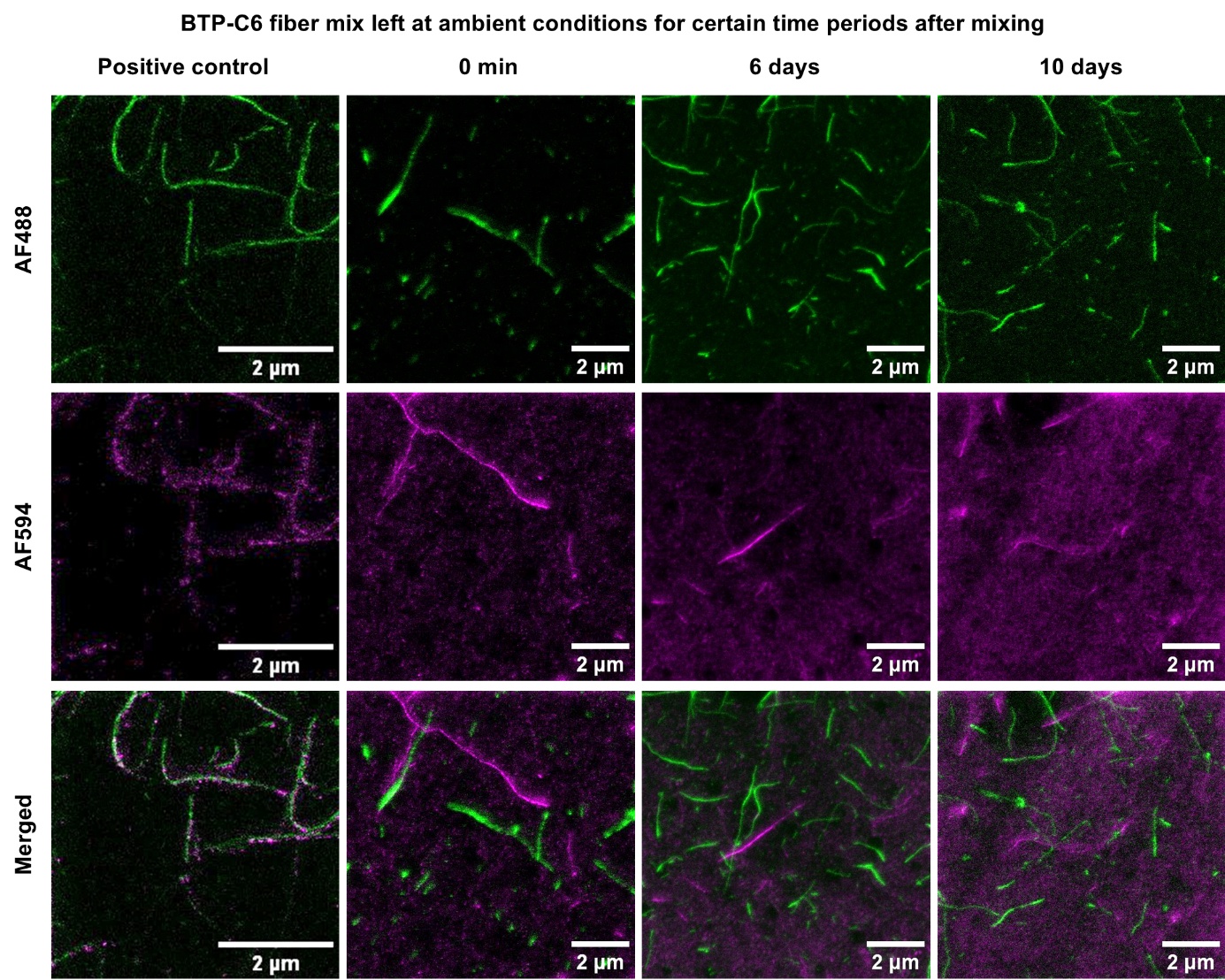


Supplementary Fig. 8. STED-microscopy images of BTP-C6 fibers containing AF488 (green) and AF594 (magenta) dyes. For the positive control the solutions were mixed before preassembly. Otherwise, the fibers were assembled separately, mixed post-assembly and measured after 0 min, 6 and 10 days. The final concentration of each solution was 0.1 mg mL-1 before the measurement.


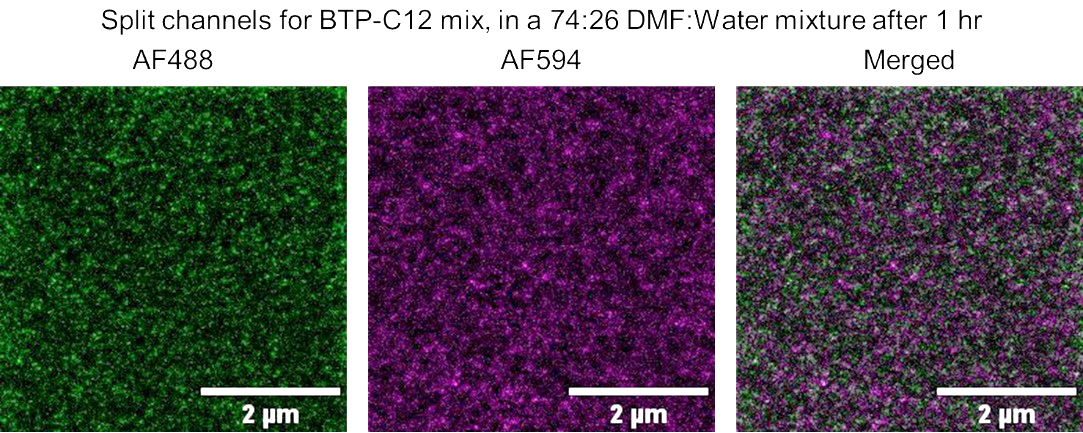


Supplementary Fig. 9. STED-microscopy images of BTP-C12 fibers mix containing AF488 (green) and AF594 (magenta) dyes in a solvent mixture of DMF:Water 74:26. The fibers were assembled separately, mixed post-assembly diluted with DMF and measured after 1 hr.


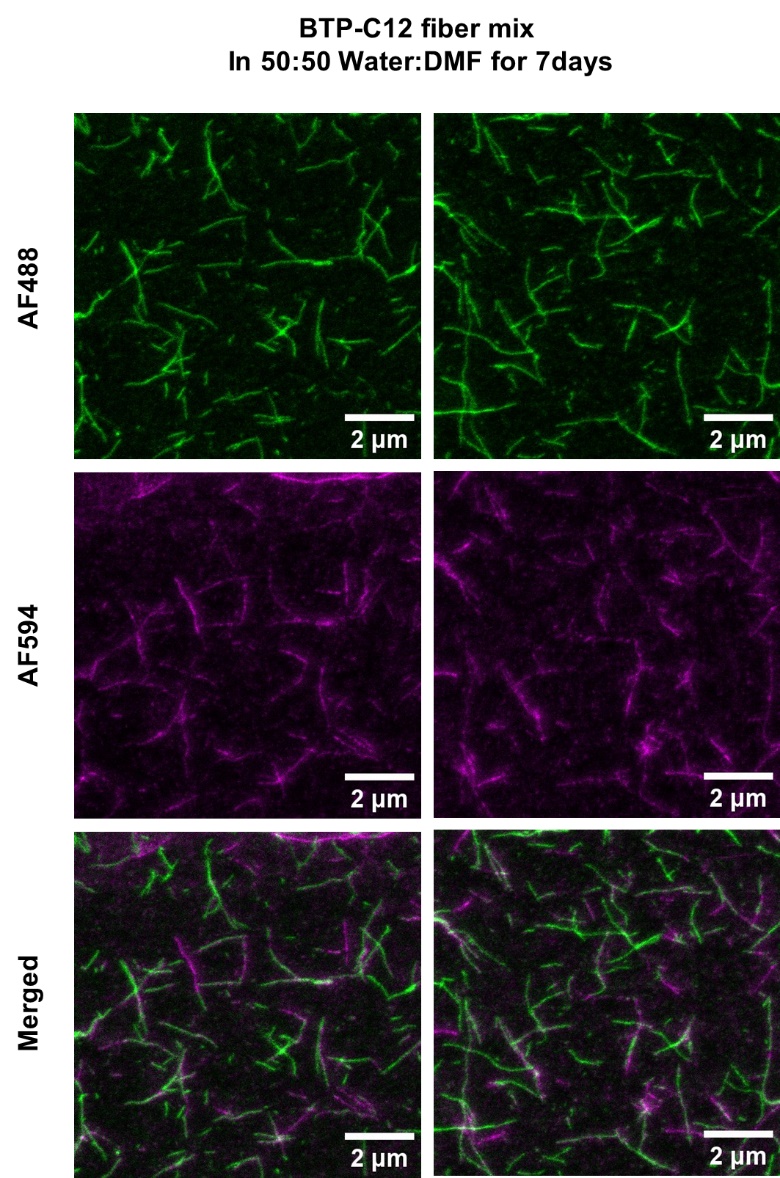


Supplementary Fig. 10. STED-microscopy images of BTP-C12 fibers containing AF488 (green) and AF594 (magenta) dyes. The fibers were assembled separately, mixed post-assembly diluted with DMF and measured after 7 days. The final concentration of each solution was 0.1 mg mL-1 before the measurement.


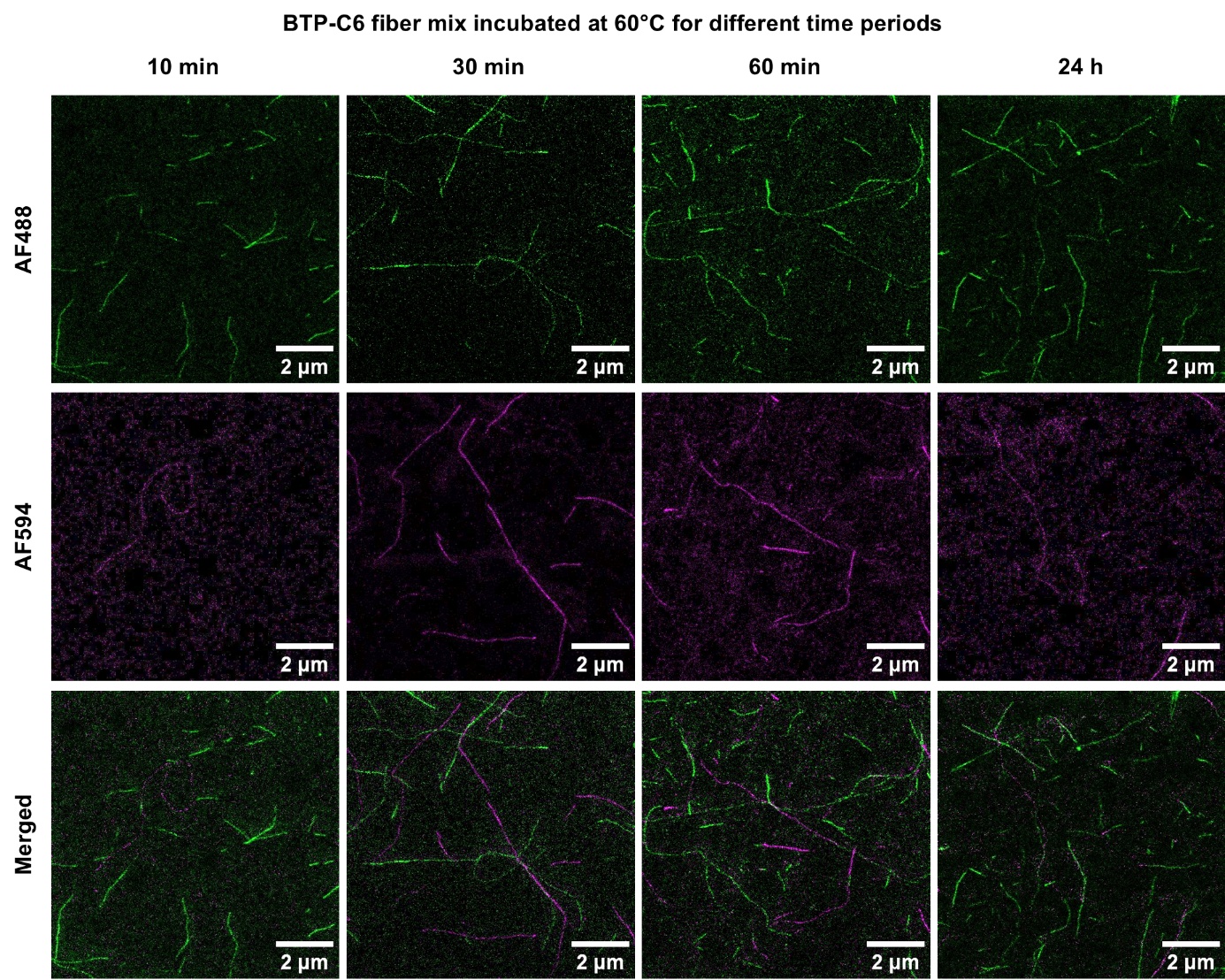


Supplementary Fig. 11. STED-microscopy images of BTP-C6 fibers containing AF488 (green) and AF594 (magenta) dyes. The fibers were assembled separately, mixed post-assembly and incubated for 10, 30 , 60 min and 1 days at 60°C. The final concentration of each solution was 0.1 mg mL-1 before the measurement.


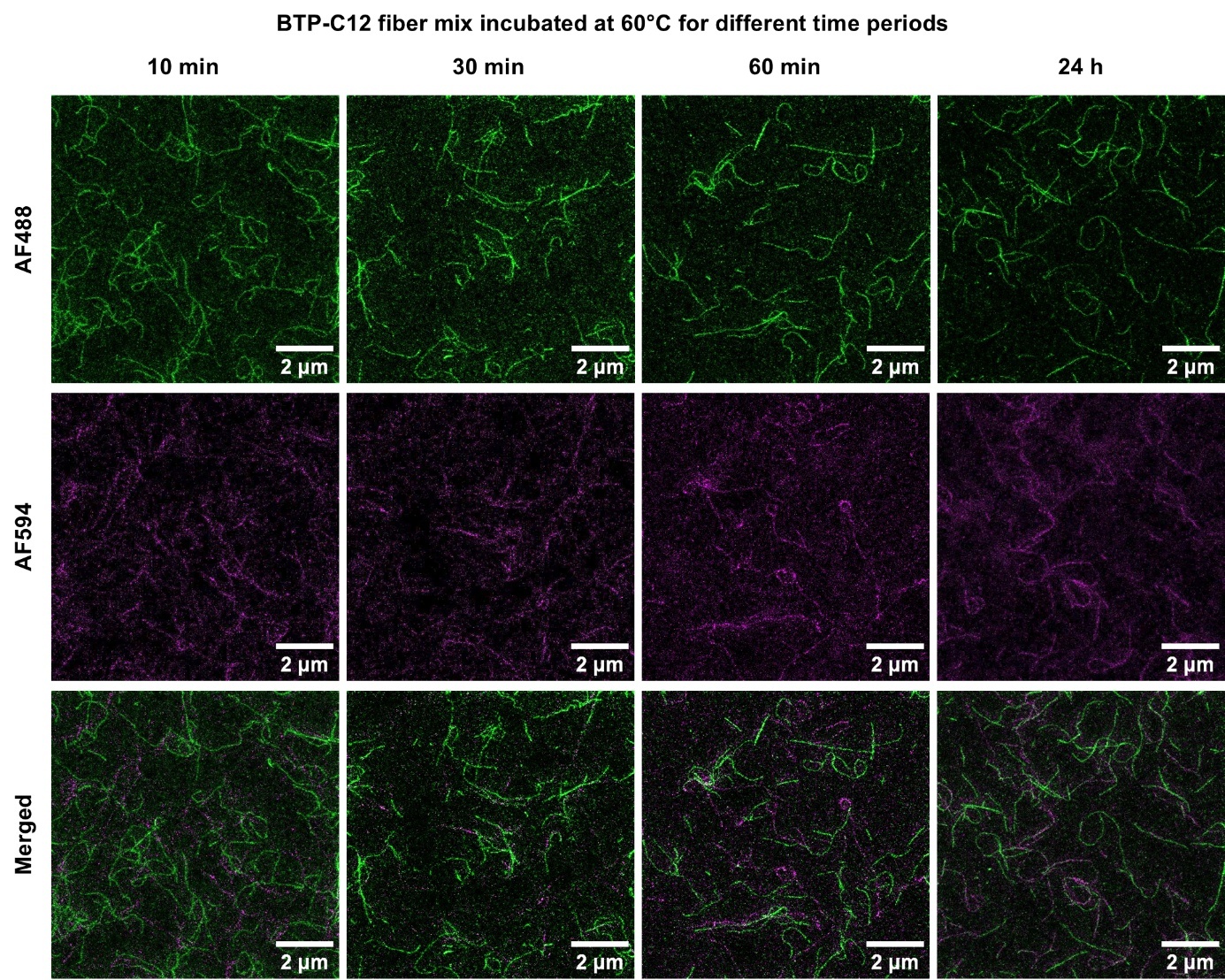


Supplementary Fig. 12. STED-microscopy images of BTP-C12 fibers containing AF488 (green) and AF594 (magenta) dyes. The fibers were assembled separately, mixed post-assembly and incubated for 10, 30 , 60 min and 1 days at 60°C. The final concentration of each solution was 0.1 mg mL-1 before the measurement.

## Automated image analysis using a JIPipe workflow


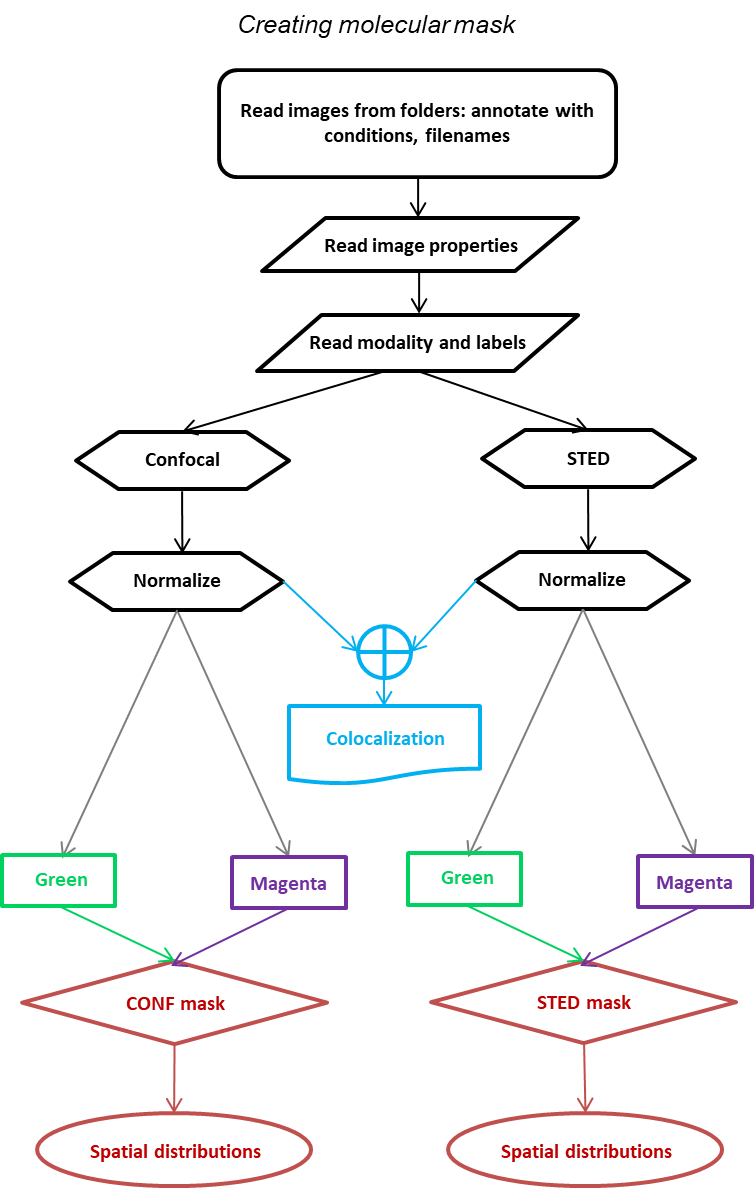


Supplementary Fig. 13. Algorithmic pipeline to calculate the molecular masks. The .msr-format native files were read into JIPipe^[5]^ and the image content was annotated according to the experimental conditions (black round-edge rectangle). The image properties (width, height, voxel size), the imaging modalities (confocal or STED), and the labeling colors (green or magenta) were placed into annotations (black parallelograms). The images were separated into confocal (black elongated hexagon, left) and STED (black elongated hexagon, right) modalities, followed by normalization (black elongated hexagons). The green and magenta normalized images were analyzed for colocalization (cyan modules). The merged green (green rectangle) and magenta (magenta rectangle) confocal and STED images (left and right, resp.) were merged to form a confocal and STED mask (brown rhombus left and right, resp.). The merged masks were later used to study the spatial distributions of the two types of nanoparticles (see Supplementary Fig. 14).


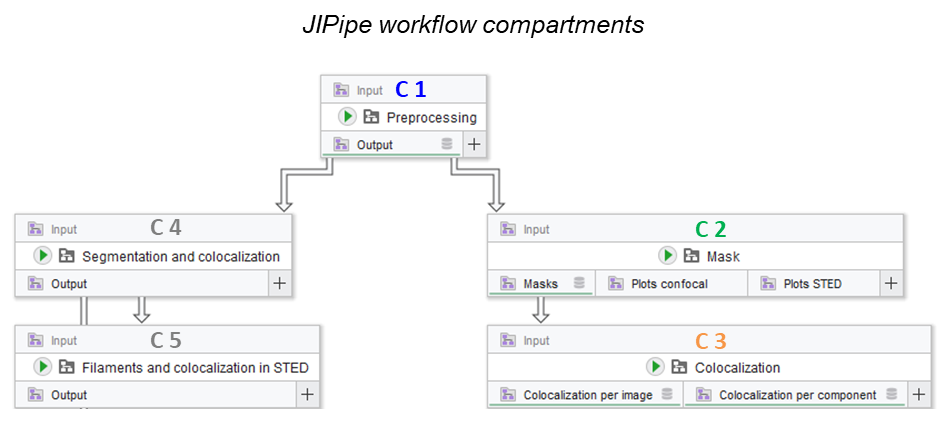


Supplementary Fig. 14. JIPipe compartments of the analysis workflow. The image preparation, preprocessing, and quantification steps were separated into compartments, which consist of groups of nodes that are functionally linked. The current pipeline utilized the first three compartments (C1, C2, and C3, in blue, green, and orange, resp.) to read and annotate the images (C1, blue), to create the confocal and STED image-based masks (C2, green), and to calculate the colocalization coefficients (C3, orange).


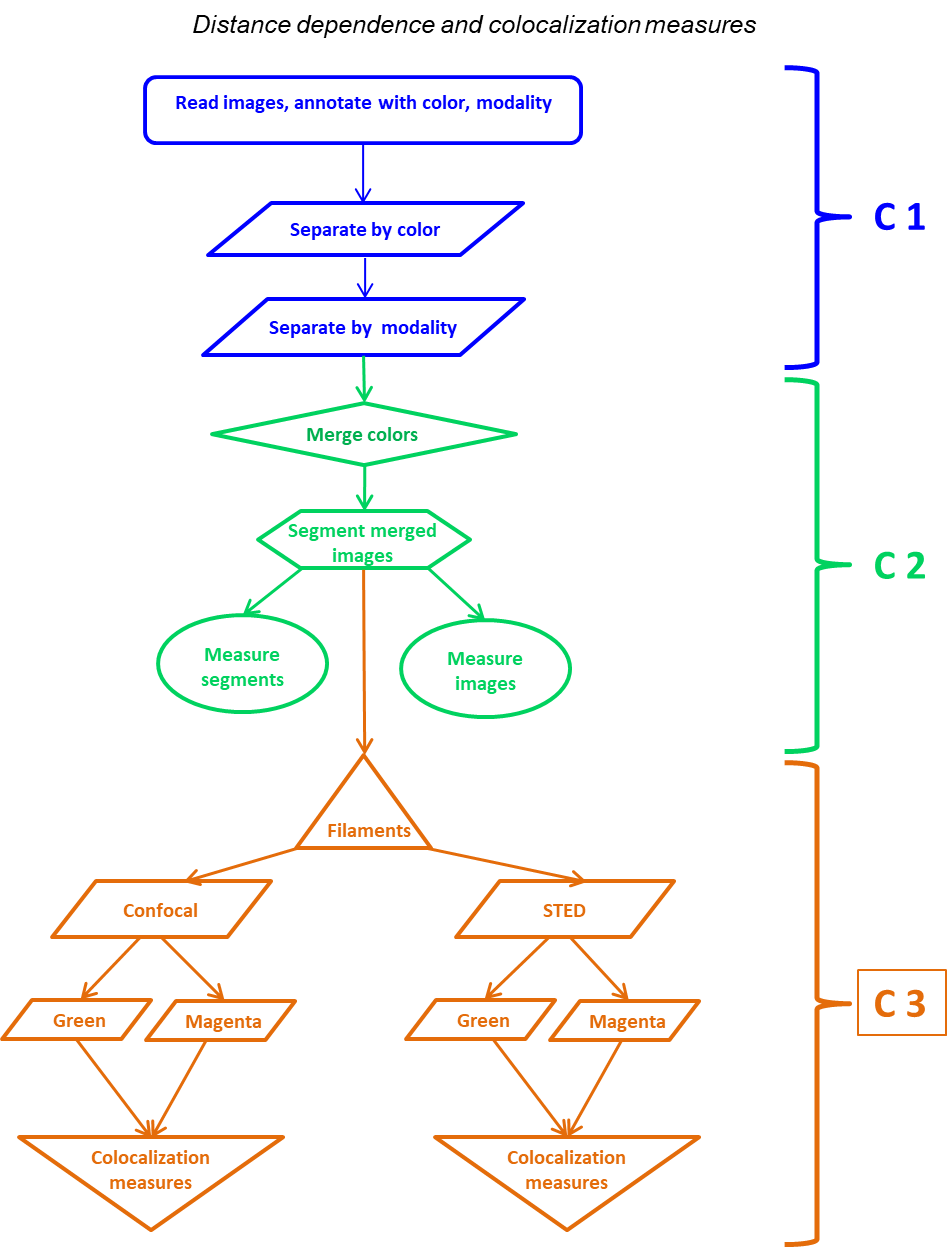


Supplementary Fig. 15. The analysis pipeline of the distance measurements and the colocalization measures. The first compartment (C1, blue) read and annotated the relevant images (round-edge rectangle), and separated them by label color and microscopy modality (blue parallelograms). The second compartment (C2, green) used the merged color channels (green rhombus) to segment the images (green elongated hexagon) and to measure their properties per molecule (green ellipsoid, left) or per image (green ellipsoid, right). The third compartment (C3, orange) used the segmented individual filaments (orange triangle) to calculate the colocalization measures (orange inverted triangles) per modality and per color (orange parallelograms).

**
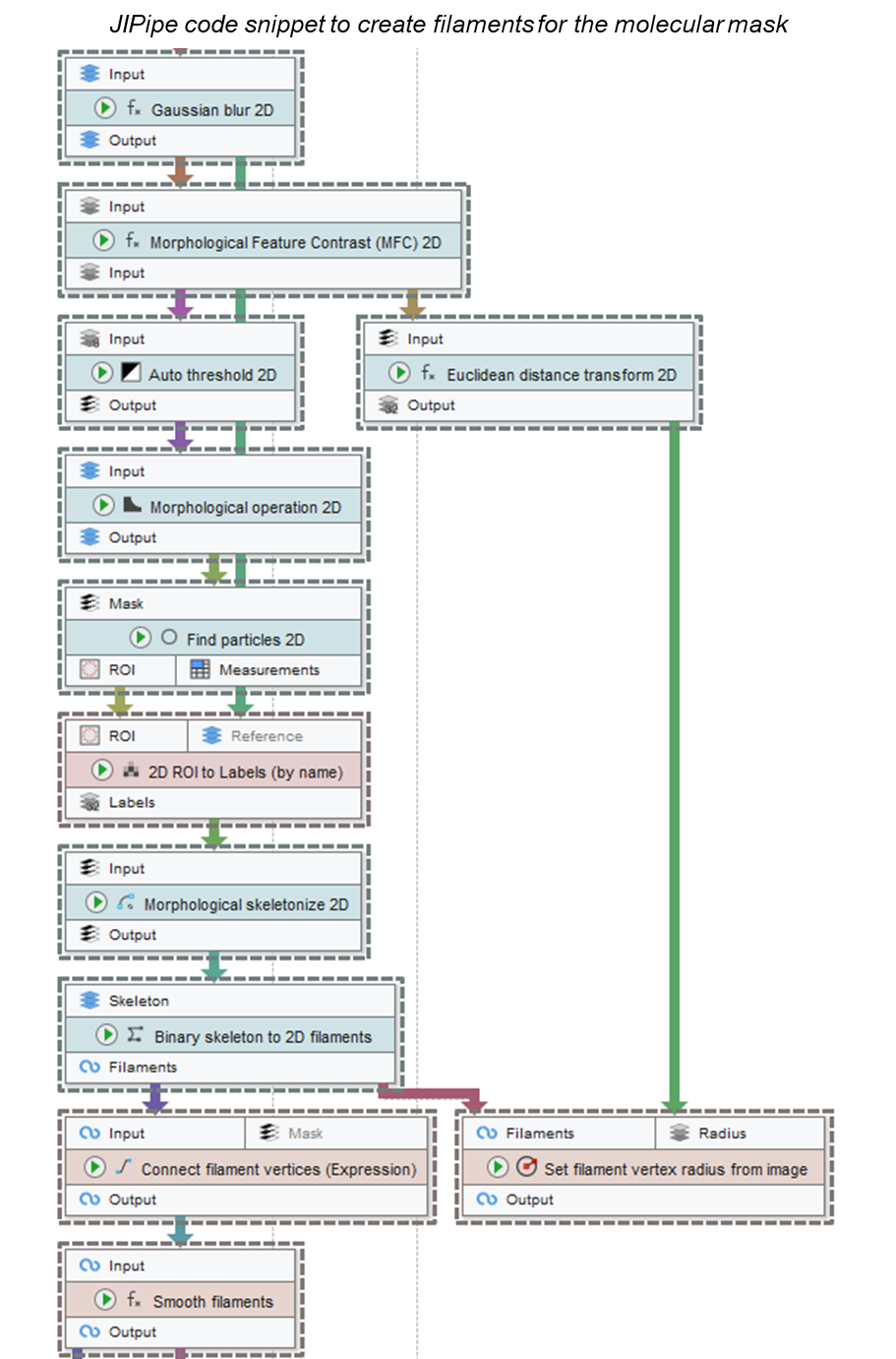
**

Supplementary Fig. 16. Excerpt of the JIPipe node structure of the mask creation process. The base of the pipeline was formed by a succession of Gaussian blurring (sigma = 2 px), Morphological Feature Contrast^[7]^ filtering, and a series of basic processing steps. The detailed description of all the nodes can be found in Chapter 5.


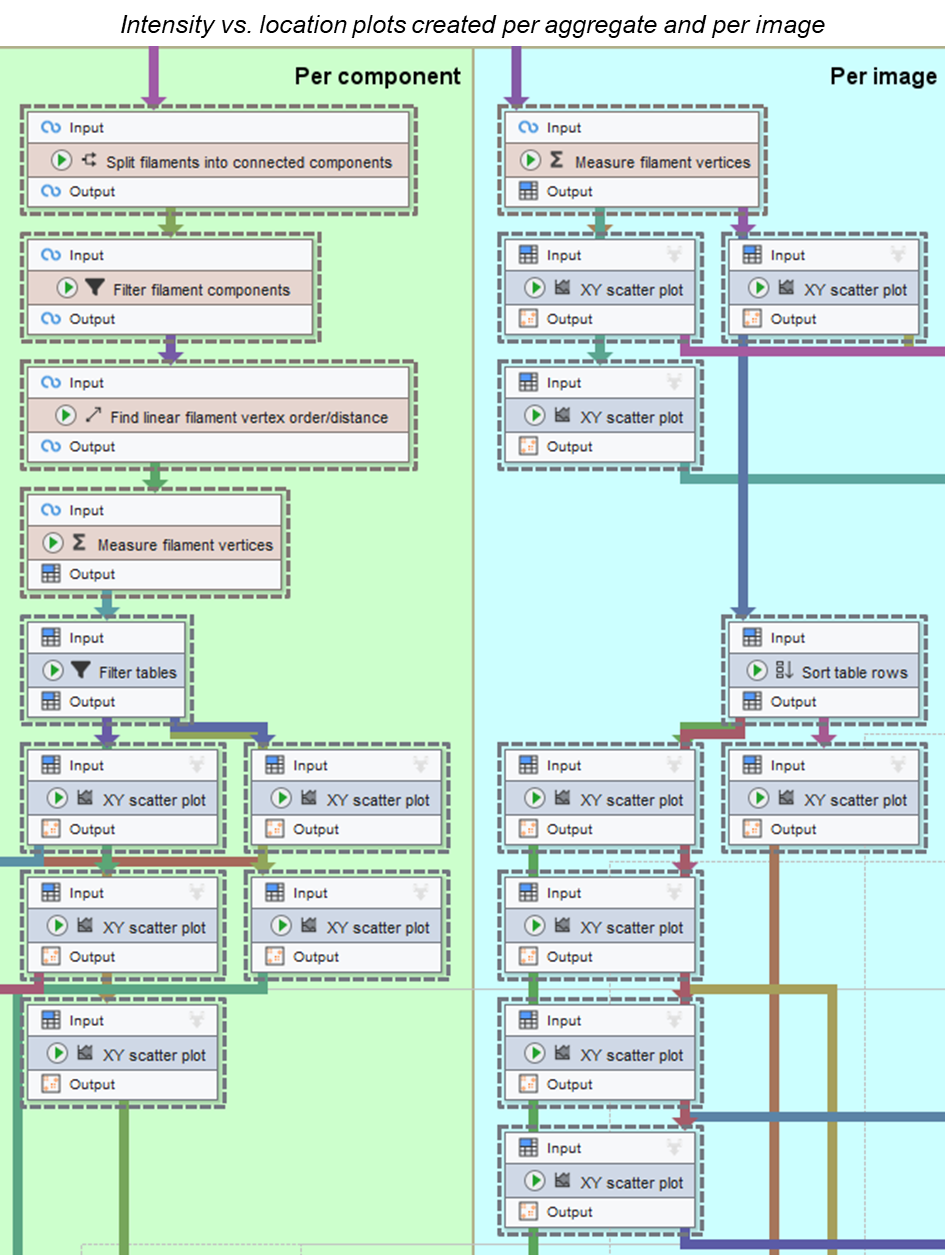


Supplementary Fig. 17. The JIPipe node structure that generates the color intensity plots as a function of location along the individual molecules. The plots show the intensity vs. location dependence both per molecular component (left, green background) and per image (right, cyan background). The detailed description of all the nodes can be found in Chapter 5.


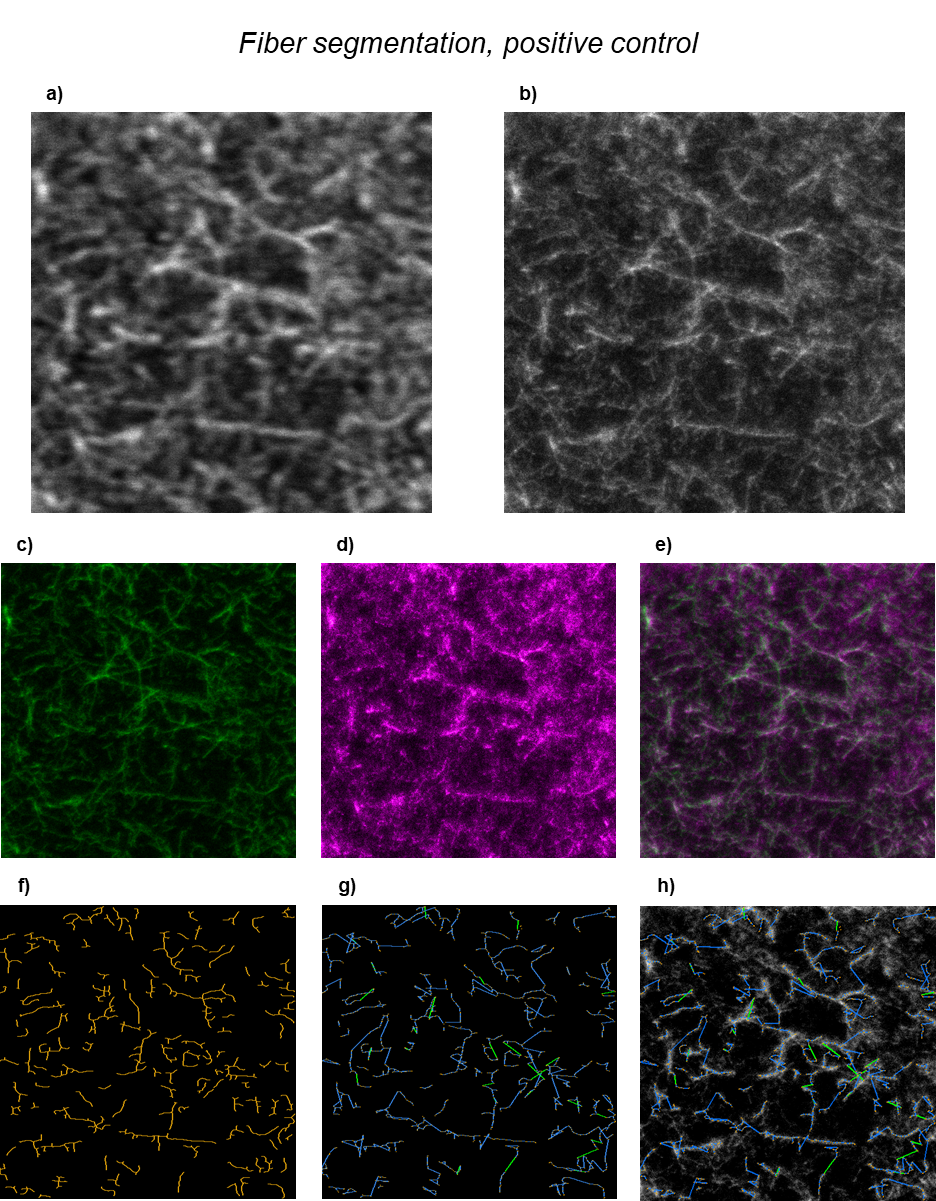


Supplementary Fig. 18. Intermediate results of the fiber segmentation and automated mask generation process for image BTP-C12 from the positive control group. The green and magenta images were merged to create a starting point for the mask generation of the confocal (a) and STED (b) images. The component images were normalized before the merging. Normalized STED images of an example from the positive control dataset, showing the AF488 (c, green) and AF594 (d, magenta) images, as well as the overlay (e). The JIPipe workflow was applied to create the first rough approximation of the filaments (f, orange) to be fitted on the individual molecules, using the merged images from panels a and b. After the correction steps applied to these filaments (g, correction elements in green, first approximation segments in blue), the final mask followed the original images well (h).


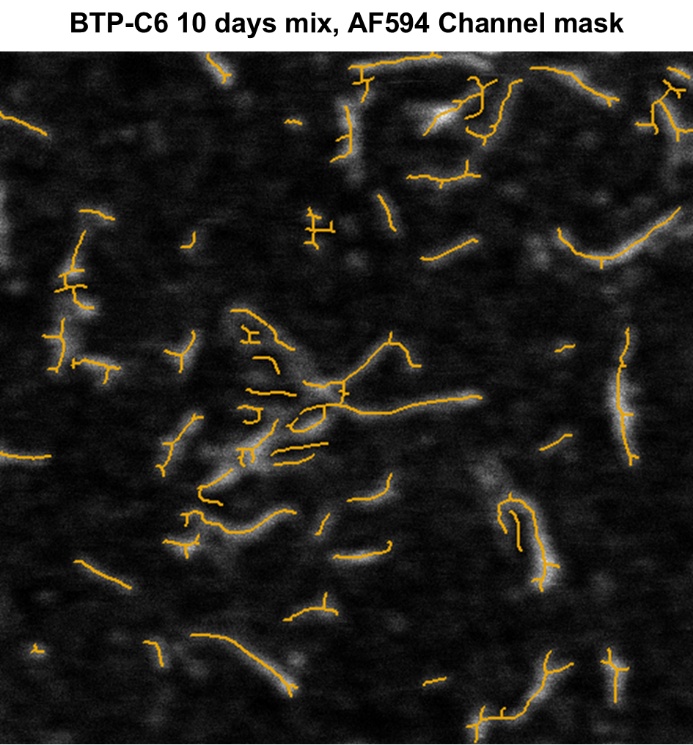


Supplementary Fig. 19. Intermediate results of the automated mask generation process for image BTP-C6 10-day samples, AF594 channel. The JIPipe workflow was applied to create the first rough approximation of the filaments (orange) to be fitted on the individual molecules.


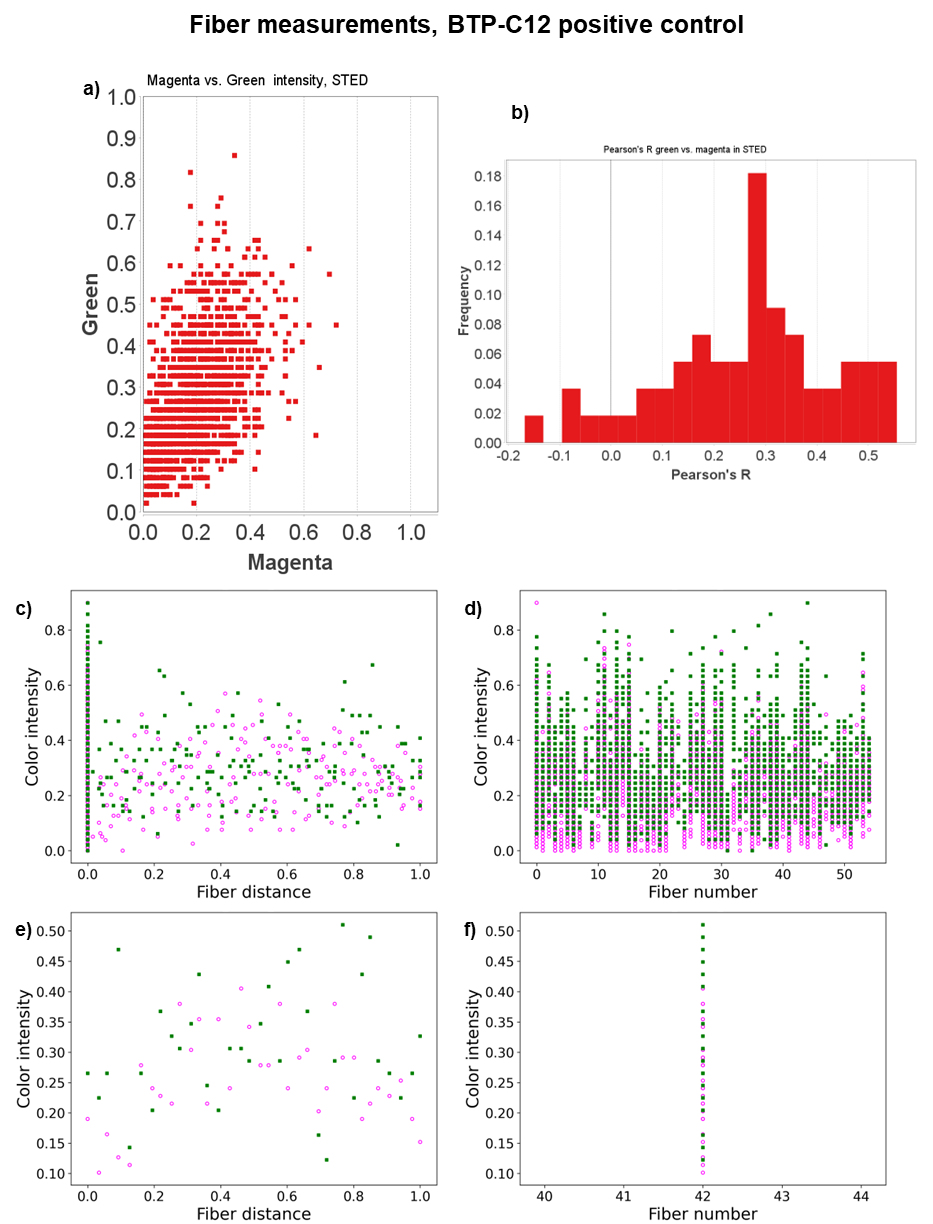


Supplementary Fig. 20. Representative results of the quantitative molecular measurements from the BTP-C12 image in the positive control group. a) The green and magenta intensities matched each other well, following a near 45-degree trendline, indicating the homogenous separated distribution of the two components, as opposed to forming intermolecular segment swaps. b) The Pearson’s R coefficients were calculated between the green and magenta intensity values inside the masks as shown in Supplementary Fig. 17. The positive R value range with a peak at 0.45 (the per-image average R coefficient values were between 0.40 and 0.60) also indicates a strong positive correlation between the two colors, indicating no block formation. The per-vertex intensities for the green and magenta channels (filled circles with the corresponding color) along individual molecules as a function of the distance from the start of the molecule (c) and alongside the molecules plotted per fiber number (d). In this image, 48 molecules were identified. The fiber distance shows the position along the fiber normalized to the total length of the molecule. e & f) Example plots from individual molecules showing the same arrangements as in (c & d).


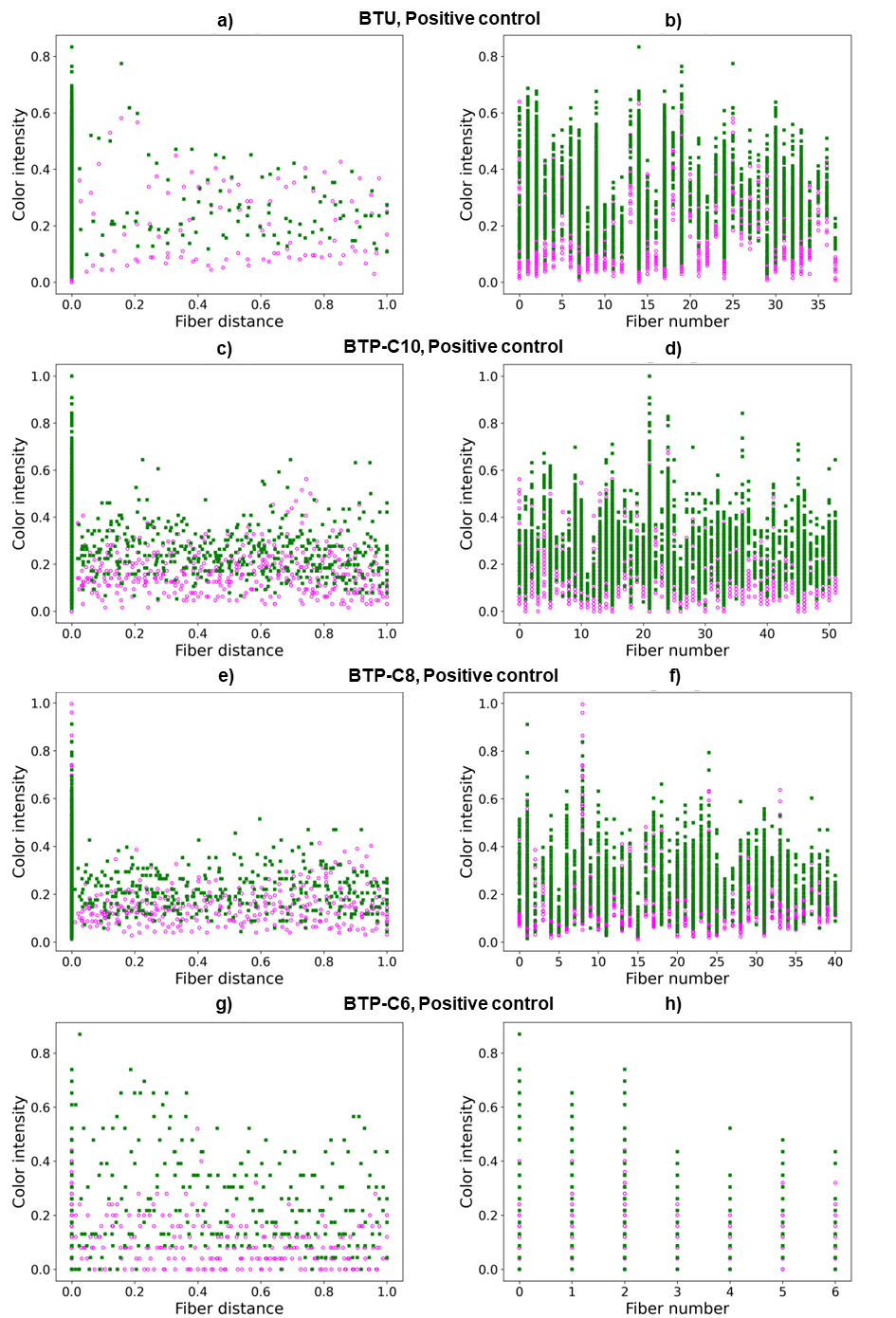


Supplementary Fig. 21.The per-vertex intensities for the green and magenta channels along individual molecules as a function of the distance from the start of the molecule (a, c, e, g) and alongside the molecules plotted per fiber number (b, d, f, h) for all positive controls.


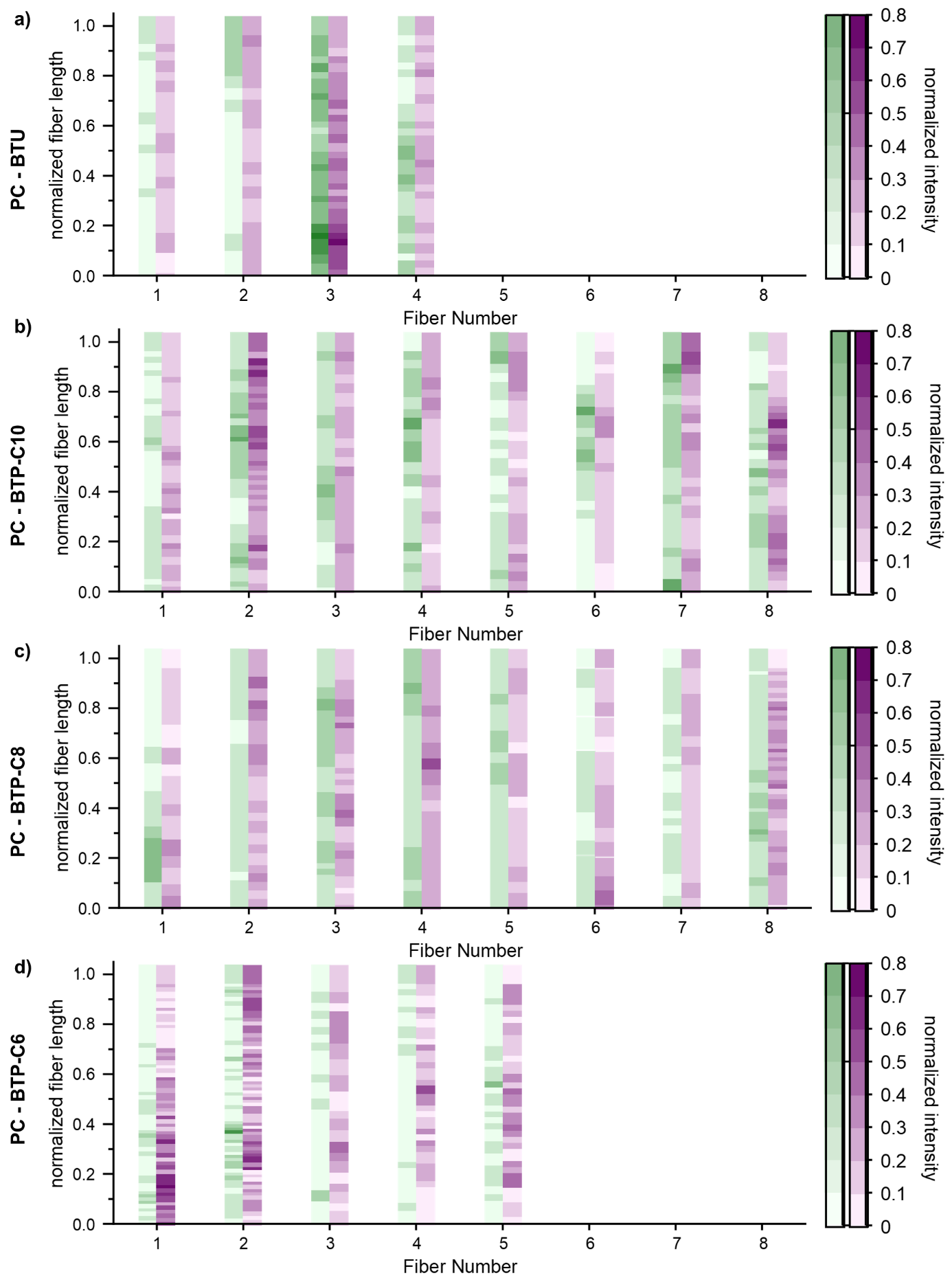


Supplementary Fig. 22. Overview of the relation between the normalized intensities for the AF488 (green) and AF594 (magenta) channels, for multiple points alongside representative fiber from one image of the BTU (a), BTP-C10 (b), BTP-C8 (c) and BTP-C6 (d) positive controls.


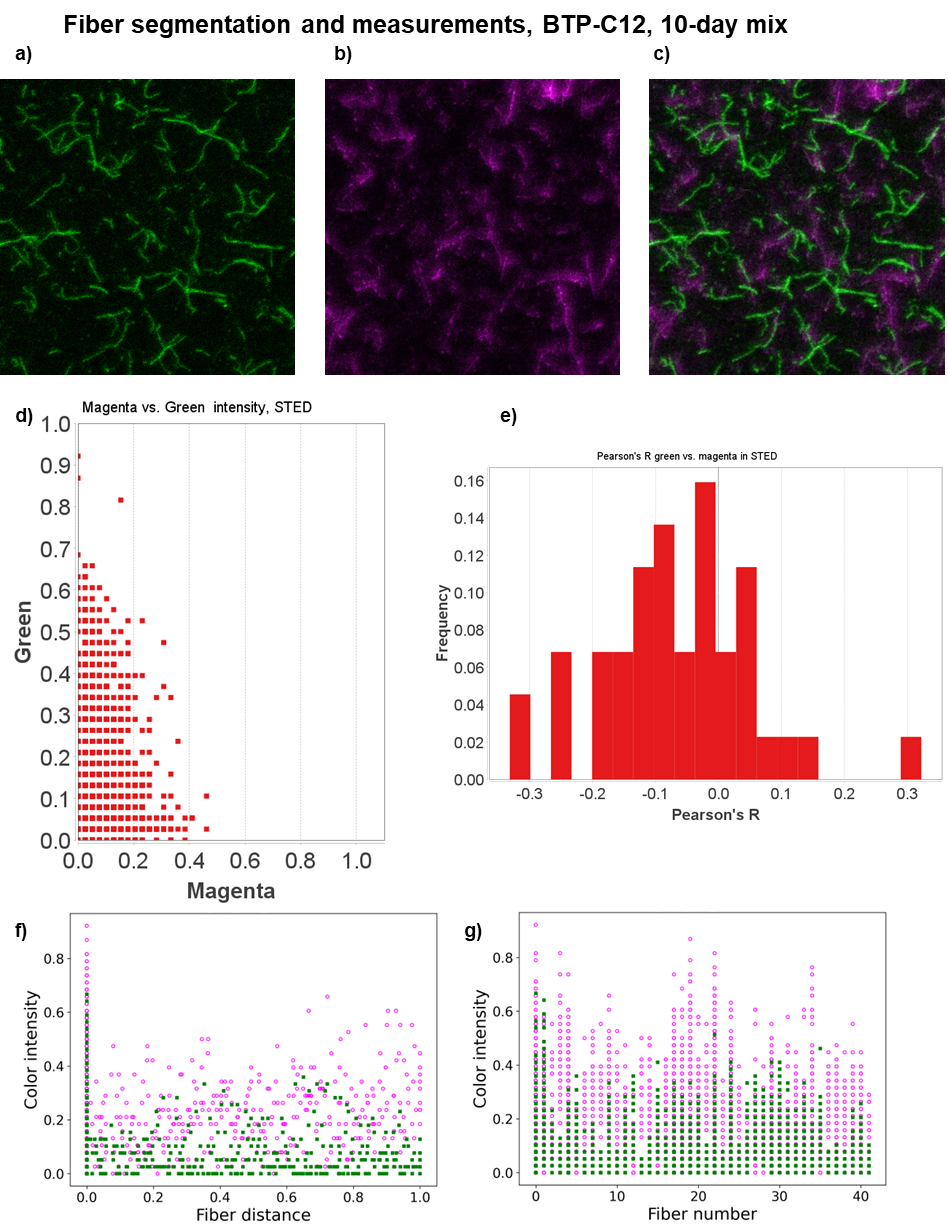


Supplementary Fig. 23. Representative results of the quantitative molecular measurements from the BTP-C12 image in the 10-day mix group. Representation of the automatically processed AF488 (a) and AF594 (b) dye channels, as well as their overlay. Same arrangement as in Supplementary Fig. 17. The green-magenta correlation (d) and the per-condition Pearson’s coefficient distribution (e) for the example image. Same arrangement as in Supplementary Fig. 20 a & b. /f & g) are the same as c & d in Supplementary Fig. 20, for the positive control.


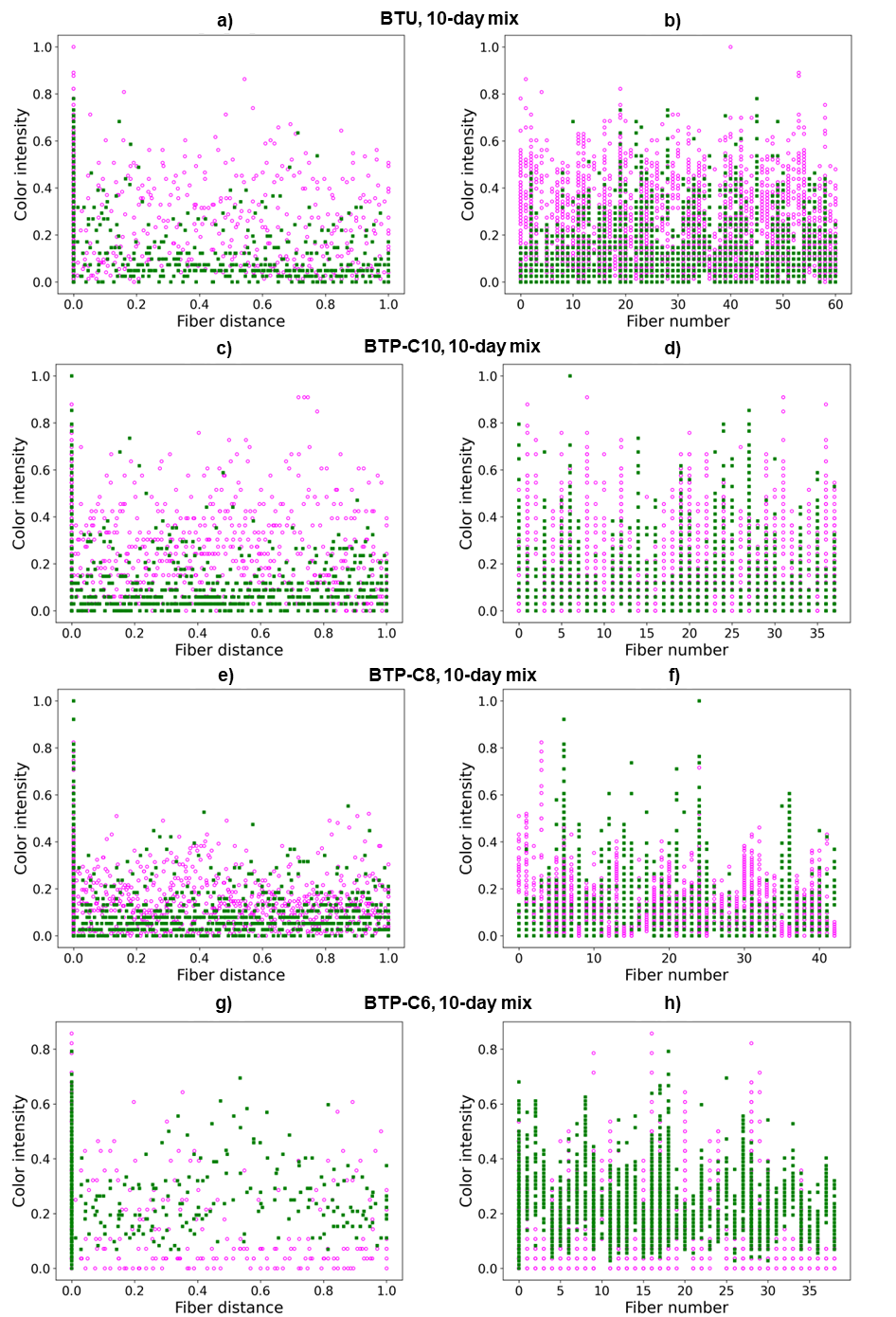


Supplementary Fig. 24. The per-vertex intensities for the green and magenta channels (filled circles with the corresponding color) along individual molecules as a function of the distance from the start of the molecule (a, c, e, g) and alongside the molecules plotted per fiber number (b, d, f, h) for all samples mixed for 10 days.


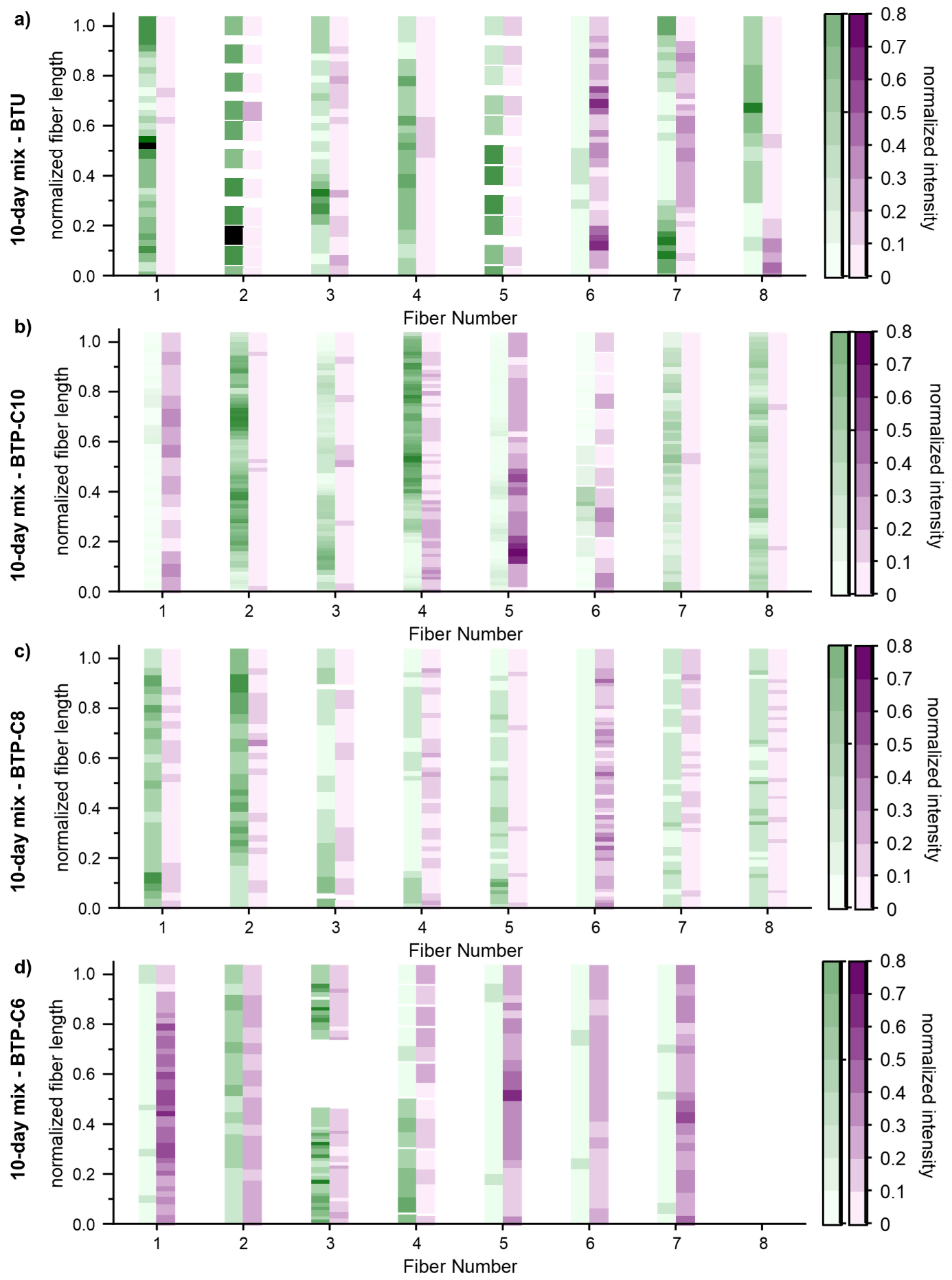


Supplementary Fig. 25: Overview of the relation between the normalized intensities for the AF488 (green) and AF594 (magenta) channels, for multiple points alongside representative fiber from one image of the BTU (a), BTP-C10 (b), BTP-C8 (c) and BTP-C6 (d) 10-day mixed sample.


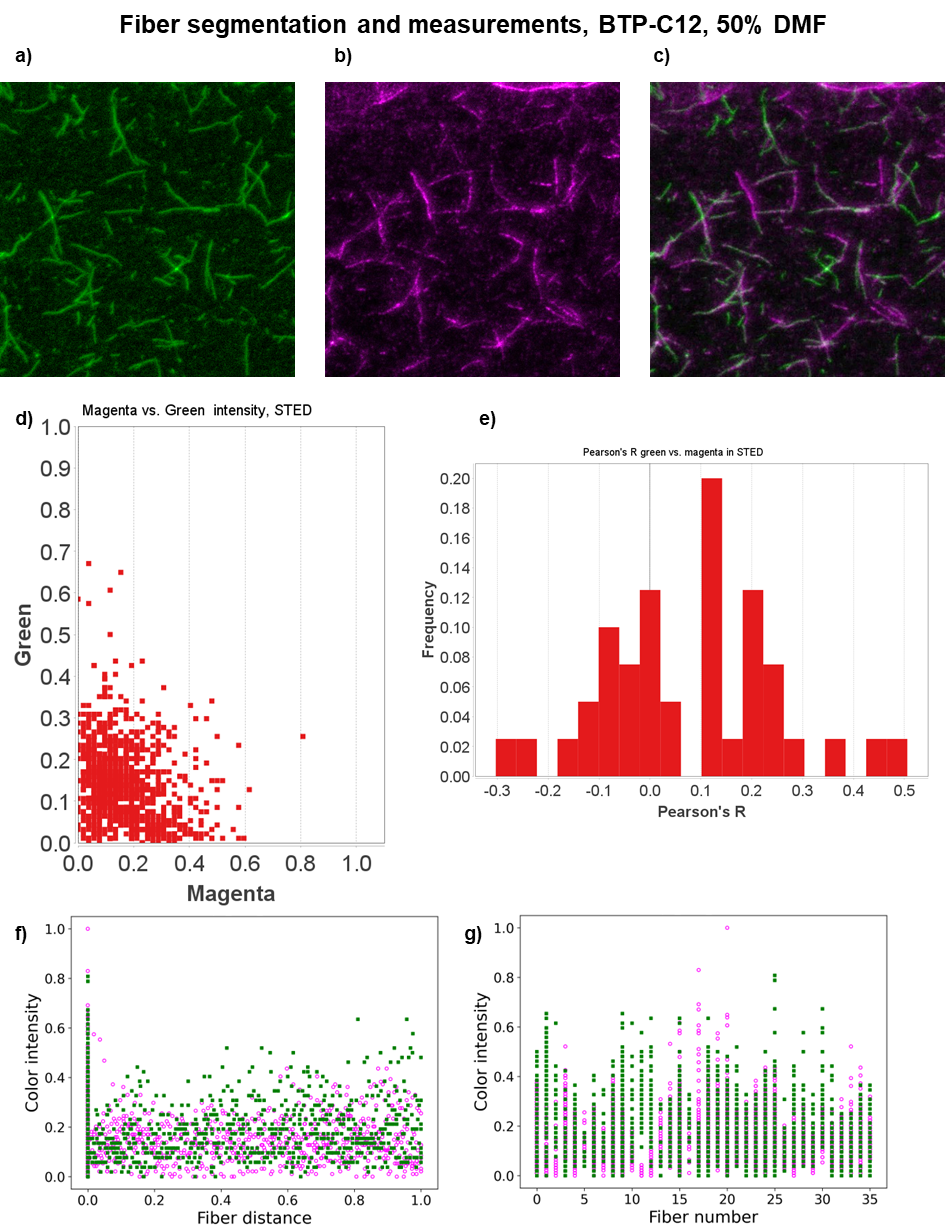


Supplementary Fig. 26. Representative results of the quantitative molecular measurements from the BTP-C12 image in the 50% DMF group. a -g) As in Supplementary Fig. 23, for the 50% DMF group.

**
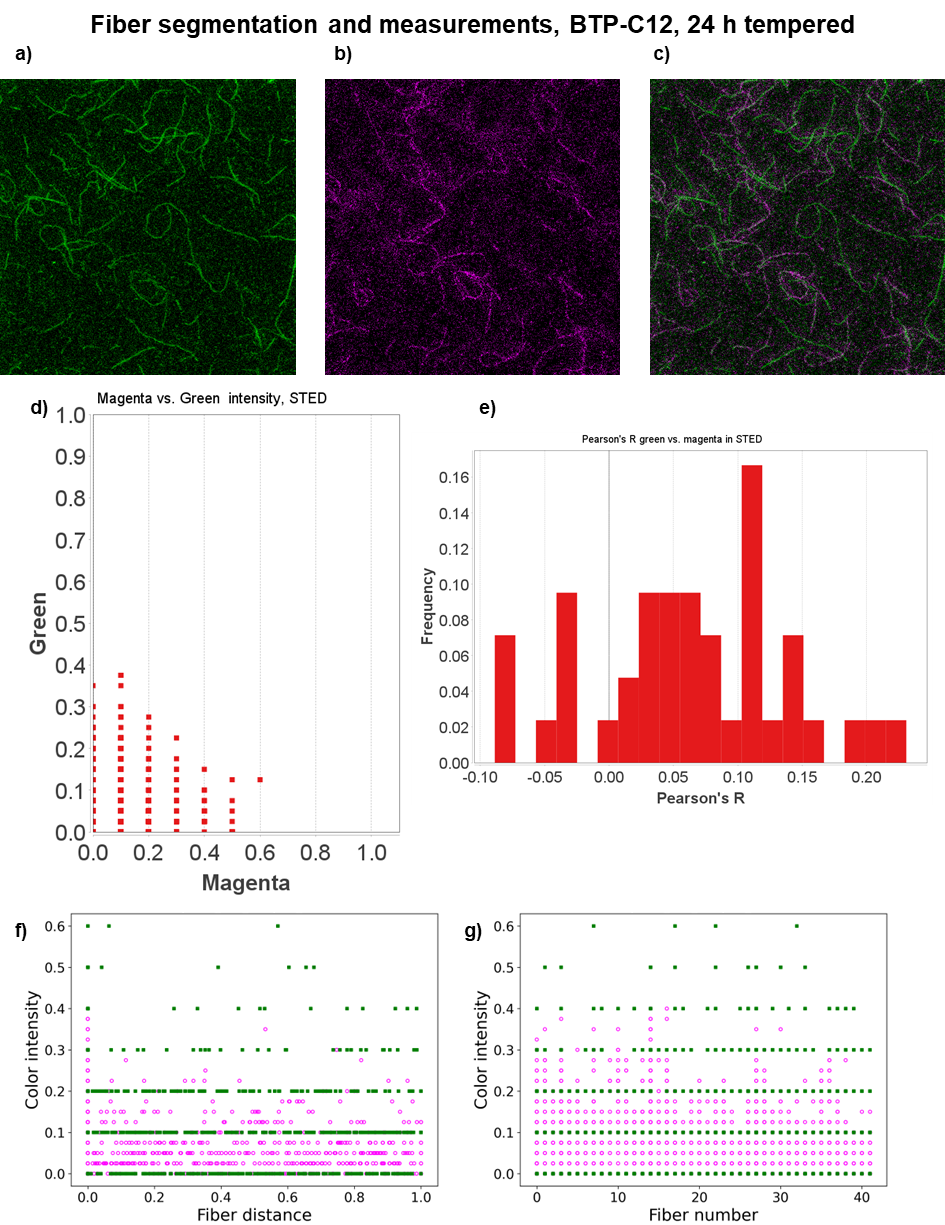
**

Supplementary Fig. 27. Representative results of the quantitative molecular measurements from the BTP-C12 image in the 24h tempered group. (a - g) As in Supplementary Fig. 23, for the 24h tempered group.

# References

[1] T. Klein, H. F. Ulrich, F. V. Gruschwitz, M. T. Kuchenbrod, R. Takahashi, S. Fujii, S. Hoeppener, I. Nischang, K. Sakurai, J. C. Brendel, *Impact of amino acids on the aqueous self-assembly of benzenetrispeptides into supramolecular polymer bottlebrushes.* *Polym. Chem.* **2020**, *11*, 6763-6771.

[2] F. V. Gruschwitz, M.-C. Fu, T. Klein, R. Takahashi, T. Higashihara, S. Hoeppener, I. Nischang, K. Sakurai, J. C. Brendel, *Unraveling Decisive Structural Parameters for the Self-Assembly of Supramolecular Polymer Bottlebrushes Based on Benzene Trisureas.* *Macromolecules* **2020**, *53*, 7552-7560.

[3] F. V. Gruschwitz, T. Klein, M. T. Kuchenbrod, N. Moriyama, S. Fujii, I. Nischang, S. Hoeppener, K. Sakurai, U. S. Schubert, J. C. Brendel, *Kinetically Controlling the Length of Self-Assembled Polymer Nanofibers Formed by Intermolecular Hydrogen Bonds.* *ACS Macro Lett.* **2021**, *10*, 837-843.

[4] Z. Zhao, D. Roy, J. Steinkühler, T. Robinson, R. Lipowsky, R. Dimova, *Super-Resolution Imaging of Highly Curved Membrane Structures in Giant Vesicles Encapsulating Molecular Condensates.* *Adv. Mater.* **2022**, *34*, 2106633.

[5] R. Gerst, Z. Cseresnyés, M. T. Figge, *JIPipe: visual batch processing for ImageJ.* *Nat Methods* **2023**, *20*, 168-169.

[6] C. A. Schneider, W. S. Rasband, K. W. Eliceiri, *NIH Image to ImageJ: 25 years of image analysis.* *Nature Methods* **2012**, *9*, 671-675.

[7] I. Zingman, D. Saupe, K. Lambers, *A morphological approach for distinguishing texture and individual features in images.* *Pattern Recognit. Lett.* **2014**, *47*, 129-138.

# Utilized nodes for the automated image analysis

| **Name** | **Description** | **Signature** |
| --- | --- | --- |
| **2D ROI calculator (AND/OR/XOR)** | Applies logical operations to the input ROI list. The logical operations are applied to the whole list, meaning that an AND operation will create the union of all ROI in the list. If you want to apply the operation only to a sub-set of ROI, preprocess using a ROI splitter algorithm. | (*ImageJ 2D ROI list* Input) -> (*ImageJ 2D ROI list* Output) |
| **2D ROI to Labels (by name)** | Converts ROI and an optional reference image into a label image. The label value is provided by mapping the name to a value. If no name mapping is provided for a ROI, a unique label is generated. If no reference image is provided, the dimensions are estimated from the ROI. The background color (where no ROI is located) is zero.  This algorithm groups the incoming data based on the annotations. Those groups can consist of one data item per slot. | (*ImageJ 2D ROI list* ROI, *ImageJ Image* Reference) -> (*ImageJ Image (float)* Labels) |
| **Add annotations as columns** | Adds column annotations to the table as new columns. | (*Results table* Input) -> (*Results table* Output) |
| **Add table column** | Adds a new column. By default no changes are applied if the column already exists. Can be optionally configured to replace existing columns. | (*Results table* Input) -> (*Results table* Output) |
| **Annotate data with table values** | Annotates the incoming data with values from the input table  This algorithm groups the incoming data based on the annotations. Those groups can consist of one data item per slot. | (*Data* Data, *Results table* Table) -> (*Data* Annotated data) |
| **Annotate with OME metadata** | Annotates an OME image with metadata extracted from the OME metadata | (*OME ImageJ Image* Input) -> (*OME ImageJ Image* Output) |
| **Annotate with image properties** | Adds data annotations that contain the image properties. | (*ImageJ Image* Image) -> (*ImageJ Image* Annotated image) |
| **Apply expression per row** | Applies an expression for each row. The column values are available as variables. | (*Results table* Input) -> (*Results table* Output) |
| **Auto threshold 2D** | Applies an auto-thresholding algorithm. If higher-dimensional data is provided, the filter is applied to each 2D slice.  This algorithm groups the incoming data based on the annotations. Those groups can consist of one data item per slot. | (*ImageJ Image (8 bit)* Input) -> (*ImageJ Image (mask)* Output) |
| **Binary skeleton to 2D filaments** | Applies a simple algorithm that converts a binary skeleton into a filament. This algorithm only supports 2D data and will apply the processing per Z/C/T slice. Please note that by default the Z voxel size is set to zero. | (*ImageJ Image* Skeleton) -> (*Filaments 3D* Filaments) |
| **Bio-Formats importer** | Imports images via the Bio-Formats plugin | (*File* Input) -> (*OME ImageJ Image* Output) |
| **Blend images** | Overlays greyscale or RGB images.  This algorithm groups the incoming data based on the annotations. Those groups can consist of one data item per slot. | (*ImageJ Image* Input) -> (*ImageJ Image (RGB)* Output) |
| **Cellpose prediction (2.x)** | Runs Cellpose on the input image with the given model(s). This node supports both segmentation in 3D and executing Cellpose for each 2D image plane. This node can generate a multitude of outputs, although only ROI is activated by default. Go to the 'Outputs' parameter section to enable the other outputs.   - **Labels:** A grayscale image where each connected component is assigned a unique value. - **Flows XY:** An RGB image that indicates the x and y flow of each pixel - **Flows Z:** Flows in Z direction (black for non-3D images) - **Flows d:** Multi-channel image that contains the flows [dZ, dY, dX, cell probability] (3D images) / [dY, dX, cell probability] (2D images) - **Probabilities:** An image indicating the cell probabilities for each pixel - **ROI:** ROI of the segmented areas.   Please note that you need to setup a valid Python environment with Cellpose installed. You can find the setting in Project > Application settings > Extensions > Cellpose.  This algorithm merges all annotations and data annotations. Use the data batch settings to determine how annotations and data annotations are merged | (*ImageJ Image* Input, *Cellpose model* Model) -> (*ImageJ Image (greyscale)* Labels, *ImageJ Image* Flows XY, *ImageJ Image* Flows Z, *ImageJ Image* Flows d, *ImageJ Image (float)* Probabilities, *ImageJ 2D ROI list* ROI) |
| **Coloc 2** | Colocalization analysis via the pixel intensity correlation over space methods of Pearson, Manders, Costes, Li and more, for scatterplots, analysis, automatic thresholding and statistical significance testing. Coloc 2 does NOT perform object based colocalization measurements, where objects are first segmented from the image, then their spatial relationships like overlap etc. are measured. This complementary approach is implemented in many ways elsewhere.  This algorithm groups the incoming data based on the annotations. Those groups can consist of one data item per slot. | (*ImageJ Image (greyscale)* Channel 1, *ImageJ Image (greyscale)* Channel 2, *ImageJ Image (mask)* Mask, *ImageJ 2D ROI list* ROI) -> (*Results table* Results, *ImageJ Image* Plots, *Results table* Histograms) |
| **Comment** | Allows you to comment an input or output slot. You can customize the color and icon of this node. This nodes has no workload attached to it and will connect to any data type. It requires no input and produces no output. | (*Data* Comment) -> (*Data* Comment) |
| **Compartment output** | Output of a compartment | () -> () |
| **Connect filament vertices (Expression)** | Connect existing vertices based on customizable criteria. Uses expressions to filter candidate edges, which can be slow.  This algorithm groups the incoming data based on the annotations. Those groups can consist of one data item per slot. | (*Filaments 3D* Input, *ImageJ Image (mask)* Mask) -> (*Filaments 3D* Output) |
| **Convert filaments to RGB** | Visualizes filaments by rendering them onto an RGB image  This algorithm groups the incoming data based on the annotations. Those groups can consist of one data item per slot. | (*Filaments 3D* Input, *ImageJ Image* Reference) -> (*ImageJ Image (RGB)* Output) |
| **Convert filaments to mask** | Converts filaments into a 3D ROI  This algorithm groups the incoming data based on the annotations. Those groups can consist of one data item per slot. | (*Filaments 3D* Input, *ImageJ Image* Reference) -> (*ImageJ Image (mask)* Output) |
| **Define multiple parameters** | Defines algorithm parameters that can be consumed by a multi-parameter algorithm | () -> (*Parameters* Parameters) |
| **Divide by maximum** | Divides greyscale pixel values by the global maximum across all slices. | (*ImageJ Image (float)* Input) -> (*ImageJ Image (float)* Output) |
| **Euclidean distance transform 2D** | Applies a euclidean distance transform on binary images.If higher-dimensional data is provided, the filter is applied to each 2D slice. | (*ImageJ Image (mask)* Input) -> (*ImageJ Image (float)* Output) |
| **Export image** | Exports incoming images into a non-JIPipe format (PNG, JPEG, BMP, AVI, TIFF). Please note support for input images depending on the file format:   - PNG, JPEG, BMP: 2D images only - AVI: 2D or 3D images only - TIFF: All images supported   This algorithm groups the incoming data based on the annotations. Those groups can consist of one data item per slot. | (*ImageJ Image* Input) -> (*File* Exported file) |
| **Export table** | Exports a results table to CSV/XLSX  This algorithm groups the incoming data based on the annotations. Those groups can consist of one data item per slot. | (*Results table* Input) -> (*File* Exported file) |
| **Fast image arithmetics** | Applies standard arithmetic and logical operations including, addition, subtraction, division, multiplication, GAMMA, EXP, LOG, SQR, SQRT, ABS, AND, OR, XOR, minimum, maximum, and more.  This algorithm groups the incoming data based on the annotations. Those groups can consist of one data item per slot. | () -> (*ImageJ Image (greyscale)* Output) |
| **Filter by annotation (If else)** | Filters data based on the annotation value. Has two outputs, one that contains the data that matches the filter, and another output that contains all data that does not match the filter | (*Data* Input) -> (*Data* Matched, *Data* Unmatched) |
| **Filter filament components** | Filters filament connected components by various properties | (*Filaments 3D* Input) -> (*Filaments 3D* Output) |
| **Filter filament vertices** | Filters filament vertices by various properties | (*Filaments 3D* Input) -> (*Filaments 3D* Output) |
| **Filter tables** | Filters tables by their properties. | (*Results table* Input) -> (*Results table* Output) |
| **Find linear filament vertex order/distance** | For all vertex components that are linear (all vertices have a degree less than 3), find the order each vertex in that line. Also can calculate the distance (in pixels) traveled from the start to the end point.The result is stored in each vertex' metadata field. | (*Filaments 3D* Input) -> (*Filaments 3D* Output) |
| **Find particles 2D** | Converts mask images into ROI by applying a connected components algorithm and generates measurements. Please note that this algorithm will always trace the external object boundaries and convert them into polygonal ROIs. This means that holes will be closed. This node requires a thresholded image as input and will extract measurements from the thresholded image. If you already have ROI available and want measurements, use 'Extract ROI statistics'.If higher-dimensional data is provided, the results are generated for each 2D slice. | (*ImageJ Image (mask)* Mask) -> (*ImageJ 2D ROI list* ROI, *Results table* Measurements) |
| **Gaussian blur 2D** | Applies convolution with a Gaussian function for smoothing. If higher-dimensional data is provided, the filter is applied to each 2D slice. | (*ImageJ Image* Input) -> (*ImageJ Image* Output) |
| **Group** | A node that contains a sub-pipeline. Double-click the node to open the contained workflow and add inputs/outputs to the 'Group input'/'Group output' nodes. To expose parameters from within the group to the surrounding group node, go to the 'Parameters' tab and add references via 'Edit parameter references'. | () -> () |
| **Group box** | A text box pre-configured to contain a title at the top left | () -> () |
| **Histogram plot** | Plots the incoming table(s). This node will create a Histogram plot. | (*Results table* Input) -> (*Plot* Output) |
| **Legacy image calculator 2D** | Applies a mathematical operation between two images. If higher-dimensional data is provided, the filter is applied to each 2D slice. Old implementation that directly uses the one provided by ImageJ. We recommend to use 'Fast image arithmetics' instead.  This algorithm groups the incoming data based on the annotations. Those groups can consist of one data item per slot. | (*ImageJ Image* Input 1, *ImageJ Image* Input 2) -> (*ImageJ Image* Output) |
| **Mask to 2D ROI** | Converts pixel values equal or higher than the given threshold to a ROI. This will create a single ROI that contains holes. If a higher-dimensional image is provided, the operation is applied for each slice. | (*ImageJ Image (mask)* Input) -> (*ImageJ 2D ROI list* Output) |
| **Measure filament components** | Measures all components in the filament graph. Produces the following measurements:   - number of vertices - number of edges - length (sum of edge lengths) - skeletonization-corrected length (adding 2 * radius for each vertex with degree 0 and 1 x radius for each vertex with degree 1) - confinement ratio (length of the simplified graph divided by the length) - number of vertices with specific degrees - min/max centroid x/y/z (per vertex) center[min/max][x/y/z] - min/max x/y/z (with sphere radius) sphere[min/max][x/y/z] - centroid x/y/z/c/t - min/max/avg radius/value   You can learn more about the generated measurements here: https://jipipe.hki-jena.de/documentation/filament-measurements.html | (*Filaments 3D* Input) -> (*Results table* Output) |
| **Measure filament vertices** | Stores all available information about the vertices into a table. You can learn more about the generated measurements here: https://jipipe.hki-jena.de/documentation/filament-measurements.html | (*Filaments 3D* Input) -> (*Results table* Output) |
| **Median filter 2D** | Applies a median filter. If a multi-channel image is provided, the operation is applied to each channel. If higher-dimensional data is provided, the filter is applied to each 2D slice. | (*ImageJ Image* Input) -> (*ImageJ Image* Output) |
| **Merge 2D ROI lists (ordered)** | Merges multiple ROI lists. The ROI from 'Source' are added to the end of the 'Target' list. Compared to 'Merge ROI lists', this node allows to control the order of the operation.  This algorithm groups the incoming data based on the annotations. Those groups can consist of multiple data items. If you want to group all data into one output, set the matching strategy to 'Custom' and leave 'Data set matching annotations' empty. | (*ImageJ 2D ROI list* Target, *ImageJ 2D ROI list* Source) -> (*ImageJ 2D ROI list* Output) |
| **Merge annotations** | Merges the annotations of all incoming data and outputs the same data with those merged annotations.  This algorithm groups the incoming data based on the annotations. Those groups can consist of one data item per slot. | () -> () |
| **Merge table rows** | Merges multiple tables into one table by merging the list of rows. Columns are automatically created if they do not exist.  This algorithm groups the incoming data based on the annotations. Those groups can consist of multiple data items. If you want to group all data into one output, set the matching strategy to 'Custom' and leave 'Data set matching annotations' empty. | (*Results table* Input) -> (*Results table* Output) |
| **Morphological Feature Contrast (MFC) 2D** | Calculates the Morphological Feature Contrast (MFC) of the image. For upper MFC, applies the following transformation to the input image f: MFC+ = MAX(0, f - Opening(r2, Closing(r1, f))). For lower MFC, applies the following transformation to the input image f: MFC- = MAX(0, Closing(r2, Opening(r1, f)) - f). For the full MFC, applies the following transformation to the input image f: MFC = MFC+ + MFC-. If higher-dimensional data is provided, the filter is applied to each 2D slice. | (*ImageJ Image (greyscale)* Input) -> (*ImageJ Image (greyscale)* Input) |
| **Morphological operation 2D** | Applies a morphological operation to greyscale images. Following operations are included: Erosion, Dilation, Opening, Closing, White Top Hat, Black Top Hat, Gradient, Laplacian, Internal Gradient, and External Gradient. More information (including examples) can be found at https://imagej.net/MorphoLibJ.html If a multi-channel image is provided, the operation is applied to each channel. If higher-dimensional data is provided, the filter is applied to each 2D slice. | (*ImageJ Image* Input) -> (*ImageJ Image* Output) |
| **Morphological skeletonize 2D** | Applies a morphological skeletonization operation to binary images.If higher-dimensional data is provided, the filter is applied to each 2D slice. | (*ImageJ Image (mask)* Input) -> (*ImageJ Image (mask)* Output) |
| **Pretrained Cellpose 2.x model** | Imports one or a selection of pretrained Cellpose 2.x models | () -> (*Cellpose model* Output) |
| **Project user directory** | Returns a project user (Project > Project settings > User directories) folder as data | () -> (*Folder* Output) |
| **Python script (iterating)** | Runs a Python script that iterates through each data batch in the input slots. This node uses an existing dedicated Python interpreter that must be set up in the application settings.  To learn more about the JIPipe Python API, visit https://jipipe.hki-jena.de/apidocs/python-current/index.html  This algorithm groups the incoming data based on the annotations. Those groups can consist of one data item per slot. | () -> () |
| **Set LUT (color map)** | Sets the LUT of the image from a predefined color map. This does not change the pixel data. | (*ImageJ Image (greyscale)* Input) -> (*ImageJ Image (greyscale)* Output) |
| **Set LUT (two colors)** | Generates a LUT from the first to the second color. This does not change the pixel data. | (*ImageJ Image (greyscale)* Input) -> (*ImageJ Image (greyscale)* Output) |
| **Set annotation (expression)** | Sets a single annotation. The name and the value are determined by expressions | (*Data* Input) -> (*Data* Output) |
| **Set filament vertex metadata to component index** | Finds all connected components and writes the component index into a metadata field | (*Filaments 3D* Input) -> (*Filaments 3D* Output) |
| **Set filament vertex radius from image** | Sets the radius of each vertex from the given input image. Please note that if the C/T coordinates are set to zero, the value is extracted from the 0/0 slice.  This algorithm groups the incoming data based on the annotations. Those groups can consist of one data item per slot. | (*Filaments 3D* Filaments, *ImageJ Image (greyscale)* Radius) -> (*Filaments 3D* Output) |
| **Set filament vertex value from image** | Sets the value/intensity of each vertex from the given input image. Please note that if the C/T coordinates are set to zero, the value is extracted from the 0/0 slice.  This algorithm groups the incoming data based on the annotations. Those groups can consist of one data item per slot. | (*Filaments 3D* Filaments, *ImageJ Image (greyscale)* Intensity) -> (*Filaments 3D* Output) |
| **Set to value (grayscale)** | Sets all pixels of the input image to the specified grayscale value. If the image is RGB, the value is converted into an integer that is parsed as RGB.  This algorithm groups the incoming data based on the annotations. Those groups can consist of one data item per slot. | (*ImageJ Image* Input) -> (*ImageJ Image* Output) |
| **Set/Edit annotations** | Modifies the specified annotations to the specified values. Supports expressions to combine existing annotations or generate new values. | (*Data* Input) -> (*Data* Output) |
| **Smooth filaments** | Applies a smoothing operation that is based around downscaling the locations and applying the 'Remove duplicate vertices' operation. The positions are then restored. | (*Filaments 3D* Input) -> (*Filaments 3D* Output) |
| **Sort table rows** | Sorts the table rows by columns | (*Results table* Input) -> (*Results table* Output) |
| **Split & filter by annotation** | Splits the input data by a specified annotation or filters data based on the annotation value. | (*Data* Input) -> () |
| **Split filaments into connected components** | Splits the filament graph into connected components and outputs one graph per component | (*Filaments 3D* Input) -> (*Filaments 3D* Output) |
| **Summarize table** | Summarize table columns by applying predefined operations like average, standard deviation, or median. | (*Results table* Input) -> (*Results table* Output) |
| **Text box** | An annotation that contains text | () -> () |
| **XY scatter plot** | Plots the incoming table(s). This node will create a XY scatter plot. | (*Results table* Input) -> (*Plot* Output) |

# Dependencies

## JIPipe extensions

| **Name** | **Version** | **Author(s)** |
| --- | --- | --- |
| **Cellpose integration** | 4.2.0-SNAPSHOT | Zoltán Cseresnyés*, Ruman Gerst*, Marc Thilo Figge# |
| **Standard plots** | 4.2.0-SNAPSHOT | Zoltán Cseresnyés*, Ruman Gerst*, Marc Thilo Figge# |
| **Python integration** | 4.2.0-SNAPSHOT | Zoltán Cseresnyés*, Ruman Gerst*, Marc Thilo Figge# |
| **Multi parameters data types** | 4.2.0-SNAPSHOT | Zoltán Cseresnyés*, Ruman Gerst*, Marc Thilo Figge# |
| **Graph annotations** | 4.2.0-SNAPSHOT | Zoltán Cseresnyés*, Ruman Gerst*, Marc Thilo Figge# |
| **ImageJ integration** | 4.2.0-SNAPSHOT | Zoltán Cseresnyés*, Ruman Gerst*, Marc Thilo Figge# |
| **Compartment management** | 4.2.0-SNAPSHOT | Zoltán Cseresnyés*, Ruman Gerst*, Marc Thilo Figge# |
| **Multi parameter algorithms** | 4.2.0-SNAPSHOT | Zoltán Cseresnyés*, Ruman Gerst*, Marc Thilo Figge# |
| **Utilities** | 4.2.0-SNAPSHOT | Zoltán Cseresnyés*, Ruman Gerst*, Marc Thilo Figge# |
| **3D Scenes** | 4.2.0-SNAPSHOT | Zoltán Cseresnyés*, Ruman Gerst*, Marc Thilo Figge# |
| **Standard table operations** | 4.2.0-SNAPSHOT | Zoltán Cseresnyés*, Ruman Gerst*, Marc Thilo Figge# |
| **3D ImageJ Suite integration** | 4.2.0-SNAPSHOT | Zoltán Cseresnyés*, Ruman Gerst*, Marc Thilo Figge# |
| **Core** | 4.2.0-SNAPSHOT | Zoltán Cseresnyés*, Ruman Gerst*, Marc Thilo Figge# |
| **Node grouping** | 4.2.0-SNAPSHOT | Zoltán Cseresnyés*, Ruman Gerst*, Marc Thilo Figge# |
| **Annotation data types and algorithms** | 4.2.0-SNAPSHOT | Zoltán Cseresnyés*, Ruman Gerst*, Marc Thilo Figge# |
| **Strings** | 4.2.0-SNAPSHOT | Zoltán Cseresnyés*, Ruman Gerst*, Marc Thilo Figge# |
| **Filaments** | 4.2.0-SNAPSHOT | Zoltán Cseresnyés*, Ruman Gerst*, Marc Thilo Figge# |
| **Filesystem types and algorithms** | 4.2.0-SNAPSHOT | Zoltán Cseresnyés*, Ruman Gerst*, Marc Thilo Figge# |
| **ImageJ algorithms** | 4.2.0-SNAPSHOT | Zoltán Cseresnyés*, Ruman Gerst*, Marc Thilo Figge# |

## ImageJ update sites

| **Name** | **URL** |
| --- | --- |
| **3D ImageJ Suite** | https://sites.imagej.net/Tboudier/ |
| **IJPB-plugins** | https://sites.imagej.net/IJPB-plugins/ |
| **ImageScience** | https://sites.imagej.net/ImageScience/ |

## External environments

| **Type** | **Name** | **Version** | **Source/URL** |
| --- | --- | --- | --- |
| JIPipePythonAdapterLibraryEnvironment |  | unknown | NA |
| PythonEnvironment |  | unknown | NA |
| PythonEnvironment | **Artifact** | unknown | org.python.python_prepackaged:3.10.15.1000-windows_amd64 |

## Citations (dependencies)

- J. Ollion, J. Cochennec, F. Loll, C. Escudé, T. Boudier. (2013) TANGO: A Generic Tool for High-throughput 3D Image Analysis for Studying Nuclear Organization. Bioinformatics 2013 Jul 15;29(14):1840-1.
- ImageScience by Erik Meijering. https://imagescience.org/meijering/software/imagescience/
- Papirus Icon Theme: https://github.com/PapirusDevelopmentTeam/papirus-icon-theme (Licensed under GPL-3)
- See https://imagej.net/plugins/coloc-2
- Melissa Linkert, Curtis T. Rueden, Chris Allan, Jean-Marie Burel, Will Moore, Andrew Patterson, Brian Loranger, Josh Moore, Carlos Neves, Donald MacDonald, Aleksandra Tarkowska, Caitlin Sticco, Emma Hill, Mike Rossner, Kevin W. Eliceiri, and Jason R. Swedlow (2010) Metadata matters: access to image data in the real world. The Journal of Cell Biology 189(5), 777-782
- Rueden, C., Schindelin, J., Hiner, M. & Eliceiri, K. (2016). SciJava Common [Software]. https://scijava.org/.
- Stringer, C., Wang, T., Michaelos, M., & Pachitariu, M. (2021). Cellpose: a generalist algorithm for cellular segmentation. Nature Methods, 18(1), 100-106.
- RandomJ by Erik Meijering. https://imagescience.org/meijering/software/randomj/
- Schneider, C. A.; Rasband, W. S. & Eliceiri, K. W. (2012), "NIH Image to ImageJ: 25 years of image analysis", Nature methods 9(7): 671-675
- https://jipipe.hki-jena.de/documentation/filament-measurements.html
- Gerst, R., Cseresnyés, Z. & Figge, M.T. JIPipe: visual batch processing for ImageJ. Nat Methods (2023). https://doi.org/10.1038/s41592-022-01744-4
- Fluent icon theme: https://github.com/vinceliuice/Fluent-icon-theme (Licensed under GPL-3)
- Rueden, C. T.; Schindelin, J. & Hiner, M. C. et al. (2017), "ImageJ2: ImageJ for the next generation of scientific image data", BMC Bioinformatics 18:529
- Eliceiri K. V., Berthold M. R., Goldberg I. G., Ibanez L., Manjunath B. S., Martone M. E., Murphy R. F., Peng H., Plant A. L., Roysam B., Stuurmann N., Swedlow J.R., Tomancak P., Carpenter A. E. (2012) Biological Imaging Software Tools Nature Methods 9(7), 697-710
- OrientationJ by Daniel Sage at the Biomedical Image Group (BIG), EPFL, Switzerland. https://bigwww.epfl.ch/demo/orientation/
- Zingman, I., Saupe, D., & Lambers, K. (2014). A morphological approach for distinguishing texture and individual features in images. Pattern Recognition Letters, 47, 129-138.
- Steger, C., 1998. An unbiased detector of curvilinear structures. IEEE Transactions on Pattern Analysis and Machine Intelligence, 20(2), pp.113–125.
- Font Awesome Free 5.12.1 (desktop): https://fontawesome.com/ (Licensed under Font Awesome Free License)
- Legland, D.; Arganda-Carreras, I. & Andrey, P. (2016), "MorphoLibJ: integrated library and plugins for mathematical morphology with ImageJ", Bioinformatics (Oxford Univ Press) 32(22): 3532-3534, PMID 27412086, doi:10.1093/bioinformatics/btw413
- Breeze Icons: https://github.com/KDE/breeze-icons (Licensed under LGPL-2.1)
- MTrackJ by Erik Meijering. https://imagescience.org/meijering/software/mtrackj/
- Font Awesome Free 6.5.1 (desktop): https://fontawesome.com/ (Licensed under Font Awesome Free License)
- Schindelin, J.; Arganda-Carreras, I. & Frise, E. et al. (2012), "Fiji: an open-source platform for biological-image analysis", Nature methods 9(7): 676-682, PMID 22743772, doi:10.1038/nmeth.2019
- FeatureJ by Erik Meijering. http://imagescience.org/meijering/software/featurej/
- See https://imagej.net/imaging/colocalization-analysis for more information abot colocalization

# Pipeline text description

## Compartment C1 "Preprocessing"

**Node #1 "Project user directory" of type "Project user directory"**

- The parameter "Project user directory key" (key) is set to **"Datafolder"**

**Node #2 "Set single annotation: modality" of type "Set annotation (expression)"**

- The parameter "Annotation name" (annotation-name) is set to **#Modality**
- The parameter "Annotation value" (annotation-value) is set to **IF_ELSE(Series == "1" OR Series == "3", "Conf", "STED")**

**Node #3 "READ: folders, subfolders and files" of type "Group"**

- Input "Input folder" of node #3 receives data from output "Output" of node #1
- This node contains a sub-graph. The "Group Input" and "Group Output" nodes contained in the graph are internally connected to the inputs and outputs of this node.

**Node #4 "Group input" of type "Group input"**

**Node #5 "Add path to annotations" of type "Add path to annotations"**

- Input "Paths" of node #5 receives data from output "Input folder" of node #4
- The parameter "Remove file extensions" (remove-extensions) is set to **false**
- The parameter "Generated annotation" (generated-annotation) is set to **"#Folder"**

**Node #6 "List subfolders" of type "List subfolders"**

- Input "Folders" of node #6 receives data from output "Annotated paths" of node #5
- The parameter "Recursive search follows links" (recursive-follows-links) is set to **false**

**Node #7 "Add path to annotations" of type "Add path to annotations"**

- Input "Paths" of node #7 receives data from output "Subfolders" of node #6
- The parameter "Remove file extensions" (remove-extensions) is set to **false**
- The parameter "Generated annotation" (generated-annotation) is set to **"#Subfolder"**

**Node #8 "Add path to annotations" of type "Add path to annotations"**

- Input "Paths" of node #8 receives data from output "Annotated paths" of node #7
- The parameter "Remove file extensions" (remove-extensions) is set to **false**
- The parameter "Annotate with full path" (full-path) is set to **true**
- The parameter "Generated annotation" (generated-annotation) is set to **"#Fullpath"**

**Node #9 "List files" of type "List files"**

- Input "Folders" of node #9 receives data from output "Annotated paths" of node #8
- The parameter "Recursive search follows links" (recursive-follows-links) is set to **false**
- The parameter "Recursive" (recursive) is set to **true**

**Node #10 "Add path to annotations" of type "Add path to annotations"**

- Input "Paths" of node #10 receives data from output "Files" of node #9
- The parameter "Remove file extensions" (remove-extensions) is set to **false**
- The parameter "Generated annotation" (generated-annotation) is set to **"#Image"**

**Node #11 "Filter paths" of type "Filter paths"**

- Input "Input" of node #11 receives data from output "Annotated paths" of node #10
- The parameter "Keep path if ..." (filters) is set to **".msr" IN name**

**Node #12 "Group output" of type "Group output"**

- Input "Annotated paths" of node #12 receives data from output "Output" of node #11

**Node #13 "Set single annotation: color" of type "Set annotation (expression)"**

- Input "Input" of node #13 receives data from output "Output" of node #2
- The parameter "Annotation name" (annotation-name) is set to **#Label**
- The parameter "Annotation value" (annotation-value) is set to **IF_ELSE(Series == "1" OR Series == "2", 488 , 594)**

**Node #14 "Bio-Formats importer" of type "Bio-Formats importer"**

- Input "Input" of node #14 receives data from output "Annotated paths" of node #3
- The parameter "Annotate with title" (title-annotation) is set to **"Image title"**
- The parameter item #1 of "Series to import" (series-to-import) is set to **0**
- The parameter item #2 of "Series to import" (series-to-import) is set to **1**
- The parameter item #3 of "Series to import" (series-to-import) is set to **2**
- The parameter item #4 of "Series to import" (series-to-import) is set to **3**
- The parameter "Extract 2D ROI" (extract-rois) is set to **false**

**Node #15 "Group: Get OME XML metadata as annotations" of type "Group"**

- Input "Input" of node #15 receives data from output "Output" of node #14
- This node contains a sub-graph. The "Group Input" and "Group Output" nodes contained in the graph are internally connected to the inputs and outputs of this node.

**Node #16 "Group input" of type "Group input"**

**Node #17 "Extract OME XML" of type "Extract OME XML"**

- Input "Input" of node #17 receives data from output "Input" of node #16

**Node #18 "Prettify XML" of type "Prettify XML"**

- Input "Input" of node #18 receives data from output "Output" of node #17

**Node #19 "Annotate with XML values" of type "Annotate with XML values"**

- Input "Input" of node #19 receives data from output "Output" of node #18
- The parameter item #1 of "Generated annotations" (entries) is set to **xpath = "/OME/Image/AcquisitionDate", annotation-name = "AcquisitionDate"**

**Node #20 "Group output" of type "Group output"**

- Input "Output" of node #20 receives data from output "Output" of node #19

**Node #21 "Annotate with image properties" of type "Annotate with image properties"**

- Input "Image" of node #21 receives data from output "Output" of node #14
- The parameter "Annotate with image height" (height-annotation) is set to **"Image height"**
- The parameter "Annotate with image width" (width-annotation) is set to **"Image width"**

**Node #22 "Set single annotation" of type "Set annotation (expression)"**

- Input "Input" of node #22 receives data from output "Output" of node #15
- The parameter "Annotation name" (annotation-name) is set to **"AcquisitionDate"**
- The parameter "Annotation value" (annotation-value) is set to **SUBSTRING(AcquisitionDate, STRING_FIRST_INDEX_OF(AcquisitionDate, ">")+1, STRING_FIRST_INDEX_OF(AcquisitionDate, "</"))**

**Node #23 "Set single annotation: label 1" of type "Set annotation (expression)"**

- Input "Input" of node #23 receives data from output "Annotated image" of node #21
- The parameter "Annotation name" (annotation-name) is set to **Label1**
- The parameter "Pass through" (jipipe:algorithm:pass-through) is set to **true**
- The parameter "Annotation value" (annotation-value) is set to **SPLIT_STRING( REPLACE_IN_STRING(#Image, " ", "-"), "_")[0]**

**Node #24 "Set single annotation" of type "Set annotation (expression)"**

- Input "Input" of node #24 receives data from output "Output" of node #22
- The parameter "Annotation name" (annotation-name) is set to **Color**
- The parameter "Annotation value" (annotation-value) is set to **SUBSTRING($"Image title", STRING_FIRST_INDEX_OF($"Image title", "STAR ")+5, STRING_FIRST_INDEX_OF($"Image title", "_"))**

**Node #25 "Set single annotation: color 1" of type "Set annotation (expression)"**

- Input "Input" of node #25 receives data from output "Output" of node #23
- The parameter "Annotation name" (annotation-name) is set to **Color1**
- The parameter "Pass through" (jipipe:algorithm:pass-through) is set to **true**
- The parameter "Annotation value" (annotation-value) is set to **SPLIT_STRING( REPLACE_IN_STRING(#Image, " ", "-"), "_")[2]**

**Node #26 "Set single annotation" of type "Set annotation (expression)"**

- Input "Input" of node #26 receives data from output "Output" of node #24
- The parameter "Annotation name" (annotation-name) is set to **Modality**
- The parameter "Annotation value" (annotation-value) is set to **SUBSTRING($"Image title", STRING_FIRST_INDEX_OF($"Image title", "_")+1, STRING_FIRST_INDEX_OF($"Image title", "{"))**

**Node #27 "Set single annotation: label 2" of type "Set annotation (expression)"**

- Input "Input" of node #27 receives data from output "Output" of node #25
- The parameter "Annotation name" (annotation-name) is set to **Label2**
- The parameter "Pass through" (jipipe:algorithm:pass-through) is set to **true**
- The parameter "Annotation value" (annotation-value) is set to **SPLIT_STRING( REPLACE_IN_STRING(#Image, " ", "-"), "_")[2]**

**Node #28 "Merge annotations" of type "Merge annotations"**

- Input "Data 1" of node #28 receives data from output "Output" of node #26
- Input "Data 2" of node #28 receives data from output "Output" of node #14
- The parameter "Grouping method" in category "Input management" (jipipe:data-batch-generation/column-matching) is set to **"Custom"**
- The parameter "Merge same data annotation values" in category "Input management" (jipipe:data-batch-generation/data-annotation-merge-strategy) is set to **"Merge"**
- The parameter "Custom grouping columns" in category "Input management" (jipipe:data-batch-generation/custom-matched-columns-expression) is set to **$"Image title"**

**Node #29 "Set single annotation: color 2" of type "Set annotation (expression)"**

- Input "Input" of node #29 receives data from output "Output" of node #27
- The parameter "Annotation name" (annotation-name) is set to **Color2**
- The parameter "Pass through" (jipipe:algorithm:pass-through) is set to **true**
- The parameter "Annotation value" (annotation-value) is set to **SPLIT_STRING( REPLACE_IN_STRING(#Image, " ", "-"), "_")[3]**

**Node #30 "Annotate with OME metadata: acquisition date" of type "Annotate with OME metadata"**

- Input "Input" of node #30 receives data from output "Output" of node #29
- The parameter "Pass through" (jipipe:algorithm:pass-through) is set to **true**
- The parameter item #1 of "Generated annotations" (entries) is set to **annotation-name = "AcquisitionDate", accessor = {"accessor-id":"getImageAcquisitionDate","parameters":{"parameters":{"param-0-image-index":{"key":"param-0-image-index","name":"Image Index","description":"the Image index.","hidden":false,"field-class":"int","value":0,"short-key":"param-0-image-index","ui-order":0,"field-class-id":"primitive.int"}}}}**

**Node #31 "Set single annotation: acquisition date" of type "Set annotation (expression)"**

- Input "Input" of node #31 receives data from output "Output" of node #30
- The parameter "Annotation name" (annotation-name) is set to **"AcquisitionDate"**
- The parameter "Pass through" (jipipe:algorithm:pass-through) is set to **true**
- The parameter "Annotation value" (annotation-value) is set to **SUBSTRING(AcquisitionDate, 0, STRING_FIRST_INDEX_OF(AcquisitionDate, "T"))**

**Node #32 "Set single annotation: color" of type "Set annotation (expression)"**

- Input "Input" of node #32 receives data from output "Output" of node #31
- The parameter "Annotation name" (annotation-name) is set to **Color**
- The parameter "Pass through" (jipipe:algorithm:pass-through) is set to **true**
- The parameter "Annotation value" (annotation-value) is set to **SUBSTRING($"Image title", STRING_FIRST_INDEX_OF($"Image title", "STAR ") + 5, STRING_LAST_INDEX_OF($"Image title", "_"))**

**Node #33 "Set single annotation: modality and color" of type "Set annotation (expression)"**

- Input "Input" of node #33 receives data from output "Output" of node #32
- The parameter "Annotation name" (annotation-name) is set to **ModalityAndColor**
- The parameter "Annotation value" (annotation-value) is set to **SUBSTRING($"Image title", STRING_LAST_INDEX_OF($"Image title", "_")+1, STRING_FIRST_INDEX_OF($"Image title", "{")-1)**

**Node #34 "Set single annotation: modality" of type "Set annotation (expression)"**

- Input "Input" of node #34 receives data from output "Output" of node #33
- The parameter "Annotation name" (annotation-name) is set to **#Modality**
- The parameter "Annotation value" (annotation-value) is set to **SUBSTRING(ModalityAndColor, STRING_LAST_INDEX_OF(ModalityAndColor, "-")+1)**

**Node #35 "Set single annotation: color" of type "Set annotation (expression)"**

- Input "Input" of node #35 receives data from output "Output" of node #34
- The parameter "Annotation name" (annotation-name) is set to **#Label**
- The parameter "Annotation value" (annotation-value) is set to **SUBSTRING( SUBSTRING_BEFORE(ModalityAndColor, STRING_LAST_INDEX_OF(ModalityAndColor, #Modality)-1), STRING_LAST_INDEX_OF( SUBSTRING_BEFORE(ModalityAndColor, STRING_LAST_INDEX_OF(ModalityAndColor, #Modality)-1), "-")+1)**

**Node #36 "Set/Edit annotations: redefine "#Image"" of type "Set/Edit annotations"**

- Input "Input" of node #36 receives data from output "Output" of node #35
- The parameter item #1 of "Annotations" (generated-annotation) is set to **(#Folder + "__" + #Subfolder + "__" + #Image, "#Image")**

**Node #37 "Output" of type "Compartment output"**

- Input "Annoated images" of node #37 receives data from output "Output" of node #36
- The parameter "jipipe:compartment:output-slot-name" (jipipe:compartment:output-slot-name) is set to **"Output"**

## Compartment C2 "Segmentation and colocalization"

- The "Segmentation and colocalization" compartment (C2) receives data from the "Preprocessing" compartment (C1)

**Node #38 "Multiple parameters: global threshold" of type "Define multiple parameters"**

- The parameter "Parameters" (parameter-table) is set to **{"columns":[{"name":"Method","description":null,"key":"method","field-class":"ij.process.AutoThresholder$Method","field-class-id":"ij.process.AutoThresholder.Method"},{"name":"Dark background","description":null,"key":"dark-background","field-class":"boolean","field-class-id":"primitive.boolean"}],"rows":[{"method":"Default","dark-background":true},{"method":"Huang","dark-background":true},{"method":"Li","dark-background":true},{"method":"MaxEntropy","dark-background":true},{"method":"Otsu","dark-background":true},{"method":"RenyiEntropy","dark-background":true},{"method":"Shanbhag","dark-background":true},{"method":"Triangle","dark-background":true},{"method":"Yen","dark-background":true},{"method":"IJ_IsoData","dark-background":true},{"method":"Intermodes","dark-background":true},{"method":"IsoData","dark-background":true},{"method":"Mean","dark-background":true},{"method":"MinError","dark-background":true},{"method":"Minimum","dark-background":true},{"method":"Moments","dark-background":true},{"method":"Percentile","dark-background":true}]}**

**Node #39 "Group: merging and multiplying multiple parameters" of type "Group"**

- This node contains a sub-graph. The "Group Input" and "Group Output" nodes contained in the graph are internally connected to the inputs and outputs of this node.

**Node #40 "Define multiple parameters" of type "Define multiple parameters"**

- The parameter "Parameters" (parameter-table) is set to **{"columns":[{"name":"Radius 2","description":"The second radius. Zingman et al suggest that the second radius should be chosen according to the following constraints: <code>maxFeatureSize &lt; r1 &lt; minTextureRegionsSize</code>, where maxFeatureSize is the maximum size of features and minTextureRegionsSize is the minimum size of texture regions.","key":"radius2","field-class":"int","field-class-id":"primitive.int"}],"rows":[{"radius2":1},{"radius2":2},{"radius2":3},{"radius2":5},{"radius2":7},{"radius2":10}]}**

**Node #41 "Define multiple parameters" of type "Define multiple parameters"**

- The parameter "Parameters" (parameter-table) is set to **{"columns":[{"name":"Radius 1","description":"The first radius. Zingman et al suggest that the first radius should be chosen according to the following constraints: <code>maxDistDetails &lt; r1 &lt; minDistIsolatedFeatures</code>, where maxDistDetails is the maximum distance between texture details and minDistIsolatedFeatures is the minimum distance to isolated features.","key":"radius1","field-class":"int","field-class-id":"primitive.int"}],"rows":[{"radius1":1},{"radius1":2},{"radius1":3},{"radius1":5},{"radius1":7},{"radius1":10}]}**

**Node #42 "Define multiple parameters" of type "Define multiple parameters"**

- The parameter "Parameters" (parameter-table) is set to **{"columns":[{"name":"Structure element","description":"The shape of the structure element (default is square)","key":"structure-element","field-class":"inra.ijpb.morphology.Strel$Shape","field-class-id":"ij1-morph:strel"}],"rows":[{"structure-element":"SQUARE"},{"structure-element":"DIAMOND"},{"structure-element":"DISK"},{"structure-element":"OCTAGON"},{"structure-element":"SQUARE"},{"structure-element":"SQUARE"}]}**

**Node #43 "Merge parameters" of type "Merge parameters"**

- Input "Radius 1" of node #43 receives data from output "Parameters" of node #41
- Input "Radius 2" of node #43 receives data from output "Parameters" of node #40
- Input "Structure" of node #43 receives data from output "Parameters" of node #42
- The parameter "Grouping method" in category "Input management" (jipipe:data-batch-generation/column-matching) is set to **"None"**

**Node #44 "Group output" of type "Group output"**

- Input "Output" of node #44 receives data from output "Output" of node #43

**Node #45 "Group input" of type "Group input"**

**Node #46 "Pretrained Cellpose 2.x model" of type "Pretrained Cellpose 2.x model"**

**Node #47 "Pretrained Cellpose 2.x model" of type "Pretrained Cellpose 2.x model"**

**Node #48 "Pretrained Cellpose 2.x model" of type "Pretrained Cellpose 2.x model"**

**Node #49 "Pretrained Cellpose 2.x model" of type "Pretrained Cellpose 2.x model"**

**Node #37 "Output" of type "Compartment output"**

- Input "Annoated images" of node #37 receives data from output "Output" of node #36
- The parameter "jipipe:compartment:output-slot-name" (jipipe:compartment:output-slot-name) is set to **"Output"**

**Node #50 "Split & filter by annotation" of type "Split & filter by annotation"**

- Input "Input" of node #50 receives data from output "Annoated images" of node #37
- The parameter "Output" in category "Filters" (target-slots/Output) is set to **#Label CONTAINS "488"**
- The parameter "Output 1" in category "Filters" (target-slots/Output 1) is set to **#Label CONTAINS "594"**

**Node #51 "Filter by annotation (If else): green" of type "Filter by annotation (If else)"**

- Input "Input" of node #51 receives data from output "Output" of node #50
- The parameter "Filter" (filter) is set to **#Modality == "Conf"**

**Node #52 "Filter by annotation (If else): magenta" of type "Filter by annotation (If else)"**

- Input "Input" of node #52 receives data from output "Output 1" of node #50
- The parameter "Filter" (filter) is set to **#Modality == "Conf"**

**Node #53 "Divide by maximum" of type "Divide by maximum"**

- Input "Input" of node #53 receives data from output "Matched" of node #51

**Node #54 "Divide by maximum" of type "Divide by maximum"**

- Input "Input" of node #54 receives data from output "Unmatched" of node #51

**Node #55 "Divide by maximum" of type "Divide by maximum"**

- Input "Input" of node #55 receives data from output "Matched" of node #52

**Node #56 "Divide by maximum" of type "Divide by maximum"**

- Input "Input" of node #56 receives data from output "Unmatched" of node #52

**Node #57 "Cellpose prediction (2.x)" of type "Cellpose prediction (2.x)"**

- Input "Input" of node #57 receives data from output "Output" of node #53
- Input "Model" of node #57 receives data from output "Output" of node #46
- The parameter "Override Python environment" (override-environment) is set to **[Disabled]**
- The parameter "Output probabilities" in category "Cellpose: Outputs" (output-parameters/output-probabilities) is set to **true**
- The parameter "Output flows XY" in category "Cellpose: Outputs" (output-parameters/output-flows-xy) is set to **true**
- The parameter "Cell probability threshold" in category "Cellpose: Thresholds" (threshold-parameters/cell-probability-threshold) is set to **-2.0**
- The parameter "Flow threshold (2D)" in category "Cellpose: Thresholds" (threshold-parameters/flow-threshold) is set to **0.0**

**Node #58 "Cellpose prediction (2.x)" of type "Cellpose prediction (2.x)"**

- Input "Input" of node #58 receives data from output "Output" of node #54
- Input "Model" of node #58 receives data from output "Output" of node #47
- The parameter "Override Python environment" (override-environment) is set to **[Disabled]**
- The parameter "Output probabilities" in category "Cellpose: Outputs" (output-parameters/output-probabilities) is set to **true**
- The parameter "Output flows XY" in category "Cellpose: Outputs" (output-parameters/output-flows-xy) is set to **true**
- The parameter "Cell probability threshold" in category "Cellpose: Thresholds" (threshold-parameters/cell-probability-threshold) is set to **-2.0**
- The parameter "Flow threshold (2D)" in category "Cellpose: Thresholds" (threshold-parameters/flow-threshold) is set to **0.0**

**Node #59 "Cellpose prediction (2.x)" of type "Cellpose prediction (2.x)"**

- Input "Input" of node #59 receives data from output "Output" of node #55
- Input "Model" of node #59 receives data from output "Output" of node #49
- The parameter "Override Python environment" (override-environment) is set to **[Disabled]**
- The parameter "Output probabilities" in category "Cellpose: Outputs" (output-parameters/output-probabilities) is set to **true**
- The parameter "Output flows XY" in category "Cellpose: Outputs" (output-parameters/output-flows-xy) is set to **true**
- The parameter "Cell probability threshold" in category "Cellpose: Thresholds" (threshold-parameters/cell-probability-threshold) is set to **-2.0**
- The parameter "Flow threshold (2D)" in category "Cellpose: Thresholds" (threshold-parameters/flow-threshold) is set to **0.0**

**Node #60 "Fast image arithmetics" of type "Fast image arithmetics"**

- Input "I1" of node #60 receives data from output "Output" of node #53
- Input "I2" of node #60 receives data from output "Output" of node #55
- The parameter "Grouping method" in category "Input management" (jipipe:data-batch-generation/column-matching) is set to **"Custom"**
- The parameter "Custom grouping columns" in category "Input management" (jipipe:data-batch-generation/custom-matched-columns-expression) is set to **#Image**

**Node #61 "Fast image arithmetics" of type "Fast image arithmetics"**

- Input "I1" of node #61 receives data from output "Output" of node #54
- Input "I2" of node #61 receives data from output "Output" of node #56
- The parameter "Grouping method" in category "Input management" (jipipe:data-batch-generation/column-matching) is set to **"Custom"**
- The parameter "Custom grouping columns" in category "Input management" (jipipe:data-batch-generation/custom-matched-columns-expression) is set to **#Image**

**Node #62 "Cellpose prediction (2.x)" of type "Cellpose prediction (2.x)"**

- Input "Input" of node #62 receives data from output "Output" of node #56
- Input "Model" of node #62 receives data from output "Output" of node #48
- The parameter "Average object diameter" (diameter) is set to **45.0**
- The parameter "Override Python environment" (override-environment) is set to **[Disabled]**
- The parameter "Output probabilities" in category "Cellpose: Outputs" (output-parameters/output-probabilities) is set to **true**
- The parameter "Output flows XY" in category "Cellpose: Outputs" (output-parameters/output-flows-xy) is set to **true**
- The parameter "Cell probability threshold" in category "Cellpose: Thresholds" (threshold-parameters/cell-probability-threshold) is set to **-2.0**
- The parameter "Flow threshold (2D)" in category "Cellpose: Thresholds" (threshold-parameters/flow-threshold) is set to **0.0**

**Node #63 "Auto threshold 2D" of type "Auto threshold 2D"**

- Input "Input" of node #63 receives data from output "Probabilities" of node #57
- The parameter "Method" (method) is set to **"Shanbhag"**

**Node #64 "Auto threshold 2D" of type "Auto threshold 2D"**

- Input "Input" of node #64 receives data from output "Probabilities" of node #59
- The parameter "Method" (method) is set to **"Minimum"**

**Node #65 "Auto threshold 2D" of type "Auto threshold 2D"**

- Input "Input" of node #65 receives data from output "Probabilities" of node #62
- The parameter "Method" (method) is set to **"Intermodes"**
- The parameter "Grouping method" in category "Input management" (jipipe:data-batch-generation/column-matching) is set to **"Custom"**
- The parameter "Custom grouping columns" in category "Input management" (jipipe:data-batch-generation/custom-matched-columns-expression) is set to **Color**

**Node #66 "Morphological skeletonize 2D" of type "Morphological skeletonize 2D"**

- Input "Input" of node #66 receives data from output "Output" of node #63

**Node #67 "Euclidean distance transform 2D" of type "Euclidean distance transform 2D"**

- Input "Input" of node #67 receives data from output "Output" of node #63

**Node #68 "Morphological skeletonize 2D" of type "Morphological skeletonize 2D"**

- Input "Input" of node #68 receives data from output "Output" of node #64

**Node #69 "Euclidean distance transform 2D" of type "Euclidean distance transform 2D"**

- Input "Input" of node #69 receives data from output "Output" of node #64

**Node #70 "Euclidean distance transform 2D" of type "Euclidean distance transform 2D"**

- Input "Input" of node #70 receives data from output "Output" of node #65

**Node #71 "Median filter 2D" of type "Median filter 2D"**

- Input "Input" of node #71 receives data from output "Output" of node #65
- The parameter "Radius" (radius) is set to **3.0**

**Node #72 "Binary skeleton to 2D filaments" of type "Binary skeleton to 2D filaments"**

- Input "Skeleton" of node #72 receives data from output "Output" of node #66

**Node #73 "Binary skeleton to 2D filaments" of type "Binary skeleton to 2D filaments"**

- Input "Skeleton" of node #73 receives data from output "Output" of node #68

**Node #74 "Morphological skeletonize 2D" of type "Morphological skeletonize 2D"**

- Input "Input" of node #74 receives data from output "Output" of node #71

**Node #75 "Smooth filaments" of type "Smooth filaments"**

- Input "Input" of node #75 receives data from output "Filaments" of node #72
- The parameter "Factor (X)" (factor-x) is set to **15.0**
- The parameter "Factor (Y)" (factor-y) is set to **15.0**

**Node #76 "Smooth filaments" of type "Smooth filaments"**

- Input "Input" of node #76 receives data from output "Filaments" of node #73
- The parameter "Factor (X)" (factor-x) is set to **15.0**
- The parameter "Factor (Y)" (factor-y) is set to **15.0**

**Node #77 "Binary skeleton to 2D filaments" of type "Binary skeleton to 2D filaments"**

- Input "Skeleton" of node #77 receives data from output "Output" of node #74

**Node #78 "Set filament vertex radius from image" of type "Set filament vertex radius from image"**

- Input "Filaments" of node #78 receives data from output "Output" of node #75
- Input "Radius" of node #78 receives data from output "Output" of node #67

**Node #79 "Set filament vertex radius from image" of type "Set filament vertex radius from image"**

- Input "Filaments" of node #79 receives data from output "Output" of node #76
- Input "Radius" of node #79 receives data from output "Output" of node #69

**Node #80 "Smooth filaments" of type "Smooth filaments"**

- Input "Input" of node #80 receives data from output "Filaments" of node #77
- The parameter "Factor (X)" (factor-x) is set to **15.0**
- The parameter "Factor (Y)" (factor-y) is set to **15.0**

**Node #81 "Convert filaments to mask" of type "Convert filaments to mask"**

- Input "Input" of node #81 receives data from output "Output" of node #78
- Input "Reference" of node #81 receives data from output "Output" of node #53

**Node #82 "Convert filaments to mask" of type "Convert filaments to mask"**

- Input "Input" of node #82 receives data from output "Output" of node #79
- Input "Reference" of node #82 receives data from output "Output" of node #55

**Node #83 "Set filament vertex radius from image" of type "Set filament vertex radius from image"**

- Input "Filaments" of node #83 receives data from output "Output" of node #80
- Input "Radius" of node #83 receives data from output "Output" of node #70

**Node #84 "Auto threshold 2D" of type "Auto threshold 2D"**

- Input "Input" of node #84 receives data from output "Probabilities" of node #58
- Input "Mask" of node #84 receives data from output "Output" of node #81
- The parameter "Method" (method) is set to **"Shanbhag"**
- The parameter "Calculate threshold based on ..." (source-area) is set to **"InsideMask"**
- The parameter "Grouping method" in category "Input management" (jipipe:data-batch-generation/column-matching) is set to **"Custom"**
- The parameter "Custom grouping columns" in category "Input management" (jipipe:data-batch-generation/custom-matched-columns-expression) is set to **Color**

**Node #85 "Mask to ROI" of type "Mask to 2D ROI"**

- Input "Input" of node #85 receives data from output "Output" of node #81

**Node #86 "Set LUT (two colors)" of type "Set LUT (two colors)"**

- Input "Input" of node #86 receives data from output "Output" of node #81
- The parameter "Second color" (second-color) is set to **"#99FF99"**

**Node #87 "Mask to ROI" of type "Mask to 2D ROI"**

- Input "Input" of node #87 receives data from output "Output" of node #82

**Node #88 "Set LUT (two colors)" of type "Set LUT (two colors)"**

- Input "Input" of node #88 receives data from output "Output" of node #82
- The parameter "Second color" (second-color) is set to **"#FF66FF"**

**Node #89 "Coloc 2: Green vs. Magenta in Conf" of type "Coloc 2"**

- Input "Channel 1" of node #89 receives data from output "Output" of node #81
- Input "Channel 2" of node #89 receives data from output "Output" of node #82
- The parameter "Channel 2 name" (channel2-name) is set to **"ORANGE"**
- The parameter "Channel 1 name" (channel1-name) is set to **"RED"**
- The parameter "Grouping method" in category "Input management" (jipipe:data-batch-generation/column-matching) is set to **"Custom"**
- The parameter "Custom grouping columns" in category "Input management" (jipipe:data-batch-generation/custom-matched-columns-expression) is set to **#Lable**
- The parameter "Skip incomplete data sets" in category "Input management" (jipipe:data-batch-generation/skip-incomplete) is set to **true**

**Node #90 "Convert filaments to mask" of type "Convert filaments to mask"**

- Input "Input" of node #90 receives data from output "Output" of node #83
- Input "Reference" of node #90 receives data from output "Output" of node #55
- The parameter "Grouping method" in category "Input management" (jipipe:data-batch-generation/column-matching) is set to **"Custom"**
- The parameter "Custom grouping columns" in category "Input management" (jipipe:data-batch-generation/custom-matched-columns-expression) is set to **#label**

**Node #91 "Median filter 2D" of type "Median filter 2D"**

- Input "Input" of node #91 receives data from output "Output" of node #84
- The parameter "Radius" (radius) is set to **3.0**

**Node #92 "Euclidean distance transform 2D" of type "Euclidean distance transform 2D"**

- Input "Input" of node #92 receives data from output "Output" of node #84

**Node #93 "Combine ROI lists" of type "Merge 2D ROI lists (ordered)"**

- Input "Target" of node #93 receives data from output "Output" of node #85
- Input "Source" of node #93 receives data from output "Output" of node #87
- The parameter "Grouping method" in category "Merging iteration step generation" (jipipe:data-batch-generation/column-matching) is set to **"Custom"**
- The parameter "Custom grouping columns" in category "Merging iteration step generation" (jipipe:data-batch-generation/custom-matched-columns-expression) is set to **#Image**

**Node #94 "Overlay Green vs Magenbta in Conf" of type "Blend images"**

- Input "Bottom" of node #94 receives data from output "Output" of node #86
- Input "Top" of node #94 receives data from output "Output" of node #88
- The parameter "Custom grouping columns" in category "Input management" (jipipe:data-batch-generation/custom-matched-columns-expression) is set to **#Image + Modality + Label1 + Label2**
- The parameter "Skip incomplete data sets" in category "Input management" (jipipe:data-batch-generation/skip-incomplete) is set to **true**
- The parameter "Bottom" in category "Layers" (layers/Bottom) is set to **{"opacity":0.5}**
- The parameter "Top" in category "Layers" (layers/Top) is set to **{"opacity":1.0}**

**Node #95 "Add annotations as columns" of type "Add annotations as columns"**

- Input "Input" of node #95 receives data from output "Results" of node #89
- The parameter "Annotation name filter" (annotation-name-filter) is set to **#Image**

**Node #96 "Set LUT (two colors)" of type "Set LUT (two colors)"**

- Input "Input" of node #96 receives data from output "Output" of node #90
- The parameter "Second color" (second-color) is set to **"#FF66FF"**

**Node #97 "Coloc 2: CONF vs. STED in Magenta" of type "Coloc 2"**

- Input "Channel 1" of node #97 receives data from output "Output" of node #82
- Input "Channel 2" of node #97 receives data from output "Output" of node #90
- The parameter "Restrict to 2D ROI/mask" (input-masks) is set to **true**
- The parameter "Channel 2 name" (channel2-name) is set to **"STED"**
- The parameter "Channel 1 name" (channel1-name) is set to **"CONF"**
- The parameter "Grouping method" in category "Input management" (jipipe:data-batch-generation/column-matching) is set to **"Custom"**
- The parameter "Custom grouping columns" in category "Input management" (jipipe:data-batch-generation/custom-matched-columns-expression) is set to **#Label**
- The parameter "Skip incomplete data sets" in category "Input management" (jipipe:data-batch-generation/skip-incomplete) is set to **true**

**Node #98 "Morphological skeletonize 2D" of type "Morphological skeletonize 2D"**

- Input "Input" of node #98 receives data from output "Output" of node #91

**Node #99 "ROI calculator: merged CONF ROIs" of type "2D ROI calculator (AND/OR/XOR)"**

- Input "Input" of node #99 receives data from output "Output" of node #93
- The parameter "Split after operation" (split-afterwards) is set to **false**
- The parameter "Operation" (operation) is set to **"LogicalOr"**

**Node #100 "Merge table rows" of type "Merge table rows"**

- Input "Input" of node #100 receives data from output "Output" of node #95
- The parameter "Grouping method" in category "Merging iteration step generation" (jipipe:data-batch-generation/column-matching) is set to **"Custom"**
- The parameter "Custom grouping columns" in category "Merging iteration step generation" (jipipe:data-batch-generation/custom-matched-columns-expression) is set to **#Modality**
- The parameter "Skip incomplete data sets" in category "Merging iteration step generation" (jipipe:data-batch-generation/skip-incomplete) is set to **true**

**Node #101 "Overlay Conf vs. STED in Magenta" of type "Blend images"**

- Input "Bottom" of node #101 receives data from output "Output" of node #88
- Input "Top" of node #101 receives data from output "Output" of node #96
- The parameter "Skip incomplete data sets" in category "Input management" (jipipe:data-batch-generation/skip-incomplete) is set to **true**
- The parameter "Bottom" in category "Layers" (layers/Bottom) is set to **{"opacity":0.5}**
- The parameter "Top" in category "Layers" (layers/Top) is set to **{"opacity":1.0}**

**Node #102 "Add annotations as columns" of type "Add annotations as columns"**

- Input "Input" of node #102 receives data from output "Results" of node #97
- The parameter "Annotation name filter" (annotation-name-filter) is set to **#Image**

**Node #103 "Binary skeleton to 2D filaments" of type "Binary skeleton to 2D filaments"**

- Input "Skeleton" of node #103 receives data from output "Output" of node #98

**Node #104 "Set to value (grayscale): masking" of type "Set to value (grayscale)"**

- Input "Input" of node #104 receives data from output "Output" of node #94
- Input "ROI" of node #104 receives data from output "Output" of node #99
- The parameter "Only apply to ..." (roi:target-area) is set to **"OutsideRoi"**
- The parameter "Skip incomplete data sets" in category "Input management" (jipipe:data-batch-generation/skip-incomplete) is set to **true**

**Node #105 "Histogram plot" of type "Histogram plot"**

- Input "Input" of node #105 receives data from output "Output" of node #100
- The parameter "Series name" (series-name) is set to **"M2: RED vs. ORANGE in CONF"**
- The parameter item #1 of "Overridden parameters" in category "Adaptive parameters" (jipipe:adaptive-parameters/overridden-parameters) is set to **(#Subfolder, "plot-parameters/legend")**
- The parameter "Value" in category "Input columns" (input-columns/Value) is set to **("ExistingColumn", "Manders' tM2 (Above autothreshold of Ch1)")**
- The parameter "Bins" in category "Plot parameters" (plot-parameters/bins) is set to **20**
- The parameter "Bin axis label" in category "Plot parameters" (plot-parameters/bin-axis-label) is set to **"M2"**
- The parameter "Title" in category "Plot parameters" (plot-parameters/title) is set to **"Manders' tM2 green vs. magenta in CONF\""**
- The parameter "Value axis label" in category "Plot parameters" (plot-parameters/value-axis-label) is set to **"Frequency"**
- The parameter "Color map" in category "Plot parameters" (plot-parameters/color-map) is set to **"Set1"**
- The parameter "Histogram type" in category "Plot parameters" (plot-parameters/histogram-type) is set to **"RelativeFrequency"**

**Node #106 "Histogram plot" of type "Histogram plot"**

- Input "Input" of node #106 receives data from output "Output" of node #100
- The parameter "Series name" (series-name) is set to **"M1: RED vs. ORANGE in CONF"**
- The parameter item #1 of "Overridden parameters" in category "Adaptive parameters" (jipipe:adaptive-parameters/overridden-parameters) is set to **(#Subfolder, "plot-parameters/legend")**
- The parameter "Value" in category "Input columns" (input-columns/Value) is set to **("ExistingColumn", "Manders' tM1 (Above autothreshold of Ch2)")**
- The parameter "Bins" in category "Plot parameters" (plot-parameters/bins) is set to **20**
- The parameter "Bin axis label" in category "Plot parameters" (plot-parameters/bin-axis-label) is set to **"M2"**
- The parameter "Title" in category "Plot parameters" (plot-parameters/title) is set to **"Manders' tM1 green vs. magenta in CONF"**
- The parameter "Value axis label" in category "Plot parameters" (plot-parameters/value-axis-label) is set to **"Frequency"**
- The parameter "Color map" in category "Plot parameters" (plot-parameters/color-map) is set to **"Set1"**
- The parameter "Histogram type" in category "Plot parameters" (plot-parameters/histogram-type) is set to **"RelativeFrequency"**

**Node #107 "Set to value (grayscale) masking" of type "Set to value (grayscale)"**

- Input "Input" of node #107 receives data from output "Output" of node #101
- Input "ROI" of node #107 receives data from output "Output" of node #99
- The parameter "Only apply to ..." (roi:target-area) is set to **"OutsideRoi"**
- The parameter "Skip incomplete data sets" in category "Input management" (jipipe:data-batch-generation/skip-incomplete) is set to **true**

**Node #108 "Merge table rows" of type "Merge table rows"**

- Input "Input" of node #108 receives data from output "Output" of node #102
- The parameter "Grouping method" in category "Merging iteration step generation" (jipipe:data-batch-generation/column-matching) is set to **"Custom"**
- The parameter "Custom grouping columns" in category "Merging iteration step generation" (jipipe:data-batch-generation/custom-matched-columns-expression) is set to **#Label**

**Node #109 "Smooth filaments" of type "Smooth filaments"**

- Input "Input" of node #109 receives data from output "Filaments" of node #103
- The parameter "Factor (X)" (factor-x) is set to **15.0**
- The parameter "Factor (Y)" (factor-y) is set to **15.0**

**Node #110 "Histogram plot" of type "Histogram plot"**

- Input "Input" of node #110 receives data from output "Output" of node #108
- The parameter "Series name" (series-name) is set to **"M2: CONF vs. STED in ORANGE"**
- The parameter item #1 of "Overridden parameters" in category "Adaptive parameters" (jipipe:adaptive-parameters/overridden-parameters) is set to **(#Subfolder, "plot-parameters/legend")**
- The parameter "Value" in category "Input columns" (input-columns/Value) is set to **("ExistingColumn", "Manders' tM2 (Above autothreshold of Ch1)")**
- The parameter "Bins" in category "Plot parameters" (plot-parameters/bins) is set to **20**
- The parameter "Bin axis label" in category "Plot parameters" (plot-parameters/bin-axis-label) is set to **"M2"**
- The parameter "Title" in category "Plot parameters" (plot-parameters/title) is set to **"Manders' tM2 CONF vs. STED in magenta"**
- The parameter "Value axis label" in category "Plot parameters" (plot-parameters/value-axis-label) is set to **"Frequency"**
- The parameter "Color map" in category "Plot parameters" (plot-parameters/color-map) is set to **"Set1"**
- The parameter "Histogram type" in category "Plot parameters" (plot-parameters/histogram-type) is set to **"RelativeFrequency"**

**Node #111 "Histogram plot" of type "Histogram plot"**

- Input "Input" of node #111 receives data from output "Output" of node #108
- The parameter "Series name" (series-name) is set to **"M1: CONF vs. STED in ORANGE"**
- The parameter item #1 of "Overridden parameters" in category "Adaptive parameters" (jipipe:adaptive-parameters/overridden-parameters) is set to **(#Subfolder, "plot-parameters/legend")**
- The parameter "Value" in category "Input columns" (input-columns/Value) is set to **("ExistingColumn", "Manders' tM1 (Above autothreshold of Ch2)")**
- The parameter "Bins" in category "Plot parameters" (plot-parameters/bins) is set to **20**
- The parameter "Bin axis label" in category "Plot parameters" (plot-parameters/bin-axis-label) is set to **"M2"**
- The parameter "Title" in category "Plot parameters" (plot-parameters/title) is set to **"Manders' tM1CONF vs. STED in magenta"**
- The parameter "Value axis label" in category "Plot parameters" (plot-parameters/value-axis-label) is set to **"Frequency"**
- The parameter "Color map" in category "Plot parameters" (plot-parameters/color-map) is set to **"Set1"**
- The parameter "Histogram type" in category "Plot parameters" (plot-parameters/histogram-type) is set to **"RelativeFrequency"**

**Node #112 "Set filament vertex radius from image" of type "Set filament vertex radius from image"**

- Input "Filaments" of node #112 receives data from output "Output" of node #109
- Input "Radius" of node #112 receives data from output "Output" of node #92

**Node #113 "Convert filaments to mask" of type "Convert filaments to mask"**

- Input "Input" of node #113 receives data from output "Output" of node #112
- Input "Reference" of node #113 receives data from output "Output" of node #53
- The parameter "Grouping method" in category "Input management" (jipipe:data-batch-generation/column-matching) is set to **"Custom"**
- The parameter "Custom grouping columns" in category "Input management" (jipipe:data-batch-generation/custom-matched-columns-expression) is set to **#Label**

**Node #114 "Coloc 2: Green vs. Magenta in STED" of type "Coloc 2"**

- Input "Channel 1" of node #114 receives data from output "Output" of node #113
- Input "Channel 2" of node #114 receives data from output "Output" of node #90
- The parameter "Channel 2 name" (channel2-name) is set to **"ORANGE"**
- The parameter "Channel 1 name" (channel1-name) is set to **"RED"**
- The parameter "Grouping method" in category "Input management" (jipipe:data-batch-generation/column-matching) is set to **"Custom"**
- The parameter "Custom grouping columns" in category "Input management" (jipipe:data-batch-generation/custom-matched-columns-expression) is set to **#Image**
- The parameter "Skip incomplete data sets" in category "Input management" (jipipe:data-batch-generation/skip-incomplete) is set to **true**
- The parameter "Li Histogram Channel 2" in category "Colocalization settings" (coloc2-settings/li-histogram-channel-2) is set to **false**
- The parameter "Li Histogram Channel 1" in category "Colocalization settings" (coloc2-settings/li-histogram-channel-1) is set to **false**
- The parameter "Costes' Significance Test" in category "Colocalization settings" (coloc2-settings/costes-significance-test) is set to **false**
- The parameter "Kendall's Tau Rank Correlation" in category "Colocalization settings" (coloc2-settings/kendall-tau-rank-correlation) is set to **false**
- The parameter "Spearman's Rank Correlation" in category "Colocalization settings" (coloc2-settings/spearman-rank-correlation) is set to **false**
- The parameter "2D Intensity Histogram" in category "Colocalization settings" (coloc2-settings/2d-intensity-histogram) is set to **false**
- The parameter "Li ICQ" in category "Colocalization settings" (coloc2-settings/li-icq) is set to **false**

**Node #115 "Set LUT (two colors)" of type "Set LUT (two colors)"**

- Input "Input" of node #115 receives data from output "Output" of node #113
- The parameter "Second color" (second-color) is set to **"#99FF99"**

**Node #116 "Add annotations as columns" of type "Add annotations as columns"**

- Input "Input" of node #116 receives data from output "Results" of node #114
- The parameter "Annotation name filter" (annotation-name-filter) is set to **#Image**

**Node #117 "Overlay Conf vs. STED in Green" of type "Blend images"**

- Input "Bottom" of node #117 receives data from output "Output" of node #86
- Input "Top" of node #117 receives data from output "Output" of node #115
- The parameter "Grouping method" in category "Input management" (jipipe:data-batch-generation/column-matching) is set to **"PrefixHashIntersection"**
- The parameter "Skip incomplete data sets" in category "Input management" (jipipe:data-batch-generation/skip-incomplete) is set to **true**
- The parameter "Bottom" in category "Layers" (layers/Bottom) is set to **{"opacity":0.5}**
- The parameter "Top" in category "Layers" (layers/Top) is set to **{"opacity":1.0}**

**Node #118 "Overlay Green vs.Magenta in STED" of type "Blend images"**

- Input "Bottom" of node #118 receives data from output "Output" of node #115
- Input "Top" of node #118 receives data from output "Output" of node #96
- The parameter "Grouping method" in category "Input management" (jipipe:data-batch-generation/column-matching) is set to **"PrefixHashIntersection"**
- The parameter "Custom grouping columns" in category "Input management" (jipipe:data-batch-generation/custom-matched-columns-expression) is set to **#Image + Modality**
- The parameter "Skip incomplete data sets" in category "Input management" (jipipe:data-batch-generation/skip-incomplete) is set to **true**
- The parameter "Bottom" in category "Layers" (layers/Bottom) is set to **{"opacity":0.5}**
- The parameter "Top" in category "Layers" (layers/Top) is set to **{"opacity":0.5}**

**Node #119 "Coloc 2: CONF vs. STED in Green" of type "Coloc 2"**

- Input "Channel 1" of node #119 receives data from output "Output" of node #86
- Input "Channel 2" of node #119 receives data from output "Output" of node #115
- The parameter "Restrict to 2D ROI/mask" (input-masks) is set to **true**
- The parameter "Channel 2 name" (channel2-name) is set to **"STED"**
- The parameter "Channel 1 name" (channel1-name) is set to **"CONF"**
- The parameter "Grouping method" in category "Input management" (jipipe:data-batch-generation/column-matching) is set to **"Custom"**
- The parameter "Custom grouping columns" in category "Input management" (jipipe:data-batch-generation/custom-matched-columns-expression) is set to **#Label**
- The parameter "Threshold regression" in category "Colocalization settings" (coloc2-settings/threshold-regression) is set to **"Bisection"**

**Node #120 "Merge table rows" of type "Merge table rows"**

- Input "Input" of node #120 receives data from output "Output" of node #116
- The parameter "Grouping method" in category "Merging iteration step generation" (jipipe:data-batch-generation/column-matching) is set to **"Custom"**
- The parameter "Custom grouping columns" in category "Merging iteration step generation" (jipipe:data-batch-generation/custom-matched-columns-expression) is set to **#Modality**
- The parameter "Skip incomplete data sets" in category "Merging iteration step generation" (jipipe:data-batch-generation/skip-incomplete) is set to **true**

**Node #121 "Set to value (grayscale) masking" of type "Set to value (grayscale)"**

- Input "Input" of node #121 receives data from output "Output" of node #117
- Input "ROI" of node #121 receives data from output "Output" of node #99
- The parameter "Only apply to ..." (roi:target-area) is set to **"OutsideRoi"**
- The parameter "Skip incomplete data sets" in category "Input management" (jipipe:data-batch-generation/skip-incomplete) is set to **true**

**Node #122 "Set to value (grayscale) masking" of type "Set to value (grayscale)"**

- Input "Input" of node #122 receives data from output "Output" of node #118
- Input "ROI" of node #122 receives data from output "Output" of node #99
- The parameter "Only apply to ..." (roi:target-area) is set to **"OutsideRoi"**
- The parameter "Skip incomplete data sets" in category "Input management" (jipipe:data-batch-generation/skip-incomplete) is set to **true**

**Node #123 "Add annotations as columns" of type "Add annotations as columns"**

- Input "Input" of node #123 receives data from output "Results" of node #119
- The parameter "Annotation name filter" (annotation-name-filter) is set to **#Image**

**Node #124 "Histogram plot" of type "Histogram plot"**

- Input "Input" of node #124 receives data from output "Output" of node #120
- The parameter "Series name" (series-name) is set to **"M2: RED vs. ORANGE in STED"**
- The parameter item #1 of "Overridden parameters" in category "Adaptive parameters" (jipipe:adaptive-parameters/overridden-parameters) is set to **(#Subfolder, "plot-parameters/legend")**
- The parameter "Value" in category "Input columns" (input-columns/Value) is set to **("ExistingColumn", "Manders' tM2 (Above autothreshold of Ch1)")**
- The parameter "Bins" in category "Plot parameters" (plot-parameters/bins) is set to **20**
- The parameter "Bin axis label" in category "Plot parameters" (plot-parameters/bin-axis-label) is set to **"M2"**
- The parameter "Title" in category "Plot parameters" (plot-parameters/title) is set to **"Manders' tM2 green vs. magenta in STED"**
- The parameter "Value axis label" in category "Plot parameters" (plot-parameters/value-axis-label) is set to **"Frequency"**
- The parameter "Color map" in category "Plot parameters" (plot-parameters/color-map) is set to **"Set1"**
- The parameter "Histogram type" in category "Plot parameters" (plot-parameters/histogram-type) is set to **"RelativeFrequency"**

**Node #125 "Histogram plot" of type "Histogram plot"**

- Input "Input" of node #125 receives data from output "Output" of node #120
- The parameter "Series name" (series-name) is set to **"M1: RED vs. ORANGE in STED"**
- The parameter item #1 of "Overridden parameters" in category "Adaptive parameters" (jipipe:adaptive-parameters/overridden-parameters) is set to **(#Subfolder, "plot-parameters/legend")**
- The parameter "Value" in category "Input columns" (input-columns/Value) is set to **("ExistingColumn", "Manders' tM1 (Above autothreshold of Ch2)")**
- The parameter "Bins" in category "Plot parameters" (plot-parameters/bins) is set to **20**
- The parameter "Bin axis label" in category "Plot parameters" (plot-parameters/bin-axis-label) is set to **"M2"**
- The parameter "Title" in category "Plot parameters" (plot-parameters/title) is set to **"Manders' tM1 green vs. magenta in STED"**
- The parameter "Value axis label" in category "Plot parameters" (plot-parameters/value-axis-label) is set to **"Frequency"**
- The parameter "Color map" in category "Plot parameters" (plot-parameters/color-map) is set to **"Set1"**
- The parameter "Histogram type" in category "Plot parameters" (plot-parameters/histogram-type) is set to **"RelativeFrequency"**

**Node #126 "Merge table rows" of type "Merge table rows"**

- Input "Input" of node #126 receives data from output "Output" of node #123
- The parameter "Grouping method" in category "Merging iteration step generation" (jipipe:data-batch-generation/column-matching) is set to **"Custom"**
- The parameter "Custom grouping columns" in category "Merging iteration step generation" (jipipe:data-batch-generation/custom-matched-columns-expression) is set to **#Label**

**Node #127 "Histogram plot" of type "Histogram plot"**

- Input "Input" of node #127 receives data from output "Output" of node #126
- The parameter "Series name" (series-name) is set to **"M2: CONF vs. STED in RED"**
- The parameter item #1 of "Overridden parameters" in category "Adaptive parameters" (jipipe:adaptive-parameters/overridden-parameters) is set to **(#Subfolder, "plot-parameters/legend")**
- The parameter "Value" in category "Input columns" (input-columns/Value) is set to **("ExistingColumn", "Manders' tM2 (Above autothreshold of Ch1)")**
- The parameter "Bins" in category "Plot parameters" (plot-parameters/bins) is set to **20**
- The parameter "Bin axis label" in category "Plot parameters" (plot-parameters/bin-axis-label) is set to **"M2"**
- The parameter "Title" in category "Plot parameters" (plot-parameters/title) is set to **"Manders' tM2 CONF vs. STED in green"**
- The parameter "Value axis label" in category "Plot parameters" (plot-parameters/value-axis-label) is set to **"Frequency"**
- The parameter "Color map" in category "Plot parameters" (plot-parameters/color-map) is set to **"Set1"**
- The parameter "Histogram type" in category "Plot parameters" (plot-parameters/histogram-type) is set to **"RelativeFrequency"**

**Node #128 "Histogram plot" of type "Histogram plot"**

- Input "Input" of node #128 receives data from output "Output" of node #126
- The parameter "Series name" (series-name) is set to **"M1: CONF vs. STED in RED"**
- The parameter item #1 of "Overridden parameters" in category "Adaptive parameters" (jipipe:adaptive-parameters/overridden-parameters) is set to **(#Subfolder, "plot-parameters/legend")**
- The parameter "Value" in category "Input columns" (input-columns/Value) is set to **("ExistingColumn", "Manders' tM1 (Above autothreshold of Ch2)")**
- The parameter "Bins" in category "Plot parameters" (plot-parameters/bins) is set to **20**
- The parameter "Bin axis label" in category "Plot parameters" (plot-parameters/bin-axis-label) is set to **"M2"**
- The parameter "Title" in category "Plot parameters" (plot-parameters/title) is set to **"Manders' tM1 CONF vs. STED in green"**
- The parameter "Value axis label" in category "Plot parameters" (plot-parameters/value-axis-label) is set to **"Frequency"**
- The parameter "Color map" in category "Plot parameters" (plot-parameters/color-map) is set to **"Set1"**
- The parameter "Histogram type" in category "Plot parameters" (plot-parameters/histogram-type) is set to **"RelativeFrequency"**

**Node #129 "Output" of type "Compartment output"**

- Input "Coloc green CONF vs STED" of node #129 receives data from output "Output" of node #126
- Input "Coloc magenta CONF vs STED" of node #129 receives data from output "Output" of node #108
- Input "Coloc CONF green vs magenta" of node #129 receives data from output "Output" of node #100
- Input "Coloc STED RED vs ORANGE" of node #129 receives data from output "Output" of node #120
- Input "Overlay green vs. magenta in CONF" of node #129 receives data from output "Output" of node #104
- Input "Overlay green vs. magenta in STED" of node #129 receives data from output "Output" of node #122
- Input "Overlay CONF vs. STED in green" of node #129 receives data from output "Output" of node #121
- Input "Overlay CONF vs. STED in magenta" of node #129 receives data from output "Output" of node #107
- Input "green CONF" of node #129 receives data from output "Output" of node #81
- Input "RED STED" of node #129 receives data from output "Output" of node #113
- Input "ORANGE CONF" of node #129 receives data from output "Output" of node #82
- Input "ORANGE STED" of node #129 receives data from output "Output" of node #90
- Input "Merged RED and ORANGE ROIs in CONF" of node #129 receives data from output "Output" of node #99
- Input "M2 CONF vs. STED in RED" of node #129 receives data from output "Output" of node #127
- Input "M1 CONF vs. STED in RED" of node #129 receives data from output "Output" of node #128
- Input "M2 CONF vs. STED in ORANGE" of node #129 receives data from output "Output" of node #110
- Input "M1 CONF vs. STED in ORANGE" of node #129 receives data from output "Output" of node #111
- Input "M2 RED vs. ORANGE in CONF" of node #129 receives data from output "Output" of node #105
- Input "M1 RED vs. ORANGE in CONF" of node #129 receives data from output "Output" of node #106
- Input "M2 RED vs. ORANGE in STED" of node #129 receives data from output "Output" of node #124
- Input "M1 RED vs. ORANGE in STED" of node #129 receives data from output "Output" of node #125
- The parameter "jipipe:compartment:output-slot-name" (jipipe:compartment:output-slot-name) is set to **"Output"**

## Compartment C3 "Mask"

- The "Mask" compartment (C3) receives data from the "Preprocessing" compartment (C1)

**Node #37 "Output" of type "Compartment output"**

- Input "Annoated images" of node #37 receives data from output "Output" of node #36
- The parameter "jipipe:compartment:output-slot-name" (jipipe:compartment:output-slot-name) is set to **"Output"**

**Node #130 "Split & filter by annotation" of type "Split & filter by annotation"**

- Input "Input" of node #130 receives data from output "Annoated images" of node #37
- The parameter "Output" in category "Filters" (target-slots/Output) is set to **#Label CONTAINS "488"**
- The parameter "Output 1" in category "Filters" (target-slots/Output 1) is set to **#Label CONTAINS "594"**

**Node #131 "Filter by annotation (If else): green" of type "Filter by annotation (If else)"**

- Input "Input" of node #131 receives data from output "Output" of node #130
- The parameter "Filter" (filter) is set to **#Modality == "Conf"**

**Node #132 "Filter by annotation (If else) magenta" of type "Filter by annotation (If else)"**

- Input "Input" of node #132 receives data from output "Output 1" of node #130
- The parameter "Filter" (filter) is set to **#Modality == "Conf"**

**Node #133 "Divide by maximum" of type "Divide by maximum"**

- Input "Input" of node #133 receives data from output "Matched" of node #131

**Node #134 "Divide by maximum" of type "Divide by maximum"**

- Input "Input" of node #134 receives data from output "Unmatched" of node #131

**Node #135 "Divide by maximum" of type "Divide by maximum"**

- Input "Input" of node #135 receives data from output "Matched" of node #132

**Node #136 "Divide by maximum" of type "Divide by maximum"**

- Input "Input" of node #136 receives data from output "Unmatched" of node #132

**Node #137 "Set LUT (color map)" of type "Set LUT (color map)"**

- Input "Input" of node #137 receives data from output "Output" of node #134
- The parameter "Color map" (color-map) is set to **"green"**

**Node #138 "Fast image arithmetics" of type "Fast image arithmetics"**

- Input "I1" of node #138 receives data from output "Output" of node #133
- Input "I2" of node #138 receives data from output "Output" of node #135
- The parameter "Grouping method" in category "Input management" (jipipe:data-batch-generation/column-matching) is set to **"Custom"**
- The parameter "Custom grouping columns" in category "Input management" (jipipe:data-batch-generation/custom-matched-columns-expression) is set to **#Image**

**Node #139 "Fast image arithmetics" of type "Fast image arithmetics"**

- Input "I1" of node #139 receives data from output "Output" of node #134
- Input "I2" of node #139 receives data from output "Output" of node #136
- The parameter "Grouping method" in category "Input management" (jipipe:data-batch-generation/column-matching) is set to **"Custom"**
- The parameter "Custom grouping columns" in category "Input management" (jipipe:data-batch-generation/custom-matched-columns-expression) is set to **#Image**

**Node #140 "Set LUT (color map)" of type "Set LUT (color map)"**

- Input "Input" of node #140 receives data from output "Output" of node #136
- The parameter "Color map" (color-map) is set to **"magenta"**

**Node #141 "Gaussian blur 2D" of type "Gaussian blur 2D"**

- Input "Input" of node #141 receives data from output "Output" of node #138
- The parameter "Sigma (X)" (sigma-x) is set to **2.0**

**Node #142 "Gaussian blur 2D" of type "Gaussian blur 2D"**

- Input "Input" of node #142 receives data from output "Output" of node #139
- The parameter "Sigma (X)" (sigma-x) is set to **2.0**

**Node #143 "Morphological Feature Contrast (MFC) 2D" of type "Morphological Feature Contrast (MFC) 2D"**

- Input "Input" of node #143 receives data from output "Output" of node #141
- The parameter "Radius 2" (radius2) is set to **5**

**Node #144 "Morphological Feature Contrast (MFC) 2D" of type "Morphological Feature Contrast (MFC) 2D"**

- Input "Input" of node #144 receives data from output "Output" of node #142
- The parameter "Radius 2" (radius2) is set to **5**

**Node #145 "Euclidean distance transform 2D" of type "Euclidean distance transform 2D"**

- Input "Input" of node #145 receives data from output "Input" of node #143

**Node #146 "Auto threshold 2D" of type "Auto threshold 2D"**

- Input "Input" of node #146 receives data from output "Input" of node #143
- The parameter "Method" (method) is set to **"Li"**

**Node #147 "Auto threshold 2D" of type "Auto threshold 2D"**

- Input "Input" of node #147 receives data from output "Input" of node #144
- The parameter "Method" (method) is set to **"Li"**

**Node #148 "Euclidean distance transform 2D" of type "Euclidean distance transform 2D"**

- Input "Input" of node #148 receives data from output "Input" of node #144

**Node #149 "Morphological operation 2D" of type "Morphological operation 2D"**

- Input "Input" of node #149 receives data from output "Output" of node #146
- The parameter "Operation" (operation) is set to **"EROSION"**

**Node #150 "Morphological operation 2D" of type "Morphological operation 2D"**

- Input "Input" of node #150 receives data from output "Output" of node #147
- The parameter "Operation" (operation) is set to **"EROSION"**

**Node #151 "Find particles 2D" of type "Find particles 2D"**

- Input "Mask" of node #151 receives data from output "Output" of node #149
- The parameter "Min particle size" (min-particle-size) is set to **100.0**

**Node #152 "Find particles 2D" of type "Find particles 2D"**

- Input "Mask" of node #152 receives data from output "Output" of node #150
- The parameter "Min particle size" (min-particle-size) is set to **100.0**

**Node #153 "2D ROI to Labels (by name)" of type "2D ROI to Labels (by name)"**

- Input "ROI" of node #153 receives data from output "ROI" of node #151
- Input "Reference" of node #153 receives data from output "Output" of node #141

**Node #154 "2D ROI to Labels (by name)" of type "2D ROI to Labels (by name)"**

- Input "ROI" of node #154 receives data from output "ROI" of node #152
- Input "Reference" of node #154 receives data from output "Output" of node #142

**Node #155 "Morphological skeletonize 2D" of type "Morphological skeletonize 2D"**

- Input "Input" of node #155 receives data from output "Labels" of node #153

**Node #156 "Morphological skeletonize 2D" of type "Morphological skeletonize 2D"**

- Input "Input" of node #156 receives data from output "Labels" of node #154

**Node #157 "Binary skeleton to 2D filaments" of type "Binary skeleton to 2D filaments"**

- Input "Skeleton" of node #157 receives data from output "Output" of node #155

**Node #158 "Binary skeleton to 2D filaments" of type "Binary skeleton to 2D filaments"**

- Input "Skeleton" of node #158 receives data from output "Output" of node #156

**Node #159 "Connect filament vertices (Expression)" of type "Connect filament vertices (Expression)"**

- Input "Input" of node #159 receives data from output "Filaments" of node #157
- The parameter "Limit created edges (source/target)" (limit-connections) is set to **1**
- The parameter "Require direction" (require-direction) is set to **true**
- The parameter "Candidate edge filter" (filter-function) is set to **length < 40 AND source.degree == 1 AND target.degree == 1 AND dot_product < -0.8**

**Node #160 "Set filament vertex radius from image" of type "Set filament vertex radius from image"**

- Input "Filaments" of node #160 receives data from output "Filaments" of node #157
- Input "Radius" of node #160 receives data from output "Output" of node #145
- The parameter "Grouping method" in category "Input management" (jipipe:data-batch-generation/column-matching) is set to **"Custom"**
- The parameter "Custom grouping columns" in category "Input management" (jipipe:data-batch-generation/custom-matched-columns-expression) is set to **#Modality**

**Node #161 "Connect filament vertices (Expression)" of type "Connect filament vertices (Expression)"**

- Input "Input" of node #161 receives data from output "Filaments" of node #158
- The parameter "Limit created edges (source/target)" (limit-connections) is set to **1**
- The parameter "Require direction" (require-direction) is set to **true**
- The parameter "Candidate edge filter" (filter-function) is set to **length < 40 AND source.degree <= 2 AND target.degree <= 2 AND dot_product < -0.8**
- The parameter "Grouping method" in category "Input management" (jipipe:data-batch-generation/column-matching) is set to **"Custom"**
- The parameter "Custom grouping columns" in category "Input management" (jipipe:data-batch-generation/custom-matched-columns-expression) is set to **#Image**

**Node #162 "Smooth filaments" of type "Smooth filaments"**

- Input "Input" of node #162 receives data from output "Output" of node #159

**Node #163 "Smooth filaments" of type "Smooth filaments"**

- Input "Input" of node #163 receives data from output "Output" of node #161

**Node #164 "Set filament vertex radius from image" of type "Set filament vertex radius from image"**

- Input "Filaments" of node #164 receives data from output "Output" of node #161
- Input "Radius" of node #164 receives data from output "Output" of node #148
- The parameter "Grouping method" in category "Input management" (jipipe:data-batch-generation/column-matching) is set to **"Custom"**
- The parameter "Custom grouping columns" in category "Input management" (jipipe:data-batch-generation/custom-matched-columns-expression) is set to **#Image**

**Node #165 "Convert filaments to mask" of type "Convert filaments to mask"**

- Input "Input" of node #165 receives data from output "Output" of node #161
- Input "Reference" of node #165 receives data from output "Output" of node #142
- The parameter "Grouping method" in category "Input management" (jipipe:data-batch-generation/column-matching) is set to **"Custom"**
- The parameter "Custom grouping columns" in category "Input management" (jipipe:data-batch-generation/custom-matched-columns-expression) is set to **#Image**

**Node #166 "Find linear filament vertex order/distance" of type "Find linear filament vertex order/distance"**

- Input "Input" of node #166 receives data from output "Output" of node #162

**Node #167 "Convert filaments to RGB" of type "Convert filaments to RGB"**

- Input "Input" of node #167 receives data from output "Output" of node #162
- Input "Reference" of node #167 receives data from output "Output" of node #138
- The parameter "Grouping method" in category "Input management" (jipipe:data-batch-generation/column-matching) is set to **"Custom"**
- The parameter "Custom grouping columns" in category "Input management" (jipipe:data-batch-generation/custom-matched-columns-expression) is set to **#Image**

**Node #168 "Convert filaments to RGB" of type "Convert filaments to RGB"**

- Input "Input" of node #168 receives data from output "Output" of node #163
- Input "Reference" of node #168 receives data from output "Output" of node #139
- The parameter "Grouping method" in category "Input management" (jipipe:data-batch-generation/column-matching) is set to **"Custom"**
- The parameter "Custom grouping columns" in category "Input management" (jipipe:data-batch-generation/custom-matched-columns-expression) is set to **#Image**

**Node #169 "Find linear filament vertex order/distance" of type "Find linear filament vertex order/distance"**

- Input "Input" of node #169 receives data from output "Output" of node #163

**Node #170 "Convert filaments to mask" of type "Convert filaments to mask"**

- Input "Input" of node #170 receives data from output "Output" of node #164
- Input "Reference" of node #170 receives data from output "Output" of node #142
- The parameter "Grouping method" in category "Input management" (jipipe:data-batch-generation/column-matching) is set to **"Custom"**
- The parameter "Custom grouping columns" in category "Input management" (jipipe:data-batch-generation/custom-matched-columns-expression) is set to **#Image**

**Node #171 "Split filaments into connected components" of type "Split filaments into connected components"**

- Input "Input" of node #171 receives data from output "Output" of node #164

**Node #172 "Set filament vertex value from image" of type "Set filament vertex value from image"**

- Input "Filaments" of node #172 receives data from output "Output" of node #166
- Input "Intensity" of node #172 receives data from output "Output" of node #133
- The parameter "Backup old value" (backup-old-value) is set to **[Disabled]**
- The parameter "Grouping method" in category "Input management" (jipipe:data-batch-generation/column-matching) is set to **"Custom"**
- The parameter "Custom grouping columns" in category "Input management" (jipipe:data-batch-generation/custom-matched-columns-expression) is set to **#Modality**

**Node #173 "Convert filaments to mask" of type "Convert filaments to mask"**

- Input "Input" of node #173 receives data from output "Output" of node #162
- Input "Reference" of node #173 receives data from output "Output" of node #167

**Node #174 "Set filament vertex value from image: green intensity" of type "Set filament vertex value from image"**

- Input "Filaments" of node #174 receives data from output "Output" of node #169
- Input "Intensity" of node #174 receives data from output "Output" of node #134
- The parameter "Backup old value" (backup-old-value) is set to **"Green"**
- The parameter "Grouping method" in category "Input management" (jipipe:data-batch-generation/column-matching) is set to **"Custom"**
- The parameter "Custom grouping columns" in category "Input management" (jipipe:data-batch-generation/custom-matched-columns-expression) is set to **#Image**

**Node #175 "Convert filaments to mask" of type "Convert filaments to mask"**

- Input "Input" of node #175 receives data from output "Output" of node #171
- Input "Reference" of node #175 receives data from output "Output" of node #142
- The parameter "Grouping method" in category "Input management" (jipipe:data-batch-generation/column-matching) is set to **"Custom"**
- The parameter "Custom grouping columns" in category "Input management" (jipipe:data-batch-generation/custom-matched-columns-expression) is set to **#Image**

**Node #176 "Set filament vertex value from image" of type "Set filament vertex value from image"**

- Input "Filaments" of node #176 receives data from output "Output" of node #172
- Input "Intensity" of node #176 receives data from output "Output" of node #135
- The parameter "Backup old value" (backup-old-value) is set to **"Green"**
- The parameter "Grouping method" in category "Input management" (jipipe:data-batch-generation/column-matching) is set to **"Custom"**
- The parameter "Custom grouping columns" in category "Input management" (jipipe:data-batch-generation/custom-matched-columns-expression) is set to **#Modality**

**Node #177 "Blend images" of type "Blend images"**

- Input "Mask" of node #177 receives data from output "Output" of node #173
- Input "Green STED" of node #177 receives data from output "Output" of node #137
- Input "Magenta STED" of node #177 receives data from output "Output" of node #140
- The parameter "Grouping method" in category "Input management" (jipipe:data-batch-generation/column-matching) is set to **"Custom"**
- The parameter "Custom grouping columns" in category "Input management" (jipipe:data-batch-generation/custom-matched-columns-expression) is set to **#Image**
- The parameter "Mask" in category "Layers" (layers/Mask) is set to **{"opacity":0.0}**
- The parameter "Green STED" in category "Layers" (layers/Green STED) is set to **{"opacity":1.0}**
- The parameter "Magenta STED" in category "Layers" (layers/Magenta STED) is set to **{"opacity":1.0}**

**Node #178 "Set filament vertex value from image: red intensity" of type "Set filament vertex value from image"**

- Input "Filaments" of node #178 receives data from output "Output" of node #174
- Input "Intensity" of node #178 receives data from output "Output" of node #136
- The parameter "Backup old value" (backup-old-value) is set to **"Green"**
- The parameter "Grouping method" in category "Input management" (jipipe:data-batch-generation/column-matching) is set to **"Custom"**
- The parameter "Custom grouping columns" in category "Input management" (jipipe:data-batch-generation/custom-matched-columns-expression) is set to **#Image**

**Node #179 "Set filament vertex metadata to component index" of type "Set filament vertex metadata to component index"**

- Input "Input" of node #179 receives data from output "Output" of node #176

**Node #180 "Set filament vertex metadata to component index" of type "Set filament vertex metadata to component index"**

- Input "Input" of node #180 receives data from output "Output" of node #178

**Node #181 "Filter filament vertices" of type "Filter filament vertices"**

- Input "Input" of node #181 receives data from output "Output" of node #179
- The parameter "Only keep vertex if" (filter) is set to **degree <= 2**

**Node #182 "Filter filament vertices" of type "Filter filament vertices"**

- Input "Input" of node #182 receives data from output "Output" of node #180
- The parameter "Only keep vertex if" (filter) is set to **degree <= 3**

**Node #183 "Split filaments into connected components" of type "Split filaments into connected components"**

- Input "Input" of node #183 receives data from output "Output" of node #181

**Node #184 "Measure filament vertices" of type "Measure filament vertices"**

- Input "Input" of node #184 receives data from output "Output" of node #181

**Node #185 "Measure filament vertices" of type "Measure filament vertices"**

- Input "Input" of node #185 receives data from output "Output" of node #182

**Node #186 "Split filaments into connected components" of type "Split filaments into connected components"**

- Input "Input" of node #186 receives data from output "Output" of node #182

**Node #187 "Convert filaments to RGB" of type "Convert filaments to RGB"**

- Input "Input" of node #187 receives data from output "Output" of node #182
- Input "Reference" of node #187 receives data from output "Output" of node #139
- The parameter "Grouping method" in category "Input management" (jipipe:data-batch-generation/column-matching) is set to **"Custom"**
- The parameter "Custom grouping columns" in category "Input management" (jipipe:data-batch-generation/custom-matched-columns-expression) is set to **#Image**

**Node #188 "Filter filament components" of type "Filter filament components"**

- Input "Input" of node #188 receives data from output "Output" of node #183
- The parameter "Only keep filament if" (filter) is set to **lengthPixels > 20**

**Node #189 "Sort table rows" of type "Sort table rows"**

- Input "Input" of node #189 receives data from output "Output" of node #184
- The parameter item #1 of "Filters" (sort-order) is set to **(component_index, "Ascending")**
- The parameter item #2 of "Filters" (sort-order) is set to **(line_distance, "Ascending")**

**Node #190 "XY scatter plot" of type "XY scatter plot"**

- Input "Input" of node #190 receives data from output "Output" of node #184
- The parameter "Series name" (series-name) is set to **#Image**
- The parameter "X" in category "Input columns" (input-columns/X) is set to **("Generate", TO_NUMBER(line_distance))**
- The parameter "Y" in category "Input columns" (input-columns/Y) is set to **("Generate", TO_NUMBER(value) / TO_NUMBER(.value.Green))**
- The parameter "Title font size" in category "Plot parameters" (plot-parameters/title-font-size) is set to **20**
- The parameter "Title" in category "Plot parameters" (plot-parameters/title) is set to **"Magenta / Green intensity, confocal"**
- The parameter "X axis label" in category "Plot parameters" (plot-parameters/x-axis-label) is set to **"Line distance"**
- The parameter "Color map" in category "Plot parameters" (plot-parameters/color-map) is set to **"Set1"**
- The parameter "Y axis label" in category "Plot parameters" (plot-parameters/y-axis-label) is set to **"Magenta / Green"**

**Node #191 "XY scatter plot" of type "XY scatter plot"**

- Input "Input" of node #191 receives data from output "Output" of node #184
- The parameter "Series name" (series-name) is set to **#Image**
- The parameter "X" in category "Input columns" (input-columns/X) is set to **("Generate", TO_NUMBER(.value.Green))**
- The parameter "Y" in category "Input columns" (input-columns/Y) is set to **("Generate", TO_NUMBER(value))**
- The parameter "Title font size" in category "Plot parameters" (plot-parameters/title-font-size) is set to **20**
- The parameter "Title" in category "Plot parameters" (plot-parameters/title) is set to **"Green vs. Magenta intensity, confocal"**
- The parameter "X axis label" in category "Plot parameters" (plot-parameters/x-axis-label) is set to **"Green"**
- The parameter "Color map" in category "Plot parameters" (plot-parameters/color-map) is set to **"Set1"**
- The parameter "Y axis label" in category "Plot parameters" (plot-parameters/y-axis-label) is set to **"Magenta"**

**Node #192 "XY scatter plot" of type "XY scatter plot"**

- Input "Input" of node #192 receives data from output "Output" of node #184
- The parameter "Series name" (series-name) is set to **#Image**
- The parameter "X" in category "Input columns" (input-columns/X) is set to **("Generate", TO_NUMBER(value))**
- The parameter "Y" in category "Input columns" (input-columns/Y) is set to **("Generate", TO_NUMBER(.value.Green))**
- The parameter "Title font size" in category "Plot parameters" (plot-parameters/title-font-size) is set to **20**
- The parameter "Title" in category "Plot parameters" (plot-parameters/title) is set to **"Magenta vs. Green intensity, confocal"**
- The parameter "X axis label" in category "Plot parameters" (plot-parameters/x-axis-label) is set to **"Magenta"**
- The parameter "Color map" in category "Plot parameters" (plot-parameters/color-map) is set to **"Set1"**
- The parameter "Y axis label" in category "Plot parameters" (plot-parameters/y-axis-label) is set to **"Green"**

**Node #193 "XY scatter plot" of type "XY scatter plot"**

- Input "Input" of node #193 receives data from output "Output" of node #185
- The parameter "Series name" (series-name) is set to **#Image**
- The parameter "X" in category "Input columns" (input-columns/X) is set to **("Generate", TO_NUMBER(line_distance))**
- The parameter "Y" in category "Input columns" (input-columns/Y) is set to **("Generate", TO_NUMBER(value) / TO_NUMBER(.value.Green))**
- The parameter "Title font size" in category "Plot parameters" (plot-parameters/title-font-size) is set to **20**
- The parameter "Title" in category "Plot parameters" (plot-parameters/title) is set to **"Magenta / Green intensity, confocal"**
- The parameter "X axis label" in category "Plot parameters" (plot-parameters/x-axis-label) is set to **"Line distance"**
- The parameter "Color map" in category "Plot parameters" (plot-parameters/color-map) is set to **"Set1"**
- The parameter "Y axis label" in category "Plot parameters" (plot-parameters/y-axis-label) is set to **"Magenta / Green"**

**Node #194 "XY scatter plot" of type "XY scatter plot"**

- Input "Input" of node #194 receives data from output "Output" of node #185
- The parameter "Series name" (series-name) is set to **#Image**
- The parameter "X" in category "Input columns" (input-columns/X) is set to **("Generate", TO_NUMBER(.value.Green))**
- The parameter "Y" in category "Input columns" (input-columns/Y) is set to **("Generate", TO_NUMBER(value))**
- The parameter "Title font size" in category "Plot parameters" (plot-parameters/title-font-size) is set to **20**
- The parameter "Title" in category "Plot parameters" (plot-parameters/title) is set to **"Green vs. Magenta intensity, STED"**
- The parameter "X axis label" in category "Plot parameters" (plot-parameters/x-axis-label) is set to **"Green"**
- The parameter "Color map" in category "Plot parameters" (plot-parameters/color-map) is set to **"Set1"**
- The parameter "Y axis label" in category "Plot parameters" (plot-parameters/y-axis-label) is set to **"Magenta"**

**Node #195 "XY scatter plot" of type "XY scatter plot"**

- Input "Input" of node #195 receives data from output "Output" of node #185
- The parameter "Series name" (series-name) is set to **#Image**
- The parameter "X" in category "Input columns" (input-columns/X) is set to **("Generate", TO_NUMBER(value))**
- The parameter "Y" in category "Input columns" (input-columns/Y) is set to **("Generate", TO_NUMBER(.value.Green))**
- The parameter "Title font size" in category "Plot parameters" (plot-parameters/title-font-size) is set to **20**
- The parameter "Title" in category "Plot parameters" (plot-parameters/title) is set to **"Magenta vs. Green intensity, STED"**
- The parameter "X axis label" in category "Plot parameters" (plot-parameters/x-axis-label) is set to **"Magenta"**
- The parameter "Color map" in category "Plot parameters" (plot-parameters/color-map) is set to **"Set1"**
- The parameter "Y axis label" in category "Plot parameters" (plot-parameters/y-axis-label) is set to **"Green"**

**Node #196 "Sort table rows" of type "Sort table rows"**

- Input "Input" of node #196 receives data from output "Output" of node #185
- The parameter item #1 of "Filters" (sort-order) is set to **(component_index, "Ascending")**
- The parameter item #2 of "Filters" (sort-order) is set to **(line_distance, "Ascending")**

**Node #197 "Filter filament components" of type "Filter filament components"**

- Input "Input" of node #197 receives data from output "Output" of node #186
- The parameter "Only keep filament if" (filter) is set to **lengthPixels > 20 AND numVertices > 10**

**Node #198 "Blend images" of type "Blend images"**

- Input "Filemnts" of node #198 receives data from output "Output" of node #187
- Input "Merged raw" of node #198 receives data from output "Output" of node #139
- The parameter "Grouping method" in category "Input management" (jipipe:data-batch-generation/column-matching) is set to **"Custom"**
- The parameter "Custom grouping columns" in category "Input management" (jipipe:data-batch-generation/custom-matched-columns-expression) is set to **#Image**
- The parameter "Filemnts" in category "Layers" (layers/Filemnts) is set to **{"opacity":1.0}**
- The parameter "Merged raw" in category "Layers" (layers/Merged raw) is set to **{"opacity":0.4}**

**Node #199 "Find linear filament vertex order/distance" of type "Find linear filament vertex order/distance"**

- Input "Input" of node #199 receives data from output "Output" of node #188

**Node #200 "XY scatter plot" of type "XY scatter plot"**

- Input "Input" of node #200 receives data from output "Output" of node #189
- The parameter "Series name" (series-name) is set to **#Image**
- The parameter "Grouping method" in category "Merging iteration step generation" (jipipe:data-batch-generation/column-matching) is set to **"Custom"**
- The parameter "X" in category "Input columns" (input-columns/X) is set to **("Generate", TO_NUMBER(component_index))**
- The parameter "Y" in category "Input columns" (input-columns/Y) is set to **("Generate", TO_NUMBER(.value.Green))**
- The parameter "Title font size" in category "Plot parameters" (plot-parameters/title-font-size) is set to **20**
- The parameter "Title" in category "Plot parameters" (plot-parameters/title) is set to **"Green vs. component index, confocal"**
- The parameter "X axis label" in category "Plot parameters" (plot-parameters/x-axis-label) is set to **"Green"**
- The parameter "Color map" in category "Plot parameters" (plot-parameters/color-map) is set to **"Set1"**
- The parameter "Y axis label" in category "Plot parameters" (plot-parameters/y-axis-label) is set to **"Magenta"**

**Node #201 "XY scatter plot" of type "XY scatter plot"**

- Input "Input" of node #201 receives data from output "Output" of node #189
- The parameter "Series name" (series-name) is set to **#Image**
- The parameter "X" in category "Input columns" (input-columns/X) is set to **("Generate", TO_NUMBER(line_distance))**
- The parameter "Y" in category "Input columns" (input-columns/Y) is set to **("Generate", TO_NUMBER(.value.Green))**
- The parameter "Title font size" in category "Plot parameters" (plot-parameters/title-font-size) is set to **20**
- The parameter "Title" in category "Plot parameters" (plot-parameters/title) is set to **"Green intensity vs. line distance, confocal"**
- The parameter "X axis label" in category "Plot parameters" (plot-parameters/x-axis-label) is set to **"Line distance"**
- The parameter "Color map" in category "Plot parameters" (plot-parameters/color-map) is set to **"Set1"**
- The parameter "Y axis label" in category "Plot parameters" (plot-parameters/y-axis-label) is set to **"Green"**

**Node #202 "XY scatter plot" of type "XY scatter plot"**

- Input "Input" of node #202 receives data from output "Output" of node #189
- The parameter "Series name" (series-name) is set to **#Image**
- The parameter "X" in category "Input columns" (input-columns/X) is set to **("Generate", TO_NUMBER(value))**
- The parameter "Y" in category "Input columns" (input-columns/Y) is set to **("Generate", TO_NUMBER(.value.Green))**
- The parameter "Title font size" in category "Plot parameters" (plot-parameters/title-font-size) is set to **20**
- The parameter "Title" in category "Plot parameters" (plot-parameters/title) is set to **"Magenta vs. Green intensity, confocal"**
- The parameter "X axis label" in category "Plot parameters" (plot-parameters/x-axis-label) is set to **"Magenta"**
- The parameter "Color map" in category "Plot parameters" (plot-parameters/color-map) is set to **"Set1"**
- The parameter "Y axis label" in category "Plot parameters" (plot-parameters/y-axis-label) is set to **"Green"**

**Node #203 "XY scatter plot" of type "XY scatter plot"**

- Input "Input" of node #203 receives data from output "Output" of node #189
- The parameter "Series name" (series-name) is set to **#Image**
- The parameter "Grouping method" in category "Merging iteration step generation" (jipipe:data-batch-generation/column-matching) is set to **"Custom"**
- The parameter "X" in category "Input columns" (input-columns/X) is set to **("Generate", TO_NUMBER(component_index))**
- The parameter "Y" in category "Input columns" (input-columns/Y) is set to **("Generate", TO_NUMBER(value))**
- The parameter "Title font size" in category "Plot parameters" (plot-parameters/title-font-size) is set to **20**
- The parameter "Title" in category "Plot parameters" (plot-parameters/title) is set to **"Magenta vs. component index, confocal"**
- The parameter "X axis label" in category "Plot parameters" (plot-parameters/x-axis-label) is set to **"Component index"**
- The parameter "Color map" in category "Plot parameters" (plot-parameters/color-map) is set to **"Set1"**
- The parameter "Y axis label" in category "Plot parameters" (plot-parameters/y-axis-label) is set to **"Magenta"**

**Node #204 "XY scatter plot" of type "XY scatter plot"**

- Input "Input" of node #204 receives data from output "Output" of node #189
- The parameter "Series name" (series-name) is set to **#Image**
- The parameter "X" in category "Input columns" (input-columns/X) is set to **("Generate", TO_NUMBER(line_distance))**
- The parameter "Y" in category "Input columns" (input-columns/Y) is set to **("Generate", TO_NUMBER(value))**
- The parameter "Title font size" in category "Plot parameters" (plot-parameters/title-font-size) is set to **20**
- The parameter "Title" in category "Plot parameters" (plot-parameters/title) is set to **"Magenta intensity vs. line distance, confocal"**
- The parameter "X axis label" in category "Plot parameters" (plot-parameters/x-axis-label) is set to **"Line distance"**
- The parameter "Color map" in category "Plot parameters" (plot-parameters/color-map) is set to **"Set1"**
- The parameter "Y axis label" in category "Plot parameters" (plot-parameters/y-axis-label) is set to **"Magenta"**

**Node #205 "XY scatter plot" of type "XY scatter plot"**

- Input "Input" of node #205 receives data from output "Output" of node #196
- The parameter "Series name" (series-name) is set to **#Image**
- The parameter "Bookmark this node" (jipipe:node:bookmarked) is set to **true**
- The parameter "Grouping method" in category "Merging iteration step generation" (jipipe:data-batch-generation/column-matching) is set to **"Custom"**
- The parameter "X" in category "Input columns" (input-columns/X) is set to **("Generate", TO_NUMBER(component_index))**
- The parameter "Y" in category "Input columns" (input-columns/Y) is set to **("Generate", TO_NUMBER(.value.Green))**
- The parameter "Title font size" in category "Plot parameters" (plot-parameters/title-font-size) is set to **20**
- The parameter "Title" in category "Plot parameters" (plot-parameters/title) is set to **"Green vs. Component index, STED"**
- The parameter "X axis label" in category "Plot parameters" (plot-parameters/x-axis-label) is set to **"Component index"**
- The parameter "Color map" in category "Plot parameters" (plot-parameters/color-map) is set to **"Set1"**
- The parameter "Y axis label" in category "Plot parameters" (plot-parameters/y-axis-label) is set to **"Green"**

**Node #206 "XY scatter plot" of type "XY scatter plot"**

- Input "Input" of node #206 receives data from output "Output" of node #196
- The parameter "Series name" (series-name) is set to **#Image**
- The parameter "Bookmark this node" (jipipe:node:bookmarked) is set to **true**
- The parameter "Grouping method" in category "Merging iteration step generation" (jipipe:data-batch-generation/column-matching) is set to **"Custom"**
- The parameter "X" in category "Input columns" (input-columns/X) is set to **("Generate", TO_NUMBER(component_index))**
- The parameter "Y" in category "Input columns" (input-columns/Y) is set to **("Generate", TO_NUMBER(value))**
- The parameter "Title font size" in category "Plot parameters" (plot-parameters/title-font-size) is set to **20**
- The parameter "Title" in category "Plot parameters" (plot-parameters/title) is set to **"Magenta vs. component index, STED"**
- The parameter "X axis label" in category "Plot parameters" (plot-parameters/x-axis-label) is set to **"Component index"**
- The parameter "Color map" in category "Plot parameters" (plot-parameters/color-map) is set to **"Set1"**
- The parameter "Y axis label" in category "Plot parameters" (plot-parameters/y-axis-label) is set to **"Magenta"**

**Node #207 "XY scatter plot" of type "XY scatter plot"**

- Input "Input" of node #207 receives data from output "Output" of node #196
- The parameter "Series name" (series-name) is set to **#Image**
- The parameter "X" in category "Input columns" (input-columns/X) is set to **("Generate", TO_NUMBER(value))**
- The parameter "Y" in category "Input columns" (input-columns/Y) is set to **("Generate", TO_NUMBER(.value.Green))**
- The parameter "Y axis maximum" in category "Plot parameters" (plot-parameters/y-axis-maximum) is set to **1.0**
- The parameter "X axis maximum" in category "Plot parameters" (plot-parameters/x-axis-maximum) is set to **1.0**
- The parameter "Title font size" in category "Plot parameters" (plot-parameters/title-font-size) is set to **20**
- The parameter "Title" in category "Plot parameters" (plot-parameters/title) is set to **"Magenta vs. Green intensity, STED"**
- The parameter "X axis label" in category "Plot parameters" (plot-parameters/x-axis-label) is set to **"Magenta"**
- The parameter "Color map" in category "Plot parameters" (plot-parameters/color-map) is set to **"Set1"**
- The parameter "X axis font size" in category "Plot parameters" (plot-parameters/x-axis-font-size) is set to **20**
- The parameter "Y axis font size" in category "Plot parameters" (plot-parameters/y-axis-font-size) is set to **20**
- The parameter "Y axis label" in category "Plot parameters" (plot-parameters/y-axis-label) is set to **"Green"**

**Node #208 "Python script (iterating): co-plotting two colors per fibre ID" of type "Python script (iterating)"**

- Input "Green" of node #208 receives data from output "Output" of node #196
- The parameter "Script" (code) is set to **{"code":"from jipipe.imagej import *\nfrom scipy.spatial import KDTree\nimport pandas as pd\nfrom sklearn.neighbors import NearestNeighbors\nimport matplotlib.pyplot as plt\nfrom pathlib import Path\n\n\n# Get the input slot\nds = jipipe_inputs[\"Green\"]\n#ds1 = jipipe_inputs[\"Magenta\"]\n\n# Get the first table from the slot\ntable = load_table_file(data_slot=ds, row=0)\nValue = table[\".value.Green\"]\nxValue = table[\"component_index\"]\n\n# Get the first table from the slot\ntable1 = load_table_file(data_slot=ds, row=0)\nValue1 = table1[\"value\"]\n\n# Create a DataFrame\ndata = {\n 'Column1': Value\n}\n\ndata1 = {\n 'Column1': Value1\n}\n\ndf = pd.DataFrame(data)\ndf1 = pd.DataFrame(data1)\n\n# Create the plot\nplt.figure(figsize=(10, 6))\nplt.scatter(xValue, Value, color='magenta', label='Magenta', marker='o', s=15, facecolors='none', edgecolors='magenta')\nplt.scatter(xValue, Value1, color='green', label='Green', marker='s', s=12)\nplt.xlabel('Fiber number', fontsize=20)\nplt.ylabel('Color intensity', fontsize=20)\nplt.title('Magenta and green vs. fiber ID' + '\\n' + jipipe_annotations[\"#Folder\"] + \"_\" + jipipe_annotations[\"#Subfolder\"], fontsize = 20)\nplt.xticks(fontsize=20)\nplt.yticks(fontsize=20)\n\n# Get the output slot\ndso = jipipe_outputs[\"Plot\"]\n\nrow = dso.add_row()\nrow_storage_path = dso.get_row_storage_path(row)\n\nplt.savefig(row_storage_path / Path('coplot.png'), dpi=300)\n","collapsed":false,"external-script-file":{"content":"","enabled":false}}**
- The parameter "Override Python environment" (override-environment) is set to **[Disabled]**
- The parameter "Grouping method" in category "Input management" (jipipe:data-batch-generation/column-matching) is set to **"Custom"**
- The parameter "Custom grouping columns" in category "Input management" (jipipe:data-batch-generation/custom-matched-columns-expression) is set to **#Subfolder AND #Image AND #Modality**

**Node #209 "Summarize table" of type "Summarize table"**

- Input "Input" of node #209 receives data from output "Output" of node #196
- The parameter item #1 of "Processors" (processors) is set to **{"input":{"expression":"line_distance"},"parameter":{"id":"statistics-max"},"output":"Maxlinelength"}**

**Node #210 "Find linear filament vertex order/distance" of type "Find linear filament vertex order/distance"**

- Input "Input" of node #210 receives data from output "Output" of node #197

**Node #211 "Set filament vertex radius from image" of type "Set filament vertex radius from image"**

- Input "Filaments" of node #211 receives data from output "Output" of node #197
- Input "Radius" of node #211 receives data from output "Output" of node #148
- The parameter "Grouping method" in category "Input management" (jipipe:data-batch-generation/column-matching) is set to **"Custom"**
- The parameter "Custom grouping columns" in category "Input management" (jipipe:data-batch-generation/custom-matched-columns-expression) is set to **#Image**

**Node #212 "Convert filaments to mask" of type "Convert filaments to mask"**

- Input "Input" of node #212 receives data from output "Output" of node #197
- Input "Reference" of node #212 receives data from output "Output" of node #142
- The parameter "Grouping method" in category "Input management" (jipipe:data-batch-generation/column-matching) is set to **"Custom"**
- The parameter "Custom grouping columns" in category "Input management" (jipipe:data-batch-generation/custom-matched-columns-expression) is set to **#Image**
- The parameter "Skip incomplete data sets" in category "Input management" (jipipe:data-batch-generation/skip-incomplete) is set to **true**

**Node #213 "Measure filament vertices" of type "Measure filament vertices"**

- Input "Input" of node #213 receives data from output "Output" of node #199

**Node #214 "Export image" of type "Export image"**

- Input "Input" of node #214 receives data from output "Plot" of node #208
- The parameter "File path" (file-path) is set to **PATH_COMBINE(project_data_dirs["Analysisfolder"], "Magenta_Green_vs_fiberNumber_perImage_" + "_" + #Image)**

**Node #215 "Annotate data with table values" of type "Annotate data with table values"**

- Input "Data" of node #215 receives data from output "Output" of node #196
- Input "Table" of node #215 receives data from output "Output" of node #209
- The parameter item #1 of "Generated annotations" (generated-annotations) is set to **name = "Maxlinelength", value = Maxlinelength**

**Node #216 "Measure filament vertices" of type "Measure filament vertices"**

- Input "Input" of node #216 receives data from output "Output" of node #210

**Node #217 "Masks" of type "Compartment output"**

- Input "Radii-corrected filaments" of node #217 receives data from output "Output" of node #164
- Input "Individual filaments radii-corrected" of node #217 receives data from output "Output" of node #211
- Input "Annotated images" of node #217 receives data from output "Annoated images" of node #37
- Input "Masks per image" of node #217 receives data from output "Output" of node #170
- Input "Masks per filament" of node #217 receives data from output "Output" of node #175
- Input "Overlay mask with raw merged STED" of node #217 receives data from output "Output" of node #198
- Input "Filaments radius 1" of node #217 receives data from output "Output" of node #161
- Input "Mask from filament radius 1" of node #217 receives data from output "Output" of node #165
- Input "Mask from filament radius 1 per filament" of node #217 receives data from output "Output" of node #212
- The parameter "jipipe:compartment:output-slot-name" (jipipe:compartment:output-slot-name) is set to **"Masks"**

**Node #218 "Filter tables" of type "Filter tables"**

- Input "Input" of node #218 receives data from output "Output" of node #213
- The parameter "Filters" (filters) is set to **num_rows > 0**

**Node #219 "Apply expression per row" of type "Apply expression per row"**

- Input "Input" of node #219 receives data from output "Annotated data" of node #215
- The parameter item #1 of "Generated values" (entries) is set to **column-name = "Relativelinedistance", value = line_distance / line_length**

**Node #220 "Filter tables" of type "Filter tables"**

- Input "Input" of node #220 receives data from output "Output" of node #216
- The parameter "Filters" (filters) is set to **num_rows > 1**

**Node #221 "XY scatter plot" of type "XY scatter plot"**

- Input "Input" of node #221 receives data from output "Output" of node #218
- The parameter "Series name" (series-name) is set to **#Image**
- The parameter "X" in category "Input columns" (input-columns/X) is set to **("Generate", TO_NUMBER(line_distance))**
- The parameter "Y" in category "Input columns" (input-columns/Y) is set to **("Generate", TO_NUMBER(.value.Green))**
- The parameter "Title font size" in category "Plot parameters" (plot-parameters/title-font-size) is set to **20**
- The parameter "Title" in category "Plot parameters" (plot-parameters/title) is set to **"Magenta / Green intensity, confocal"**
- The parameter "X axis label" in category "Plot parameters" (plot-parameters/x-axis-label) is set to **"Line distance"**
- The parameter "Color map" in category "Plot parameters" (plot-parameters/color-map) is set to **"Set1"**
- The parameter "Y axis label" in category "Plot parameters" (plot-parameters/y-axis-label) is set to **"Green"**

**Node #222 "XY scatter plot" of type "XY scatter plot"**

- Input "Input" of node #222 receives data from output "Output" of node #218
- The parameter "Series name" (series-name) is set to **#Image**
- The parameter "X" in category "Input columns" (input-columns/X) is set to **("Generate", TO_NUMBER(.value.Green))**
- The parameter "Y" in category "Input columns" (input-columns/Y) is set to **("Generate", TO_NUMBER(value) / TO_NUMBER(.value.Green))**
- The parameter "Title font size" in category "Plot parameters" (plot-parameters/title-font-size) is set to **20**
- The parameter "Title" in category "Plot parameters" (plot-parameters/title) is set to **"Magenta / Green intensity, confocal"**
- The parameter "X axis label" in category "Plot parameters" (plot-parameters/x-axis-label) is set to **"Green"**
- The parameter "Color map" in category "Plot parameters" (plot-parameters/color-map) is set to **"Set1"**
- The parameter "Y axis label" in category "Plot parameters" (plot-parameters/y-axis-label) is set to **"Magenta / Green"**

**Node #223 "XY scatter plot" of type "XY scatter plot"**

- Input "Input" of node #223 receives data from output "Output" of node #218
- The parameter "Series name" (series-name) is set to **#Image**
- The parameter "X" in category "Input columns" (input-columns/X) is set to **("Generate", TO_NUMBER(.value.Green))**
- The parameter "Y" in category "Input columns" (input-columns/Y) is set to **("Generate", TO_NUMBER(.value.Green) / TO_NUMBER(value))**
- The parameter "Title font size" in category "Plot parameters" (plot-parameters/title-font-size) is set to **20**
- The parameter "Title" in category "Plot parameters" (plot-parameters/title) is set to **"Green / Magenta intensity, confocal"**
- The parameter "X axis label" in category "Plot parameters" (plot-parameters/x-axis-label) is set to **"Green"**
- The parameter "Color map" in category "Plot parameters" (plot-parameters/color-map) is set to **"Set1"**
- The parameter "Y axis label" in category "Plot parameters" (plot-parameters/y-axis-label) is set to **"Green / Magenta"**

**Node #224 "XY scatter plot" of type "XY scatter plot"**

- Input "Input" of node #224 receives data from output "Output" of node #218
- The parameter "Series name" (series-name) is set to **#Image**
- The parameter "X" in category "Input columns" (input-columns/X) is set to **("Generate", TO_NUMBER(line_distance))**
- The parameter "Y" in category "Input columns" (input-columns/Y) is set to **("Generate", TO_NUMBER(value))**
- The parameter "Title font size" in category "Plot parameters" (plot-parameters/title-font-size) is set to **20**
- The parameter "Title" in category "Plot parameters" (plot-parameters/title) is set to **"Magenta intensity, confocal"**
- The parameter "X axis label" in category "Plot parameters" (plot-parameters/x-axis-label) is set to **"Line distance"**
- The parameter "Color map" in category "Plot parameters" (plot-parameters/color-map) is set to **"Set1"**
- The parameter "Y axis label" in category "Plot parameters" (plot-parameters/y-axis-label) is set to **"Magenta"**

**Node #225 "XY scatter plot" of type "XY scatter plot"**

- Input "Input" of node #225 receives data from output "Output" of node #218
- The parameter "Series name" (series-name) is set to **#Image**
- The parameter "X" in category "Input columns" (input-columns/X) is set to **("Generate", TO_NUMBER(line_distance))**
- The parameter "Y" in category "Input columns" (input-columns/Y) is set to **("Generate", TO_NUMBER(value) / TO_NUMBER(.value.Green))**
- The parameter "Title font size" in category "Plot parameters" (plot-parameters/title-font-size) is set to **20**
- The parameter "Title" in category "Plot parameters" (plot-parameters/title) is set to **"Magenta / Green intensity, confocal"**
- The parameter "X axis label" in category "Plot parameters" (plot-parameters/x-axis-label) is set to **"Line distance"**
- The parameter "Color map" in category "Plot parameters" (plot-parameters/color-map) is set to **"Set1"**
- The parameter "Y axis label" in category "Plot parameters" (plot-parameters/y-axis-label) is set to **"Magenta / Green"**

**Node #226 "Apply expression per row" of type "Apply expression per row"**

- Input "Input" of node #226 receives data from output "Output" of node #219
- The parameter item #1 of "Generated values" (entries) is set to **column-name = "RelativelinedistanceCorr", value = IF_ELSE_EXPR(IS_NAN(Relativelinedistance), 0, Relativelinedistance)**

**Node #227 "Add table column" of type "Add table column"**

- Input "Input" of node #227 receives data from output "Output" of node #220
- The parameter item #1 of "Columns" (columns) is set to **(0, "line_distance")**
- The parameter item #2 of "Columns" (columns) is set to **(0, "line_length")**
- The parameter "Ensure minimum number of rows" (ensure-min-number-of-rows) is set to **1**

**Node #228 "Plots confocal" of type "Compartment output"**

- Input "Ratio vs. green" of node #228 receives data from output "Output" of node #222
- Input "Ratio vs. line distance" of node #228 receives data from output "Output" of node #225
- Input "Inverse ratio vs green" of node #228 receives data from output "Output" of node #223
- Input "Magenta vs. line distance" of node #228 receives data from output "Output" of node #224
- Input "Green vs. line distance" of node #228 receives data from output "Output" of node #221
- Input "Green vs. component index per image" of node #228 receives data from output "Output" of node #200
- Input "Green vs. Magenta per image" of node #228 receives data from output "Output" of node #202
- Input "Green vs. line distance per image" of node #228 receives data from output "Output" of node #201
- Input "Magenta vs. component index per image" of node #228 receives data from output "Output" of node #203
- Input "Magenta vs. line distance per image" of node #228 receives data from output "Output" of node #204
- Input "Ratio vs green per image" of node #228 receives data from output "Output" of node #190
- Input "Magenta vs Green per image" of node #228 receives data from output "Output" of node #191
- Input "Green vs. Magenta per image2" of node #228 receives data from output "Output" of node #192
- The parameter "jipipe:compartment:output-slot-name" (jipipe:compartment:output-slot-name) is set to **"Plots confocal"**

**Node #229 "XY scatter plot: green along the fibre" of type "XY scatter plot"**

- Input "Input" of node #229 receives data from output "Output" of node #226
- The parameter "Series name" (series-name) is set to **#Image**
- The parameter "X" in category "Input columns" (input-columns/X) is set to **("Generate", TO_NUMBER(RelativelinedistanceCorr))**
- The parameter "Y" in category "Input columns" (input-columns/Y) is set to **("Generate", TO_NUMBER(.value.Green))**
- The parameter "Title font size" in category "Plot parameters" (plot-parameters/title-font-size) is set to **20**
- The parameter "Title" in category "Plot parameters" (plot-parameters/title) is set to **"Green intensity, STED"**
- The parameter "X axis label" in category "Plot parameters" (plot-parameters/x-axis-label) is set to **"Relative distance along the molecule"**
- The parameter "Color map" in category "Plot parameters" (plot-parameters/color-map) is set to **"Accent"**
- The parameter "Y axis label" in category "Plot parameters" (plot-parameters/y-axis-label) is set to **"Magenta / Green"**

**Node #230 "Python script (iterating): co-plotting two colors per fibre ID" of type "Python script (iterating)"**

- Input "Green" of node #230 receives data from output "Output" of node #226
- The parameter "Script" (code) is set to **{"code":"from jipipe.imagej import *\nfrom scipy.spatial import KDTree\nimport pandas as pd\nfrom sklearn.neighbors import NearestNeighbors\nimport matplotlib.pyplot as plt\nfrom pathlib import Path\n\n\n# Get the input slot\nds = jipipe_inputs[\"Green\"]\n#ds1 = jipipe_inputs[\"Magenta\"]\n\n# Get the first table from the slot\ntable = load_table_file(data_slot=ds, row=0)\nValue = table[\".value.Green\"]\nxValue = table[\"RelativelinedistanceCorr\"]\n\n# Get the first table from the slot\ntable1 = load_table_file(data_slot=ds, row=0)\nValue1 = table1[\"value\"]\n\n# Create a DataFrame\ndata = {\n 'Column1': Value\n}\n\ndata1 = {\n 'Column1': Value1\n}\n\ndf = pd.DataFrame(data)\ndf1 = pd.DataFrame(data1)\n\n# Create the plot\nplt.figure(figsize=(10, 6))\nplt.scatter(xValue, Value, color='magenta', label='Magenta', marker='o', s=15, facecolors='none', edgecolors='magenta')\nplt.scatter(xValue, Value1, color='green', label='Green', marker='s', s=12)\nplt.xlabel('Fiber distance', fontsize=20)\nplt.ylabel('Color intensity', fontsize=20)\nplt.title('Magenta and green vs. fiber distance' + '\\n' + jipipe_annotations[\"#Folder\"] + \"_\" + jipipe_annotations[\"#Subfolder\"], fontsize = 20)\nplt.xticks(fontsize=20)\nplt.yticks(fontsize=20)\n\n# Get the output slot\ndso = jipipe_outputs[\"Plot\"]\n\nrow = dso.add_row()\nrow_storage_path = dso.get_row_storage_path(row)\n\nplt.savefig(row_storage_path / Path('coplot.png'), dpi=300)\n","collapsed":false,"external-script-file":{"content":"","enabled":false}}**
- The parameter "Override Python environment" (override-environment) is set to **[Disabled]**
- The parameter "Grouping method" in category "Input management" (jipipe:data-batch-generation/column-matching) is set to **"Custom"**
- The parameter "Custom grouping columns" in category "Input management" (jipipe:data-batch-generation/custom-matched-columns-expression) is set to **#Subfolder AND #Image AND #Modality**

**Node #231 "XY scatter plot: magenta along the fibre" of type "XY scatter plot"**

- Input "Input" of node #231 receives data from output "Output" of node #226
- The parameter "Series name" (series-name) is set to **#Image**
- The parameter "X" in category "Input columns" (input-columns/X) is set to **("Generate", TO_NUMBER(RelativelinedistanceCorr))**
- The parameter "Y" in category "Input columns" (input-columns/Y) is set to **("Generate", TO_NUMBER(value))**
- The parameter "Title font size" in category "Plot parameters" (plot-parameters/title-font-size) is set to **20**
- The parameter "Title" in category "Plot parameters" (plot-parameters/title) is set to **"Magenta intensity vs. line distance, STED"**
- The parameter "X axis label" in category "Plot parameters" (plot-parameters/x-axis-label) is set to **"Relative distance along the molecule"**
- The parameter "Color map" in category "Plot parameters" (plot-parameters/color-map) is set to **"Dark2"**
- The parameter "Y axis label" in category "Plot parameters" (plot-parameters/y-axis-label) is set to **"Magenta"**

**Node #232 "Python script (iterating): co-plotting two colors per fibre ID" of type "Python script (iterating)"**

- Input "Green" of node #232 receives data from output "Output" of node #227
- The parameter "Script" (code) is set to **{"code":"from jipipe.imagej import *\nfrom scipy.spatial import KDTree\nimport pandas as pd\nfrom sklearn.neighbors import NearestNeighbors\nimport matplotlib.pyplot as plt\nfrom pathlib import Path\n\n\n# Get the input slot\nds = jipipe_inputs[\"Green\"]\n#ds1 = jipipe_inputs[\"Magenta\"]\n\n# Get the first table from the slot\ntable = load_table_file(data_slot=ds, row=0)\nValue = table[\".value.Green\"]\nxValue = table[\"component_index\"]\n\n# Get the first table from the slot\ntable1 = load_table_file(data_slot=ds, row=0)\nValue1 = table1[\"value\"]\n\n# Create a DataFrame\ndata = {\n 'Column1': Value\n}\n\ndata1 = {\n 'Column1': Value1\n}\n\ndf = pd.DataFrame(data)\ndf1 = pd.DataFrame(data1)\n\n# Create the plot\nplt.figure(figsize=(10, 6))\nplt.scatter(xValue, Value, color='magenta', label='Magenta', marker='o', s=15, facecolors='none', edgecolors='magenta')\nplt.scatter(xValue, Value1, color='green', label='Green', marker='s', s=12)\nplt.xlabel('Fiber number', fontsize=20)\nplt.ylabel('Color intensity', fontsize=20)\nplt.title('Magenta and green vs. fiber ID' + '\\n' + jipipe_annotations[\"#Folder\"] + \"_\" + jipipe_annotations[\"#Subfolder\"], fontsize=20)\nplt.xticks(fontsize=20)\nplt.yticks(fontsize=20)\n\n# Get the output slot\ndso = jipipe_outputs[\"Plot\"]\n\nrow = dso.add_row()\nrow_storage_path = dso.get_row_storage_path(row)\n\nplt.savefig(row_storage_path / Path('coplot.png'), dpi=300)\n","collapsed":false,"external-script-file":{"content":"","enabled":false}}**
- The parameter "Override Python environment" (override-environment) is set to **[Disabled]**
- The parameter "Grouping method" in category "Input management" (jipipe:data-batch-generation/column-matching) is set to **"Custom"**
- The parameter "Custom grouping columns" in category "Input management" (jipipe:data-batch-generation/custom-matched-columns-expression) is set to **#Subfolder AND #Image AND #Modality**

**Node #233 "XY scatter plot" of type "XY scatter plot"**

- Input "Input" of node #233 receives data from output "Output" of node #227
- The parameter "Series name" (series-name) is set to **#Image**
- The parameter "X" in category "Input columns" (input-columns/X) is set to **("Generate", TO_NUMBER(RelativelinedistanceCorr))**
- The parameter "Y" in category "Input columns" (input-columns/Y) is set to **("Generate", TO_NUMBER(value) / TO_NUMBER(.value.Green))**
- The parameter "Title font size" in category "Plot parameters" (plot-parameters/title-font-size) is set to **20**
- The parameter "Title" in category "Plot parameters" (plot-parameters/title) is set to **"Magenta / Green intensity, STED"**
- The parameter "X axis label" in category "Plot parameters" (plot-parameters/x-axis-label) is set to **"Line distance"**
- The parameter "Color map" in category "Plot parameters" (plot-parameters/color-map) is set to **"Set1"**
- The parameter "Y axis label" in category "Plot parameters" (plot-parameters/y-axis-label) is set to **"Magenta / Green"**

**Node #234 "XY scatter plot" of type "XY scatter plot"**

- Input "Input" of node #234 receives data from output "Output" of node #227
- The parameter "Series name" (series-name) is set to **#Image**
- The parameter "X" in category "Input columns" (input-columns/X) is set to **("Generate", TO_NUMBER(.value.Green))**
- The parameter "Y" in category "Input columns" (input-columns/Y) is set to **("Generate", TO_NUMBER(value) / TO_NUMBER(.value.Green))**
- The parameter "Title font size" in category "Plot parameters" (plot-parameters/title-font-size) is set to **20**
- The parameter "Title" in category "Plot parameters" (plot-parameters/title) is set to **"Magenta / Green intensity, STED"**
- The parameter "X axis label" in category "Plot parameters" (plot-parameters/x-axis-label) is set to **"Green"**
- The parameter "Color map" in category "Plot parameters" (plot-parameters/color-map) is set to **"Set1"**
- The parameter "Y axis label" in category "Plot parameters" (plot-parameters/y-axis-label) is set to **"Magenta / Green"**

**Node #235 "Apply expression per row" of type "Apply expression per row"**

- Input "Input" of node #235 receives data from output "Output" of node #227
- The parameter item #1 of "Generated values" (entries) is set to **column-name = "Relativelinedistance", value = line_distance / line_length**

**Node #236 "XY scatter plot" of type "XY scatter plot"**

- Input "Input" of node #236 receives data from output "Output" of node #227
- The parameter "Series name" (series-name) is set to **#Image**
- The parameter "X" in category "Input columns" (input-columns/X) is set to **("Generate", TO_NUMBER(.value.Green))**
- The parameter "Y" in category "Input columns" (input-columns/Y) is set to **("Generate", TO_NUMBER(.value.Green) / TO_NUMBER(value))**
- The parameter "Title font size" in category "Plot parameters" (plot-parameters/title-font-size) is set to **20**
- The parameter "Title" in category "Plot parameters" (plot-parameters/title) is set to **"Green vs. Magenta intensity, STED"**
- The parameter "X axis label" in category "Plot parameters" (plot-parameters/x-axis-label) is set to **"Green"**
- The parameter "Color map" in category "Plot parameters" (plot-parameters/color-map) is set to **"Set1"**
- The parameter "Y axis label" in category "Plot parameters" (plot-parameters/y-axis-label) is set to **"Green / Magenta"**

**Node #237 "Export image" of type "Export image"**

- Input "Input" of node #237 receives data from output "Plot" of node #230
- The parameter "File path" (file-path) is set to **PATH_COMBINE(project_data_dirs["Analysisfolder"], "Magenta_Green_vs_fiberDistance_perImage_" + "_" + #Image)**

**Node #238 "Export image" of type "Export image"**

- Input "Input" of node #238 receives data from output "Plot" of node #232
- The parameter "File path" (file-path) is set to **PATH_COMBINE(project_data_dirs["Analysisfolder"], "Magenta_Green_vs_fiberNumber_perFibre_" + "__Image_" + #Image + "__FiberID_" + #Component)**

**Node #239 "Apply expression per row" of type "Apply expression per row"**

- Input "Input" of node #239 receives data from output "Output" of node #235
- The parameter item #1 of "Generated values" (entries) is set to **column-name = "RelativelinedistanceCorr", value = IF_ELSE_EXPR(IS_NAN(Relativelinedistance), 0, Relativelinedistance)**

**Node #240 "XY scatter plot: green along the fibre" of type "XY scatter plot"**

- Input "Input" of node #240 receives data from output "Output" of node #239
- The parameter "Series name" (series-name) is set to **#Image**
- The parameter "X" in category "Input columns" (input-columns/X) is set to **("Generate", TO_NUMBER(RelativelinedistanceCorr))**
- The parameter "Y" in category "Input columns" (input-columns/Y) is set to **("Generate", TO_NUMBER(.value.Green))**
- The parameter "Title font size" in category "Plot parameters" (plot-parameters/title-font-size) is set to **20**
- The parameter "Title" in category "Plot parameters" (plot-parameters/title) is set to **"Green intensity, STED"**
- The parameter "X axis label" in category "Plot parameters" (plot-parameters/x-axis-label) is set to **"Line distance"**
- The parameter "Color map" in category "Plot parameters" (plot-parameters/color-map) is set to **"Set1"**
- The parameter "X axis font size" in category "Plot parameters" (plot-parameters/x-axis-font-size) is set to **20**
- The parameter "Y axis font size" in category "Plot parameters" (plot-parameters/y-axis-font-size) is set to **20**
- The parameter "Y axis label" in category "Plot parameters" (plot-parameters/y-axis-label) is set to **"Green"**
- The parameter "Show legend" in category "Plot parameters" (plot-parameters/with-legend) is set to **false**

**Node #241 "Python script (iterating): co-plotting two colors per fibre ID" of type "Python script (iterating)"**

- Input "Green" of node #241 receives data from output "Output" of node #239
- The parameter "Script" (code) is set to **{"code":"from jipipe.imagej import *\nfrom scipy.spatial import KDTree\nimport pandas as pd\nfrom sklearn.neighbors import NearestNeighbors\nimport matplotlib.pyplot as plt\nfrom pathlib import Path\n\n\n# Get the input slot\nds = jipipe_inputs[\"Green\"]\n#ds1 = jipipe_inputs[\"Magenta\"]\n\n# Get the first table from the slot\ntable = load_table_file(data_slot=ds, row=0)\nValue = table[\".value.Green\"]\nxValue = table[\"RelativelinedistanceCorr\"]\n\n# Get the first table from the slot\ntable1 = load_table_file(data_slot=ds, row=0)\nValue1 = table1[\"value\"]\n\n# Create a DataFrame\ndata = {\n 'Column1': Value\n}\n\ndata1 = {\n 'Column1': Value1\n}\n\ndf = pd.DataFrame(data)\ndf1 = pd.DataFrame(data1)\n\n# Create the scatter plot\nplt.figure(figsize=(10, 6))\nplt.scatter(xValue, Value, color='magenta', label='Magenta', marker='o', s=15, facecolors='none', edgecolors='magenta')\nplt.scatter(xValue, Value1, color='green', label='Green', marker='s', s=12)\nplt.xlabel('Fiber distance', fontsize = 20)\nplt.ylabel('Color intensity', fontsize = 20)\nplt.title('Magenta and green vs. fiber distance' + '\\n' + jipipe_annotations[\"#Folder\"] + \"_\" + jipipe_annotations[\"#Subfolder\"], fontsize = 20)\nplt.xticks(fontsize=20)\nplt.yticks(fontsize=20)\n\n\n# Get the output slot\ndso = jipipe_outputs[\"Plot\"]\n\nrow = dso.add_row()\nrow_storage_path = dso.get_row_storage_path(row)\n\nplt.savefig(row_storage_path / Path('coplot.png'), dpi=300)\n\n\n\n# Create the line plot\nplt.figure(figsize=(10, 6))\n\nplt.plot(\n xValue, Value, \n marker='o', # Marker type (circle)\n markersize=15, # Marker size\n markerfacecolor='magenta', # Marker fill color\n markeredgecolor='black', # Marker edge color (border)\n color='magenta', # Line color\n linestyle='-' # Line style (dashed)\n)\n\nplt.plot(\n xValue, Value1, \n marker='o', # Marker type (circle)\n markersize=15, # Marker size\n markerfacecolor='green', # Marker fill color\n markeredgecolor='black', # Marker edge color (border)\n color='green', # Line color\n linestyle='--' # Line style (dashed)\n)\n\nplt.xlabel('Fiber distance', fontsize = 20)\nplt.ylabel('Color intensity', fontsize = 20)\nplt.title('Magenta and green vs. fiber distance' + '\\n' + jipipe_annotations[\"#Folder\"] + \"_\" + jipipe_annotations[\"#Subfolder\"], fontsize = 20)\nplt.xticks(fontsize=20)\nplt.yticks(fontsize=20)\n\n\n\n# Get the output slot\ndso = jipipe_outputs[\"Plot\"]\n\nrow = dso.add_row()\nrow_storage_path = dso.get_row_storage_path(row)\n\nplt.savefig(row_storage_path / Path('coplot.png'), dpi=300)\n","collapsed":false,"external-script-file":{"content":"","enabled":false}}**
- The parameter "Override Python environment" (override-environment) is set to **[Disabled]**
- The parameter "Grouping method" in category "Input management" (jipipe:data-batch-generation/column-matching) is set to **"Custom"**
- The parameter "Custom grouping columns" in category "Input management" (jipipe:data-batch-generation/custom-matched-columns-expression) is set to **#Subfolder AND #Image AND #Modality**

**Node #242 "XY scatter plot: magenta along the fibre" of type "XY scatter plot"**

- Input "Input" of node #242 receives data from output "Output" of node #239
- The parameter "Series name" (series-name) is set to **#Image**
- The parameter "X" in category "Input columns" (input-columns/X) is set to **("Generate", TO_NUMBER(RelativelinedistanceCorr))**
- The parameter "Y" in category "Input columns" (input-columns/Y) is set to **("Generate", TO_NUMBER(value))**
- The parameter "Title font size" in category "Plot parameters" (plot-parameters/title-font-size) is set to **20**
- The parameter "Title" in category "Plot parameters" (plot-parameters/title) is set to **"Magenta intensity, STED"**
- The parameter "X axis label" in category "Plot parameters" (plot-parameters/x-axis-label) is set to **"Line distance"**
- The parameter "Color map" in category "Plot parameters" (plot-parameters/color-map) is set to **"Set1"**
- The parameter "X axis font size" in category "Plot parameters" (plot-parameters/x-axis-font-size) is set to **20**
- The parameter "Y axis font size" in category "Plot parameters" (plot-parameters/y-axis-font-size) is set to **20**
- The parameter "Y axis label" in category "Plot parameters" (plot-parameters/y-axis-label) is set to **"Magenta"**
- The parameter "Show legend" in category "Plot parameters" (plot-parameters/with-legend) is set to **false**

**Node #243 "Export image" of type "Export image"**

- Input "Input" of node #243 receives data from output "Plot" of node #241
- The parameter "File path" (file-path) is set to **PATH_COMBINE(project_data_dirs["Analysisfolder"], "Magenta_Green_vs_fiberDistance_perFiber_" + "Image__" + #Image + "__FiberID_" + #Component)**

**Node #244 "Plots STED" of type "Compartment output"**

- Input "Ratio vs. green" of node #244 receives data from output "Output" of node #234
- Input "Ratio vs line distance" of node #244 receives data from output "Output" of node #233
- Input "Inverse ratio vs. green" of node #244 receives data from output "Output" of node #236
- Input "Magenta vs. line distance" of node #244 receives data from output "Output" of node #242
- Input "Green vs. line distance" of node #244 receives data from output "Output" of node #240
- Input "Green vs component index per image" of node #244 receives data from output "Output" of node #205
- Input "Green vs Magenta per image" of node #244 receives data from output "Output" of node #207
- Input "Magenta vs line distance per image" of node #244 receives data from output "Output" of node #231
- Input "Magenta vs.component index per image" of node #244 receives data from output "Output" of node #206
- Input "Ratio vs line distance per image" of node #244 receives data from output "Output" of node #193
- Input "Magenta vs green per image" of node #244 receives data from output "Output" of node #194
- Input "Green vs magenta per image" of node #244 receives data from output "Output" of node #195
- Input "Green vs. line distance per image" of node #244 receives data from output "Output" of node #229
- The parameter "jipipe:compartment:output-slot-name" (jipipe:compartment:output-slot-name) is set to **"Plots STED"**

## Compartment C4 "Filaments and colocalization in STED"

- The "Filaments and colocalization in STED" compartment (C4) receives data from the "Segmentation and colocalization" compartment (C2)

**Node #129 "Output" of type "Compartment output"**

- Input "Coloc green CONF vs STED" of node #129 receives data from output "Output" of node #126
- Input "Coloc magenta CONF vs STED" of node #129 receives data from output "Output" of node #108
- Input "Coloc CONF green vs magenta" of node #129 receives data from output "Output" of node #100
- Input "Coloc STED RED vs ORANGE" of node #129 receives data from output "Output" of node #120
- Input "Overlay green vs. magenta in CONF" of node #129 receives data from output "Output" of node #104
- Input "Overlay green vs. magenta in STED" of node #129 receives data from output "Output" of node #122
- Input "Overlay CONF vs. STED in green" of node #129 receives data from output "Output" of node #121
- Input "Overlay CONF vs. STED in magenta" of node #129 receives data from output "Output" of node #107
- Input "green CONF" of node #129 receives data from output "Output" of node #81
- Input "RED STED" of node #129 receives data from output "Output" of node #113
- Input "ORANGE CONF" of node #129 receives data from output "Output" of node #82
- Input "ORANGE STED" of node #129 receives data from output "Output" of node #90
- Input "Merged RED and ORANGE ROIs in CONF" of node #129 receives data from output "Output" of node #99
- Input "M2 CONF vs. STED in RED" of node #129 receives data from output "Output" of node #127
- Input "M1 CONF vs. STED in RED" of node #129 receives data from output "Output" of node #128
- Input "M2 CONF vs. STED in ORANGE" of node #129 receives data from output "Output" of node #110
- Input "M1 CONF vs. STED in ORANGE" of node #129 receives data from output "Output" of node #111
- Input "M2 RED vs. ORANGE in CONF" of node #129 receives data from output "Output" of node #105
- Input "M1 RED vs. ORANGE in CONF" of node #129 receives data from output "Output" of node #106
- Input "M2 RED vs. ORANGE in STED" of node #129 receives data from output "Output" of node #124
- Input "M1 RED vs. ORANGE in STED" of node #129 receives data from output "Output" of node #125
- The parameter "jipipe:compartment:output-slot-name" (jipipe:compartment:output-slot-name) is set to **"Output"**

**Node #245 "Morphological skeletonize 2D" of type "Morphological skeletonize 2D"**

- Input "Input" of node #245 receives data from output "RED STED" of node #129

**Node #246 "Morphological operation 2D" of type "Morphological operation 2D"**

- Input "Input" of node #246 receives data from output "RED STED" of node #129

**Node #247 "Morphological skeletonize 2D" of type "Morphological skeletonize 2D"**

- Input "Input" of node #247 receives data from output "ORANGE STED" of node #129

**Node #248 "Morphological operation 2D" of type "Morphological operation 2D"**

- Input "Input" of node #248 receives data from output "ORANGE STED" of node #129

**Node #249 "Set to value (grayscale)" of type "Set to value (grayscale)"**

- Input "Input" of node #249 receives data from output "Output" of node #245
- The parameter "Only apply to ..." (roi:target-area) is set to **"OutsideMask"**
- The parameter "Skip incomplete data sets" in category "Input management" (jipipe:data-batch-generation/skip-incomplete) is set to **true**

**Node #250 "Morphological operation 2D" of type "Morphological operation 2D"**

- Input "Input" of node #250 receives data from output "Output" of node #246
- The parameter "Operation" (operation) is set to **"EROSION"**

**Node #251 "Set to value (grayscale)" of type "Set to value (grayscale)"**

- Input "Input" of node #251 receives data from output "Output" of node #247
- Input "Mask" of node #251 receives data from output "ORANGE CONF" of node #129
- The parameter "Only apply to ..." (roi:target-area) is set to **"OutsideMask"**
- The parameter "Skip incomplete data sets" in category "Input management" (jipipe:data-batch-generation/skip-incomplete) is set to **true**

**Node #252 "Morphological operation 2D" of type "Morphological operation 2D"**

- Input "Input" of node #252 receives data from output "Output" of node #248
- The parameter "Operation" (operation) is set to **"EROSION"**

**Node #253 "Binary skeleton to 2D filaments" of type "Binary skeleton to 2D filaments"**

- Input "Skeleton" of node #253 receives data from output "Output" of node #249

**Node #254 "Gaussian blur 2D" of type "Gaussian blur 2D"**

- Input "Input" of node #254 receives data from output "Output" of node #250

**Node #255 "Binary skeleton to 2D filaments" of type "Binary skeleton to 2D filaments"**

- Input "Skeleton" of node #255 receives data from output "Output" of node #251

**Node #256 "Gaussian blur 2D" of type "Gaussian blur 2D"**

- Input "Input" of node #256 receives data from output "Output" of node #252

**Node #257 "Filter filaments" of type "Filter filament components"**

- Input "Input" of node #257 receives data from output "Filaments" of node #253
- The parameter "Only keep filament if" (filter) is set to **lengthPixels > 3**

**Node #258 "Auto threshold 2D" of type "Auto threshold 2D"**

- Input "Input" of node #258 receives data from output "Output" of node #254
- The parameter "Method" (method) is set to **"Otsu"**
- The parameter "Threshold annotation" (threshold-annotation) is set to **"Threshold"**

**Node #259 "Filter filaments" of type "Filter filament components"**

- Input "Input" of node #259 receives data from output "Filaments" of node #255
- The parameter "Only keep filament if" (filter) is set to **lengthPixels > 3**

**Node #260 "Auto threshold 2D" of type "Auto threshold 2D"**

- Input "Input" of node #260 receives data from output "Output" of node #256
- The parameter "Method" (method) is set to **"Otsu"**
- The parameter "Threshold annotation" (threshold-annotation) is set to **"Threshold"**

**Node #261 "Measure filaments" of type "Measure filament components"**

- Input "Input" of node #261 receives data from output "Output" of node #257

**Node #262 "Convert filaments to mask" of type "Convert filaments to mask"**

- Input "Input" of node #262 receives data from output "Output" of node #257
- Input "Reference" of node #262 receives data from output "RED STED" of node #129
- The parameter "Skip incomplete data sets" in category "Input management" (jipipe:data-batch-generation/skip-incomplete) is set to **true**

**Node #263 "Morphological skeletonize 2D" of type "Morphological skeletonize 2D"**

- Input "Input" of node #263 receives data from output "Output" of node #258

**Node #264 "Convert filaments to mask" of type "Convert filaments to mask"**

- Input "Input" of node #264 receives data from output "Output" of node #259
- Input "Reference" of node #264 receives data from output "ORANGE STED" of node #129
- The parameter "Skip incomplete data sets" in category "Input management" (jipipe:data-batch-generation/skip-incomplete) is set to **true**

**Node #265 "Measure filaments" of type "Measure filament components"**

- Input "Input" of node #265 receives data from output "Output" of node #259

**Node #266 "Morphological skeletonize 2D" of type "Morphological skeletonize 2D"**

- Input "Input" of node #266 receives data from output "Output" of node #260

**Node #267 "Add annotations as columns" of type "Add annotations as columns"**

- Input "Input" of node #267 receives data from output "Output" of node #261
- The parameter "Annotation name filter" (annotation-name-filter) is set to **#Image**

**Node #268 "Set to value (grayscale)" of type "Set to value (grayscale)"**

- Input "Input" of node #268 receives data from output "Output" of node #263
- The parameter "Only apply to ..." (roi:target-area) is set to **"OutsideMask"**
- The parameter "Skip incomplete data sets" in category "Input management" (jipipe:data-batch-generation/skip-incomplete) is set to **true**

**Node #269 "Coloc 2: positive RED vs. positive ORANGE in STED" of type "Coloc 2"**

- Input "ROI" of node #269 receives data from output "Merged RED and ORANGE ROIs in CONF" of node #129
- Input "Channel 1" of node #269 receives data from output "Output" of node #262
- Input "Channel 2" of node #269 receives data from output "Output" of node #264
- The parameter "Channel 2 name" (channel2-name) is set to **"ORANGE"**
- The parameter "Channel 1 name" (channel1-name) is set to **"RED"**
- The parameter "Skip incomplete data sets" in category "Input management" (jipipe:data-batch-generation/skip-incomplete) is set to **true**
- The parameter "Li Histogram Channel 2" in category "Colocalization settings" (coloc2-settings/li-histogram-channel-2) is set to **false**
- The parameter "Li Histogram Channel 1" in category "Colocalization settings" (coloc2-settings/li-histogram-channel-1) is set to **false**
- The parameter "Costes' Significance Test" in category "Colocalization settings" (coloc2-settings/costes-significance-test) is set to **false**
- The parameter "Kendall's Tau Rank Correlation" in category "Colocalization settings" (coloc2-settings/kendall-tau-rank-correlation) is set to **false**
- The parameter "PSF" in category "Colocalization settings" (coloc2-settings/psf) is set to **1**
- The parameter "Costes randomizations" in category "Colocalization settings" (coloc2-settings/costes-randomizations) is set to **1**
- The parameter "Spearman's Rank Correlation" in category "Colocalization settings" (coloc2-settings/spearman-rank-correlation) is set to **false**
- The parameter "2D Intensity Histogram" in category "Colocalization settings" (coloc2-settings/2d-intensity-histogram) is set to **false**
- The parameter "Li ICQ" in category "Colocalization settings" (coloc2-settings/li-icq) is set to **false**

**Node #270 "Add annotations as columns" of type "Add annotations as columns"**

- Input "Input" of node #270 receives data from output "Output" of node #265
- The parameter "Annotation name filter" (annotation-name-filter) is set to **#Image**

**Node #271 "Set to value (grayscale)" of type "Set to value (grayscale)"**

- Input "Input" of node #271 receives data from output "Output" of node #266
- Input "Mask" of node #271 receives data from output "ORANGE CONF" of node #129
- The parameter "Only apply to ..." (roi:target-area) is set to **"OutsideMask"**
- The parameter "Skip incomplete data sets" in category "Input management" (jipipe:data-batch-generation/skip-incomplete) is set to **true**

**Node #272 "Merge table rows" of type "Merge table rows"**

- Input "Input" of node #272 receives data from output "Output" of node #267
- The parameter "Grouping method" in category "Merging iteration step generation" (jipipe:data-batch-generation/column-matching) is set to **"MergeAll"**

**Node #273 "Binary skeleton to 2D filaments" of type "Binary skeleton to 2D filaments"**

- Input "Skeleton" of node #273 receives data from output "Output" of node #268

**Node #274 "Add annotations as columns" of type "Add annotations as columns"**

- Input "Input" of node #274 receives data from output "Results" of node #269
- The parameter "Annotation name filter" (annotation-name-filter) is set to **#Image**

**Node #275 "Merge table rows" of type "Merge table rows"**

- Input "Input" of node #275 receives data from output "Output" of node #270
- The parameter "Grouping method" in category "Merging iteration step generation" (jipipe:data-batch-generation/column-matching) is set to **"MergeAll"**

**Node #276 "Binary skeleton to 2D filaments" of type "Binary skeleton to 2D filaments"**

- Input "Skeleton" of node #276 receives data from output "Output" of node #271

**Node #277 "Filter filaments" of type "Filter filament components"**

- Input "Input" of node #277 receives data from output "Filaments" of node #273
- The parameter "Only keep filament if" (filter) is set to **lengthPixels > 2**

**Node #278 "Merge table rows" of type "Merge table rows"**

- Input "Input" of node #278 receives data from output "Output" of node #274
- The parameter "Grouping method" in category "Merging iteration step generation" (jipipe:data-batch-generation/column-matching) is set to **"Custom"**
- The parameter "Custom grouping columns" in category "Merging iteration step generation" (jipipe:data-batch-generation/custom-matched-columns-expression) is set to **#Modality**

**Node #279 "Filter filaments" of type "Filter filament components"**

- Input "Input" of node #279 receives data from output "Filaments" of node #276
- The parameter "Only keep filament if" (filter) is set to **lengthPixels > 2**

**Node #280 "Measure filaments" of type "Measure filament components"**

- Input "Input" of node #280 receives data from output "Output" of node #277

**Node #281 "Convert filaments to mask" of type "Convert filaments to mask"**

- Input "Input" of node #281 receives data from output "Output" of node #277
- The parameter "Skip incomplete data sets" in category "Input management" (jipipe:data-batch-generation/skip-incomplete) is set to **true**

**Node #282 "Histogram plot" of type "Histogram plot"**

- Input "Input" of node #282 receives data from output "Output" of node #278
- The parameter "Series name" (series-name) is set to **"M2: positive RED vs. positive ORANGE in STED"**
- The parameter item #1 of "Overridden parameters" in category "Adaptive parameters" (jipipe:adaptive-parameters/overridden-parameters) is set to **(#Subfolder, "plot-parameters/title")**
- The parameter "Value" in category "Input columns" (input-columns/Value) is set to **("ExistingColumn", "Manders' tM2 (Above autothreshold of Ch1)")**
- The parameter "Bins" in category "Plot parameters" (plot-parameters/bins) is set to **20**
- The parameter "Bin axis label" in category "Plot parameters" (plot-parameters/bin-axis-label) is set to **"M2"**
- The parameter "Title" in category "Plot parameters" (plot-parameters/title) is set to **""**
- The parameter "Value axis label" in category "Plot parameters" (plot-parameters/value-axis-label) is set to **"Frequency"**
- The parameter "Color map" in category "Plot parameters" (plot-parameters/color-map) is set to **"Set1"**
- The parameter "Histogram type" in category "Plot parameters" (plot-parameters/histogram-type) is set to **"RelativeFrequency"**

**Node #283 "Histogram plot" of type "Histogram plot"**

- Input "Input" of node #283 receives data from output "Output" of node #278
- The parameter "Series name" (series-name) is set to **"M1: positive RED vs. positive ORANGE in STED"**
- The parameter item #1 of "Overridden parameters" in category "Adaptive parameters" (jipipe:adaptive-parameters/overridden-parameters) is set to **(#Subfolder, "plot-parameters/title")**
- The parameter "Value" in category "Input columns" (input-columns/Value) is set to **("ExistingColumn", "Manders' tM1 (Above autothreshold of Ch2)")**
- The parameter "Bins" in category "Plot parameters" (plot-parameters/bins) is set to **20**
- The parameter "Bin axis label" in category "Plot parameters" (plot-parameters/bin-axis-label) is set to **"M1"**
- The parameter "Title" in category "Plot parameters" (plot-parameters/title) is set to **""**
- The parameter "Value axis label" in category "Plot parameters" (plot-parameters/value-axis-label) is set to **"Frequency"**
- The parameter "Color map" in category "Plot parameters" (plot-parameters/color-map) is set to **"Set1"**
- The parameter "Histogram type" in category "Plot parameters" (plot-parameters/histogram-type) is set to **"RelativeFrequency"**

**Node #284 "Convert filaments to mask" of type "Convert filaments to mask"**

- Input "Input" of node #284 receives data from output "Output" of node #279
- Input "Reference" of node #284 receives data from output "ORANGE STED" of node #129
- The parameter "Skip incomplete data sets" in category "Input management" (jipipe:data-batch-generation/skip-incomplete) is set to **true**

**Node #285 "Measure filaments" of type "Measure filament components"**

- Input "Input" of node #285 receives data from output "Output" of node #279

**Node #286 "Add annotations as columns" of type "Add annotations as columns"**

- Input "Input" of node #286 receives data from output "Output" of node #280
- The parameter "Annotation name filter" (annotation-name-filter) is set to **#Image**

**Node #287 "Morphological operation 2D" of type "Morphological operation 2D"**

- Input "Input" of node #287 receives data from output "Output" of node #281
- The parameter "Structure element" (element) is set to **"LINE_DIAG_DOWN"**

**Node #288 "Image calculator 2D: gaps in RED" of type "Legacy image calculator 2D"**

- Input "Input 1" of node #288 receives data from output "Output" of node #281
- Input "Input 2" of node #288 receives data from output "Output" of node #262
- The parameter "Function" (operation) is set to **"Subtract"**

**Node #289 "Image calculator 2D: gaps in ORANGE" of type "Legacy image calculator 2D"**

- Input "Input 1" of node #289 receives data from output "Output" of node #284
- Input "Input 2" of node #289 receives data from output "Output" of node #264
- The parameter "Function" (operation) is set to **"Subtract"**

**Node #290 "Morphological operation 2D" of type "Morphological operation 2D"**

- Input "Input" of node #290 receives data from output "Output" of node #284
- The parameter "Structure element" (element) is set to **"LINE_DIAG_DOWN"**

**Node #291 "Add annotations as columns" of type "Add annotations as columns"**

- Input "Input" of node #291 receives data from output "Output" of node #285
- The parameter "Annotation name filter" (annotation-name-filter) is set to **#Image**

**Node #292 "Merge table rows" of type "Merge table rows"**

- Input "Input" of node #292 receives data from output "Output" of node #286
- The parameter "Grouping method" in category "Merging iteration step generation" (jipipe:data-batch-generation/column-matching) is set to **"MergeAll"**

**Node #293 "Set LUT (two colors): RED" of type "Set LUT (two colors)"**

- Input "Input" of node #293 receives data from output "Output" of node #287

**Node #294 "Set LUT (two colors): RED" of type "Set LUT (two colors)"**

- Input "Input" of node #294 receives data from output "Output" of node #288

**Node #295 "Coloc 2: negative RED vs. positive ORANGE in STED " of type "Coloc 2"**

- Input "ROI" of node #295 receives data from output "Merged RED and ORANGE ROIs in CONF" of node #129
- Input "Channel 1" of node #295 receives data from output "Output" of node #288
- Input "Channel 2" of node #295 receives data from output "Output" of node #264
- The parameter "Channel 2 name" (channel2-name) is set to **"ORANGE"**
- The parameter "Channel 1 name" (channel1-name) is set to **"RED"**
- The parameter "Skip incomplete data sets" in category "Input management" (jipipe:data-batch-generation/skip-incomplete) is set to **true**
- The parameter "Li Histogram Channel 2" in category "Colocalization settings" (coloc2-settings/li-histogram-channel-2) is set to **false**
- The parameter "Li Histogram Channel 1" in category "Colocalization settings" (coloc2-settings/li-histogram-channel-1) is set to **false**
- The parameter "Costes' Significance Test" in category "Colocalization settings" (coloc2-settings/costes-significance-test) is set to **false**
- The parameter "Kendall's Tau Rank Correlation" in category "Colocalization settings" (coloc2-settings/kendall-tau-rank-correlation) is set to **false**
- The parameter "PSF" in category "Colocalization settings" (coloc2-settings/psf) is set to **1**
- The parameter "Costes randomizations" in category "Colocalization settings" (coloc2-settings/costes-randomizations) is set to **1**
- The parameter "Spearman's Rank Correlation" in category "Colocalization settings" (coloc2-settings/spearman-rank-correlation) is set to **false**
- The parameter "2D Intensity Histogram" in category "Colocalization settings" (coloc2-settings/2d-intensity-histogram) is set to **false**
- The parameter "Li ICQ" in category "Colocalization settings" (coloc2-settings/li-icq) is set to **false**

**Node #296 "Coloc 2: RED vs. ORANGE in STED negative ROIs" of type "Coloc 2"**

- Input "ROI" of node #296 receives data from output "Merged RED and ORANGE ROIs in CONF" of node #129
- Input "Channel 1" of node #296 receives data from output "Output" of node #288
- Input "Channel 2" of node #296 receives data from output "Output" of node #289
- The parameter "Channel 2 name" (channel2-name) is set to **"ORANGE"**
- The parameter "Channel 1 name" (channel1-name) is set to **"RED"**
- The parameter "Skip incomplete data sets" in category "Input management" (jipipe:data-batch-generation/skip-incomplete) is set to **true**
- The parameter "Li Histogram Channel 2" in category "Colocalization settings" (coloc2-settings/li-histogram-channel-2) is set to **false**
- The parameter "Li Histogram Channel 1" in category "Colocalization settings" (coloc2-settings/li-histogram-channel-1) is set to **false**
- The parameter "Costes' Significance Test" in category "Colocalization settings" (coloc2-settings/costes-significance-test) is set to **false**
- The parameter "Kendall's Tau Rank Correlation" in category "Colocalization settings" (coloc2-settings/kendall-tau-rank-correlation) is set to **false**
- The parameter "PSF" in category "Colocalization settings" (coloc2-settings/psf) is set to **1**
- The parameter "Costes randomizations" in category "Colocalization settings" (coloc2-settings/costes-randomizations) is set to **1**
- The parameter "Spearman's Rank Correlation" in category "Colocalization settings" (coloc2-settings/spearman-rank-correlation) is set to **false**
- The parameter "2D Intensity Histogram" in category "Colocalization settings" (coloc2-settings/2d-intensity-histogram) is set to **false**
- The parameter "Li ICQ" in category "Colocalization settings" (coloc2-settings/li-icq) is set to **false**

**Node #297 "Set LUT (two colors): ORANGE" of type "Set LUT (two colors)"**

- Input "Input" of node #297 receives data from output "Output" of node #289
- The parameter "Second color" (second-color) is set to **"#FF8C00"**

**Node #298 "Coloc 2: positive RED vs. negative ORANGE in STED " of type "Coloc 2"**

- Input "ROI" of node #298 receives data from output "Merged RED and ORANGE ROIs in CONF" of node #129
- Input "Channel 1" of node #298 receives data from output "Output" of node #262
- Input "Channel 2" of node #298 receives data from output "Output" of node #289
- The parameter "Channel 2 name" (channel2-name) is set to **"ORANGE"**
- The parameter "Channel 1 name" (channel1-name) is set to **"RED"**
- The parameter "Skip incomplete data sets" in category "Input management" (jipipe:data-batch-generation/skip-incomplete) is set to **true**
- The parameter "Li Histogram Channel 2" in category "Colocalization settings" (coloc2-settings/li-histogram-channel-2) is set to **false**
- The parameter "Li Histogram Channel 1" in category "Colocalization settings" (coloc2-settings/li-histogram-channel-1) is set to **false**
- The parameter "Costes' Significance Test" in category "Colocalization settings" (coloc2-settings/costes-significance-test) is set to **false**
- The parameter "Kendall's Tau Rank Correlation" in category "Colocalization settings" (coloc2-settings/kendall-tau-rank-correlation) is set to **false**
- The parameter "PSF" in category "Colocalization settings" (coloc2-settings/psf) is set to **1**
- The parameter "Costes randomizations" in category "Colocalization settings" (coloc2-settings/costes-randomizations) is set to **1**
- The parameter "Spearman's Rank Correlation" in category "Colocalization settings" (coloc2-settings/spearman-rank-correlation) is set to **false**
- The parameter "2D Intensity Histogram" in category "Colocalization settings" (coloc2-settings/2d-intensity-histogram) is set to **false**
- The parameter "Li ICQ" in category "Colocalization settings" (coloc2-settings/li-icq) is set to **false**

**Node #299 "Set LUT (two colors): ORANGE" of type "Set LUT (two colors)"**

- Input "Input" of node #299 receives data from output "Output" of node #290
- The parameter "Second color" (second-color) is set to **"#FF8C00"**

**Node #300 "Merge table rows" of type "Merge table rows"**

- Input "Input" of node #300 receives data from output "Output" of node #291
- The parameter "Grouping method" in category "Merging iteration step generation" (jipipe:data-batch-generation/column-matching) is set to **"MergeAll"**

**Node #301 "Add annotations as columns" of type "Add annotations as columns"**

- Input "Input" of node #301 receives data from output "Results" of node #295
- The parameter "Annotation name filter" (annotation-name-filter) is set to **#Image**

**Node #302 "Add annotations as columns" of type "Add annotations as columns"**

- Input "Input" of node #302 receives data from output "Results" of node #296
- The parameter "Annotation name filter" (annotation-name-filter) is set to **#Image**

**Node #303 "Blend images:: overlay of RED and ORANGE gaps" of type "Blend images"**

- Input "Bottom" of node #303 receives data from output "Output" of node #294
- Input "Top" of node #303 receives data from output "Output" of node #297
- The parameter "Skip incomplete data sets" in category "Input management" (jipipe:data-batch-generation/skip-incomplete) is set to **true**
- The parameter "Bottom" in category "Layers" (layers/Bottom) is set to **{"opacity":1.0}**
- The parameter "Top" in category "Layers" (layers/Top) is set to **{"opacity":1.0}**

**Node #304 "Add annotations as columns" of type "Add annotations as columns"**

- Input "Input" of node #304 receives data from output "Results" of node #298
- The parameter "Annotation name filter" (annotation-name-filter) is set to **#Image**

**Node #305 "Blend images:: overlay of RED and ORANGE " of type "Blend images"**

- Input "Bottom" of node #305 receives data from output "Output" of node #293
- Input "Top" of node #305 receives data from output "Output" of node #299
- The parameter "Skip incomplete data sets" in category "Input management" (jipipe:data-batch-generation/skip-incomplete) is set to **true**
- The parameter "Bottom" in category "Layers" (layers/Bottom) is set to **{"opacity":1.0}**
- The parameter "Top" in category "Layers" (layers/Top) is set to **{"opacity":1.0}**

**Node #306 "Merge table rows" of type "Merge table rows"**

- Input "Input" of node #306 receives data from output "Output" of node #301
- The parameter "Grouping method" in category "Merging iteration step generation" (jipipe:data-batch-generation/column-matching) is set to **"Custom"**
- The parameter "Custom grouping columns" in category "Merging iteration step generation" (jipipe:data-batch-generation/custom-matched-columns-expression) is set to **#Modality**

**Node #307 "Merge table rows" of type "Merge table rows"**

- Input "Input" of node #307 receives data from output "Output" of node #302
- The parameter "Grouping method" in category "Merging iteration step generation" (jipipe:data-batch-generation/column-matching) is set to **"Custom"**
- The parameter "Custom grouping columns" in category "Merging iteration step generation" (jipipe:data-batch-generation/custom-matched-columns-expression) is set to **#Modality**

**Node #308 "Merge table rows" of type "Merge table rows"**

- Input "Input" of node #308 receives data from output "Output" of node #304
- The parameter "Grouping method" in category "Merging iteration step generation" (jipipe:data-batch-generation/column-matching) is set to **"Custom"**
- The parameter "Custom grouping columns" in category "Merging iteration step generation" (jipipe:data-batch-generation/custom-matched-columns-expression) is set to **#Modality**

**Node #309 "Histogram plot" of type "Histogram plot"**

- Input "Input" of node #309 receives data from output "Output" of node #306
- The parameter "Series name" (series-name) is set to **"M1: negative RED vs. positive ORANGE in STED"**
- The parameter item #1 of "Overridden parameters" in category "Adaptive parameters" (jipipe:adaptive-parameters/overridden-parameters) is set to **(#Subfolder, "plot-parameters/title")**
- The parameter "Value" in category "Input columns" (input-columns/Value) is set to **("ExistingColumn", "Manders' tM1 (Above autothreshold of Ch2)")**
- The parameter "Bins" in category "Plot parameters" (plot-parameters/bins) is set to **20**
- The parameter "Bin axis label" in category "Plot parameters" (plot-parameters/bin-axis-label) is set to **"M1"**
- The parameter "Title" in category "Plot parameters" (plot-parameters/title) is set to **""**
- The parameter "Value axis label" in category "Plot parameters" (plot-parameters/value-axis-label) is set to **"Frequency"**
- The parameter "Color map" in category "Plot parameters" (plot-parameters/color-map) is set to **"Set1"**
- The parameter "Histogram type" in category "Plot parameters" (plot-parameters/histogram-type) is set to **"RelativeFrequency"**

**Node #310 "Histogram plot" of type "Histogram plot"**

- Input "Input" of node #310 receives data from output "Output" of node #306
- The parameter "Series name" (series-name) is set to **"M2: negative RED vs. positive ORANGE in STED"**
- The parameter item #1 of "Overridden parameters" in category "Adaptive parameters" (jipipe:adaptive-parameters/overridden-parameters) is set to **(#Subfolder, "plot-parameters/title")**
- The parameter "Value" in category "Input columns" (input-columns/Value) is set to **("ExistingColumn", "Manders' tM2 (Above autothreshold of Ch1)")**
- The parameter "Bins" in category "Plot parameters" (plot-parameters/bins) is set to **20**
- The parameter "Bin axis label" in category "Plot parameters" (plot-parameters/bin-axis-label) is set to **"M2"**
- The parameter "Title" in category "Plot parameters" (plot-parameters/title) is set to **""**
- The parameter "Value axis label" in category "Plot parameters" (plot-parameters/value-axis-label) is set to **"Frequency"**
- The parameter "Color map" in category "Plot parameters" (plot-parameters/color-map) is set to **"Set1"**
- The parameter "Histogram type" in category "Plot parameters" (plot-parameters/histogram-type) is set to **"RelativeFrequency"**

**Node #311 "Histogram plot" of type "Histogram plot"**

- Input "Input" of node #311 receives data from output "Output" of node #307
- The parameter "Series name" (series-name) is set to **"M2: RED vs. ORANGE in STED negative ROIs"**
- The parameter item #1 of "Overridden parameters" in category "Adaptive parameters" (jipipe:adaptive-parameters/overridden-parameters) is set to **(#Subfolder, "plot-parameters/title")**
- The parameter "Value" in category "Input columns" (input-columns/Value) is set to **("ExistingColumn", "Manders' tM2 (Above autothreshold of Ch1)")**
- The parameter "Bins" in category "Plot parameters" (plot-parameters/bins) is set to **20**
- The parameter "Bin axis label" in category "Plot parameters" (plot-parameters/bin-axis-label) is set to **"M2"**
- The parameter "Title" in category "Plot parameters" (plot-parameters/title) is set to **""**
- The parameter "Value axis label" in category "Plot parameters" (plot-parameters/value-axis-label) is set to **"Frequency"**
- The parameter "Color map" in category "Plot parameters" (plot-parameters/color-map) is set to **"Set1"**
- The parameter "Histogram type" in category "Plot parameters" (plot-parameters/histogram-type) is set to **"RelativeFrequency"**

**Node #312 "Histogram plot" of type "Histogram plot"**

- Input "Input" of node #312 receives data from output "Output" of node #307
- The parameter "Series name" (series-name) is set to **"M1: RED vs. ORANGE in STED negative ROIs"**
- The parameter item #1 of "Overridden parameters" in category "Adaptive parameters" (jipipe:adaptive-parameters/overridden-parameters) is set to **(#Subfolder, "plot-parameters/title")**
- The parameter "Value" in category "Input columns" (input-columns/Value) is set to **("ExistingColumn", "Manders' tM1 (Above autothreshold of Ch2)")**
- The parameter "Bins" in category "Plot parameters" (plot-parameters/bins) is set to **20**
- The parameter "Bin axis label" in category "Plot parameters" (plot-parameters/bin-axis-label) is set to **"M1"**
- The parameter "Title" in category "Plot parameters" (plot-parameters/title) is set to **""**
- The parameter "Value axis label" in category "Plot parameters" (plot-parameters/value-axis-label) is set to **"Frequency"**
- The parameter "Color map" in category "Plot parameters" (plot-parameters/color-map) is set to **"Set1"**
- The parameter "Histogram type" in category "Plot parameters" (plot-parameters/histogram-type) is set to **"RelativeFrequency"**

**Node #313 "Histogram plot" of type "Histogram plot"**

- Input "Input" of node #313 receives data from output "Output" of node #308
- The parameter "Series name" (series-name) is set to **"M1: positive RED vs. negative ORANGE in STED"**
- The parameter item #1 of "Overridden parameters" in category "Adaptive parameters" (jipipe:adaptive-parameters/overridden-parameters) is set to **(#Subfolder, "plot-parameters/title")**
- The parameter "Value" in category "Input columns" (input-columns/Value) is set to **("ExistingColumn", "Manders' tM1 (Above autothreshold of Ch2)")**
- The parameter "Bins" in category "Plot parameters" (plot-parameters/bins) is set to **20**
- The parameter "Bin axis label" in category "Plot parameters" (plot-parameters/bin-axis-label) is set to **"M1"**
- The parameter "Title" in category "Plot parameters" (plot-parameters/title) is set to **""**
- The parameter "Value axis label" in category "Plot parameters" (plot-parameters/value-axis-label) is set to **"Frequency"**
- The parameter "Color map" in category "Plot parameters" (plot-parameters/color-map) is set to **"Set1"**
- The parameter "Histogram type" in category "Plot parameters" (plot-parameters/histogram-type) is set to **"RelativeFrequency"**

**Node #314 "Histogram plot" of type "Histogram plot"**

- Input "Input" of node #314 receives data from output "Output" of node #308
- The parameter "Series name" (series-name) is set to **"M2: positive RED vs. negative ORANGE in STED"**
- The parameter item #1 of "Overridden parameters" in category "Adaptive parameters" (jipipe:adaptive-parameters/overridden-parameters) is set to **(#Subfolder, "plot-parameters/title")**
- The parameter "Value" in category "Input columns" (input-columns/Value) is set to **("ExistingColumn", "Manders' tM2 (Above autothreshold of Ch1)")**
- The parameter "Bins" in category "Plot parameters" (plot-parameters/bins) is set to **20**
- The parameter "Bin axis label" in category "Plot parameters" (plot-parameters/bin-axis-label) is set to **"M2"**
- The parameter "Title" in category "Plot parameters" (plot-parameters/title) is set to **""**
- The parameter "Value axis label" in category "Plot parameters" (plot-parameters/value-axis-label) is set to **"Frequency"**
- The parameter "Color map" in category "Plot parameters" (plot-parameters/color-map) is set to **"Set1"**
- The parameter "Histogram type" in category "Plot parameters" (plot-parameters/histogram-type) is set to **"RelativeFrequency"**

**Node #315 "Output" of type "Compartment output"**

- Input "Colocalization RED vs. ORANGE in STED negative ROIs" of node #315 receives data from output "Output" of node #307
- Input "Colocalization positive RED vs. negative ORANGE in STED" of node #315 receives data from output "Output" of node #308
- Input "Colocalization negative RED vs. positive ORANGE in STED" of node #315 receives data from output "Output" of node #306
- Input "Colocalization positive RED vs. positive ORANGE in STED" of node #315 receives data from output "Output" of node #278
- Input "Overlay of RED and ORANGE gaps" of node #315 receives data from output "Output" of node #303
- Input "Filament measurments RED STED continuous filaments" of node #315 receives data from output "Output" of node #292
- Input "Filament measurments RED STED filaments with gaps" of node #315 receives data from output "Output" of node #272
- Input "Filament measurments ORANGE STED continuous filaments" of node #315 receives data from output "Output" of node #300
- Input "Filament measurments ORANGE STED filaments with gaps" of node #315 receives data from output "Output" of node #275
- Input "Histo M2 RED vs. ORANGE in STED negative ROIs" of node #315 receives data from output "Output" of node #311
- Input "M1 RED vs. ORANGE in STED negative ROI" of node #315 receives data from output "Output" of node #312
- Input "M2 positive RED vs. negative ORANGE in STED" of node #315 receives data from output "Output" of node #314
- Input "M1 positive RED vs. negative ORANGE in STED" of node #315 receives data from output "Output" of node #310
- Input "M1 negative RED vs. positive ORANGE in STED" of node #315 receives data from output "Output" of node #309
- Input "M2 positive RED vs. positive ORANGE in STED" of node #315 receives data from output "Output" of node #282
- Input "M1 positive RED vs. positive ORANGE in STED" of node #315 receives data from output "Output" of node #283
- Input "Overlay of RED and ORANGE" of node #315 receives data from output "Output" of node #305
- The parameter "jipipe:compartment:output-slot-name" (jipipe:compartment:output-slot-name) is set to **"Output"**

## Compartment C5 "Colocalization"

- The "Colocalization" compartment (C5) receives data from the "Mask" compartment (C3)

**Node #217 "Masks" of type "Compartment output"**

- Input "Radii-corrected filaments" of node #217 receives data from output "Output" of node #164
- Input "Individual filaments radii-corrected" of node #217 receives data from output "Output" of node #211
- Input "Annotated images" of node #217 receives data from output "Annoated images" of node #37
- Input "Masks per image" of node #217 receives data from output "Output" of node #170
- Input "Masks per filament" of node #217 receives data from output "Output" of node #175
- Input "Overlay mask with raw merged STED" of node #217 receives data from output "Output" of node #198
- Input "Filaments radius 1" of node #217 receives data from output "Output" of node #161
- Input "Mask from filament radius 1" of node #217 receives data from output "Output" of node #165
- Input "Mask from filament radius 1 per filament" of node #217 receives data from output "Output" of node #212
- The parameter "jipipe:compartment:output-slot-name" (jipipe:compartment:output-slot-name) is set to **"Masks"**

**Node #316 "Split & filter by annotation" of type "Split & filter by annotation"**

- Input "Input" of node #316 receives data from output "Annotated images" of node #217
- The parameter "Output" in category "Filters" (target-slots/Output) is set to **#Label CONTAINS "488"**
- The parameter "Output 1" in category "Filters" (target-slots/Output 1) is set to **#Label CONTAINS "594"**

**Node #317 "Filter by annotation (If else): green" of type "Filter by annotation (If else)"**

- Input "Input" of node #317 receives data from output "Output" of node #316
- The parameter "Filter" (filter) is set to **#Modality == "Conf"**

**Node #318 "Filter by annotation (If else): magenta" of type "Filter by annotation (If else)"**

- Input "Input" of node #318 receives data from output "Output 1" of node #316
- The parameter "Filter" (filter) is set to **#Modality == "Conf"**

**Node #319 "Divide by maximum" of type "Divide by maximum"**

- Input "Input" of node #319 receives data from output "Matched" of node #317

**Node #320 "Divide by maximum" of type "Divide by maximum"**

- Input "Input" of node #320 receives data from output "Unmatched" of node #317

**Node #321 "Divide by maximum" of type "Divide by maximum"**

- Input "Input" of node #321 receives data from output "Matched" of node #318

**Node #322 "Divide by maximum" of type "Divide by maximum"**

- Input "Input" of node #322 receives data from output "Unmatched" of node #318

**Node #323 "Coloc 2: CONF vs. STED in Green" of type "Coloc 2"**

- Input "Channel 1" of node #323 receives data from output "Output" of node #319
- Input "Channel 2" of node #323 receives data from output "Output" of node #320
- Input "Mask" of node #323 receives data from output "Masks per image" of node #217
- The parameter "Restrict to 2D ROI/mask" (input-masks) is set to **true**
- The parameter "Channel 2 name" (channel2-name) is set to **"STED"**
- The parameter "Channel 1 name" (channel1-name) is set to **"CONF"**
- The parameter "Grouping method" in category "Input management" (jipipe:data-batch-generation/column-matching) is set to **"Custom"**
- The parameter "Custom grouping columns" in category "Input management" (jipipe:data-batch-generation/custom-matched-columns-expression) is set to **#Image**
- The parameter "Threshold regression" in category "Colocalization settings" (coloc2-settings/threshold-regression) is set to **"Bisection"**

**Node #324 "Coloc 2: CONF vs. STED in Green" of type "Coloc 2"**

- Input "Channel 1" of node #324 receives data from output "Output" of node #319
- Input "Channel 2" of node #324 receives data from output "Output" of node #320
- Input "Mask" of node #324 receives data from output "Masks per filament" of node #217
- The parameter "Restrict to 2D ROI/mask" (input-masks) is set to **true**
- The parameter "Channel 2 name" (channel2-name) is set to **"STED"**
- The parameter "Channel 1 name" (channel1-name) is set to **"CONF"**
- The parameter "Grouping method" in category "Input management" (jipipe:data-batch-generation/column-matching) is set to **"Custom"**
- The parameter "Custom grouping columns" in category "Input management" (jipipe:data-batch-generation/custom-matched-columns-expression) is set to **#Image**
- The parameter "Threshold regression" in category "Colocalization settings" (coloc2-settings/threshold-regression) is set to **"Bisection"**

**Node #325 "Coloc 2: Green vs. Magenta in Conf" of type "Coloc 2"**

- Input "Channel 1" of node #325 receives data from output "Output" of node #319
- Input "Channel 2" of node #325 receives data from output "Output" of node #321
- Input "Mask" of node #325 receives data from output "Masks per filament" of node #217
- The parameter "Restrict to 2D ROI/mask" (input-masks) is set to **true**
- The parameter "Channel 2 name" (channel2-name) is set to **"ORANGE"**
- The parameter "Channel 1 name" (channel1-name) is set to **"RED"**
- The parameter "Grouping method" in category "Input management" (jipipe:data-batch-generation/column-matching) is set to **"Custom"**
- The parameter "Custom grouping columns" in category "Input management" (jipipe:data-batch-generation/custom-matched-columns-expression) is set to **#Image**
- The parameter "Skip incomplete data sets" in category "Input management" (jipipe:data-batch-generation/skip-incomplete) is set to **true**

**Node #326 "Coloc 2: Green vs. Magenta in Conf" of type "Coloc 2"**

- Input "Channel 1" of node #326 receives data from output "Output" of node #319
- Input "Channel 2" of node #326 receives data from output "Output" of node #321
- Input "Mask" of node #326 receives data from output "Masks per image" of node #217
- The parameter "Restrict to 2D ROI/mask" (input-masks) is set to **true**
- The parameter "Channel 2 name" (channel2-name) is set to **"ORANGE"**
- The parameter "Channel 1 name" (channel1-name) is set to **"RED"**
- The parameter "Grouping method" in category "Input management" (jipipe:data-batch-generation/column-matching) is set to **"Custom"**
- The parameter "Custom grouping columns" in category "Input management" (jipipe:data-batch-generation/custom-matched-columns-expression) is set to **#Image**
- The parameter "Skip incomplete data sets" in category "Input management" (jipipe:data-batch-generation/skip-incomplete) is set to **true**

**Node #327 "Coloc 2: CONF vs. STED in Magenta" of type "Coloc 2"**

- Input "Channel 1" of node #327 receives data from output "Output" of node #321
- Input "Channel 2" of node #327 receives data from output "Output" of node #322
- Input "Mask" of node #327 receives data from output "Masks per image" of node #217
- The parameter "Restrict to 2D ROI/mask" (input-masks) is set to **true**
- The parameter "Channel 2 name" (channel2-name) is set to **"STED"**
- The parameter "Channel 1 name" (channel1-name) is set to **"CONF"**
- The parameter "Grouping method" in category "Input management" (jipipe:data-batch-generation/column-matching) is set to **"Custom"**
- The parameter "Custom grouping columns" in category "Input management" (jipipe:data-batch-generation/custom-matched-columns-expression) is set to **#Image**
- The parameter "Skip incomplete data sets" in category "Input management" (jipipe:data-batch-generation/skip-incomplete) is set to **true**

**Node #328 "Coloc 2: CONF vs. STED in Magenta" of type "Coloc 2"**

- Input "Channel 1" of node #328 receives data from output "Output" of node #321
- Input "Channel 2" of node #328 receives data from output "Output" of node #322
- Input "Mask" of node #328 receives data from output "Masks per filament" of node #217
- The parameter "Restrict to 2D ROI/mask" (input-masks) is set to **true**
- The parameter "Channel 2 name" (channel2-name) is set to **"STED"**
- The parameter "Channel 1 name" (channel1-name) is set to **"CONF"**
- The parameter "Grouping method" in category "Input management" (jipipe:data-batch-generation/column-matching) is set to **"Custom"**
- The parameter "Custom grouping columns" in category "Input management" (jipipe:data-batch-generation/custom-matched-columns-expression) is set to **#Image**
- The parameter "Skip incomplete data sets" in category "Input management" (jipipe:data-batch-generation/skip-incomplete) is set to **true**

**Node #329 "Coloc 2: Green vs. Magenta in STED" of type "Coloc 2"**

- Input "Channel 1" of node #329 receives data from output "Output" of node #320
- Input "Channel 2" of node #329 receives data from output "Output" of node #322
- Input "Mask" of node #329 receives data from output "Masks per image" of node #217
- The parameter "Restrict to 2D ROI/mask" (input-masks) is set to **true**
- The parameter "Channel 2 name" (channel2-name) is set to **"ORANGE"**
- The parameter "Channel 1 name" (channel1-name) is set to **"RED"**
- The parameter "Grouping method" in category "Input management" (jipipe:data-batch-generation/column-matching) is set to **"Custom"**
- The parameter "Custom grouping columns" in category "Input management" (jipipe:data-batch-generation/custom-matched-columns-expression) is set to **#Image**
- The parameter "Skip incomplete data sets" in category "Input management" (jipipe:data-batch-generation/skip-incomplete) is set to **true**
- The parameter "Li Histogram Channel 2" in category "Colocalization settings" (coloc2-settings/li-histogram-channel-2) is set to **false**
- The parameter "Li Histogram Channel 1" in category "Colocalization settings" (coloc2-settings/li-histogram-channel-1) is set to **false**
- The parameter "Costes' Significance Test" in category "Colocalization settings" (coloc2-settings/costes-significance-test) is set to **false**
- The parameter "Kendall's Tau Rank Correlation" in category "Colocalization settings" (coloc2-settings/kendall-tau-rank-correlation) is set to **false**
- The parameter "Spearman's Rank Correlation" in category "Colocalization settings" (coloc2-settings/spearman-rank-correlation) is set to **false**
- The parameter "2D Intensity Histogram" in category "Colocalization settings" (coloc2-settings/2d-intensity-histogram) is set to **false**
- The parameter "Li ICQ" in category "Colocalization settings" (coloc2-settings/li-icq) is set to **false**

**Node #330 "Coloc 2: Green vs. Magenta in STED" of type "Coloc 2"**

- Input "Channel 1" of node #330 receives data from output "Output" of node #320
- Input "Channel 2" of node #330 receives data from output "Output" of node #322
- Input "Mask" of node #330 receives data from output "Masks per filament" of node #217
- The parameter "Restrict to 2D ROI/mask" (input-masks) is set to **true**
- The parameter "Channel 2 name" (channel2-name) is set to **"ORANGE"**
- The parameter "Channel 1 name" (channel1-name) is set to **"RED"**
- The parameter "Grouping method" in category "Input management" (jipipe:data-batch-generation/column-matching) is set to **"Custom"**
- The parameter "Custom grouping columns" in category "Input management" (jipipe:data-batch-generation/custom-matched-columns-expression) is set to **#Image**
- The parameter "Skip incomplete data sets" in category "Input management" (jipipe:data-batch-generation/skip-incomplete) is set to **true**
- The parameter "Li Histogram Channel 2" in category "Colocalization settings" (coloc2-settings/li-histogram-channel-2) is set to **false**
- The parameter "Li Histogram Channel 1" in category "Colocalization settings" (coloc2-settings/li-histogram-channel-1) is set to **false**
- The parameter "Costes' Significance Test" in category "Colocalization settings" (coloc2-settings/costes-significance-test) is set to **false**
- The parameter "Kendall's Tau Rank Correlation" in category "Colocalization settings" (coloc2-settings/kendall-tau-rank-correlation) is set to **false**
- The parameter "Spearman's Rank Correlation" in category "Colocalization settings" (coloc2-settings/spearman-rank-correlation) is set to **false**
- The parameter "2D Intensity Histogram" in category "Colocalization settings" (coloc2-settings/2d-intensity-histogram) is set to **false**
- The parameter "Li ICQ" in category "Colocalization settings" (coloc2-settings/li-icq) is set to **false**

**Node #331 "Add annotations as columns" of type "Add annotations as columns"**

- Input "Input" of node #331 receives data from output "Results" of node #323
- The parameter "Annotation name filter" (annotation-name-filter) is set to **#Image**

**Node #332 "Add annotations as columns" of type "Add annotations as columns"**

- Input "Input" of node #332 receives data from output "Results" of node #324
- The parameter "Annotation name filter" (annotation-name-filter) is set to **#Image**

**Node #333 "Add annotations as columns" of type "Add annotations as columns"**

- Input "Input" of node #333 receives data from output "Results" of node #325
- The parameter "Annotation name filter" (annotation-name-filter) is set to **#Image**

**Node #334 "Add annotations as columns" of type "Add annotations as columns"**

- Input "Input" of node #334 receives data from output "Results" of node #326
- The parameter "Annotation name filter" (annotation-name-filter) is set to **#Image**

**Node #335 "Add annotations as columns" of type "Add annotations as columns"**

- Input "Input" of node #335 receives data from output "Results" of node #327
- The parameter "Annotation name filter" (annotation-name-filter) is set to **#Image**

**Node #336 "Add annotations as columns" of type "Add annotations as columns"**

- Input "Input" of node #336 receives data from output "Results" of node #328
- The parameter "Annotation name filter" (annotation-name-filter) is set to **#Image**

**Node #337 "Add annotations as columns" of type "Add annotations as columns"**

- Input "Input" of node #337 receives data from output "Results" of node #329
- The parameter "Annotation name filter" (annotation-name-filter) is set to **#Image**

**Node #338 "Add annotations as columns" of type "Add annotations as columns"**

- Input "Input" of node #338 receives data from output "Results" of node #330
- The parameter "Annotation name filter" (annotation-name-filter) is set to **#Image**

**Node #339 "Merge table rows" of type "Merge table rows"**

- Input "Input" of node #339 receives data from output "Output" of node #331
- The parameter "Grouping method" in category "Merging iteration step generation" (jipipe:data-batch-generation/column-matching) is set to **"Custom"**
- The parameter "Custom grouping columns" in category "Merging iteration step generation" (jipipe:data-batch-generation/custom-matched-columns-expression) is set to **#Label**

**Node #340 "Merge table rows" of type "Merge table rows"**

- Input "Input" of node #340 receives data from output "Output" of node #332
- The parameter "Grouping method" in category "Merging iteration step generation" (jipipe:data-batch-generation/column-matching) is set to **"Custom"**
- The parameter "Custom grouping columns" in category "Merging iteration step generation" (jipipe:data-batch-generation/custom-matched-columns-expression) is set to **#Label**

**Node #341 "Merge table rows" of type "Merge table rows"**

- Input "Input" of node #341 receives data from output "Output" of node #333
- The parameter "Grouping method" in category "Merging iteration step generation" (jipipe:data-batch-generation/column-matching) is set to **"Custom"**
- The parameter "Custom grouping columns" in category "Merging iteration step generation" (jipipe:data-batch-generation/custom-matched-columns-expression) is set to **#Modality**
- The parameter "Skip incomplete data sets" in category "Merging iteration step generation" (jipipe:data-batch-generation/skip-incomplete) is set to **true**

**Node #342 "Merge table rows" of type "Merge table rows"**

- Input "Input" of node #342 receives data from output "Output" of node #334
- The parameter "Grouping method" in category "Merging iteration step generation" (jipipe:data-batch-generation/column-matching) is set to **"Custom"**
- The parameter "Custom grouping columns" in category "Merging iteration step generation" (jipipe:data-batch-generation/custom-matched-columns-expression) is set to **#Modality**
- The parameter "Skip incomplete data sets" in category "Merging iteration step generation" (jipipe:data-batch-generation/skip-incomplete) is set to **true**

**Node #343 "Merge table rows" of type "Merge table rows"**

- Input "Input" of node #343 receives data from output "Output" of node #335
- The parameter "Grouping method" in category "Merging iteration step generation" (jipipe:data-batch-generation/column-matching) is set to **"Custom"**
- The parameter "Custom grouping columns" in category "Merging iteration step generation" (jipipe:data-batch-generation/custom-matched-columns-expression) is set to **#Label**

**Node #344 "Merge table rows" of type "Merge table rows"**

- Input "Input" of node #344 receives data from output "Output" of node #336
- The parameter "Grouping method" in category "Merging iteration step generation" (jipipe:data-batch-generation/column-matching) is set to **"Custom"**
- The parameter "Custom grouping columns" in category "Merging iteration step generation" (jipipe:data-batch-generation/custom-matched-columns-expression) is set to **#Label**

**Node #345 "Merge table rows" of type "Merge table rows"**

- Input "Input" of node #345 receives data from output "Output" of node #337
- The parameter "Grouping method" in category "Merging iteration step generation" (jipipe:data-batch-generation/column-matching) is set to **"Custom"**
- The parameter "Custom grouping columns" in category "Merging iteration step generation" (jipipe:data-batch-generation/custom-matched-columns-expression) is set to **#Modality**
- The parameter "Skip incomplete data sets" in category "Merging iteration step generation" (jipipe:data-batch-generation/skip-incomplete) is set to **true**

**Node #346 "Merge table rows" of type "Merge table rows"**

- Input "Input" of node #346 receives data from output "Output" of node #338
- The parameter "Grouping method" in category "Merging iteration step generation" (jipipe:data-batch-generation/column-matching) is set to **"Custom"**
- The parameter "Custom grouping columns" in category "Merging iteration step generation" (jipipe:data-batch-generation/custom-matched-columns-expression) is set to **#Modality**
- The parameter "Skip incomplete data sets" in category "Merging iteration step generation" (jipipe:data-batch-generation/skip-incomplete) is set to **true**

**Node #347 "Histogram plot" of type "Histogram plot"**

- Input "Input" of node #347 receives data from output "Output" of node #339
- The parameter "Series name" (series-name) is set to **"M2: CONF vs. STED in RED"**
- The parameter item #1 of "Overridden parameters" in category "Adaptive parameters" (jipipe:adaptive-parameters/overridden-parameters) is set to **(#Subfolder, "plot-parameters/legend")**
- The parameter "Value" in category "Input columns" (input-columns/Value) is set to **("ExistingColumn", "Manders' tM2 (Above autothreshold of Ch1)")**
- The parameter "Bins" in category "Plot parameters" (plot-parameters/bins) is set to **20**
- The parameter "Bin axis label" in category "Plot parameters" (plot-parameters/bin-axis-label) is set to **"M2"**
- The parameter "Title" in category "Plot parameters" (plot-parameters/title) is set to **"Manders' tM2 CONF vs. STED in green"**
- The parameter "Value axis label" in category "Plot parameters" (plot-parameters/value-axis-label) is set to **"Frequency"**
- The parameter "Color map" in category "Plot parameters" (plot-parameters/color-map) is set to **"Set1"**
- The parameter "Histogram type" in category "Plot parameters" (plot-parameters/histogram-type) is set to **"RelativeFrequency"**

**Node #348 "Histogram plot" of type "Histogram plot"**

- Input "Input" of node #348 receives data from output "Output" of node #339
- The parameter "Series name" (series-name) is set to **"M1: CONF vs. STED in RED"**
- The parameter item #1 of "Overridden parameters" in category "Adaptive parameters" (jipipe:adaptive-parameters/overridden-parameters) is set to **(#Subfolder, "plot-parameters/legend")**
- The parameter "Value" in category "Input columns" (input-columns/Value) is set to **("ExistingColumn", "Manders' tM1 (Above autothreshold of Ch2)")**
- The parameter "Bins" in category "Plot parameters" (plot-parameters/bins) is set to **20**
- The parameter "Bin axis label" in category "Plot parameters" (plot-parameters/bin-axis-label) is set to **"M2"**
- The parameter "Title" in category "Plot parameters" (plot-parameters/title) is set to **"Manders' tM1 CONF vs. STED in green"**
- The parameter "Value axis label" in category "Plot parameters" (plot-parameters/value-axis-label) is set to **"Frequency"**
- The parameter "Color map" in category "Plot parameters" (plot-parameters/color-map) is set to **"Set1"**
- The parameter "Histogram type" in category "Plot parameters" (plot-parameters/histogram-type) is set to **"RelativeFrequency"**

**Node #349 "Histogram plot" of type "Histogram plot"**

- Input "Input" of node #349 receives data from output "Output" of node #340
- The parameter "Series name" (series-name) is set to **"M1: CONF vs. STED in RED"**
- The parameter item #1 of "Overridden parameters" in category "Adaptive parameters" (jipipe:adaptive-parameters/overridden-parameters) is set to **(#Subfolder, "plot-parameters/legend")**
- The parameter "Value" in category "Input columns" (input-columns/Value) is set to **("ExistingColumn", "Manders' tM1 (Above autothreshold of Ch2)")**
- The parameter "Bins" in category "Plot parameters" (plot-parameters/bins) is set to **20**
- The parameter "Bin axis label" in category "Plot parameters" (plot-parameters/bin-axis-label) is set to **"M2"**
- The parameter "Title" in category "Plot parameters" (plot-parameters/title) is set to **"Manders' tM1 CONF vs. STED in green"**
- The parameter "Value axis label" in category "Plot parameters" (plot-parameters/value-axis-label) is set to **"Frequency"**
- The parameter "Color map" in category "Plot parameters" (plot-parameters/color-map) is set to **"Set1"**
- The parameter "Histogram type" in category "Plot parameters" (plot-parameters/histogram-type) is set to **"RelativeFrequency"**

**Node #350 "Histogram plot" of type "Histogram plot"**

- Input "Input" of node #350 receives data from output "Output" of node #340
- The parameter "Series name" (series-name) is set to **"M2: CONF vs. STED in RED"**
- The parameter item #1 of "Overridden parameters" in category "Adaptive parameters" (jipipe:adaptive-parameters/overridden-parameters) is set to **(#Subfolder, "plot-parameters/legend")**
- The parameter "Value" in category "Input columns" (input-columns/Value) is set to **("ExistingColumn", "Manders' tM2 (Above autothreshold of Ch1)")**
- The parameter "Bins" in category "Plot parameters" (plot-parameters/bins) is set to **20**
- The parameter "Bin axis label" in category "Plot parameters" (plot-parameters/bin-axis-label) is set to **"M2"**
- The parameter "Title" in category "Plot parameters" (plot-parameters/title) is set to **"Manders' tM2 CONF vs. STED in green"**
- The parameter "Value axis label" in category "Plot parameters" (plot-parameters/value-axis-label) is set to **"Frequency"**
- The parameter "Color map" in category "Plot parameters" (plot-parameters/color-map) is set to **"Set1"**
- The parameter "Histogram type" in category "Plot parameters" (plot-parameters/histogram-type) is set to **"RelativeFrequency"**

**Node #351 "Histogram plot" of type "Histogram plot"**

- Input "Input" of node #351 receives data from output "Output" of node #341
- The parameter "Series name" (series-name) is set to **"M1: RED vs. ORANGE in CONF"**
- The parameter item #1 of "Overridden parameters" in category "Adaptive parameters" (jipipe:adaptive-parameters/overridden-parameters) is set to **(#Subfolder, "plot-parameters/legend")**
- The parameter "Value" in category "Input columns" (input-columns/Value) is set to **("ExistingColumn", "Manders' tM1 (Above autothreshold of Ch2)")**
- The parameter "Bins" in category "Plot parameters" (plot-parameters/bins) is set to **20**
- The parameter "Bin axis label" in category "Plot parameters" (plot-parameters/bin-axis-label) is set to **"M2"**
- The parameter "Title" in category "Plot parameters" (plot-parameters/title) is set to **"Manders' tM1 green vs. magenta in CONF"**
- The parameter "Value axis label" in category "Plot parameters" (plot-parameters/value-axis-label) is set to **"Frequency"**
- The parameter "Color map" in category "Plot parameters" (plot-parameters/color-map) is set to **"Set1"**
- The parameter "Histogram type" in category "Plot parameters" (plot-parameters/histogram-type) is set to **"RelativeFrequency"**

**Node #352 "Histogram plot" of type "Histogram plot"**

- Input "Input" of node #352 receives data from output "Output" of node #341
- The parameter "Series name" (series-name) is set to **"M2: RED vs. ORANGE in CONF"**
- The parameter item #1 of "Overridden parameters" in category "Adaptive parameters" (jipipe:adaptive-parameters/overridden-parameters) is set to **(#Subfolder, "plot-parameters/legend")**
- The parameter "Value" in category "Input columns" (input-columns/Value) is set to **("ExistingColumn", "Manders' tM2 (Above autothreshold of Ch1)")**
- The parameter "Bins" in category "Plot parameters" (plot-parameters/bins) is set to **20**
- The parameter "Bin axis label" in category "Plot parameters" (plot-parameters/bin-axis-label) is set to **"M2"**
- The parameter "Title" in category "Plot parameters" (plot-parameters/title) is set to **"Manders' tM2 green vs. magenta in CONF\""**
- The parameter "Value axis label" in category "Plot parameters" (plot-parameters/value-axis-label) is set to **"Frequency"**
- The parameter "Color map" in category "Plot parameters" (plot-parameters/color-map) is set to **"Set1"**
- The parameter "Histogram type" in category "Plot parameters" (plot-parameters/histogram-type) is set to **"RelativeFrequency"**

**Node #353 "Histogram plot" of type "Histogram plot"**

- Input "Input" of node #353 receives data from output "Output" of node #342
- The parameter "Series name" (series-name) is set to **"M2: RED vs. ORANGE in CONF"**
- The parameter item #1 of "Overridden parameters" in category "Adaptive parameters" (jipipe:adaptive-parameters/overridden-parameters) is set to **(#Subfolder, "plot-parameters/legend")**
- The parameter "Value" in category "Input columns" (input-columns/Value) is set to **("ExistingColumn", "Manders' tM2 (Above autothreshold of Ch1)")**
- The parameter "Bins" in category "Plot parameters" (plot-parameters/bins) is set to **20**
- The parameter "Bin axis label" in category "Plot parameters" (plot-parameters/bin-axis-label) is set to **"M2"**
- The parameter "Title" in category "Plot parameters" (plot-parameters/title) is set to **"Manders' tM2 green vs. magenta in CONF\""**
- The parameter "Value axis label" in category "Plot parameters" (plot-parameters/value-axis-label) is set to **"Frequency"**
- The parameter "Color map" in category "Plot parameters" (plot-parameters/color-map) is set to **"Set1"**
- The parameter "Histogram type" in category "Plot parameters" (plot-parameters/histogram-type) is set to **"RelativeFrequency"**

**Node #354 "Histogram plot" of type "Histogram plot"**

- Input "Input" of node #354 receives data from output "Output" of node #342
- The parameter "Series name" (series-name) is set to **"M1: RED vs. ORANGE in CONF"**
- The parameter item #1 of "Overridden parameters" in category "Adaptive parameters" (jipipe:adaptive-parameters/overridden-parameters) is set to **(#Subfolder, "plot-parameters/legend")**
- The parameter "Value" in category "Input columns" (input-columns/Value) is set to **("ExistingColumn", "Manders' tM1 (Above autothreshold of Ch2)")**
- The parameter "Bins" in category "Plot parameters" (plot-parameters/bins) is set to **20**
- The parameter "Bin axis label" in category "Plot parameters" (plot-parameters/bin-axis-label) is set to **"M2"**
- The parameter "Title" in category "Plot parameters" (plot-parameters/title) is set to **"Manders' tM1 green vs. magenta in CONF"**
- The parameter "Value axis label" in category "Plot parameters" (plot-parameters/value-axis-label) is set to **"Frequency"**
- The parameter "Color map" in category "Plot parameters" (plot-parameters/color-map) is set to **"Set1"**
- The parameter "Histogram type" in category "Plot parameters" (plot-parameters/histogram-type) is set to **"RelativeFrequency"**

**Node #355 "Histogram plot" of type "Histogram plot"**

- Input "Input" of node #355 receives data from output "Output" of node #343
- The parameter "Series name" (series-name) is set to **"M2: CONF vs. STED in ORANGE"**
- The parameter item #1 of "Overridden parameters" in category "Adaptive parameters" (jipipe:adaptive-parameters/overridden-parameters) is set to **(#Subfolder, "plot-parameters/legend")**
- The parameter "Value" in category "Input columns" (input-columns/Value) is set to **("ExistingColumn", "Manders' tM2 (Above autothreshold of Ch1)")**
- The parameter "Bins" in category "Plot parameters" (plot-parameters/bins) is set to **20**
- The parameter "Bin axis label" in category "Plot parameters" (plot-parameters/bin-axis-label) is set to **"M2"**
- The parameter "Title" in category "Plot parameters" (plot-parameters/title) is set to **"Manders' tM2 CONF vs. STED in magenta"**
- The parameter "Value axis label" in category "Plot parameters" (plot-parameters/value-axis-label) is set to **"Frequency"**
- The parameter "Color map" in category "Plot parameters" (plot-parameters/color-map) is set to **"Set1"**
- The parameter "Histogram type" in category "Plot parameters" (plot-parameters/histogram-type) is set to **"RelativeFrequency"**

**Node #356 "Histogram plot" of type "Histogram plot"**

- Input "Input" of node #356 receives data from output "Output" of node #343
- The parameter "Series name" (series-name) is set to **"M1: CONF vs. STED in ORANGE"**
- The parameter item #1 of "Overridden parameters" in category "Adaptive parameters" (jipipe:adaptive-parameters/overridden-parameters) is set to **(#Subfolder, "plot-parameters/legend")**
- The parameter "Value" in category "Input columns" (input-columns/Value) is set to **("ExistingColumn", "Manders' tM1 (Above autothreshold of Ch2)")**
- The parameter "Bins" in category "Plot parameters" (plot-parameters/bins) is set to **20**
- The parameter "Bin axis label" in category "Plot parameters" (plot-parameters/bin-axis-label) is set to **"M2"**
- The parameter "Title" in category "Plot parameters" (plot-parameters/title) is set to **"Manders' tM1CONF vs. STED in magenta"**
- The parameter "Value axis label" in category "Plot parameters" (plot-parameters/value-axis-label) is set to **"Frequency"**
- The parameter "Color map" in category "Plot parameters" (plot-parameters/color-map) is set to **"Set1"**
- The parameter "Histogram type" in category "Plot parameters" (plot-parameters/histogram-type) is set to **"RelativeFrequency"**

**Node #357 "Histogram plot" of type "Histogram plot"**

- Input "Input" of node #357 receives data from output "Output" of node #344
- The parameter "Series name" (series-name) is set to **"M1: CONF vs. STED in ORANGE"**
- The parameter item #1 of "Overridden parameters" in category "Adaptive parameters" (jipipe:adaptive-parameters/overridden-parameters) is set to **(#Subfolder, "plot-parameters/legend")**
- The parameter "Value" in category "Input columns" (input-columns/Value) is set to **("ExistingColumn", "Manders' tM1 (Above autothreshold of Ch2)")**
- The parameter "Bins" in category "Plot parameters" (plot-parameters/bins) is set to **20**
- The parameter "Bin axis label" in category "Plot parameters" (plot-parameters/bin-axis-label) is set to **"M2"**
- The parameter "Title" in category "Plot parameters" (plot-parameters/title) is set to **"Manders' tM1CONF vs. STED in magenta"**
- The parameter "Value axis label" in category "Plot parameters" (plot-parameters/value-axis-label) is set to **"Frequency"**
- The parameter "Color map" in category "Plot parameters" (plot-parameters/color-map) is set to **"Set1"**
- The parameter "Histogram type" in category "Plot parameters" (plot-parameters/histogram-type) is set to **"RelativeFrequency"**

**Node #358 "Histogram plot" of type "Histogram plot"**

- Input "Input" of node #358 receives data from output "Output" of node #344
- The parameter "Series name" (series-name) is set to **"M2: CONF vs. STED in ORANGE"**
- The parameter item #1 of "Overridden parameters" in category "Adaptive parameters" (jipipe:adaptive-parameters/overridden-parameters) is set to **(#Subfolder, "plot-parameters/legend")**
- The parameter "Value" in category "Input columns" (input-columns/Value) is set to **("ExistingColumn", "Manders' tM2 (Above autothreshold of Ch1)")**
- The parameter "Bins" in category "Plot parameters" (plot-parameters/bins) is set to **20**
- The parameter "Bin axis label" in category "Plot parameters" (plot-parameters/bin-axis-label) is set to **"M2"**
- The parameter "Title" in category "Plot parameters" (plot-parameters/title) is set to **"Manders' tM2 CONF vs. STED in magenta"**
- The parameter "Value axis label" in category "Plot parameters" (plot-parameters/value-axis-label) is set to **"Frequency"**
- The parameter "Color map" in category "Plot parameters" (plot-parameters/color-map) is set to **"Set1"**
- The parameter "Histogram type" in category "Plot parameters" (plot-parameters/histogram-type) is set to **"RelativeFrequency"**

**Node #359 "Colocalization per image" of type "Compartment output"**

- Input "CONF vs STED, green per image" of node #359 receives data from output "Output" of node #339
- Input "CONF vs STED, magenta per image" of node #359 receives data from output "Output" of node #343
- Input "Green vs Magenta, CONF per image" of node #359 receives data from output "Output" of node #342
- Input "Green vs magenta, STED per image" of node #359 receives data from output "Output" of node #345
- The parameter "jipipe:compartment:output-slot-name" (jipipe:compartment:output-slot-name) is set to **"Colocalization per image"**

**Node #360 "Histogram plot" of type "Histogram plot"**

- Input "Input" of node #360 receives data from output "Output" of node #345
- The parameter "Series name" (series-name) is set to **"M1: RED vs. ORANGE in STED"**
- The parameter item #1 of "Overridden parameters" in category "Adaptive parameters" (jipipe:adaptive-parameters/overridden-parameters) is set to **(#Subfolder, "plot-parameters/legend")**
- The parameter "Value" in category "Input columns" (input-columns/Value) is set to **("ExistingColumn", "Manders' tM1 (Above autothreshold of Ch2)")**
- The parameter "Bins" in category "Plot parameters" (plot-parameters/bins) is set to **20**
- The parameter "Bin axis label" in category "Plot parameters" (plot-parameters/bin-axis-label) is set to **"M2"**
- The parameter "Title" in category "Plot parameters" (plot-parameters/title) is set to **"Manders' tM1 green vs. magenta in STED"**
- The parameter "Value axis label" in category "Plot parameters" (plot-parameters/value-axis-label) is set to **"Frequency"**
- The parameter "Color map" in category "Plot parameters" (plot-parameters/color-map) is set to **"Set1"**
- The parameter "Histogram type" in category "Plot parameters" (plot-parameters/histogram-type) is set to **"RelativeFrequency"**

**Node #361 "Histogram plot" of type "Histogram plot"**

- Input "Input" of node #361 receives data from output "Output" of node #345
- The parameter "Series name" (series-name) is set to **"M2: RED vs. ORANGE in STED"**
- The parameter item #1 of "Overridden parameters" in category "Adaptive parameters" (jipipe:adaptive-parameters/overridden-parameters) is set to **(#Subfolder, "plot-parameters/legend")**
- The parameter "Value" in category "Input columns" (input-columns/Value) is set to **("ExistingColumn", "Manders' tM2 (Above autothreshold of Ch1)")**
- The parameter "Bins" in category "Plot parameters" (plot-parameters/bins) is set to **20**
- The parameter "Bin axis label" in category "Plot parameters" (plot-parameters/bin-axis-label) is set to **"M2"**
- The parameter "Title" in category "Plot parameters" (plot-parameters/title) is set to **"Manders' tM2 green vs. magenta in STED"**
- The parameter "Value axis label" in category "Plot parameters" (plot-parameters/value-axis-label) is set to **"Frequency"**
- The parameter "Color map" in category "Plot parameters" (plot-parameters/color-map) is set to **"Set1"**
- The parameter "Histogram type" in category "Plot parameters" (plot-parameters/histogram-type) is set to **"RelativeFrequency"**

**Node #362 "Histogram plot" of type "Histogram plot"**

- Input "Input" of node #362 receives data from output "Output" of node #346
- The parameter "Series name" (series-name) is set to **"M1: RED vs. ORANGE in STED"**
- The parameter item #1 of "Overridden parameters" in category "Adaptive parameters" (jipipe:adaptive-parameters/overridden-parameters) is set to **(#Subfolder, "plot-parameters/legend")**
- The parameter "Value" in category "Input columns" (input-columns/Value) is set to **("ExistingColumn", "Manders' tM1 (Above autothreshold of Ch2)")**
- The parameter "Bins" in category "Plot parameters" (plot-parameters/bins) is set to **20**
- The parameter "Bin axis label" in category "Plot parameters" (plot-parameters/bin-axis-label) is set to **"M2"**
- The parameter "Title" in category "Plot parameters" (plot-parameters/title) is set to **"Manders' tM1 green vs. magenta in STED"**
- The parameter "Value axis label" in category "Plot parameters" (plot-parameters/value-axis-label) is set to **"Frequency"**
- The parameter "Color map" in category "Plot parameters" (plot-parameters/color-map) is set to **"Set1"**
- The parameter "Histogram type" in category "Plot parameters" (plot-parameters/histogram-type) is set to **"RelativeFrequency"**

**Node #363 "Histogram plot" of type "Histogram plot"**

- Input "Input" of node #363 receives data from output "Output" of node #346
- The parameter "Series name" (series-name) is set to **#Folder**
- The parameter item #1 of "Overridden parameters" in category "Adaptive parameters" (jipipe:adaptive-parameters/overridden-parameters) is set to **(#Subfolder, "plot-parameters/legend")**
- The parameter "Value" in category "Input columns" (input-columns/Value) is set to **("ExistingColumn", "Pearson's R value (no threshold)")**
- The parameter "Bins" in category "Plot parameters" (plot-parameters/bins) is set to **20**
- The parameter "Title font size" in category "Plot parameters" (plot-parameters/title-font-size) is set to **20**
- The parameter "Bin axis label" in category "Plot parameters" (plot-parameters/bin-axis-label) is set to **"Pearson's R"**
- The parameter "Bin axis font size" in category "Plot parameters" (plot-parameters/bin-axis-font-size) is set to **20**
- The parameter "Title" in category "Plot parameters" (plot-parameters/title) is set to **"Pearson's R green vs. magenta in STED"**
- The parameter "Value axis font size" in category "Plot parameters" (plot-parameters/value-axis-font-size) is set to **20**
- The parameter "Value axis label" in category "Plot parameters" (plot-parameters/value-axis-label) is set to **"Frequency"**
- The parameter "Color map" in category "Plot parameters" (plot-parameters/color-map) is set to **"Set1"**
- The parameter "Histogram type" in category "Plot parameters" (plot-parameters/histogram-type) is set to **"RelativeFrequency"**

**Node #364 "Histogram plot" of type "Histogram plot"**

- Input "Input" of node #364 receives data from output "Output" of node #346
- The parameter "Series name" (series-name) is set to **"M2: RED vs. ORANGE in STED"**
- The parameter item #1 of "Overridden parameters" in category "Adaptive parameters" (jipipe:adaptive-parameters/overridden-parameters) is set to **(#Subfolder, "plot-parameters/legend")**
- The parameter "Value" in category "Input columns" (input-columns/Value) is set to **("ExistingColumn", "Manders' tM2 (Above autothreshold of Ch1)")**
- The parameter "Bins" in category "Plot parameters" (plot-parameters/bins) is set to **20**
- The parameter "Bin axis label" in category "Plot parameters" (plot-parameters/bin-axis-label) is set to **"M2"**
- The parameter "Title" in category "Plot parameters" (plot-parameters/title) is set to **"Manders' tM2 green vs. magenta in STED"**
- The parameter "Value axis label" in category "Plot parameters" (plot-parameters/value-axis-label) is set to **"Frequency"**
- The parameter "Color map" in category "Plot parameters" (plot-parameters/color-map) is set to **"Set1"**
- The parameter "Histogram type" in category "Plot parameters" (plot-parameters/histogram-type) is set to **"RelativeFrequency"**

**Node #365 "Colocalization per component" of type "Compartment output"**

- Input "CONF vs STED, green" of node #365 receives data from output "Output" of node #340
- Input "CONF vs STED, magenta" of node #365 receives data from output "Output" of node #344
- Input "Green vs Magenta, CONF" of node #365 receives data from output "Output" of node #341
- Input "Green vs. Magenta, STED" of node #365 receives data from output "Output" of node #346
- Input "Histo Pearsons R green vs. magenta in STED" of node #365 receives data from output "Output" of node #363
- The parameter "jipipe:compartment:output-slot-name" (jipipe:compartment:output-slot-name) is set to **"Colocalization per component"**

## Compartment C6 "Saving"

- The "Saving" compartment (C6) receives data from the "Segmentation and colocalization" compartment (C2)
- The "Saving" compartment (C6) receives data from the "Filaments and colocalization in STED" compartment (C4)

**Node #366 "Export table" of type "Export table"**

- The parameter "File path" (file-path) is set to **PATH_COMBINE(project_data_dirs["Analysisfolder"], "_ColocCONF_Red_Orange")**

**Node #367 "Export image" of type "Export image"**

- The parameter "File path" (file-path) is set to **PATH_COMBINE(project_data_dirs["Analysisfolder"] , #Image + "_OverlayCONF_Red_Orange")**

**Node #368 "Export image" of type "Export image"**

- The parameter "File path" (file-path) is set to **PATH_COMBINE(project_data_dirs["Analysisfolder"] , #Image + "_OverlaySTED_Red_Orange")**

**Node #369 "Export image" of type "Export image"**

- The parameter "File path" (file-path) is set to **PATH_COMBINE(project_data_dirs["Analysisfolder"] , #Image + "_Overlay_CONFvsSTED_Red")**

**Node #370 "Export image" of type "Export image"**

- The parameter "File path" (file-path) is set to **PATH_COMBINE(project_data_dirs["Analysisfolder"] , #Image + "_Overlay_CONFvsSTED_Orange")**

**Node #129 "Output" of type "Compartment output"**

- Input "Coloc green CONF vs STED" of node #129 receives data from output "Output" of node #126
- Input "Coloc magenta CONF vs STED" of node #129 receives data from output "Output" of node #108
- Input "Coloc CONF green vs magenta" of node #129 receives data from output "Output" of node #100
- Input "Coloc STED RED vs ORANGE" of node #129 receives data from output "Output" of node #120
- Input "Overlay green vs. magenta in CONF" of node #129 receives data from output "Output" of node #104
- Input "Overlay green vs. magenta in STED" of node #129 receives data from output "Output" of node #122
- Input "Overlay CONF vs. STED in green" of node #129 receives data from output "Output" of node #121
- Input "Overlay CONF vs. STED in magenta" of node #129 receives data from output "Output" of node #107
- Input "green CONF" of node #129 receives data from output "Output" of node #81
- Input "RED STED" of node #129 receives data from output "Output" of node #113
- Input "ORANGE CONF" of node #129 receives data from output "Output" of node #82
- Input "ORANGE STED" of node #129 receives data from output "Output" of node #90
- Input "Merged RED and ORANGE ROIs in CONF" of node #129 receives data from output "Output" of node #99
- Input "M2 CONF vs. STED in RED" of node #129 receives data from output "Output" of node #127
- Input "M1 CONF vs. STED in RED" of node #129 receives data from output "Output" of node #128
- Input "M2 CONF vs. STED in ORANGE" of node #129 receives data from output "Output" of node #110
- Input "M1 CONF vs. STED in ORANGE" of node #129 receives data from output "Output" of node #111
- Input "M2 RED vs. ORANGE in CONF" of node #129 receives data from output "Output" of node #105
- Input "M1 RED vs. ORANGE in CONF" of node #129 receives data from output "Output" of node #106
- Input "M2 RED vs. ORANGE in STED" of node #129 receives data from output "Output" of node #124
- Input "M1 RED vs. ORANGE in STED" of node #129 receives data from output "Output" of node #125
- The parameter "jipipe:compartment:output-slot-name" (jipipe:compartment:output-slot-name) is set to **"Output"**

**Node #371 "Export table" of type "Export table"**

- Input "Input" of node #371 receives data from output "Coloc STED RED vs ORANGE" of node #129
- The parameter "File path" (file-path) is set to **PATH_COMBINE(project_data_dirs["Analysisfolder"], "_ColocSTED_Red_Orange")**

**Node #315 "Output" of type "Compartment output"**

- Input "Colocalization RED vs. ORANGE in STED negative ROIs" of node #315 receives data from output "Output" of node #307
- Input "Colocalization positive RED vs. negative ORANGE in STED" of node #315 receives data from output "Output" of node #308
- Input "Colocalization negative RED vs. positive ORANGE in STED" of node #315 receives data from output "Output" of node #306
- Input "Colocalization positive RED vs. positive ORANGE in STED" of node #315 receives data from output "Output" of node #278
- Input "Overlay of RED and ORANGE gaps" of node #315 receives data from output "Output" of node #303
- Input "Filament measurments RED STED continuous filaments" of node #315 receives data from output "Output" of node #292
- Input "Filament measurments RED STED filaments with gaps" of node #315 receives data from output "Output" of node #272
- Input "Filament measurments ORANGE STED continuous filaments" of node #315 receives data from output "Output" of node #300
- Input "Filament measurments ORANGE STED filaments with gaps" of node #315 receives data from output "Output" of node #275
- Input "Histo M2 RED vs. ORANGE in STED negative ROIs" of node #315 receives data from output "Output" of node #311
- Input "M1 RED vs. ORANGE in STED negative ROI" of node #315 receives data from output "Output" of node #312
- Input "M2 positive RED vs. negative ORANGE in STED" of node #315 receives data from output "Output" of node #314
- Input "M1 positive RED vs. negative ORANGE in STED" of node #315 receives data from output "Output" of node #310
- Input "M1 negative RED vs. positive ORANGE in STED" of node #315 receives data from output "Output" of node #309
- Input "M2 positive RED vs. positive ORANGE in STED" of node #315 receives data from output "Output" of node #282
- Input "M1 positive RED vs. positive ORANGE in STED" of node #315 receives data from output "Output" of node #283
- Input "Overlay of RED and ORANGE" of node #315 receives data from output "Output" of node #305
- The parameter "jipipe:compartment:output-slot-name" (jipipe:compartment:output-slot-name) is set to **"Output"**

**Node #372 "Export table" of type "Export table"**

- Input "Input" of node #372 receives data from output "Colocalization negative RED vs. positive ORANGE in STED" of node #315
- The parameter "File path" (file-path) is set to **PATH_COMBINE(project_data_dirs["Analysisfolder"] , "_ColocSTED_negRed_posOrange")**

**Node #373 "Export table" of type "Export table"**

- Input "Input" of node #373 receives data from output "Colocalization positive RED vs. positive ORANGE in STED" of node #315
- The parameter "File path" (file-path) is set to **PATH_COMBINE(project_data_dirs["Analysisfolder"], "_ColocSTED_posRed_posOrange")**

**Node #374 "Export table" of type "Export table"**

- Input "Input" of node #374 receives data from output "Filament measurments RED STED filaments with gaps" of node #315
- The parameter "File path" (file-path) is set to **PATH_COMBINE(project_data_dirs["Analysisfolder"], "_FilamentsSTED_Red")**

**Node #375 "Export table" of type "Export table"**

- Input "Input" of node #375 receives data from output "Filament measurments ORANGE STED filaments with gaps" of node #315
- The parameter "File path" (file-path) is set to **PATH_COMBINE(project_data_dirs["Analysisfolder"], "_FilamentsSTED_Orange")**

**Node #376 "Export image" of type "Export image"**

- Input "Input" of node #376 receives data from output "Overlay of RED and ORANGE" of node #315
- The parameter "File format" (file-format) is set to **"TIFF"**
- The parameter "File path" (file-path) is set to **PATH_COMBINE(project_data_dirs["Analysisfolder"] , #Image + "_Overlay_Red_Orange")**

**Node #377 "Output" of type "Compartment output"**

- Input "Coloc CONF red orange" of node #377 receives data from output "Exported file" of node #366
- Input "Coloc STED red orange" of node #377 receives data from output "Exported file" of node #371
- Input "Overlay CONF red orange" of node #377 receives data from output "Exported file" of node #367
- Input "ColocSTED_negRed_posOrange" of node #377 receives data from output "Exported file" of node #372
- Input "ColocSTED_posRed_posOrange" of node #377 receives data from output "Exported file" of node #373
- Input "OverlaySTED_Red_Orange" of node #377 receives data from output "Exported file" of node #368
- Input "FilamentsSTED_Red" of node #377 receives data from output "Exported file" of node #374
- Input "FilamentsSTED_Orange" of node #377 receives data from output "Exported file" of node #375
- Input "Overlay_CONFvsSTED_Red" of node #377 receives data from output "Exported file" of node #369
- Input "Overlay_CONFvsSTED_Orange" of node #377 receives data from output "Exported file" of node #370
- Input "Overlay_Red_Orange" of node #377 receives data from output "Exported file" of node #376
- The parameter "jipipe:compartment:output-slot-name" (jipipe:compartment:output-slot-name) is set to **"Output"**
